# Epitaxial-strain engineering of supported tensile noble metallene oxides for enhanced oxidation catalysis

**DOI:** 10.1038/s41467-026-78119-2

**Published:** 2026-10-13

**Authors:** Shoujie Zhang, Dong Lin, Anwen Yu, Haifeng Qi, Zhongyu Jiang, Xiuhui Zheng, Xiang Feng, Yongning Yang, Zhikang Fu, Sheng Dai, Lina Li, Aiyong Wang, Yanglong Guo, Wangcheng Zhan, Li Wang, Yun Guo, Xuan Tang

**Affiliations:** 1 State Key Laboratory of Green Chemical Engineering and Industrial Catalysis, Research Institute of Industrial Catalysis, School of Chemistry and Molecular Engineering, East China University of Science and Technology, Shanghai, P. R. China; 2 Max Planck-Cardiff Centre on the Fundamentals of Heterogeneous Catalysis FUNCAT, Cardiff Catalysis Institute, School of Chemistry, Cardiff University, Cardiff, UK; 3 State Key Laboratory of Heavy Oil Processing, China University of Petroleum, Qingdao, P. R. China; 4 Key Laboratory for Advanced Materials and Joint International Research Laboratory of Precision Chemistry and Molecular Engineering, Feringa Nobel Prize Scientist Joint Research Center, School of Chemistry and Molecular Engineering, East China University of Science and Technology, Shanghai, P. R. China; 5 Shanghai Synchrotron Radiation Facility, Shanghai Advanced Research Institute, Chinese Academy of Sciences, Shanghai, P. R. China

**Keywords:** Two-dimensional materials, Pollution remediation, Heterogeneous catalysis

## Abstract

Supported two-dimensional noble metal oxides (metallene oxides) hold great promise for heterogeneous catalysis owing to their maximized surface exposure and tunable electronic structures. However, their high surface energy and low oxidation potentials make them intrinsically unstable under reaction conditions. Here, we report an epitaxial strain strategy to stabilize and modulate the electronic structure of supported PtO_2_ metallene oxide. By employing rutile TiO_2_ and rutile SnO_2_ supports with distinct lattice parameters, we achieve epitaxial growth of PtO_2_ metallene oxide layers with controlled strain states, where the larger-lattice SnO_2_ substrate imposes biaxial tensile strain on PtO_2_. The tensile-strained PtO_2_/SnO_2_ exhibits a reconfigured Pt–O electronic environment that simultaneously improves structural resilience and enhances the intrinsic reactivity of PtO_2_, delivering markedly improved methane oxidation performance with a T_90_ of 370 °C (versus 434 °C for PtO_2_/TiO_2_ and 469 °C for PtO_x_/Al_2_O_3_) and <1% conversion loss over 38 h under both dry and wet conditions. Moreover, this epitaxial strain strategy is generalizable to other environmentally relevant oxidation reactions and rutile-type noble metal oxides, establishing tensile strain imposed by epitaxial lattice matching as a general principle for stabilizing atomically thin noble metal oxides while tuning their electronic structure.

## Abstract (web summary)

Supported two-dimensional noble-metal oxides (metallene oxides) are promising catalysts for heterogeneous catalysis, but their high surface energy and low oxidation potential make them intrinsically unstable under reaction conditions. Here, the authors develop an epitaxial-strain strategy to stabilize supported PtO₂ metallene oxides while tuning their electronic structure, thereby enhancing their catalytic performance for oxidation reactions.

## Introduction

Supported noble-metal catalysts have been extensively employed in heterogeneous catalysis, energy conversion, and chemical sensing owing to their high activity and durability^1,2^. Atomically dispersed noble-metal catalysts further maximize metal utilization, approaching 100%^3–6^. However, their rigid local coordination and the complete isolation of active sites often suppress cooperative effects between neighboring metal atoms, which can limit their catalytic performance in certain reactions^7–10^. Two-dimensional (2D) noble-metal materials (metallenes) have emerged as an attractive alternative^11,12^. Their atomically thin nature ensures nearly complete atom utilization and maximized active-site density, while also offering tunable and cooperative electronic structures, efficient mass transport, and unusual mechanical properties^13,14^. In practical industrial catalysis, however, most 2D noble-metal systems rely on strong interactions with supports, where interfacial coupling plays a crucial role in determining their geometric configuration and electronic state^15,16^. Constructing ordered atomic arrangements in such supported metallene systems not only maximizes noble-metal utilization but also enables precise modulation of their electronic structure, such as the *d*-band center, for rational catalyst design^17,18^.

Beyond metallic metallenes, noble-metal oxides represent an important but far less explored class of 2D catalytic materials^19,20^. Their flexible oxidation states and tunable M–O bond covalency facilitate efficient redox cycling and oxygen activation, providing inherent advantages for oxidation catalysis^21–24^. Nevertheless, synthesizing stable, supported 2D noble-metal oxides (metallene oxides) remains extremely challenging, as their high surface energy and low oxidation potentials make atomically thin oxide phases prone to structural collapse and reduction^25,26^. Our recent work showed that PtO_2_ atomic layers can epitaxially grow on rutile TiO_2_ owing to their matched lattice symmetry^27^. However, although such PtO_2_ layers can modulate the oxidation state of Pt species upon subsequent reduction, they remain metastable and structurally fragile under reaction conditions, thereby limiting their practical catalytic applicability.

Strain engineering has been proposed as a conceptual route to modulate metal-oxygen interactions and electronic states, thereby tuning both structural stability and catalytic reactivity^28–33^. Although epitaxial strain has been exploited in perovskite oxides and semiconductor thin films to tailor structural and electronic properties^34–36^, its application to supported noble-metal oxide catalysts has remained essentially unexplored, primarily because supported atomically thin noble-metal oxide phases are exceedingly difficult to stabilize and maintain under realistic reaction conditions. Whether such epitaxial strain can stabilize a metastable 2D PtO_2_ phase and meaningfully enhance its catalytic properties, however, has remained an open question.

Here, we demonstrate an epitaxial-strain strategy that addresses this challenge by stabilizing and tuning the electronic structure of supported two-dimensional noble-metal oxides, referred to as noble metallene oxides (MOs). Rutile SnO_2_, which possesses a slightly larger lattice constant than rutile PtO_2_ and TiO_2_^37^, provides a rational platform for imposing biaxial tensile strain on an epitaxially grown PtO_2_ layer. By employing rutile TiO_2_ and SnO_2_ as supports with distinct lattice parameters, we achieve the epitaxial growth of PtO_2_–MO layers with well-defined strain states, where the larger-lattice SnO_2_ substrate imposes pronounced tensile strain on the PtO_2_–MO layer. Atomic-scale characterization reveals that tensile strain induces a reconfiguration of the Pt–O electronic environment and the Pt *d*-electron states, thereby optimizing the redox balance between structural stability and catalytic activity. As a result, the tensile-strained PtO_2_/SnO_2_ catalyst exhibits markedly enhanced performance and durability in complete methane oxidation, surpassing both PtO_2_–MO on TiO_2_ and conventional Pt-based catalysts. Moreover, this epitaxial strain approach is broadly applicable to other oxidation reactions, such as toluene oxidation, as well as to other rutile-type noble metal oxides, including RuO_2_ and IrO_2_, underscoring the generality of strain engineering as a design paradigm for stabilizing and activating 2D oxidation catalysts.

## Results

### Synthesis and characterization of PtO_2_ metallene oxide

Both PtO_2_/SnO_2_ and PtO_2_/TiO_2_ catalysts were prepared through an impregnation-calcination method, previously reported for the epitaxial growth of PtO_2_ atomic layers on rutile TiO_2_. Pt(NO_3_)_2_ was selected as the Pt precursor because it enables epitaxial formation of PtO_2_ layers even at low Pt loadings. The growth likely proceeds via a Frank-van der Merwe layer-by-layer mechanism under oxidative conditions.^27^

**Fig. 1:**
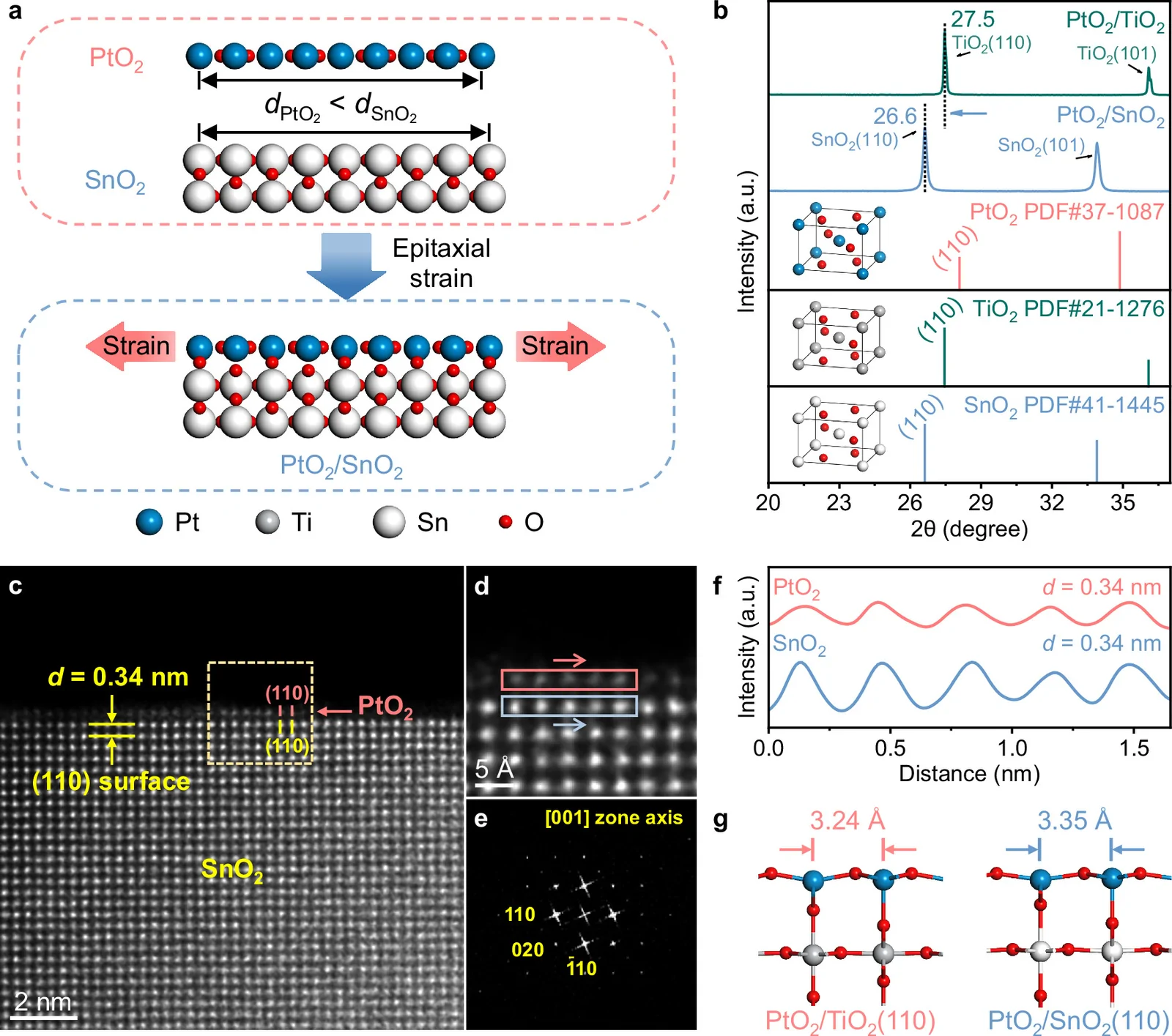
Epitaxial-strain construction and structural characterization of PtO_2_–MO. **a** Schematic illustration of the epitaxial strain strategy for the PtO_2_/SnO_2_ catalyst. **b** The XRD patterns of PtO_2_/SnO_2_, PtO_2_/TiO_2_, and the unit cell schematic illustration for PtO_2_, TiO_2_, and SnO_2_. **c** Representative low-damage HAADF-STEM image of PtO_2_/SnO_2_. **d** Enlarged atomic-scale HAADF-STEM image of the boxed region in (**c**), and **e** the corresponding FFT pattern. **f** Intensity profiles of PtO_2_ and SnO_2_ taken from the selected red and blue areas in (**d**). **g** Schematic illustration of the DFT-optimized lattice spacing of the (110) plane in PtO_2_/TiO_2_(110) and PtO_2_/SnO_2_(110), viewed from the front. The blue, gray, white, and red balls are Pt, Ti, Sn, and O atoms, respectively.

Inductively coupled plasma optical emission spectroscopy (ICP-OES) analysis confirmed similar Pt loadings of 0.73 wt% and 0.71 wt% for PtO_2_/SnO_2_ and PtO_2_/TiO_2_, respectively (Supplementary Table 1). X-ray diffraction (XRD) patterns (Fig. 1b) display only reflections corresponding to the respective rutile supports, with no detectable peaks assignable to Pt-containing phases. The absence of Pt-related diffraction features suggests that Pt species are highly dispersed and present at low concentrations, requiring further examination by advanced high-resolution structural characterization. Notably, the diffraction peak positions of both PtO_2_/SnO_2_ and PtO_2_/TiO_2_ are identical to those of their corresponding supports (Supplementary Figs. 1 and 2), indicating that the incorporation of Pt does not perturb the host lattice structure. As highlighted in Fig. 1b, the (110) reflection of rutile SnO_2_ appears at a smaller diffraction angle (26.6°) than that of rutile TiO_2_ (27.5°), consistent with the larger lattice parameter of SnO_2_ (Supplementary Table 2). Consequently, if PtO_2_ were to grow epitaxially on SnO_2_, the resulting layer would be expected to experience biaxial tensile lattice strain relative to that on TiO_2_, due to lattice matching with the larger lattice parameter of the SnO_2_ support (Fig. 1a).

High-angle annular dark-field scanning transmission electron microscopy (HAADF-STEM) image of the PtO_2_/SnO_2_ catalyst reveals no discernible Pt nanoparticles on the SnO_2_ support (Supplementary Fig. 3), consistent with the absence of Pt-related reflections in the XRD patterns (Fig. 1b). Given the electron-beam sensitivity of PtO_2_ layers, the low-damage HAADF-STEM imaging approach was employed to resolve the intrinsic structure of Pt species at the atomic scale while minimizing beam-induced damage^38^. Direct identification of Pt species by Z-contrast imaging is complicated by projection effects from the relatively thick SnO_2_ particles (~ 60 nm), despite the larger atomic number of Pt (*Z* = 78) than Sn (*Z* = 50). To circumvent this limitation, we exploited the differing beam stability of PtO_2_ and SnO_2_: whereas the SnO_2_ support remains structurally stable (Supplementary Fig. 4), PtO_2_ species are susceptible to beam irradiation-induced structural changes^27^. Upon prolonged electron-beam exposure, the outermost atomic layer on the SnO_2_ surface transformed into nanoparticles (Supplementary Figs. 5a, b and 6), which were subsequently identified as Pt species by energy-dispersive X-ray spectroscopy (EDS) elemental mapping (Supplementary Fig. 5c, d). These observations confirm the successful epitaxial growth of an atomically thin PtO_2_ layer on the SnO_2_ surface in the PtO_2_/SnO_2_ catalyst (Fig. 1c). This layer is only one Pt atom thick, while Pt atoms are laterally connected through oxygen bridges to form an extended two-dimensional Pt–O–Pt network. Given its two-dimensional nature, this atomically thin PtO_2_ layer is herein referred to as “PtO_2_ metallene oxide (PtO_2_–MO)”.

Figure 1d presents the enlarged HAADF-STEM image of the boxed region in Fig. 1c, which corresponds to the same region shown in Supplementary Fig. 5 and was rotated for display to facilitate comparison with the structural model, and Fig. 1e shows the corresponding fast Fourier transform (FFT) pattern. As highlighted in Fig. 1c–e, the (110) planes of the PtO_2_–MO are coherently aligned with the (110) planes of the SnO_2_ support along the [001] zone axis, owing to their identical rutile structure. The line intensity profile extracted from the selected region in Fig. 1d reveals comparable interplanar spacings of 0.34 nm for PtO_2_ and 0.34 nm for SnO_2_ (Fig. 1f). HAADF-STEM image simulations further show that, at low PtO_2_ coverage, the apparent Pt-column contrast is strongly attenuated by projection through the SnO_2_ slab, explaining why the surface Pt-containing layer in this low-loading PtO_2_/SnO_2_ catalyst does not appear brighter than the SnO_2_ support (Supplementary Figs. 7–10).

In comparison, HAADF-STEM imaging of PtO_2_/TiO_2_ (Supplementary Fig. 11), together with our previous findings, confirms the epitaxial growth of PtO_2_–MO on the TiO_2_ surface, exhibiting a smaller interplanar spacing of 0.32 nm for PtO_2_(110) and 0.32 nm for TiO_2_(110). Line-intensity profiles extracted from the marked regions in Supplementary Fig. 11c further indicate the matched projected periodicities between the PtO_2_ overlayer and the TiO_2_ support (Supplementary Fig. 11d). The slightly elongated lattice spacing of PtO_2_ on SnO_2_ relative to TiO_2_ corresponds to an approximately 6% expansion in the measured lattice spacing, as viewed along the [001] zone axis. This expansion originates from lattice matching with the larger lattice parameter of the SnO_2_ support during epitaxial growth. This result suggests that lattice expansion in the SnO_2_ support effectively induces tensile strain in the PtO_2_–MO through epitaxial strain engineering.

As a control, Pt deposited on non-rutile Al_2_O_3_ under the same preparation conditions forms PtO_x_ clusters rather than an epitaxially registered PtO_2_–MO overlayer (Supplementary Fig. 12), further supporting that PtO_2_–MO formation requires the rutile structural template and lattice matching rather than simple Pt–O coordination with the support.

### The role of lattice strain in tuning the electronic configuration of PtO_2_ metallene oxide

Variation in tensile strain is expected to spatially modulate Pt–O bond lengths, thereby altering the orbital overlap between Pt and O, and ultimately tuning the electronic structure and chemical properties of PtO_2_–MO. To elucidate these effects, we then conducted comparative characterizations and density functional theory (DFT) calculations on PtO_2_/SnO_2_ and PtO_2_/TiO_2_, aiming to establish the relationship between strain-induced structural distortion and Pt–O interaction.

**Fig. 2:**
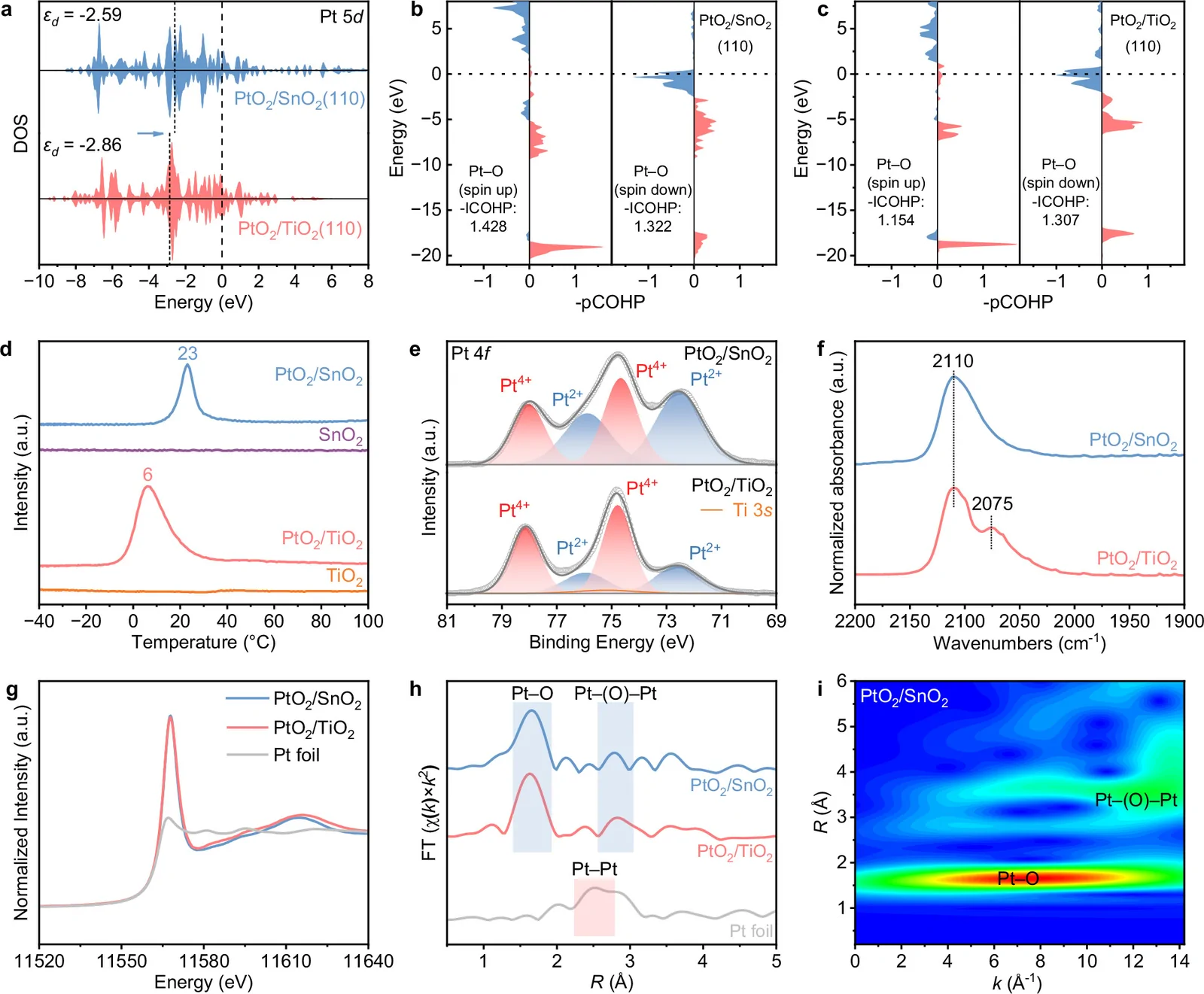
Electronic and local structural modulation of PtO_2_–MO by epitaxial strain. **a** Calculated density of states (DOS) of PtO_2_/TiO_2_(110) and PtO_2_/SnO_2_(110). **b**, **c** The pCOHPs of Pt–O bonds on the interface of (**b**) PtO_2_/SnO_2_(110) and (c) PtO_2_/TiO_2_(110). **d** H_2_-TPR profiles of PtO_2_/SnO_2_, SnO_2_, PtO_2_/TiO_2_ and TiO_2_. **e** XPS of Pt 4 *f* region for PtO_2_/SnO_2_ and PtO_2_/TiO_2_. **f** In situ DRIFTS of CO adsorbed at saturation coverage on PtO_2_/SnO_2_ and PtO_2_/TiO_2_. The spectra are normalized to the maximum intensity of the CO stretching band for each catalyst. **g** Normalized XANES spectra at the Pt *L*_3_-edge of PtO_2_/SnO_2_, PtO_2_/TiO_2_ and Pt foil. **h** FT-EXAFS spectra at the Pt *L*_3_-edge of PtO_2_/SnO_2_, PtO_2_/TiO_2_ and Pt foil. **i** WT-EXAFS contour plot of PtO_2_/SnO_2_ at the Pt *L*_3_-edge.

DFT calculations were first performed to investigate the impact of tensile strain on the orbital interactions between Pt and O atoms in PtO_2_ supported on SnO_2_ and rutile TiO_2_. Slab models of PtO_2_(110) epitaxially supported on SnO_2_(110) (PtO_2_/SnO_2_(110)) and TiO_2_(110) (PtO_2_/TiO_2_(110)) were constructed to simulate the experimentally obtained catalysts (Supplementary Fig. 13). As shown in Fig. 1g and Supplementary Fig. 14, the DFT-optimized structures reveal that the interplanar spacing of PtO_2_(110) expands from 3.18 Å to 3.35 Å and 3.24 Å on SnO_2_ and TiO_2_, respectively, in agreement with our STEM observations. As shown in Supplementary Fig. 15, the differential charge density plots indicate that the PtO_2_ in PtO_2_/SnO_2_(110) exhibits a much more anisotropic charge redistribution than that in PtO_2_/TiO_2_(110). Moreover, as shown in Fig. 2a, the calculated Pt *d*-band centers for PtO_2_/SnO_2_(110) and PtO_2_/TiO_2_(110) are located at −2.59 eV and −2.86 eV, respectively, indicating that the *d*-band center of PtO_2_/SnO_2_(110) lies closer to the Fermi level. These findings suggest that tensile strain in PtO_2_ on SnO_2_ modulates Pt–O orbital overlap, raises the *d*-band center, and consequently enhances the reactivity of the *d* electrons.

To further quantify the differences in interfacial Pt–O bonds, we calculated integrated crystal orbital Hamilton population (-ICOHP) values for chemical bonds at the PtO_2_/support interface (Fig. 2b, c and Supplementary Figs. 16 and 17). The Pt–O bonds of the PtO_2_/SnO_2_(110) interface (Fig. 2b) show a larger total |-ICOHP| (~ 2.750) than that of the PtO_2_/TiO_2_(110) interface (Fig. 2c), indicating a stronger net interfacial bonding interaction and thus a more robust anchoring of PtO_2_ on SnO_2_. Combined with the *d*-band upshift and the anisotropic charge redistribution, these results suggest that tensile strain and interfacial electronic coupling synergistically reconfigure the Pt–O electronic environment, enabling a favorable balance between stability and reactivity.

H_2_-temperature-programmed reduction (H_2_-TPR) experiments were conducted to probe the strength of Pt–O interactions. As shown in Fig. 2d, the PtO_2_/SnO_2_ catalyst exhibits a distinct reduction peak at 23 °C, corresponding to the reduction of PtO_2_. In contrast, PtO_2_/TiO_2_ displays the PtO_2_ reduction peak at a lower temperature of 6 °C, suggesting that PtO_2_–MO on SnO_2_ is more resistant to reduction. To further compare the electronic structures and chemical states of Pt species in PtO_2_/SnO_2_ and PtO_2_/TiO_2_, X-ray photoelectron spectroscopy (XPS) was carried out for both catalysts. The high-resolution Pt 4 *f* spectra of both PtO_2_/SnO_2_ and PtO_2_/TiO_2_ (Fig. 2e) can be deconvoluted into four components corresponding to Pt^2+^ 4*f*_7/2_, Pt^4+^ 4*f*_7/2_, Pt^2+^ 4*f*_5/2_, and Pt^4+^ 4*f*_5/2_, located at 72.6 ± 0.1, 74.7 ± 0.1, 75.9 ± 0.1, and 78.0 ± 0.1 eV, respectively. PtO_2_/SnO_2_ shows relative proportions of Pt^2+^ (49.6%) and Pt^4+^ (50.4%) (Supplementary Table 3). The lower proportion of Pt^4+^ relative to PtO_2_/TiO_2_ (68.3%) suggests a strain-induced redistribution of electron density at the Pt centers, consistent with the DFT results showing that tensile strain shifts the Pt *d* states closer to the Fermi level and enhances the reactivity of Pt *d* electrons^28^. In addition, the nearly identical ultraviolet photoelectron spectroscopy (UPS)-derived work functions of SnO_2_ and TiO_2_ suggest that support-energy-alignment effects are limited and are unlikely to be the dominant origin of the observed Pt electronic difference (Supplementary Fig. 18).

CO-probe diffuse reflectance infrared Fourier transform spectroscopy (DRIFTS) was performed at 6 °C to further elucidate the role of lattice strain in modulating the electronic state of Pt. As shown in Fig. 2f, the normalized DRIFTS of CO adsorbed at saturation coverage reveal a dominant stretching band at 2110 cm^−1^ for PtO_2_/SnO_2_, characteristic of CO bound to high-valent Pt species^39,40^. A similar feature at 2110 cm^−1^ is also observed for PtO_2_/TiO_2_. However, in the latter case, an additional distinct band emerges at 2075 cm^−1^, attributable to CO adsorption on metallic Pt^41^. The absence of Pt^0^ signals in the XPS of PtO_2_/TiO_2_ (Fig. 2e) indicates that partial reduction of Pt species likely occurs upon CO exposure under DRIFTS conditions. This is consistent with the fact that PtO_2_ can undergo partial reduction at similar temperatures under H_2_, as evidenced by the H_2_-TPR profile (Fig. 2d). In contrast, no such reduction is evident for PtO_2_/SnO_2_. These results indicate that PtO_2_–MO supported on SnO₂ exhibits a markedly higher resistance to reduction than its TiO_2_-supported counterpart, consistent with the reconfiguration of the Pt–O electronic environment revealed by both spectroscopic and theoretical analyses.

X-ray absorption spectroscopy (XAS) was further performed to obtain sample-averaged structural and electronic information on the PtO_2_–MO catalysts. Figure 2g presents the Pt *L*_3_-edge X-ray absorption near-edge structure (XANES) spectra of PtO_2_/SnO_2_ and PtO_2_/TiO_2_. The two catalysts show broadly similar XANES profiles, and both exhibit enhanced white-line intensities relative to Pt foil, consistent with the oxidized nature of Pt species in the two catalysts. The Fourier transform (FT) of the k^2^-weighted extended X-ray absorption fine-structure (EXAFS) spectra (Fig. 2h) of both PtO_2_/SnO_2_ and PtO_2_/TiO_2_ shows a dominant first-shell peak at ≈1.7 Å (before phase correction), corresponding to Pt–O coordination, and a weaker higher-shell peak at ≈2.8 Å, assigned to Pt–(O)–Pt scattering. The Pt–(O)–Pt backscattering assignment is further supported by wavelet transform (WT) analysis of PtO_2_/SnO_2_, which shows a maximum WT intensity at k ≈ 12 Å^−1^, characteristic of scattering from a heavy Pt backscatterer (Fig. 2i). Quantitative EXAFS fitting gives a Pt–O coordination number of 4.1 ± 0.3 with a Pt–O distance of 1.99 ± 0.01 Å, and a Pt–(O)–Pt distance of 3.12 ± 0.02 Å for PtO_2_/SnO_2_ (Supplementary Fig. 19 and Supplementary Table 4). In comparison, PtO_2_/TiO_2_ exhibits a similar Pt–O distance of 2.00 ± 0.02 Å and a shorter fitted Pt–(O)–Pt distance of 3.04 ± 0.06 Å, indicating a support-dependent trend in the higher-shell Pt–(O)–Pt geometry, although the higher-shell contribution is weaker and associated with larger uncertainty (Supplementary Fig. 20 and Supplementary Table 4). This expanded Pt–(O)–Pt geometry in PtO_2_/SnO_2_ is consistent with tensile distortion of the epitaxial PtO_2_–MO overlayer on the SnO_2_ support. Consequently, the local electronic environment of Pt in PtO_2_–MO on SnO_2_ is distinct from that on TiO_2_, with potential implications for its catalytic reactivity.

### Catalytic performance in methane oxidation

The epitaxial strain-induced reconfiguration of the Pt–O electronic environment is expected to modulate the redox properties of PtO_2_–MO, which are critical for oxidation catalysis. To assess how such strain-induced electronic modifications translate into catalytic behavior, we investigated the complete oxidation of methane under lean-burn conditions, a prototypical structure-sensitive reaction^42,43^. For comparison, an Al_2_O_3_-supported Pt catalyst (PtO_x_/Al_2_O_3_) was used as a control sample.

**Fig. 3:**
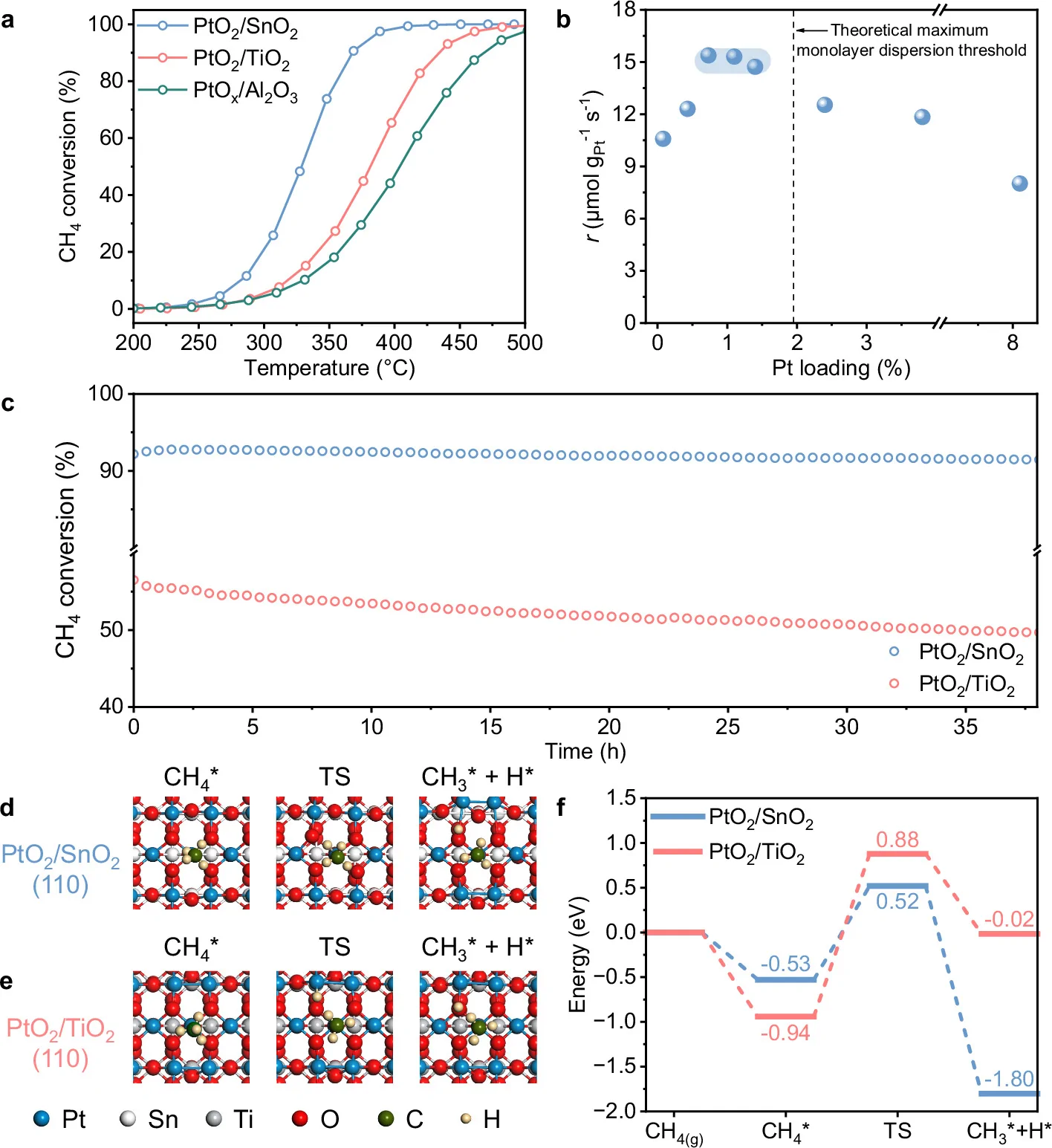
Methane oxidation performance and C–H activation of PtO_2_–MO. **a** The light-off curves of methane oxidation of PtO_2_/SnO_2_, PtO_2_/TiO_2_, and PtO_x_/Al_2_O_3_. **b** Pt-mass-normalized methane oxidation rates over PtO_2_/SnO_2_ catalysts with different Pt loadings at 260 °C. **c** The long-term stability of PtO_2_/SnO_2_ and PtO_2_/TiO_2_ during methane oxidation at 385 °C. **d**, **e** Schematic illustration of the cleavage of the first C–H bond in CH_4_ on (d) PtO_2_/SnO_2_(110) and (e) PtO_2_/TiO_2_(110). **f** The reaction energy profiles for the cleavage of the first C–H bond in CH_4_ on PtO_2_/SnO_2_(110) and PtO_2_/TiO_2_(110).

The catalytic performance of all catalysts in complete methane oxidation was evaluated in successive cycles from 150 to 500 °C under lean-burn conditions. All the supports exhibited negligible activity below 350 °C (Supplementary Figs. 21–23). As shown in Fig. 3a, the PtO_2_/TiO_2_ catalyst displayed slightly higher activity than the PtO_x_/Al_2_O_3_ catalyst, reaching T_90_ values (the temperature at which 90% methane conversion was achieved) of ~434 and 469 °C, respectively. In contrast, the PtO_2_/SnO_2_ catalyst showed a markedly enhanced activity, achieving T_90_ at 370 °C. Notably, Pd-based catalysts are generally regarded as the most active materials for methane oxidation under lean-burn conditions^44,45^. However, unlike PdO, conventional PtO_2_ species formed during methane oxidation are thermally unstable and readily decompose into metallic Pt^0^ at relatively low temperatures (< 400 °C), resulting in significantly lower activity of monometallic Pt catalysts under lean-burn conditions^46,47^. Remarkably, the PtO_2_/SnO_2_ catalyst outperforms most of the state-of-the-art Pt-based catalysts reported in the literature (Supplementary Table 5). Its activity is even comparable to Pd-based benchmarks, as evidenced by the nearly identical methane conversion profiles of PtO_2_/SnO_2_ and homemade Pd/Al_2_O_3_ and Pd/SnO_2_ catalysts with slightly higher noble-metal loadings (~ 0.9 wt%; Supplementary Table 6) under identical lean-burn conditions (Supplementary Fig. 24).

In addition, the PtO_2_/SnO_2_ catalyst also exhibited excellent catalytic stability during methane oxidation. As shown in Supplementary Fig. 25, the light-off curves of methane conversion for PtO_2_/SnO_2_ remained essentially unchanged over three consecutive reaction cycles, with T_90_ consistently maintained at 370 °C. The light-off curves of methane conversion for PtO_2_/TiO_2_ and PtO_x_/Al_2_O_3_ exhibited a slight decline under identical conditions (Supplementary Figs. 26 and 27). Regarding long-term stability (Fig. 3c), the PtO_2_/TiO_2_ catalyst underwent noticeable deactivation within 38 h, with methane conversion decreasing from 56 to 49% at 385 °C. In contrast, PtO_2_/SnO_2_ displayed exceptional durability, showing less than a 1% drop in conversion under identical conditions. When water vapor was introduced, the PtO_2_/SnO_2_ catalyst also maintained comparable stability for 38 h, indicating its resistance to water-induced deactivation (Supplementary Fig. 28). Post-reaction characterization supports that PtO_2_–MO preserved its atomic monolayer structure and electronic states on the SnO_2_ surface after catalysis (Supplementary Figs. 29–34), evidencing high structural stability under operating conditions. Additional harsh air-calcination tests further indicate that metallic Pt starts to appear only after treatment at 700 °C (Supplementary Fig. 35). In contrast, the coordination environment of the PtO_2_–MO on TiO_2_ exhibited slight changes after the methane oxidation reaction (Supplementary Figs. 36 and 37), which may account for the minor decline in activity observed over three consecutive reaction cycles. This superior stability of the PtO_2_–MO on SnO_2_, relative to its TiO_2_-supported counterpart, can be attributed to the tensile strain within the PtO_2_–MO layer, which induces an optimal reconfiguration of the Pt–O electronic environment and thereby enhances stability at elevated reaction temperatures.

To elucidate whether the active sites in PtO_2_/SnO_2_ mainly originate from the PtO_2_–MO layer itself, or from interfacial sites, a series of PtO_2_/SnO_2_ catalysts with varying Pt loadings was then synthesized and evaluated through kinetic measurements at 260 °C. As shown in Fig. 3b, the reaction rate of methane oxidation gradually increased from 10.6 to 15.4 μmol g_Pt_^−1^ s^−1^ as the Pt loading was raised from 0.08 wt% to 0.73 wt%. At the ultra-low loading (0.08 wt%), the amount of Pt deposited on the SnO_2_ surface is expected to be atomically dispersed, resulting in insufficient surface coverage to form a laterally connected PtO_2_ layer. Therefore, the observed enhancement can be attributed to the transition from atomically dispersed Pt species at 0.08 wt% to the formation of laterally connected PtO_2_–MO domains with sufficient surface coverage as the Pt loading increases. Upon further increasing the Pt loading, the reaction rate remained nearly constant, reaching a plateau at 15 μmol g_Pt_^−1^ s^−1^ within the 0.73–1.40 wt% range (highlighted by the blue region in Fig. 3b). Such a plateau behavior strongly indicates that the intrinsic highly active sites arise from the surface PtO_2_–MO layer rather than Pt–O–Sn interfacial sites. At higher loadings (2.40–8.10 wt%), the reaction rate decreased slightly from 12.5 to 8.0 μmol g_Pt_^−1^ s^−1^, primarily due to the formation of multilayered PtO_2_ domains on SnO_2_ (Supplementary Figs. 38–43) once the Pt loading exceeded the theoretical maximum dispersion threshold for a PtO_2_ monolayer on SnO_2_ (1.95 wt%) (Fig. 3b and Supplementary Fig. 38). These findings reveal that the epitaxially strained PtO_2_–MO monolayer serves as the intrinsic highly active phase for methane oxidation, highlighting strain-stabilized 2D noble-metal oxides as a general paradigm for designing efficient oxidation catalysts.

The enhanced reactivity of the tensile-strained PtO_2_–MO in methane oxidation was further corroborated by DFT calculations. The apparent reaction orders of methane (0.51) and oxygen (−0.05) for PtO_2_/SnO_2_ indicate that the reaction rate is primarily governed by methane activation and is insensitive to oxygen partial pressure (Supplementary Figs. 44 and 45), consistent with a Mars-van Krevelen mechanism involving lattice oxygen participation. In this scenario, the dissociation of the first C–H bond is considered the rate-limiting step^43^; therefore, the corresponding activation barrier was compared by DFT to assess reactivity. Reaction energy profiles and optimized structures for adsorbed CH_4_*, transition states (TSs), and final states (CH_3_* + H*) were computed (Fig. 3d, e). As shown in Fig. 3f, the activation barrier for methane dissociation is significantly lower on PtO_2_/SnO_2_(110) (1.0 eV) than on PtO_2_/TiO_2_(110) (1.8 eV), demonstrating that tensile strain on the PtO_2_ surface substantially promotes C–H bond activation.

### General applicability of epitaxial strain engineering

**Fig. 4:**
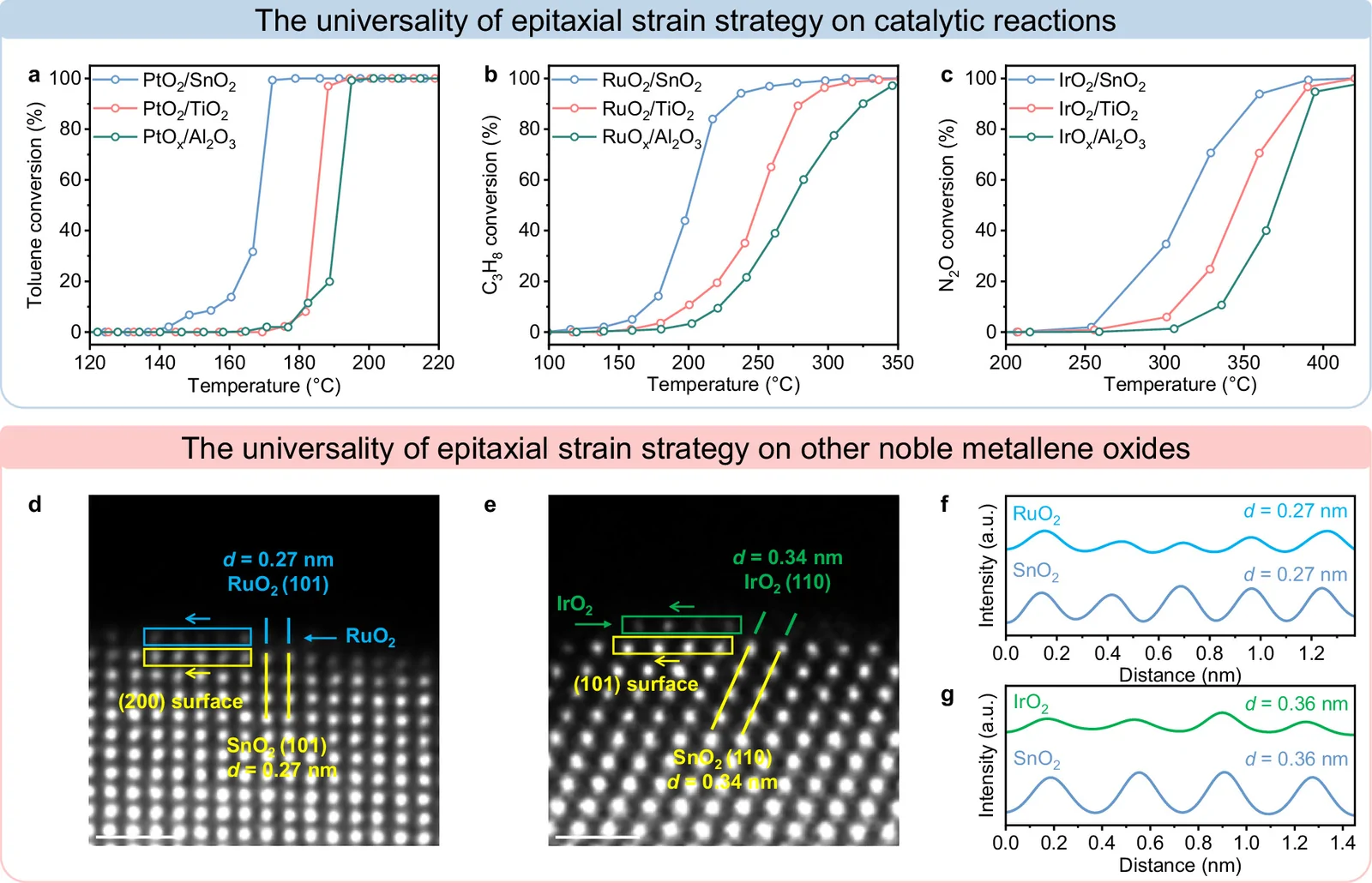
The universality of the epitaxial strain strategy across catalytic reactions and other noble-metal metallene oxides. **a** The light-off curves of toluene oxidation of PtO_2_/SnO_2_, PtO_2_/TiO_2_, and PtO_x_/Al_2_O_3_. **b** The light-off curves of C_3_H_8_ oxidation of RuO_2_/SnO_2_, RuO_2_/TiO_2_, and RuO_x_/Al_2_O_3_. **c** The light-off curves of N_2_O decomposition of IrO_2_/SnO_2_, IrO_2_/TiO_2_, and IrO_x_/Al_2_O_3_. **d**, **e** The representative atomic-scale HAADF-STEM images of **d** RuO_2_/SnO_2_ catalyst and **e** IrO_2_/SnO_2_ catalyst. Scale bar: 1 nm for (**d**, **e**). **f** Intensity profiles of RuO_2_ and SnO_2_ taken from the selected blue and yellow areas in (**d**). **g** Intensity profiles of IrO_2_ and SnO_2_ taken from the selected green and yellow areas in (**e**).

Furthermore, we extended the applicability of the epitaxial strain engineering strategy to other oxidation reactions, exemplified by toluene oxidation, which is representative of aromatic hydrocarbon VOCs. All the supports exhibited negligible activity below 220 °C for toluene oxidation (Supplementary Figs. 46–48). As shown in Fig. 4a, the PtO_2_/SnO_2_ catalyst exhibited a pronounced enhancement in toluene oxidation activity compared to PtO_2_/TiO_2_ and PtOₓ/Al_2_O_3_, lowering the T_90_ from 187 °C and 194 °C to 171 °C. In addition, the PtO_2_/SnO_2_ catalyst also exhibited excellent long-term stability for toluene oxidation under both dry and wet conditions (Supplementary Fig. 49). These results demonstrate that epitaxial strain engineering not only enhances the catalytic performance for methane oxidation but also effectively promotes other oxidation reactions, such as toluene oxidation.

More importantly, this epitaxial strain strategy is not limited to PtO_2_ systems. To further probe its generality across different noble-metal oxides and environmentally relevant oxidation reactions, we selected RuO_2_ and IrO_2_ as representative rutile-type oxides, which are well-known catalysts for alkane oxidation^48^ and N_2_O decomposition^49^, respectively. Similar tensile strain effects were observed for both RuO_2_ and IrO_2_ when epitaxially grown on SnO_2_ (Supplementary Figs. 50–55). The epitaxial layers of RuO_2_ and IrO_2_ on the (200) and (101) surfaces of rutile SnO_2_, respectively, were directly visualized at the atomic scale using low-damage HAADF-STEM (Fig. 4d, e). The (101) planes of RuO_2_ (0.27 nm) and the (110) planes of IrO_2_ (0.34 nm) exhibit excellent lattice matching with the corresponding (101) (0.27 nm) and (110) (0.34 nm) planes of SnO_2_, which are slightly larger than those of the layers epitaxially grown on rutile TiO_2_ (0.25 nm for (101) and 0.32 nm for (110))^27,50^. Line-intensity profiles extracted from the marked regions in Fig. 4d, e further confirm the closely matched projected periodicities between RuO_2_ (Fig. 4f) or IrO_2_ (Fig. 4g) and the SnO_2_ support. These findings indicate that epitaxial growth on the larger-lattice-parameter SnO_2_ support can universally induce tensile strain across different rutile-type metallene oxides.

Notably, although all the supports exhibited negligible activity below 300 °C for propane oxidation and N_2_O decomposition (Supplementary Figs. 56–61), the tensile-strained RuO_2_/SnO_2_ and IrO_2_/SnO_2_ catalysts showed markedly enhanced catalytic performance compared with their TiO_2_-supported counterparts and conventional Al_2_O_3_-supported references. Specifically, the T_90_ for propane oxidation decreased from 281 °C on RuO_2_/TiO_2_ to 226 °C on RuO_2_/SnO_2_, while the T_90_ for N_2_O decomposition was reduced from 383 °C on IrO_2_/TiO_2_ to 355 °C on IrO_2_/SnO_2_, as shown in Fig. 4b, c, respectively. Consistently, both catalysts also outperformed the corresponding Al_2_O_3_-supported references under identical conditions, giving T_90_ values of 326 °C for propane oxidation on RuO_x_/Al_2_O_3_ and 392 °C for N_2_O decomposition on IrO_x_/Al_2_O_3_. These results highlight the versatility of epitaxial strain engineering as a general platform for optimizing environmentally relevant oxidation catalysis across different noble metal oxides.

## Discussion

In summary, we have demonstrated a general epitaxial strain engineering strategy to stabilize and modulate MOs on rutile oxide supports. By employing supports with distinct lattice parameters, biaxial tensile strain is introduced into PtO_2_–MO epitaxially grown on SnO_2_, leading to a reconfiguration of the Pt–O electronic environment and Pt *d*-electron states that optimizes the balance between structural stability and redox reactivity. As a result, the strained PtO_2_–MO monolayer exhibits exceptional activity and durability in complete methane oxidation, outperforming both PtO_2_–MO on TiO_2_ and conventional Pt-based catalysts. Kinetic analysis indicates that the intrinsically highly active phase originates from the epitaxially strained PtO_2_–MO itself. Moreover, this epitaxial strain strategy extends to other oxidation systems, such as toluene oxidation, and to other rutile-type noble-metal oxides, including RuO_2_ and IrO_2_, underscoring its generality. This work establishes strain-stabilized 2D metallene oxides as a versatile platform for the rational design of advanced catalysts for environmentally relevant oxidation reactions. Given the broad technological relevance of noble-metal/SnO_2_ materials in thermal catalysis^51,52^, electrocatalysis^53,54^, photocatalysis^55^, chemical sensing^56,57^, among others^58^, the epitaxial strain concept established here offers a versatile framework for tuning interfacial interactions and electronic structures across a wide range of supported material applications.

## Methods

### Chemicals and materials

All reagents and solvents were commercially obtained and used without further purification. Platinum nitrate (Pt(NO_3_)_2_), palladium nitrate (Pd(NO_3_)_2_), ruthenium nitrate (Ru(NO_3_)_3_), and iridium acetate (Ir(CH_3_COO)_3_) were purchased from the Kunming Institute of Precious Metals. Tin dioxide (SnO_2_) and titanium dioxide (TiO_2_, Rutile) were purchased from Shanghai Aladdin Biochemical Technology Co., Ltd. Aluminum oxide (Al_2_O_3_) was purchased from SASOL. Deionized water was obtained from a Milli-Q integral water purification system (Millipore, 18.2 MΩ cm).

### Synthesis of catalysts

The catalysts were prepared by a facile wet impregnation method. Typically, a certain amount of Pt(NO_3_)_2_ was added to SnO_2_ or other supports (e.g., TiO_2_, Al_2_O_3_), followed by continuous ultrasonic stirring for 30 min. After aging for 6 h, the mixture was transferred to the oven at 80 °C overnight. Unless otherwise stated, the freshly prepared catalysts were calcined in air at 500 °C for 2 h. The PtO_2_/SnO_2_, PtO_2_/TiO_2_, and PtO_x_/Al_2_O_3_ catalysts were prepared following the same protocol to minimize the interference from synthetic conditions.

### Evaluation of catalytic performance

The catalytic activity of the catalyst for complete methane oxidation was evaluated in a quartz fixed-bed reactor at atmospheric pressure, and 100 mg of catalyst (40–60 mesh) was used. The feed gas with 1 vol% CH_4_, 20 vol% O_2_, balanced with N_2_, passed through the catalyst bed at a flow rate of 50 mL min^−1^. To record light-off curves, the temperature of the catalyst bed was programmed from 150 to 500 °C at a rate of 4 °C min^−1^. The conversion of methane was measured by an online gas chromatograph (Agilent GC 7890A) equipped with a flame ionization detector (FID). The selectivity of CO_2_ was close to 100% over the catalysts under CH_4_ lean conditions, and the other possible products were below the detection limit of the GC. The CH_4_ conversion (${X}_{{{\mbox{CH}}}_{4}}$) was calculated by the following equation:

1 $${X}_{{{\mbox{CH}}}_{4}}=\frac{{[{c}_{{{\mbox{CH}}}_{4}}]}_{{\mbox{in}}}{-[{c}_{{{\mbox{CH}}}_{4}}]}_{{\mbox{out}}}}{{[{c}_{{{\mbox{CH}}}_{4}}]}_{{\mbox{in}}}}\times 100\%$$

where ${[{c}_{{{\mbox{CH}}}_{4}}]}_{{\mbox{in}}}$ and ${[{c}_{{{\mbox{CH}}}_{4}}]}_{{\mbox{out}}}$ are the CH_4_ concentrations in the inlet and outlet gases, respectively.

The kinetic measurements were performed at 260 °C, and the space velocity was adjusted with a feed gas of 1 vol% CH_4_, 20 vol% O_2_, and N_2_ balance to control the CH_4_ conversion to lower than 15% in order to eliminate the thermal and diffusion effects. The kinetic reaction data were obtained after 30 min of stable reaction. The reaction rate (*r*) of CH_4_ was calculated by the equation:

2 $${r}_{{{\mbox{CH}}}_{4}}=\frac{{c}_{{{\mbox{CH}}}_{4}}{X}_{{{\mbox{CH}}}_{4}}V\times {10}^{6}}{{m}_{{\mbox{Pt}}}}({\upmu} {{\rm{mol}}}{{{\mbox{g}}}_{{\mbox{Pt}}}}^{-1}{{\mbox{s}}}^{-1})$$

where ${c}_{{{\mbox{CH}}}_{4}}$ (mol mL^−1^) is the concentration of CH_4_ at the reactor inlet, ${X}_{{{\mbox{CH}}}_{4}}$ is the conversion of CH_4_, *V* (mL s^−1^) is the total flow rate, and *m*_Pt_ (g) is the mass of Pt contained in the catalyst used for the measurement.

The turnover frequency (*TOF*, s^−1^) was calculated using the following formula:

3 $${TOF}=\frac{{r}_{{{\mbox{CH}}}_{4}}\times {{10}^{-6}M}_{{\mbox{Pt}}}}{{D}_{{\mbox{Pt}}}}({{\mbox{s}}}^{-1})$$

where ${r}_{{{\mbox{CH}}}_{4}}$ is the reaction rate of CH_4_, ${M}_{{\mbox{Pt}}}$ is the molar mass of Pt, with a value of 195.08 g mol^−1^, ${D}_{{\mbox{Pt}}}$ is the dispersion of Pt.

The catalytic activity of the catalyst for complete toluene oxidation was evaluated in a quartz fixed-bed reactor at atmospheric pressure, and 100 mg (50 mg for stability test) of catalyst (40–60 mesh) was used. The feed gas with 1000 ppm toluene and 20 vol% O_2_, balanced with N_2_, passed through the catalyst bed at a flow rate of 50 mL min^−1^. To record light-off curves, the temperature of the catalyst bed was programmed from 100 to 300 °C at a rate of 0.83 °C min^−1^. The conversion of toluene was measured by an online gas chromatograph (RUINENG GC3900C) equipped with a flame ionization detector (FID). The toluene conversion (${X}_{{\mbox{toluene}}}$) was calculated by the following equation:

4 $${X}_{{\mbox{toluene}}}=\frac{{[{c}_{{\mbox{toluene}}}]}_{{\mbox{in}}}{-[{c}_{{\mbox{toluene}}}]}_{{\mbox{out}}}}{{[{c}_{{\mbox{toluene}}}]}_{{\mbox{in}}}}\times 100\%$$

where ${[{c}_{{\mbox{toluene}}}]}_{{\mbox{in}}}$ and${[{c}_{{\mbox{toluene}}}]}_{{\mbox{out}}}$ are the toluene concentrations in the inlet and outlet gases, respectively.

The catalytic activity of the catalyst for complete propane oxidation was evaluated in a quartz fixed-bed reactor at atmospheric pressure, and 100 mg of catalyst (40–60 mesh) was used. The feed gas with 2000 ppm C_3_H_8_ and 20 vol% O_2_, balanced with N_2_, passed through the catalyst bed at a flow rate of 50 mL min^−1^. To record light-off curves, the temperature of the catalyst bed was programmed from 100 to 400 °C at a rate of 2.5 °C min^−1^. The conversion of propane was measured by an online gas chromatograph (GC-9790) equipped with a flame ionization detector (FID). The C_3_H_8_ conversion (${X}_{{{\mbox{C}}}_{3}{{\mbox{H}}}_{8}}$) was calculated by the following equation:

5 $${X}_{{{{\mbox{C}}}_{3}{\mbox{H}}}_{8}}=\frac{{[{c}_{{{\mbox{C}}}_{3}{{\mbox{H}}}_{8}}]}_{{\mbox{in}}}{-[{c}_{{{\mbox{C}}}_{3}{{\mbox{H}}}_{8}}]}_{{\mbox{out}}}}{{[{c}_{{{\mbox{C}}}_{3}{{\mbox{H}}}_{8}}]}_{{\mbox{in}}}}\times 100\%$$

where ${[{c}_{{{\mbox{C}}}_{3}{{\mbox{H}}}_{8}}]}_{{\mbox{in}}}$ and ${[{c}_{{{\mbox{C}}}_{3}{{\mbox{H}}}_{8}}]}_{{\mbox{out}}}$ are the C_3_H_8_ concentrations in the inlet and outlet gases, respectively.

The catalytic activity of the catalyst for N_2_O decomposition was evaluated in a quartz fixed-bed reactor at atmospheric pressure, and 200 mg of catalyst (40–60 mesh) was used. The feed gas with 1500 ppm N_2_O and 4 vol% O_2_, balanced with N_2_, passed through the catalyst bed at a flow rate of 50 mL min^−1^. To record light-off curves, the temperature of the catalyst bed was programmed from 150 to 500 °C at a rate of 2 °C min^−1^. The conversion of N_2_O was measured by an online gas chromatograph (GC-9790) equipped with a thermal conductivity detector (TCD). The N_2_O conversion (${X}_{{{\mbox{N}}}_{2}{\mbox{O}}}$) was calculated by the following equation:

6 $${X}_{{{\mbox{N}}}_{2}{\mbox{O}}}=\frac{{[{c}_{{{\mbox{N}}}_{2}{\mbox{O}}}]}_{{\mbox{in}}}{-[{c}_{{{\mbox{N}}}_{2}{\mbox{O}}}]}_{{\mbox{out}}}}{{[{c}_{{{\mbox{N}}}_{2}{\mbox{O}}}]}_{{\mbox{in}}}}\times 100\%$$

where ${[{c}_{{{\mbox{N}}}_{2}{\mbox{O}}}]}_{{\mbox{in}}}$ and ${[{c}_{{{\mbox{N}}}_{2}{\mbox{O}}}]}_{{\mbox{out}}}$ are the N_2_O concentrations in the inlet and outlet gases, respectively.

### Catalyst characterization

X-ray powder diffraction (XRD) patterns were obtained from a Bruker D8 focus diffraction spectrometer using Cu Kα radiation (1.54056 Å, 40 kV, and 30 mA), scanning from 10 to 80° at a speed of 4° min^−1^. The specific surface areas of the catalysts were measured using a Micromeritics ASAP 2400 accelerated surface area and porosimetry system at 77 K and calculated using the Brunauer−Emmett−Teller (BET) method. An inductively coupled plasma optical emission spectrometer (ICP-OES) was used to quantify the actual loading of Pt in the catalysts on the Agilent 725ES. Aberration-corrected scanning transmission electron microscopy (AC-STEM) was operated on a ThermoFisher Themis Z transmission electron microscope equipped with two aberration correctors, operated at 300 kV with a convergence semi-angle of 17.8 mrad. The microscope was aligned using a standard gold sample before observations. High-angle annular dark-field (HAADF)-STEM images were recorded using a camera length of 115 mm on HAADF detectors, and the inner- and outer collection angles of 47 and 200 mrad, respectively. The acquired images were processed using the Gaussian blur filter. X-ray absorption (XAS) experiments at the Pt *L*_3_-edge were carried out at the beamline BL14W of the Shanghai Synchrotron Radiation Facility (SSRF). The spectra were collected in fluorescence mode. The XANES and EXAFS data were processed and analyzed with ATHENA and ARTEMIS software. H_2_-temperature-programmed reduction (H_2_-TPR) was carried out on a TPDRO 1100 (CE Instruments) with a thermal conductivity detector (TCD). 50 mg of catalyst was filled into the reactor and then directly heated from −50 to 800 °C at a rate of 10 °C min^−1^ in a flow of 5 vol.% H_2_/N_2_ (50 mL min^−1^). X-ray photoelectron spectroscopy (XPS) was performed using a Thermo ESCALAB 250Xi spectrometer. For the quantitative analysis, the raw XPS spectra were fitted using the CASA XPS software. Diffuse Reflectance Infrared Fourier Transform Spectroscopy (DRIFTS) of CO adsorption on catalysts was measured on a Nicolet Nexus 6700 spectrometer. The DRIFTS were collected with a resolution of 4 cm^−1^ and 64 scans in Kubelka–Munk units. Before CO adsorption, the samples were pretreated by Ar at 110 °C for 1 h. The CO adsorption on various catalysts was measured at 6 °C.

### DFT calculations

The DFT calculations were conducted using the Vienna ab initio simulation package (VASP 5.4.4). Projected-augmented-wave potentials were employed to elucidate the interaction between the internal core and external valence electrons. A cutoff energy of 500 eV and the GGA-PBE exchange-correlation energy function were utilized. Plane wave basis sets were employed to expand the Kohn-Sham orbitals. A 2 × 1 × 1 k-point grid was employed. The optimization was terminated when the energy difference between consecutive steps fell below 1 × 10^−5^ eV, and the forces were simultaneously less than 0.03 eV/Å. According to the intergrown structure, the SnO_2_ and TiO_2_ surfaces were integrated with the PtO_2_ surface to construct the PtO_2_-SnO_2_ (Pt_16_Sn_48_O_128_) and PtO_2_–TiO_2_ (Pt_16_Ti_48_O_128_) intergrown models. The adsorption configurations and active sites were determined through iterative optimization of the methane oxidation steps using DFT calculations.

In addition, a Bader charge analysis, based on a rapid algorithm, was employed to quantitatively determine the charge variations of different atoms. The charge differences were visualized using the Visualization for Electronic and Structural Analysis (VESTA) software, following the equation: Δρ (r) = ρ_total_ (r) – ρ_part1_ (r) - ρ_part2_ (r), where ρ_total_ (r) represents the total electronic density of the system, ρ_part1_ (r) signifies the electronic density of part 1, and ρ_part2_ (r) corresponds to the electronic density of part 2.

## Supplementary information

Supplementary Information

Transparent Peer Review file

## Source data

Source Data

## Data Availability

Additional data supporting the findings of this study are provided in the Supplementary Information. Data are available from the corresponding authors upon request. Source data are provided with this paper.
