# Supplementary material for: Epitaxial-strain engineering of supported tensile noble metallene oxides for enhanced oxidation catalysis
Source: Nat Commun. 2026 Oct 13;17:11579. doi: 10.1038/s41467-026-78119-2 (PMC13752311; doi:10.1038/s41467-026-78119-2)
Supplement: Supplementary file 1 [file 41467_2026_78119_MOESM1_ESM.pdf]

Supplementary Information for

Epitaxial-strain engineering of supported tensile noble metallene oxides for enhanced oxidation catalysis

Shoujie Zhang[1†], Dong Lin[2†], Anwen Yu[1†], Haifeng Qi[2], Zhongyu Jiang[2], Xiuhui Zheng[3], Xiang Feng[3]*, Yongning Yang[1], Zhikang Fu[1], Sheng Dai[4]*, Lina Li[5], Aiyong Wang[1], Yanglong Guo[1], Wangcheng Zhan[1], Li Wang[1], Yun Guo[1]*, Xuan Tang[1, 4]*

[1] *State Key Laboratory of Green Chemical Engineering and Industrial Catalysis, Research Institute of Industrial Catalysis, School of Chemistry and Molecular Engineering, East China University of Science and Technology, Shanghai 200237, P. R. China*

[2] *Max Planck-Cardiff Centre on the Fundamentals of Heterogeneous Catalysis FUNCAT, Cardiff Catalysis Institute, School of Chemistry, Cardiff University, Cardiff CF24 4HQ, United Kingdom*

[3] *State Key Laboratory of Heavy Oil Processing, China University of Petroleum, Qingdao 266580, P. R. China*

[4] *Key Laboratory for Advanced Materials and Joint International Research Laboratory of Precision Chemistry and Molecular Engineering, Feringa Nobel Prize Scientist Joint Research Center, School of Chemistry and Molecular Engineering, East China University of Science and Technology, Shanghai 200237, P. R. China*

[5] *Shanghai Synchrotron Radiation Facility, Shanghai Advanced Research Institute, Chinese Academy of Sciences, Shanghai 201210, P. R. China*

[†] These authors contributed equally

* Corresponding E-mail: xiangfeng@upc.edu.cn (X. F.); shengdai@ecust.edu.cn (S. D.); yunguo@ecust.edu.cn (Y. G.); xuantang@ecust.edu.cn (X. T.)

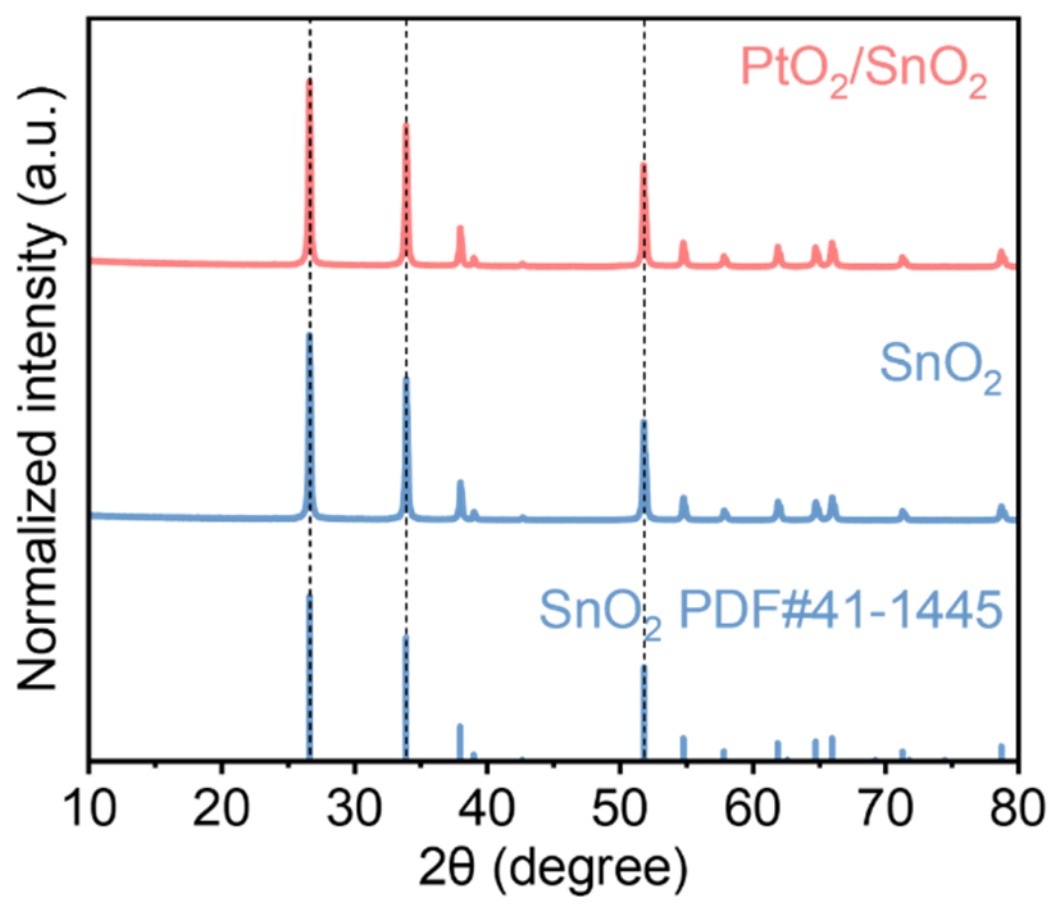

Supplementary Fig. 1 XRD characterization of PtO_2/SnO_2. The XRD patterns of PtO_2/SnO_2 and SnO_2.

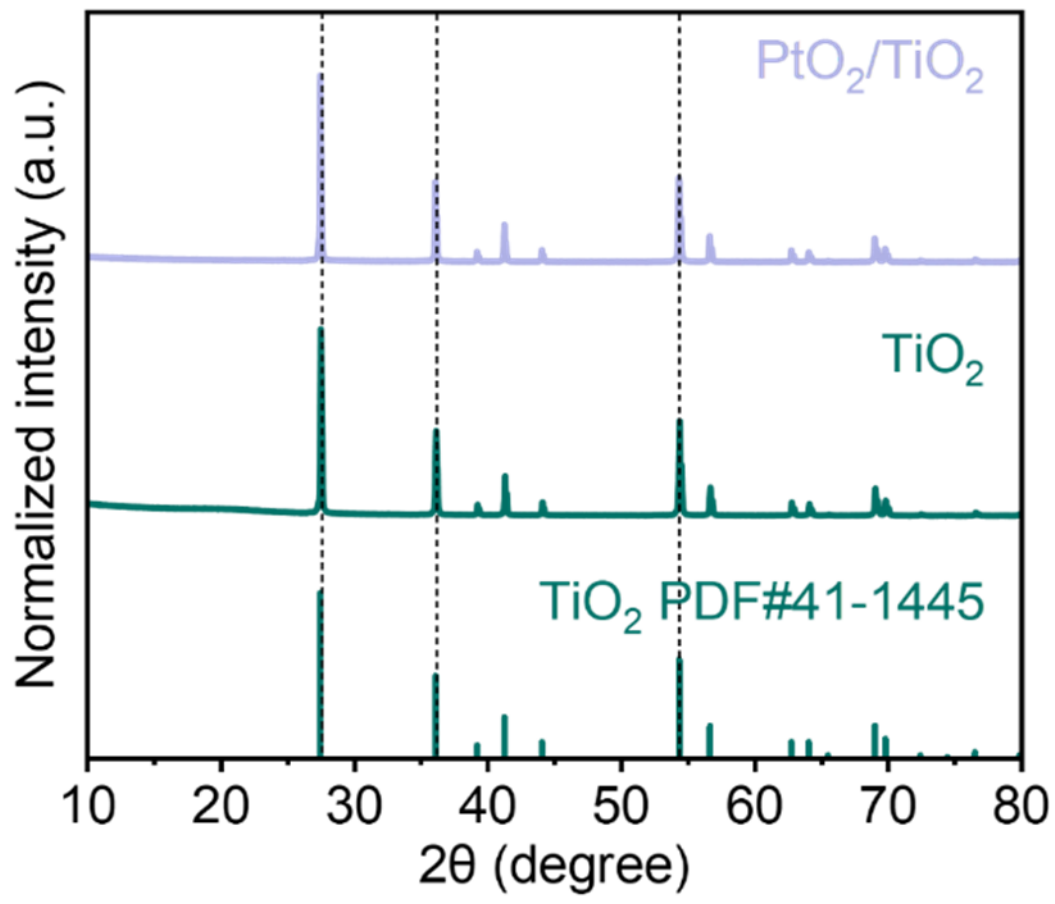

Supplementary Fig. 2 XRD characterization of PtO_2/TiO_2. The XRD patterns of PtO_2/TiO_2 and TiO_2.

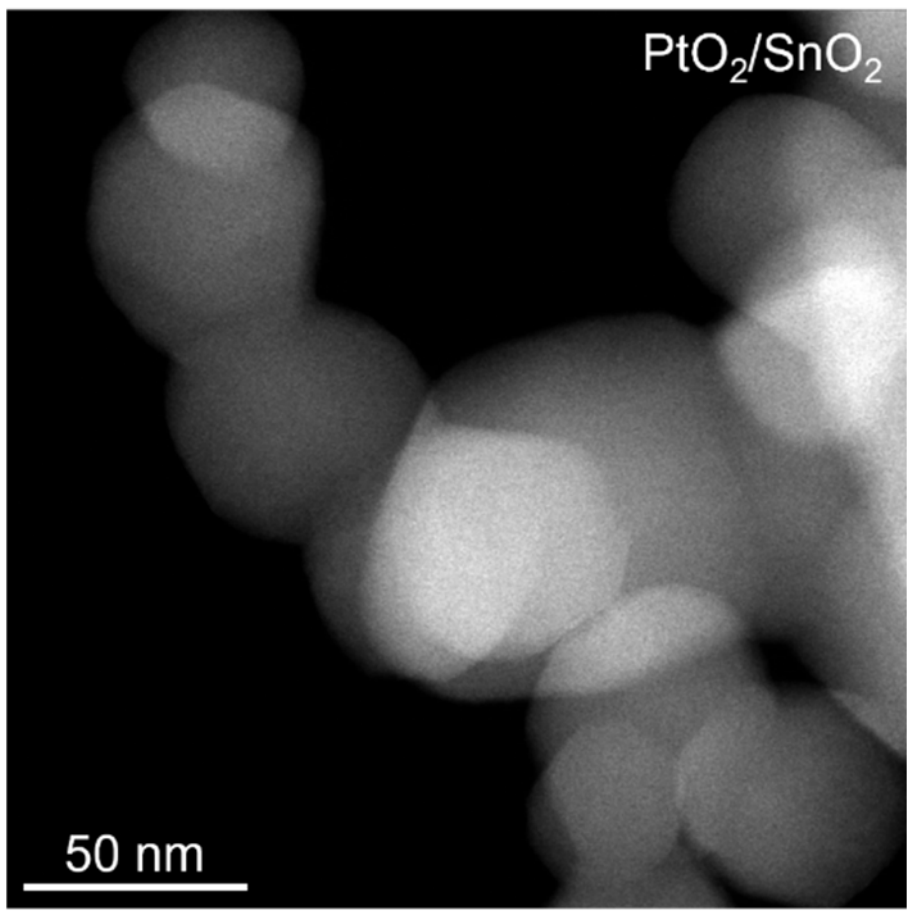

Supplementary Fig. 3 HAADF-STEM image of PtO_2/SnO_2. The representative low-magnification HAADF-STEM image of the PtO_2/SnO_2 catalyst.

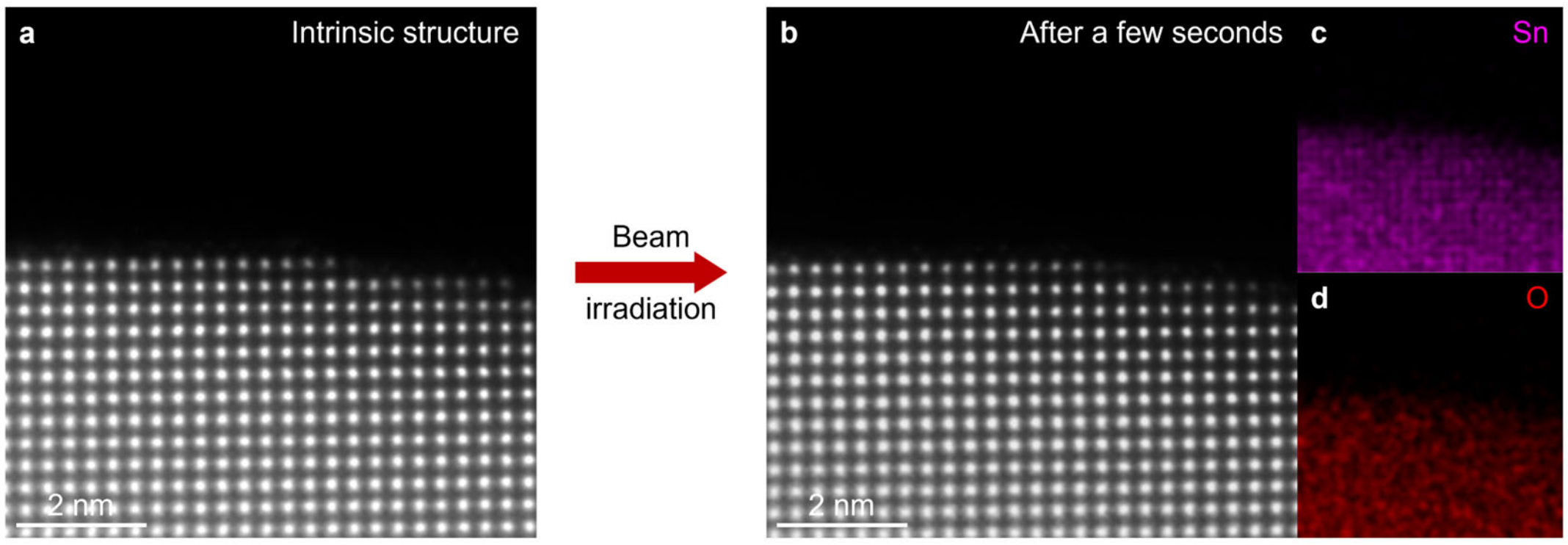

Supplementary Fig. 4 Electron-beam stability of SnO_2. (a) The HAADF-STEM image of pure SnO_2 support was acquired by the low-damage HAADF-STEM imaging method under the beam current of ~ 50 pA. (b) The HAADF-STEM image was acquired by observing the same area in (a) continuously under HAADF-STEM mode at the beam current of ~ 50 pA for a few seconds. (c-d) The corresponding EDS elemental mapping images of (c) Sn and (d) O compositions.

Pure SnO_2 support remains structurally stable upon prolonged electron-beam irradiation, with no obvious surface reconstruction or nanoparticle formation. This demonstrates that the nanoparticle-like species observed after beam exposure in PtO_2/SnO_2 (Supplementary Fig. 5) do not originate from the SnO_2 surface itself, but from the beam-induced restructuring of the supported Pt oxide layer.

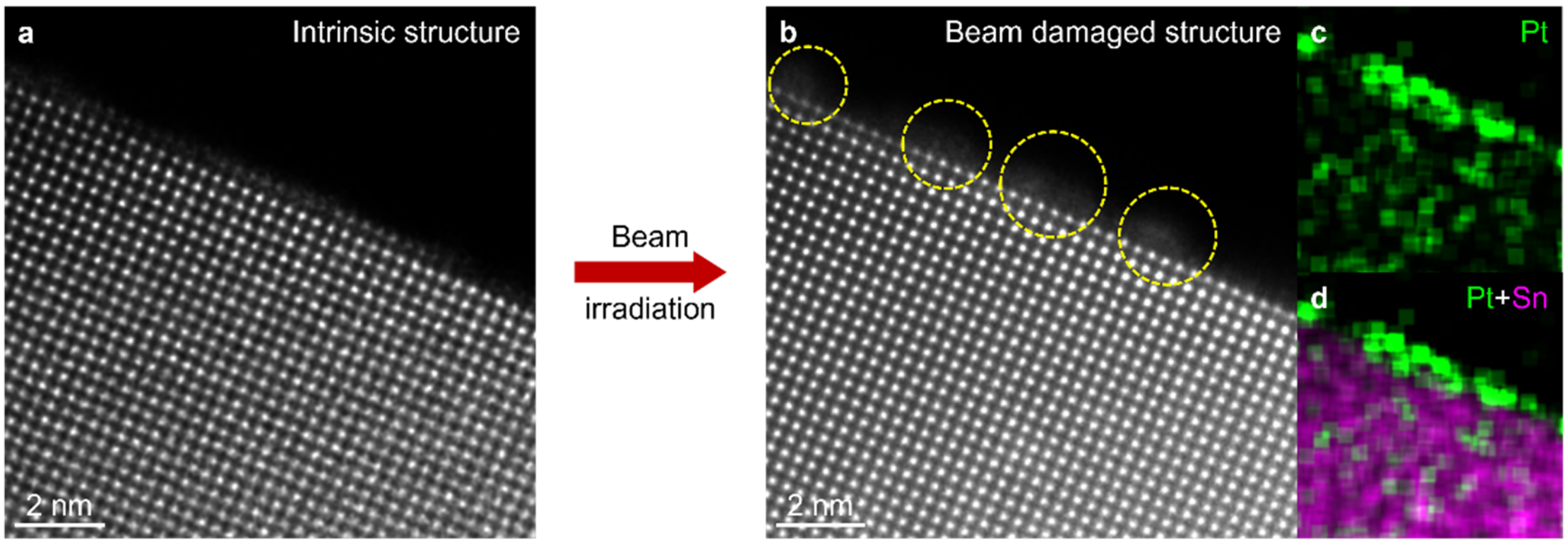

Supplementary Fig. 5 Electron-beam-induced restructuring of PtO_2/SnO_2. (a) The HAADF-STEM image of PtO_2/SnO_2 was acquired by the low-damage HAADF-STEM imaging method under the beam current of ~ 50 pA. (b) The HAADF-STEM image was acquired by observing the same area in (a) continuously under HAADF-STEM mode at the beam current of ~ 50 pA for a few seconds. (c-d) The corresponding EDS elemental mapping images of (c) Pt and (d) Pt+Sn compositions. The yellow circle regions in HAADF-STEM image identify the beam-damaged structure of Pt (small Pt nanoparticles). The HAADF-STEM image in (a) corresponds to the same region shown in Fig. 1c, before rotation for display.

Supplementary Note 1: The stronger Pt signal near the particle edge in the EDS map mainly arises from projection geometry after beam-induced restructuring. During EDS acquisition, the beam-sensitive PtO_2 overlayer can restructure into Pt-containing nanoparticle-like species. For the laterally exposed side surface or particle edge, the EDS signal includes projected contributions from Pt-containing species generated along a relatively long side-surface projection length. In contrast, on the projected top surface, only an atomically thin PtO_2 overlayer contributes along the beam direction, resulting in a weaker Pt signal. Therefore, the stronger Pt signal near the particle edge does not indicate that Pt is detached from the SnO_2 support or located only at the edge, but reflects the different projected Pt amounts from the side and top surfaces in two-dimensional EDS mapping.

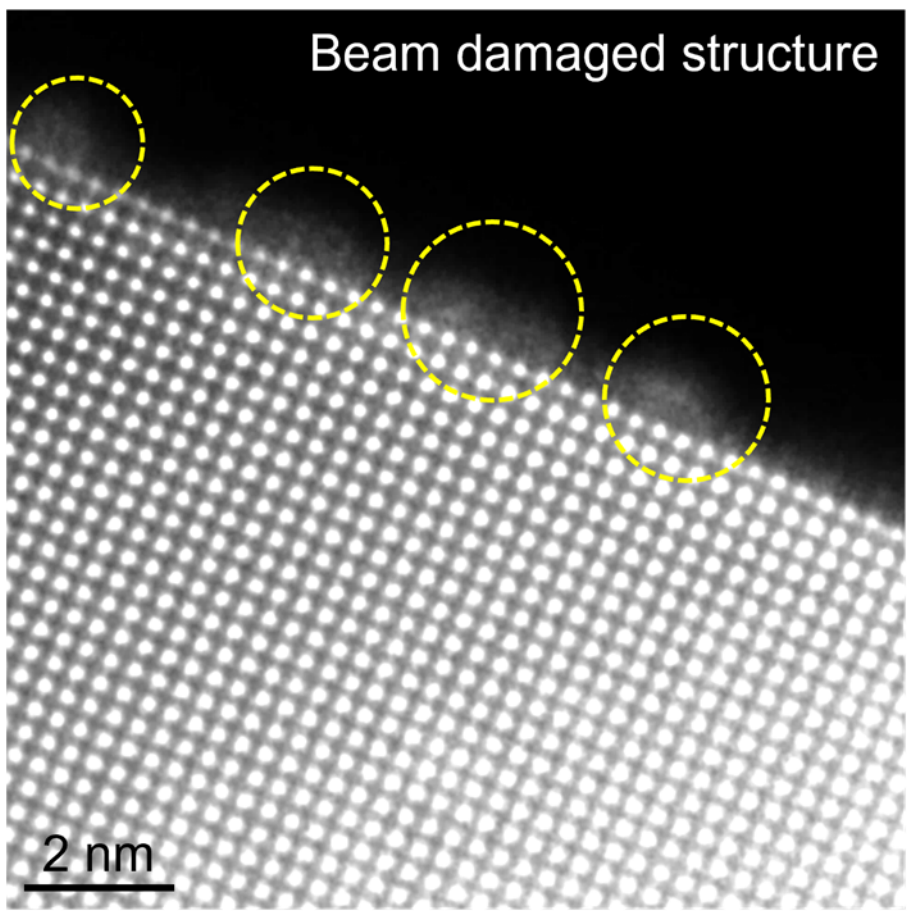

Supplementary Fig. 6 Beam-damaged PtO_2/SnO_2. The contrast-adjusted HAADF-STEM image of PtO_2/SnO_2 after electron beam damage. The image is derived from Supplementary Fig. 5b with only contrast adjusted to improve the visibility of beam-induced Pt-containing nanoparticle-like species, which are marked by yellow circles.

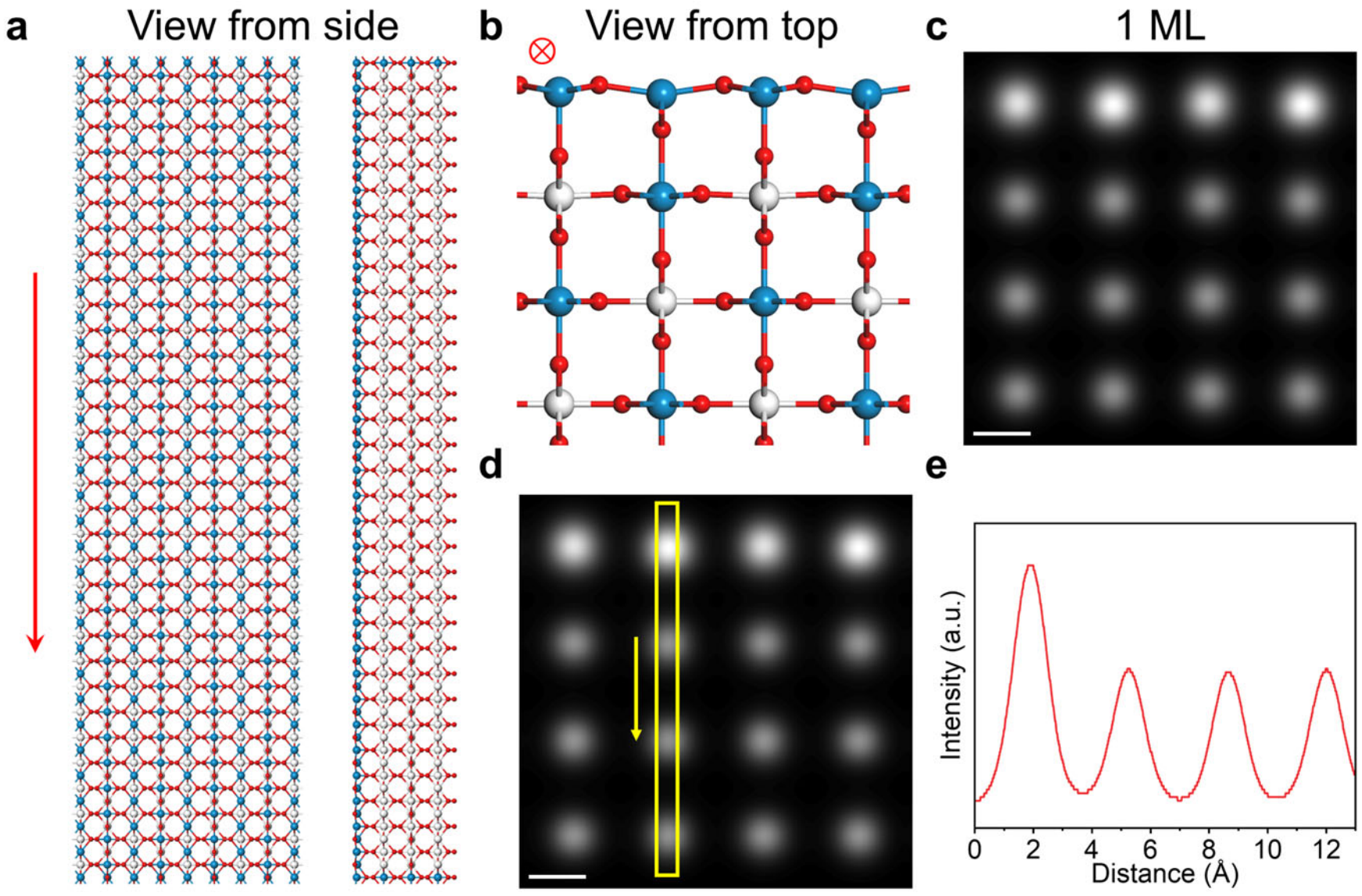

Supplementary Fig. 7 Simulated HAADF-STEM images of 1 ML PtO_2/SnO_2. (a, b) Atomic structural models, (c, d) simulated HAADF-STEM images, and (e) corresponding intensity profile for 1 ML of PtO_2 on the $SnO_2(110)$ surface. The blue, white, and red balls denote Pt, Sn, and O atoms, respectively. The intensity profile was taken from the rectangular box along the yellow arrow in (d). Scale bars: 2 Å in (c) and (d).

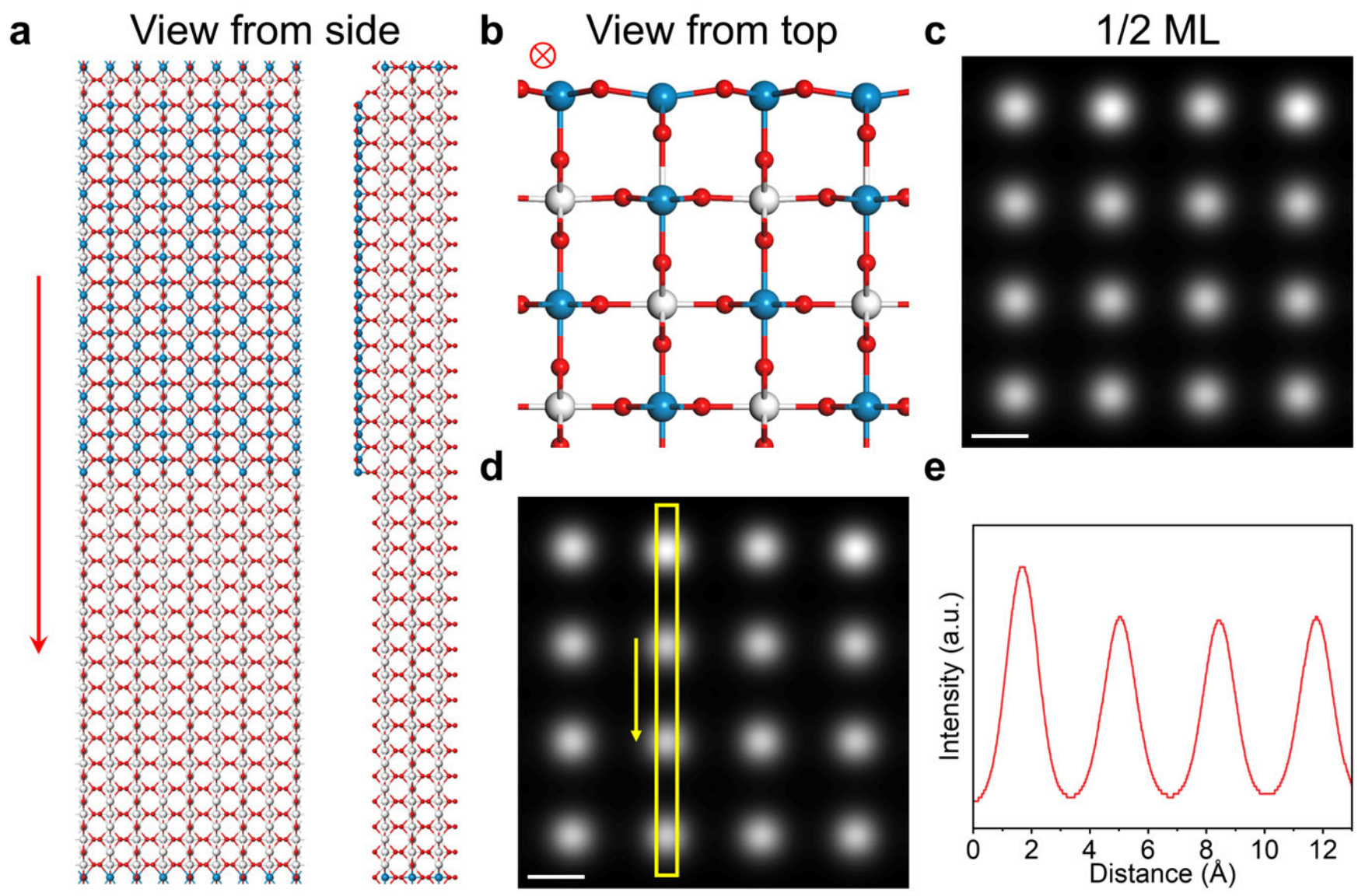

Supplementary Fig. 8 Simulated HAADF-STEM images of 1/2 ML PtO_2/SnO_2. (a, b) Atomic structural models, (c, d) simulated HAADF-STEM images, and (e) corresponding intensity profile for 1/2 ML of PtO_2 on the SnO_2(110) surface. The blue, white, and red balls denote Pt, Sn, and O atoms, respectively. The intensity profile was taken from the rectangular box along the yellow arrow in (d). Scale bars: 2 Å in (c) and (d).

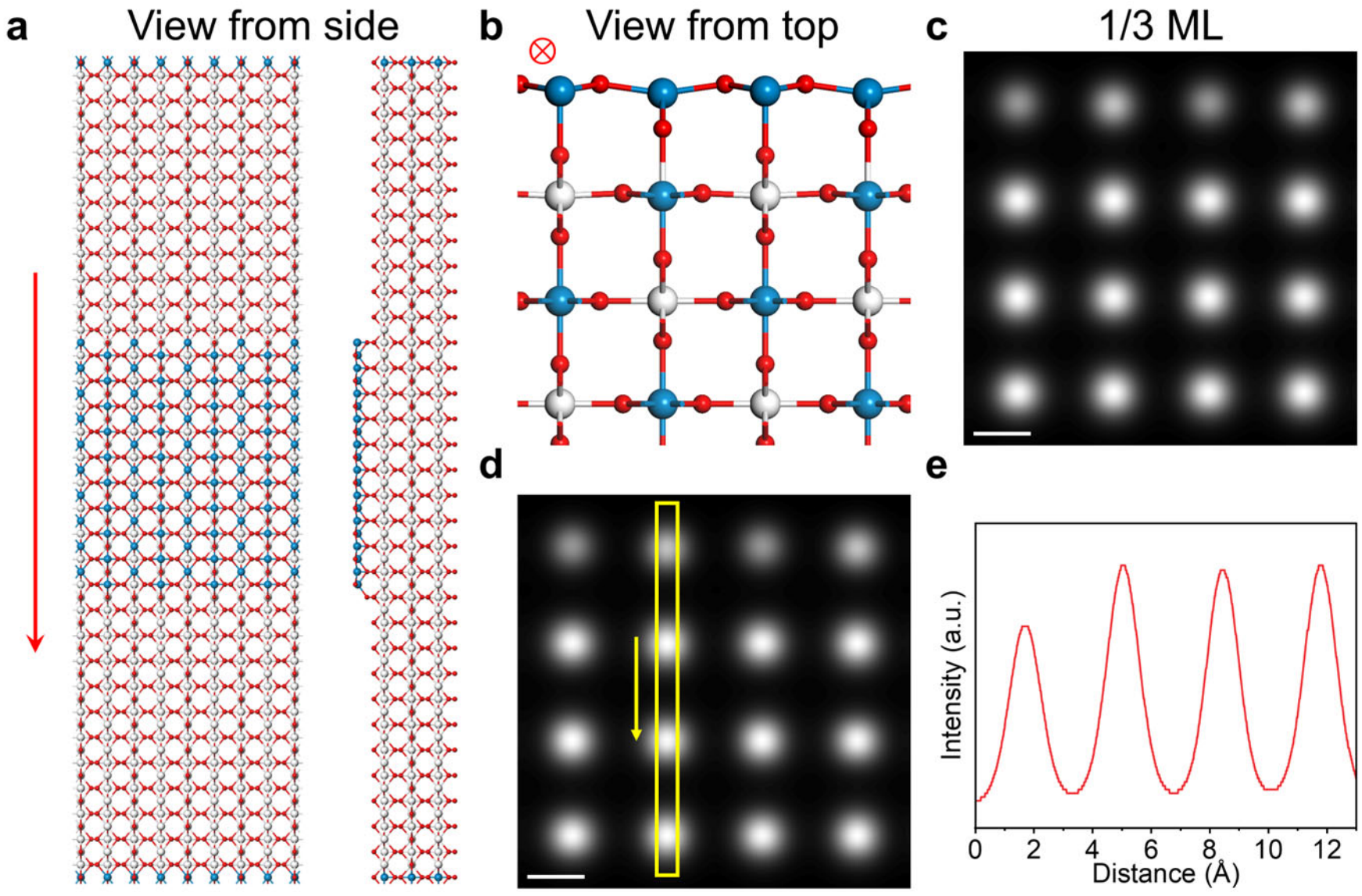

Supplementary Fig. 9 Simulated HAADF-STEM images of 1/3 ML PtO_2/SnO_2. (a, b) Atomic structural models, (c, d) simulated HAADF-STEM images, and (e) corresponding intensity profile for 1/3 ML of PtO_2 on the $SnO_2(110)$ surface. The blue, white, and red balls denote Pt, Sn, and O atoms, respectively. The intensity profile was taken from the rectangular box along the yellow arrow in (d). Scale bars: 2 Å in (c) and (d).

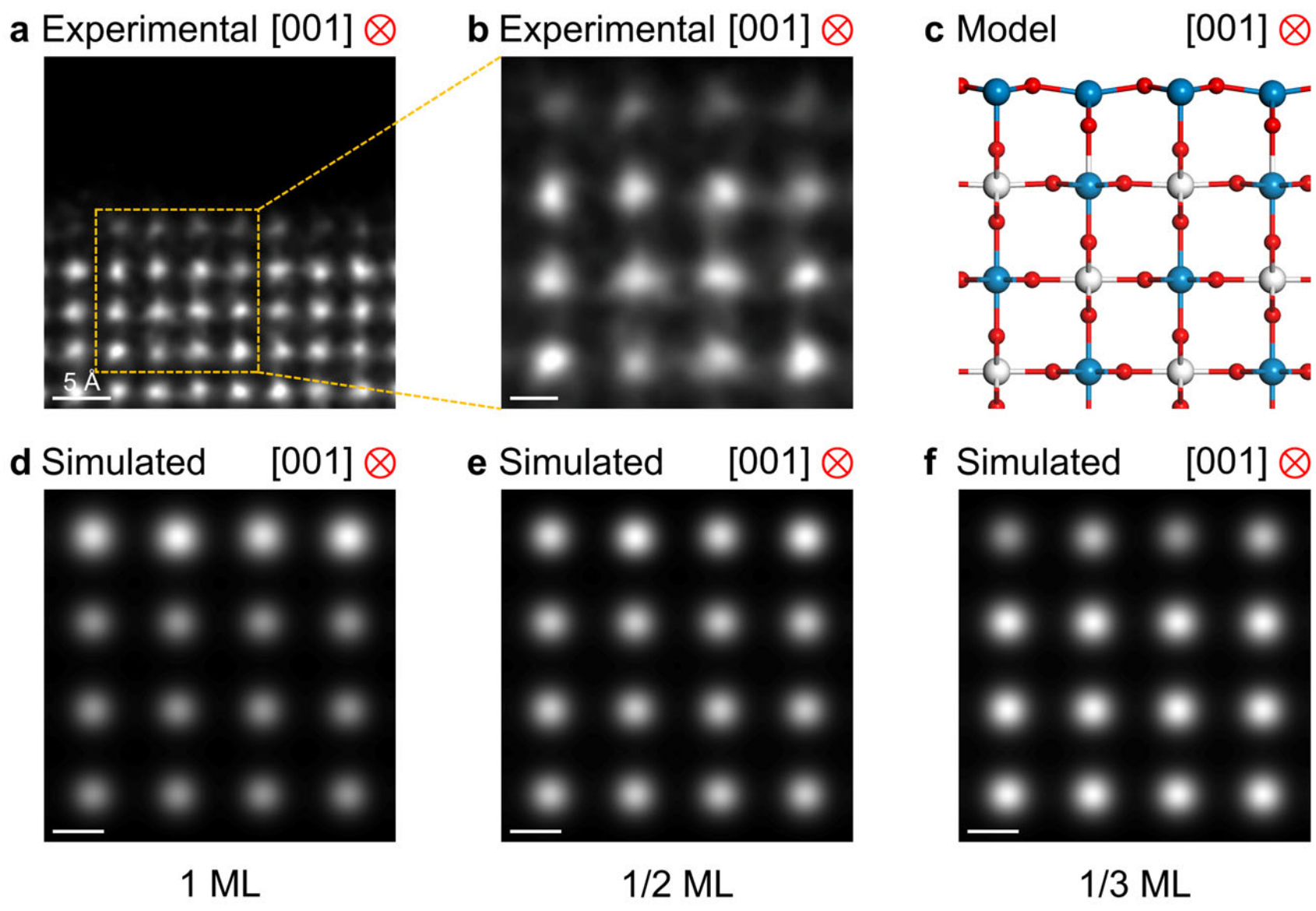

Supplementary Fig. 10 Experimental and simulated HAADF-STEM images. (a) HAADF-STEM image taken from Fig. 1d. (b) Magnified HAADF-STEM image of the golden boxed region in (a). (c) Atomic structural model of PtO_2 on the $SnO_2(110)$ surface. (d-f) Simulated HAADF-STEM images for PtO_2 coverages of 1 ML, 1/2 ML, and 1/3 ML on the $SnO_2(110)$ surface, respectively. Scale bars, 2 Å in (b) and (d-f).

Supplementary Note 2: HAADF-STEM image simulations were performed using a $SnO_2(110)$ slab model with a thickness of approximately 10 nm, in which PtO_2 overlayers with different edge-exposed coverages, including one monolayer (1 ML), one-half monolayer (1/2 ML), and one-third monolayer (1/3 ML), were constructed (**Supplementary Figs. 7-10**). The simulation results show that Pt columns are brighter than Sn columns at full monolayer coverage, as expected from Z-contrast (**Supplementary Fig. 7**). However, the relative Pt-column intensity decreases markedly as the PtO_2 coverage decreases (**Supplementary Fig. 8**). At approximately 1/3 ML coverage, the simulated Pt-containing columns become less intense than the SnO_2 support columns (**Supplementary Fig. 9**). The line-intensity analysis also shows that the apparent contrast is strongly affected by projection through the SnO_2 slab. These simulations therefore explain why the surface Pt-containing layer in the low-

loading (0.73 wt%) PtO_2/SnO_2 catalyst does not appear brighter than the SnO_2 support in the experimental HAADF-STEM images (**Supplementary Figs. 10a-b**).

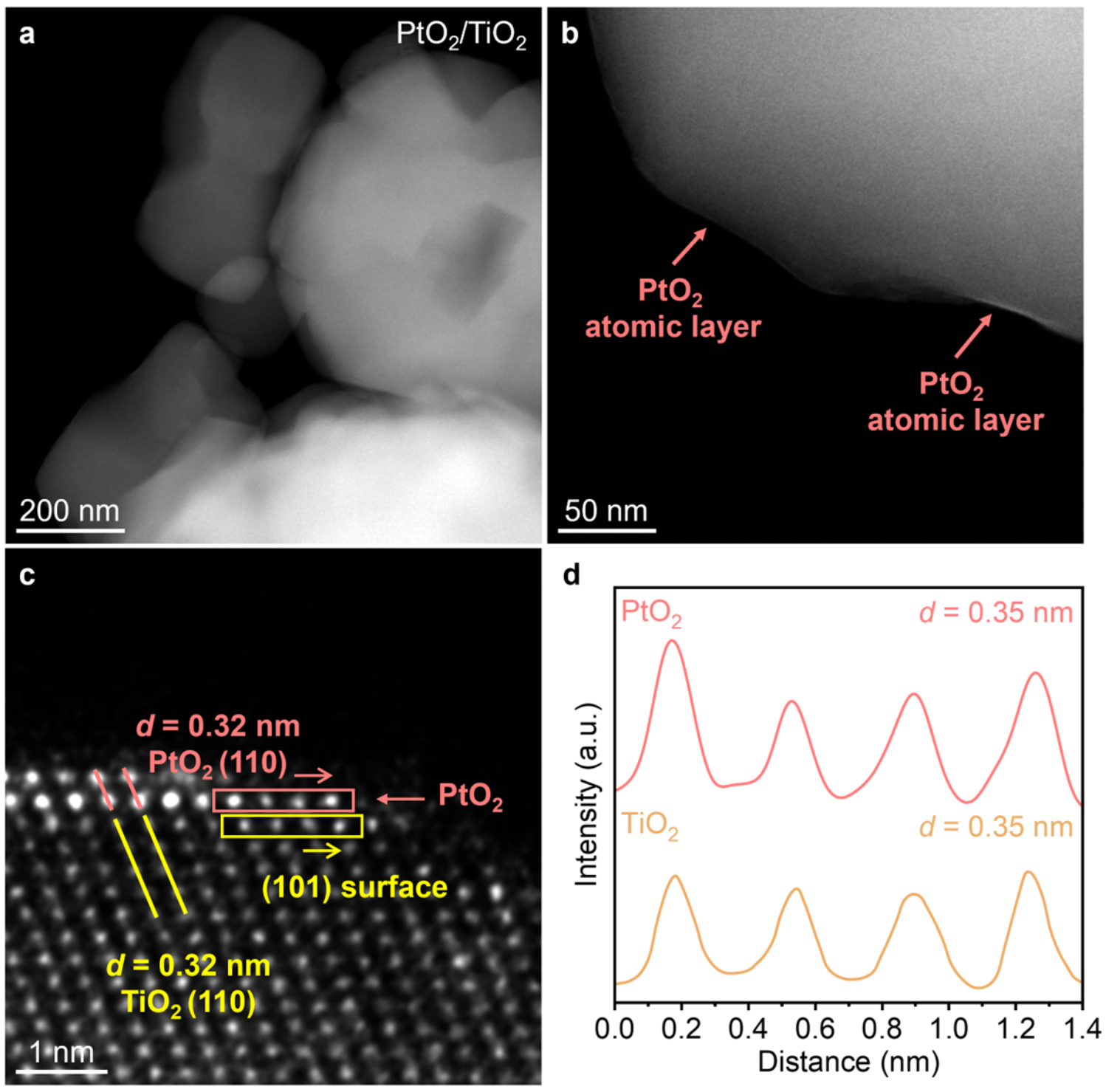

Supplementary Fig. 11 HAADF-STEM characterization of PtO_2/TiO_2. Representative (a, b) low-magnification and (c) atomic-scale HAADF-STEM images of PtO_2/TiO_2 catalyst. (d) Intensity profiles of PtO_2 and TiO_2 taken from the selected red and yellow areas in (c).

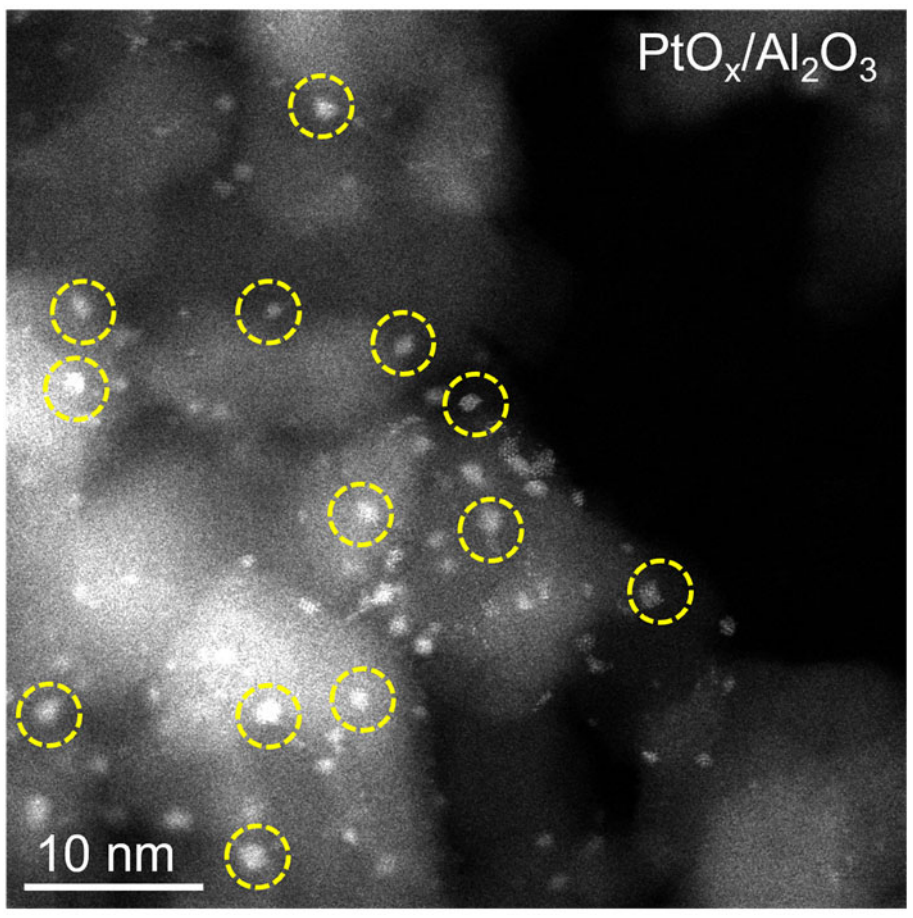

Supplementary Fig. 12 HAADF-STEM image of PtO_x/Al_2O_3. Representative HAADF-STEM image of PtO_x/Al_2O_3. The yellow circle regions in HAADF-STEM image identify the Pt clusters.

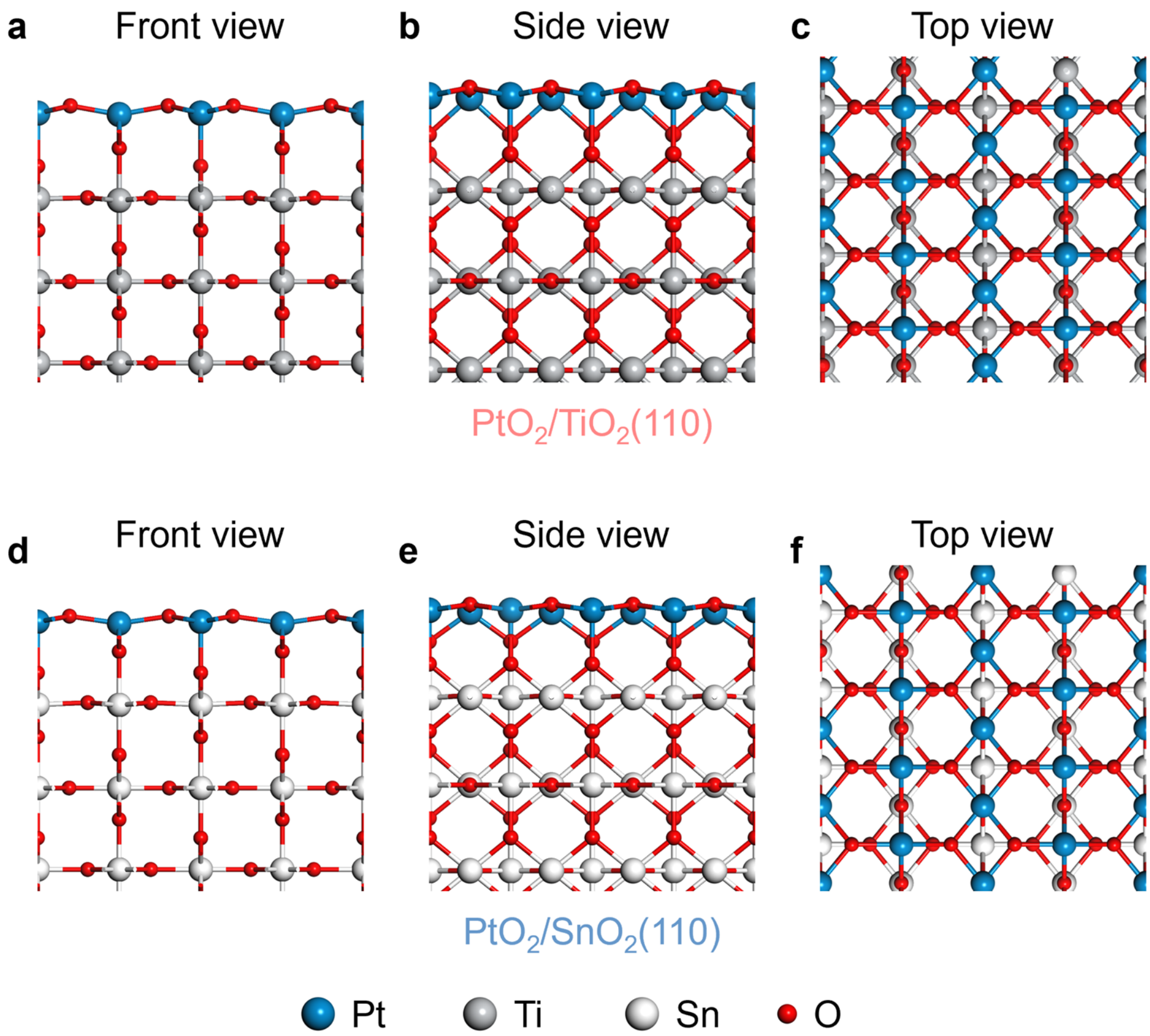

Supplementary Fig. 13 DFT models of $PtO_2/TiO_2(110)$ and $PtO_2/SnO_2(110)$. (a-c) The DFT-optimized model of $PtO_2/TiO_2(110)$, viewed from the (a) front, (b) side, and (c) top. (d-f) The DFT-optimized model of $PtO_2/SnO_2(110)$, viewed from the (d) front, (e) side, and (f) top.

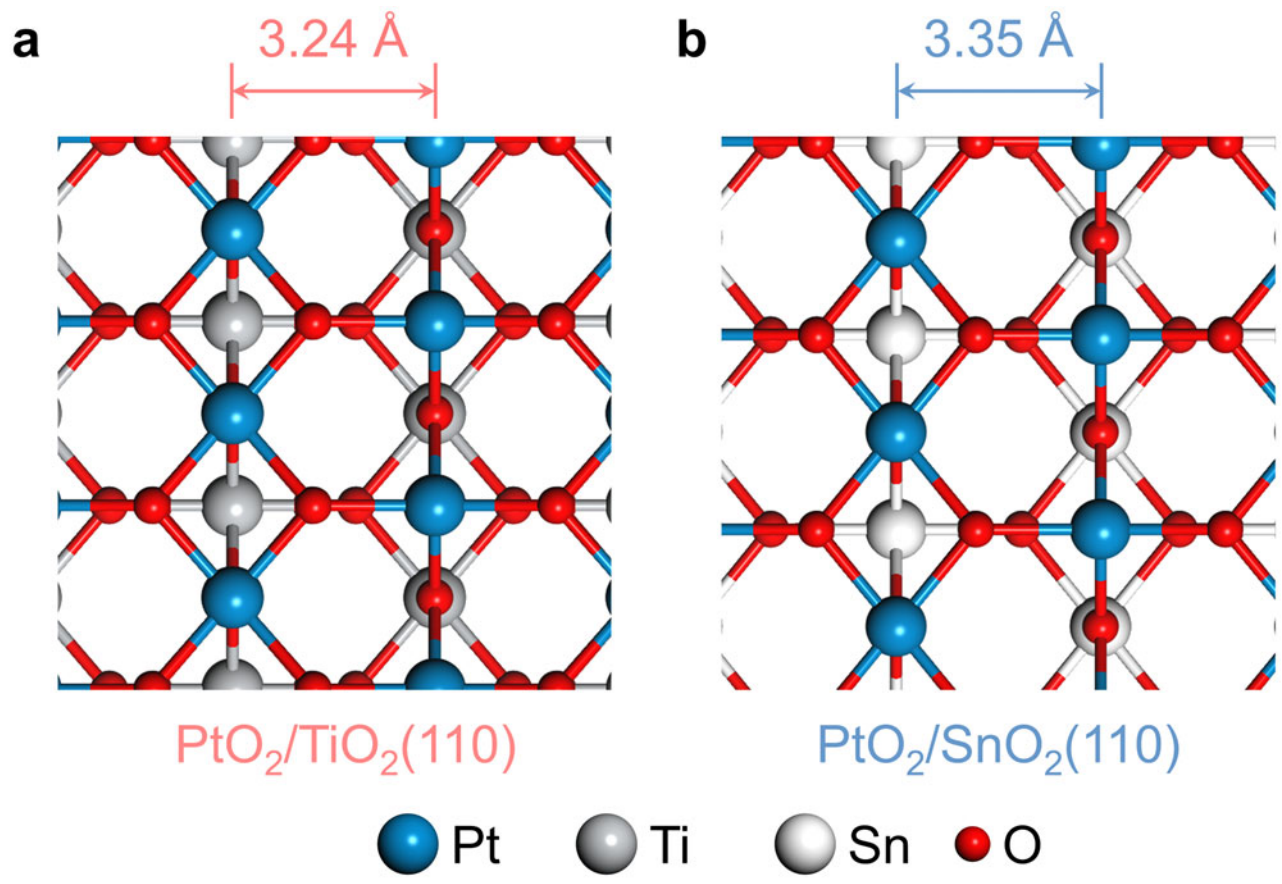

Supplementary Fig. 14 Comparison of lattice spacings in $PtO_2/TiO_2(110)$ and $PtO_2/SnO_2(110)$. Schematic illustration of lattice spacing for the (110) plane of (a) $PtO_2/TiO_2(110)$ and (b) $PtO_2/SnO_2(110)$, viewed from the top.

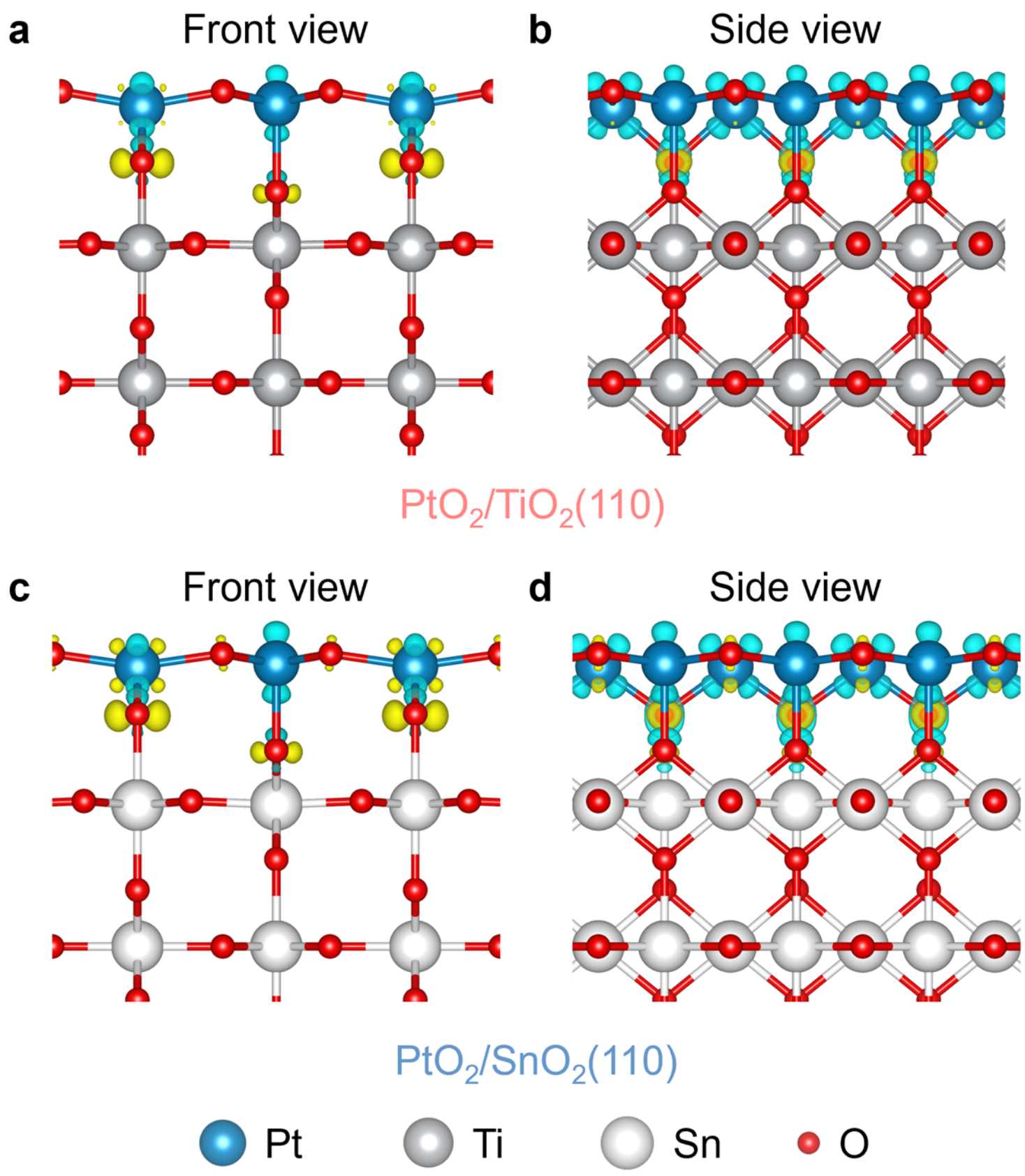

Supplementary Fig. 15 Interfacial charge redistribution in $PtO_2/TiO_2(110)$ and $PtO_2/SnO_2(110)$. The differential charge density plots of (a-b) $PtO_2/TiO_2(110)$ and (c-d) $PtO_2/SnO_2(110)$. The light blue and light yellow iso-surfaces represent the decrease and increase in charge density, respectively.

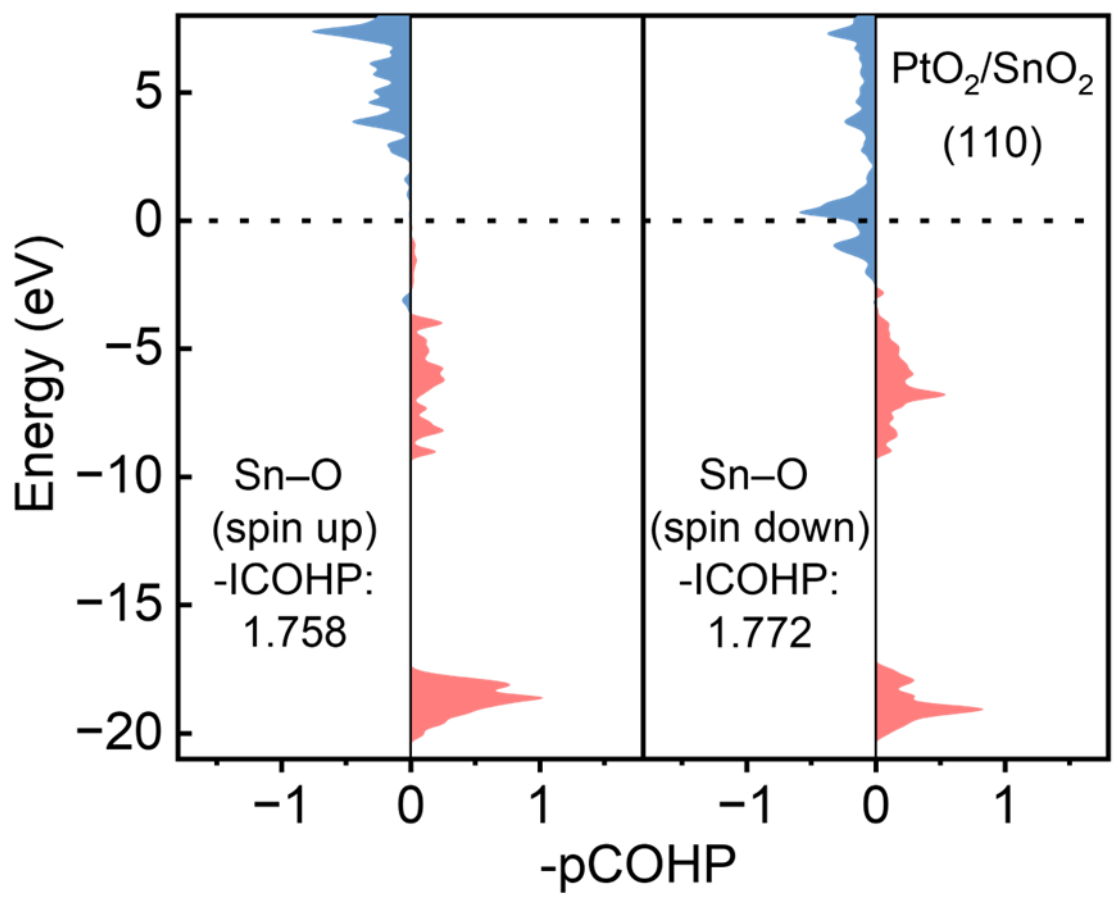

Supplementary Fig. 16 pCOHP analysis of PtO_2/SnO_2. The pCOHPs of Sn–O bonds on the interface of $PtO_2/SnO_2(110)$.

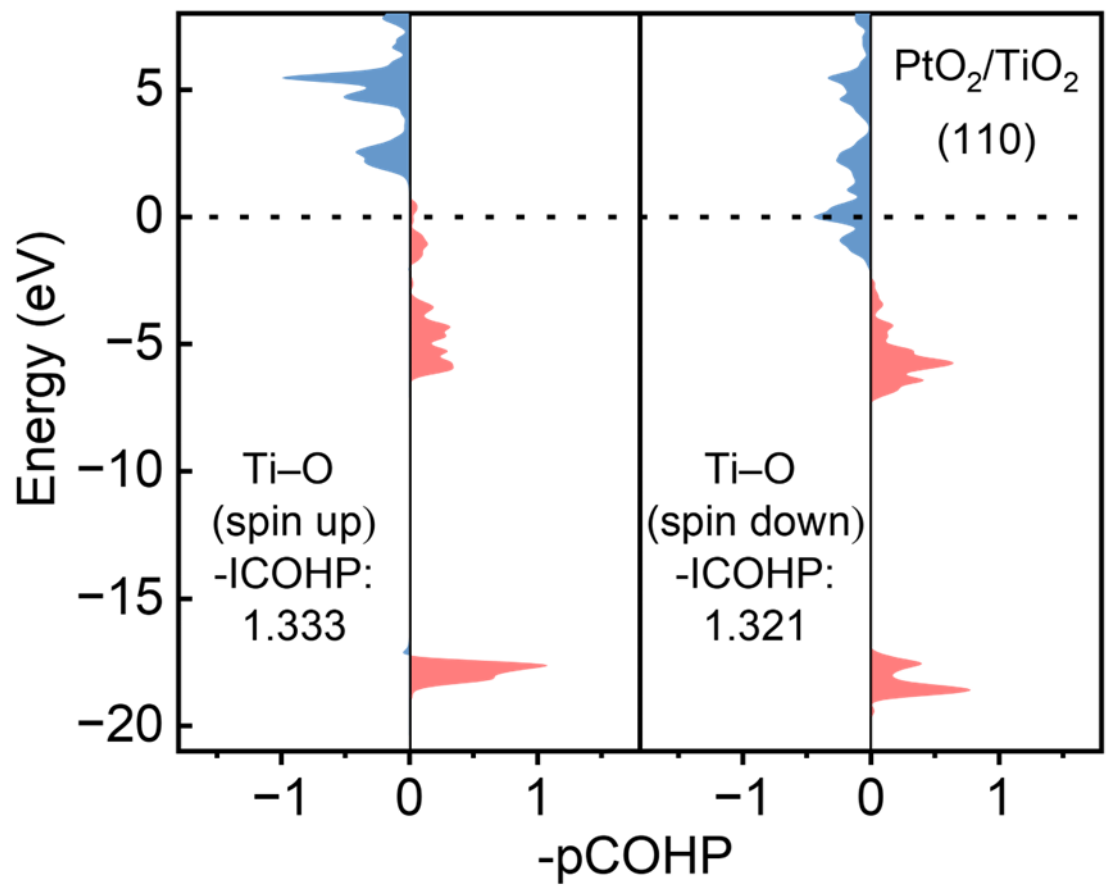

Supplementary Fig. 17 pCOHP analysis of PtO_2/TiO_2. The pCOHPs of Ti–O bonds on the interface of $PtO_2/TiO_2(110)$.

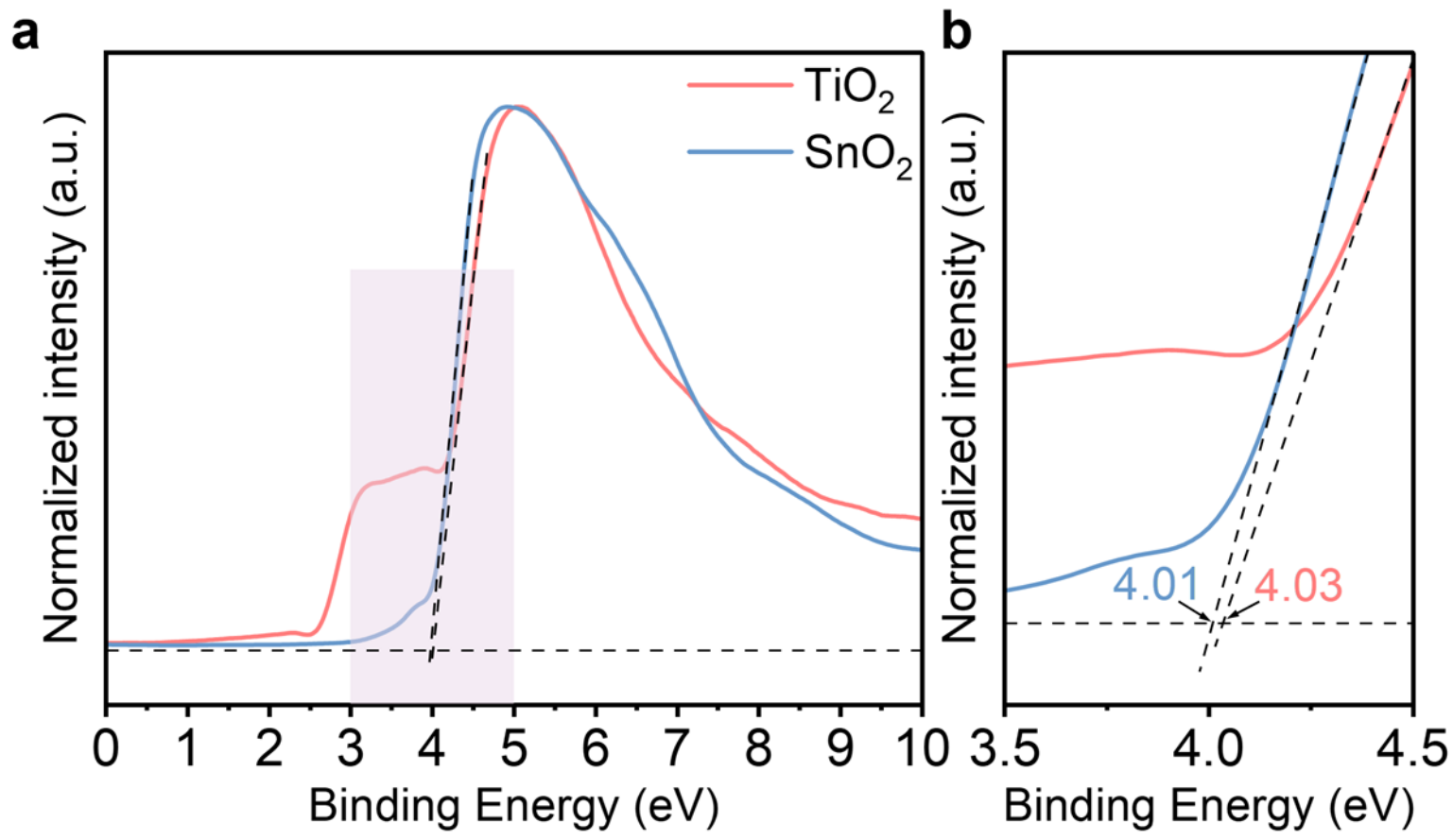

Supplementary Fig. 18 UPS analysis of TiO_2 and SnO_2. (a) UPS spectra and (b) UPS-derived work functions of the TiO_2 and SnO_2 supports.

The UPS-derived work functions of SnO_2 and TiO_2 are 4.01 and 4.03 eV, respectively, with a very small difference of only 0.02 eV. This result suggests that the difference in support-induced interfacial charge transfer arising solely from energy alignment is likely limited. Therefore, although the contribution from interfacial energy alignment cannot be completely excluded, it is unlikely to be the dominant origin of the observed difference in the Pt^{4+} fraction between PtO_2/SnO_2 and PtO_2/TiO_2.

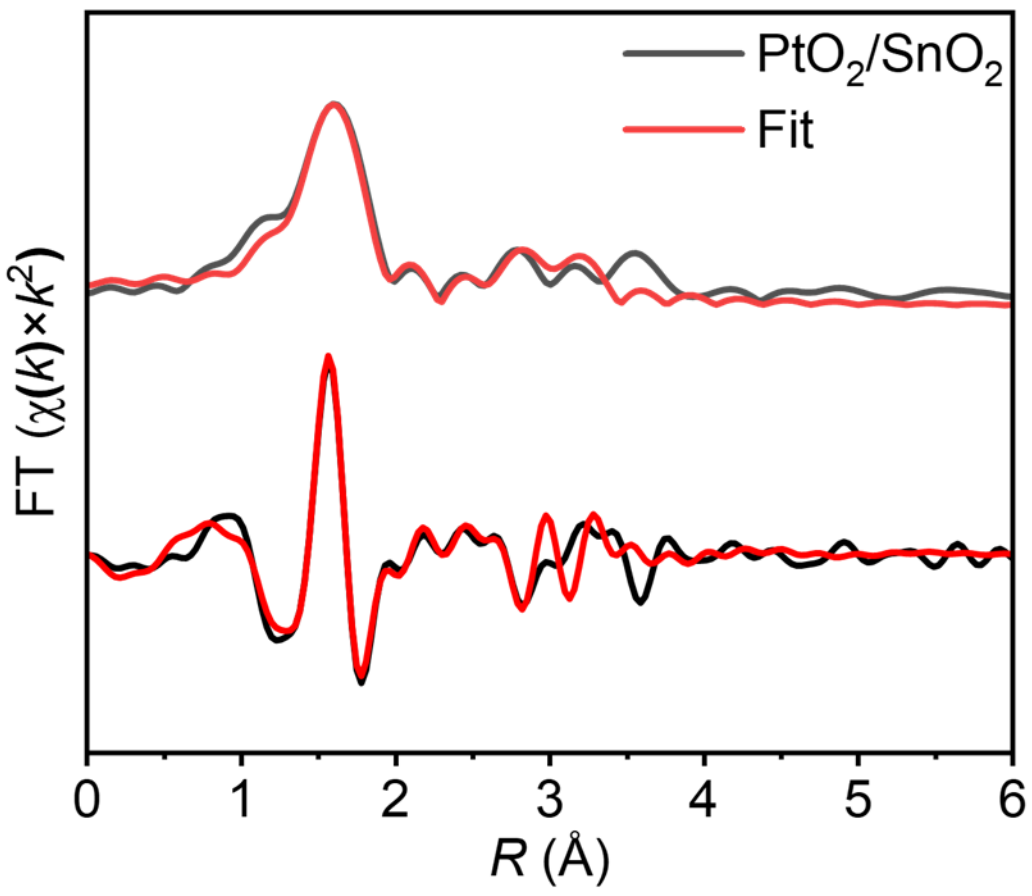

Supplementary Fig. 19 EXAFS fitting of PtO_2/SnO_2. The fit of the FT EXAFS spectra (top) and the FT imaginary portion (bottom) of PtO_2/SnO_2.

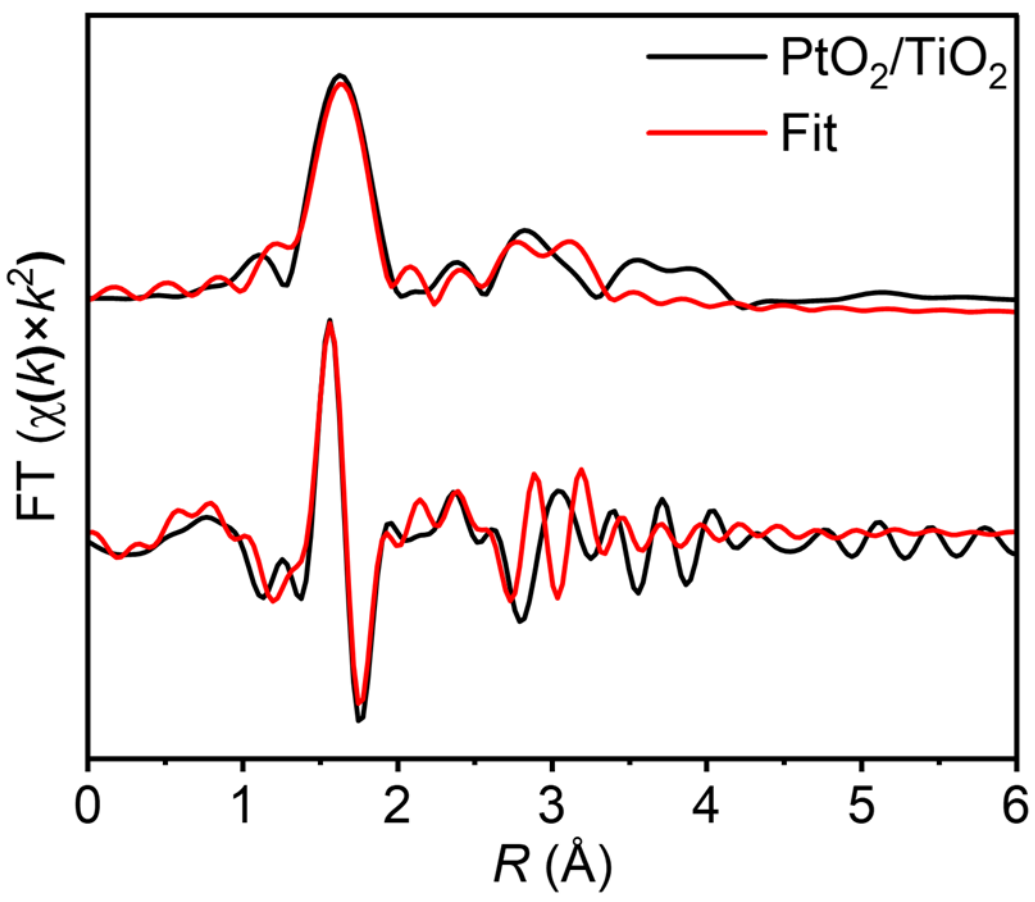

Supplementary Fig. 20 EXAFS fitting of PtO_2/TiO_2. The fit of the FT EXAFS spectra (top) and the FT imaginary portion (bottom) of PtO_2/TiO_2.

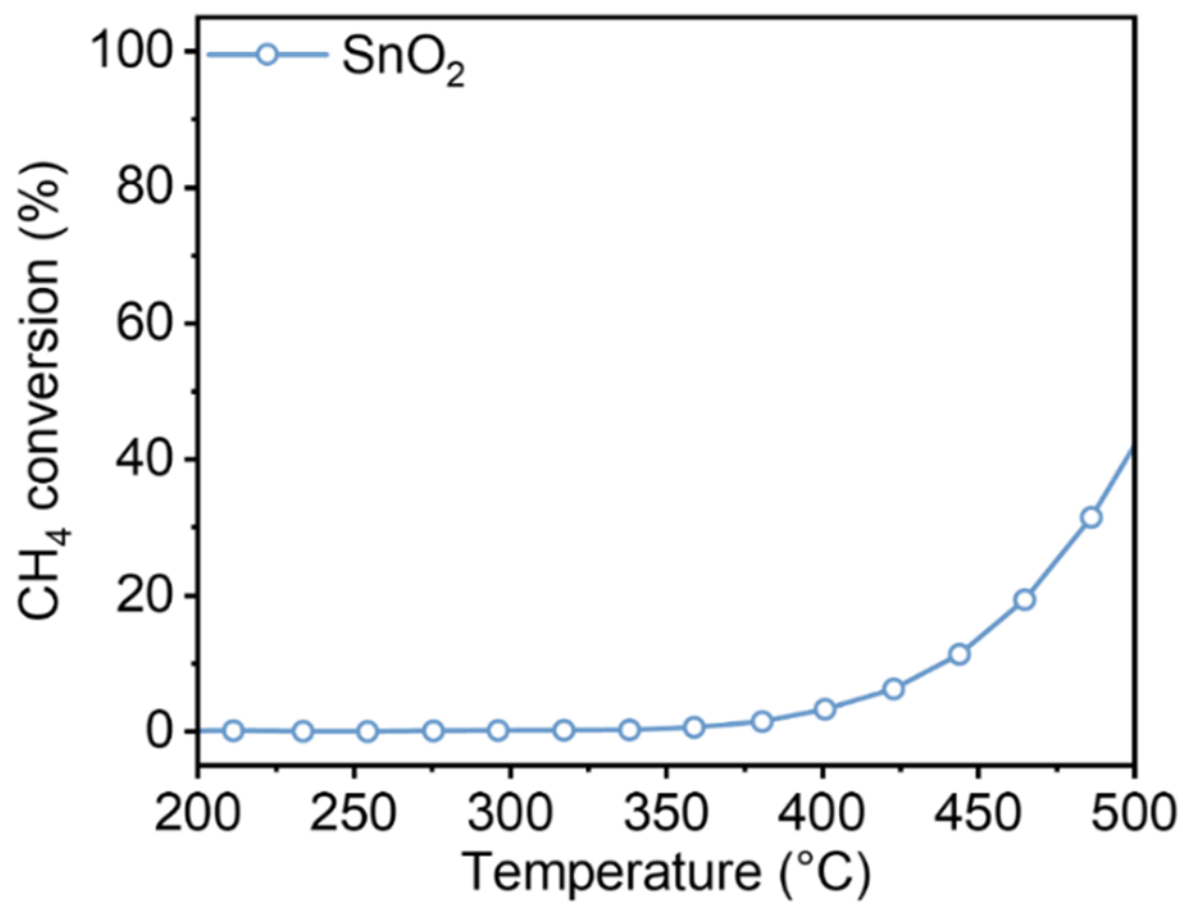

Supplementary Fig. 21 Methane oxidation over SnO_2. The light-off curve of methane oxidation over SnO_2 support.

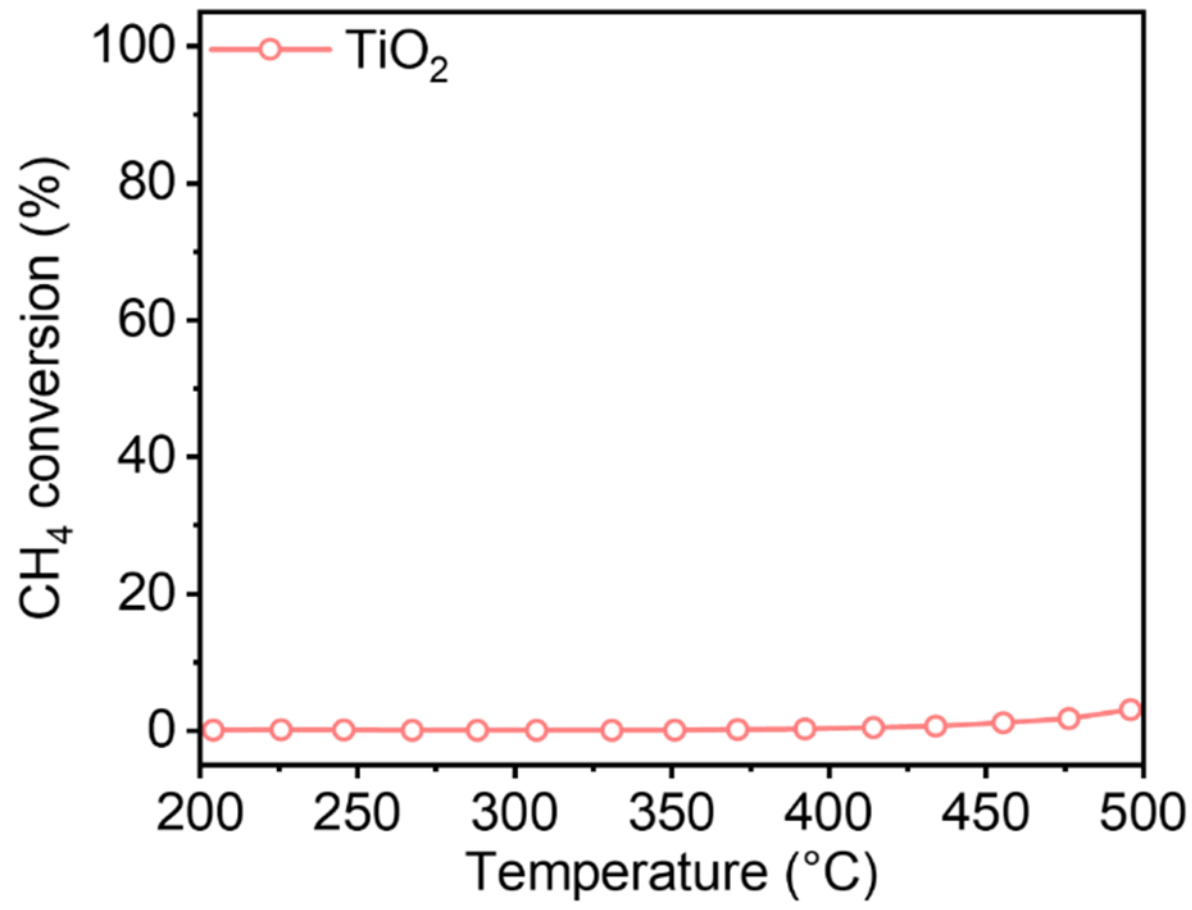

Supplementary Fig. 22 Methane oxidation over TiO_2. The light-off curve of methane oxidation over TiO_2 support.

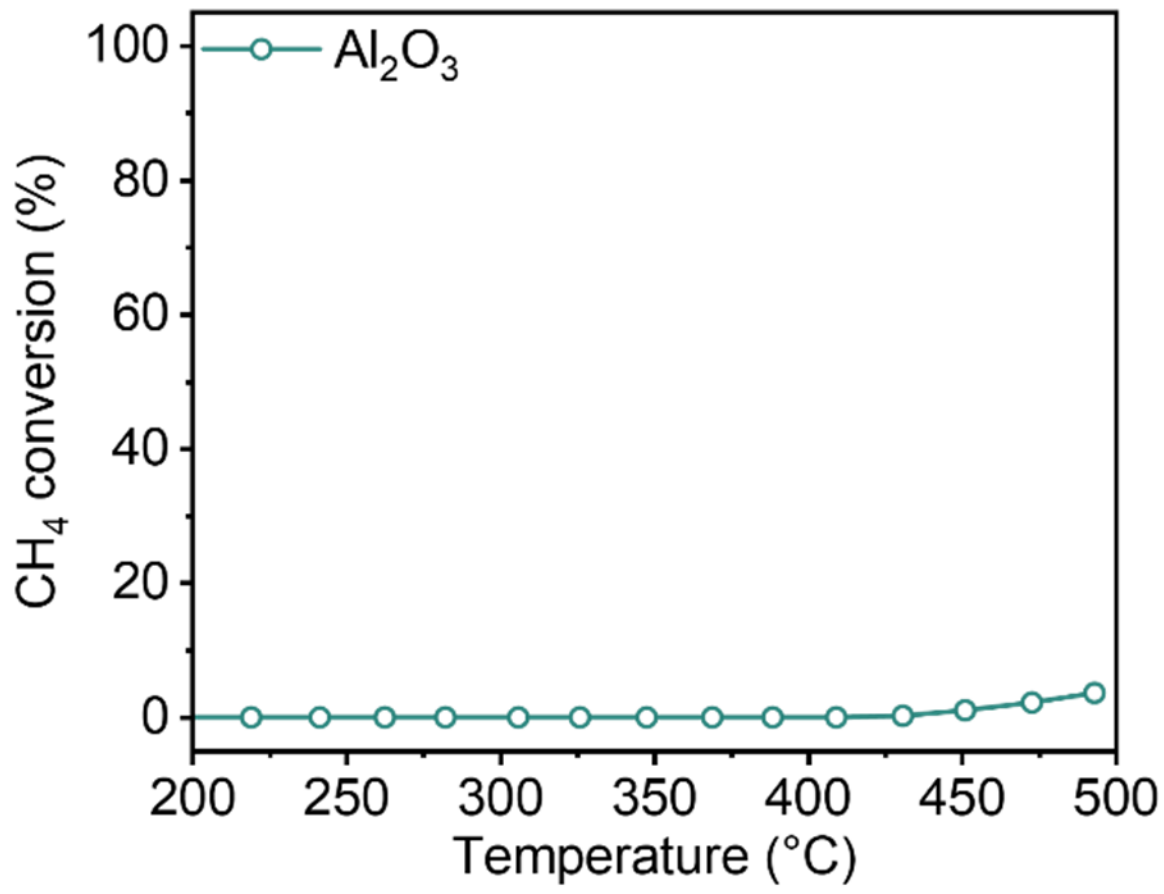

Supplementary Fig. 23 Methane oxidation over Al_2O_3. The light-off curve of methane oxidation over Al_2O_3 support.

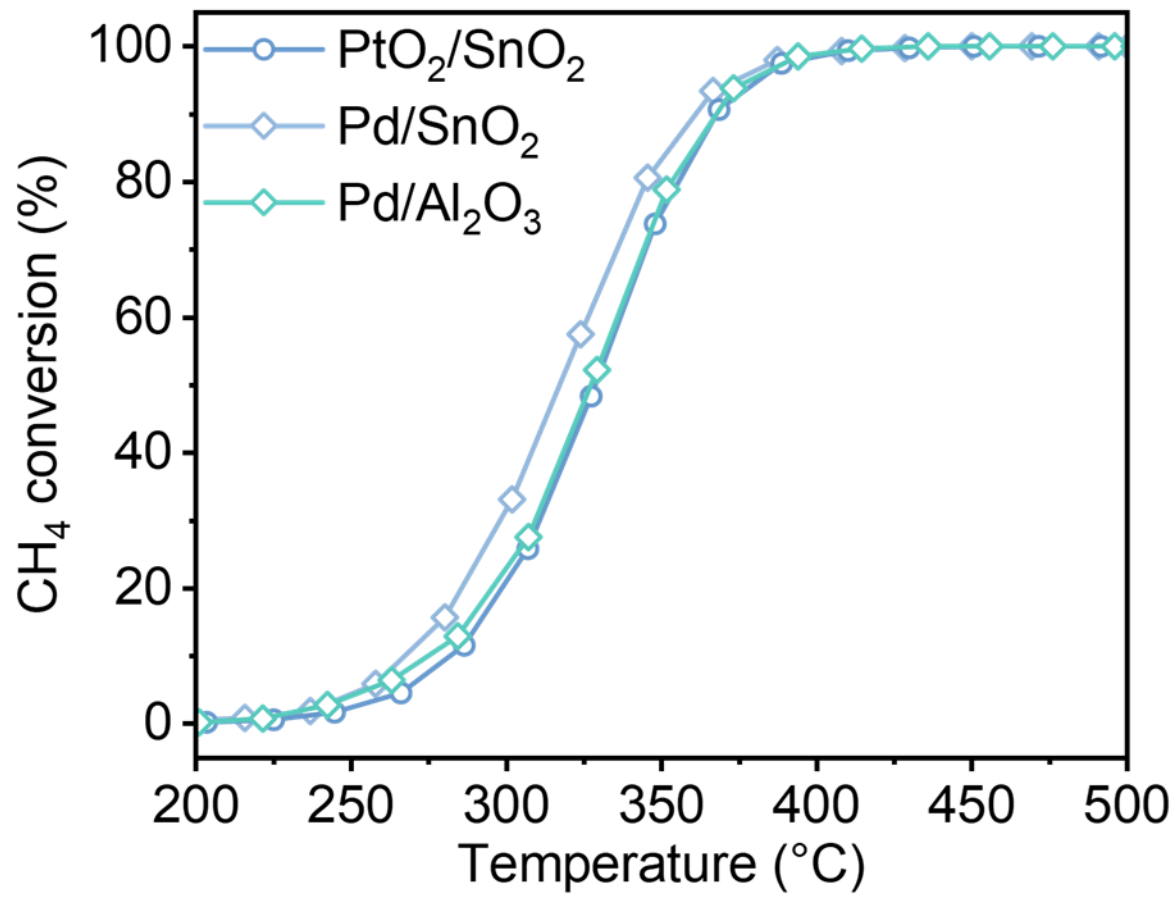

Supplementary Fig. 24 Comparison of methane oxidation activity. The light-off curve of methane oxidation over PtO_2/SnO_2, Pd/SnO_2, and Pd/Al_2O_3.

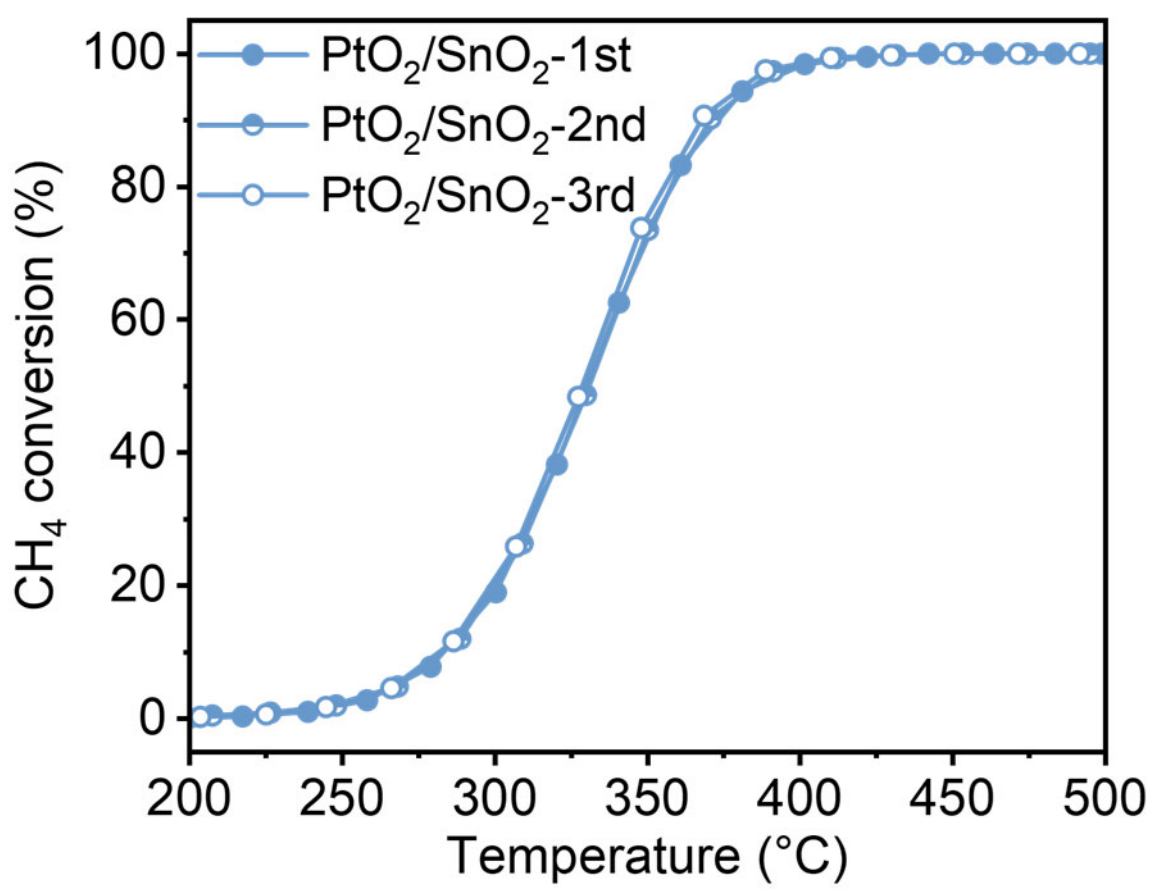

Supplementary Fig. 25 Cycling stability of PtO_2/SnO_2. Repeating ignition cycles of light-off curves of methane oxidation over PtO_2/SnO_2 catalyst.

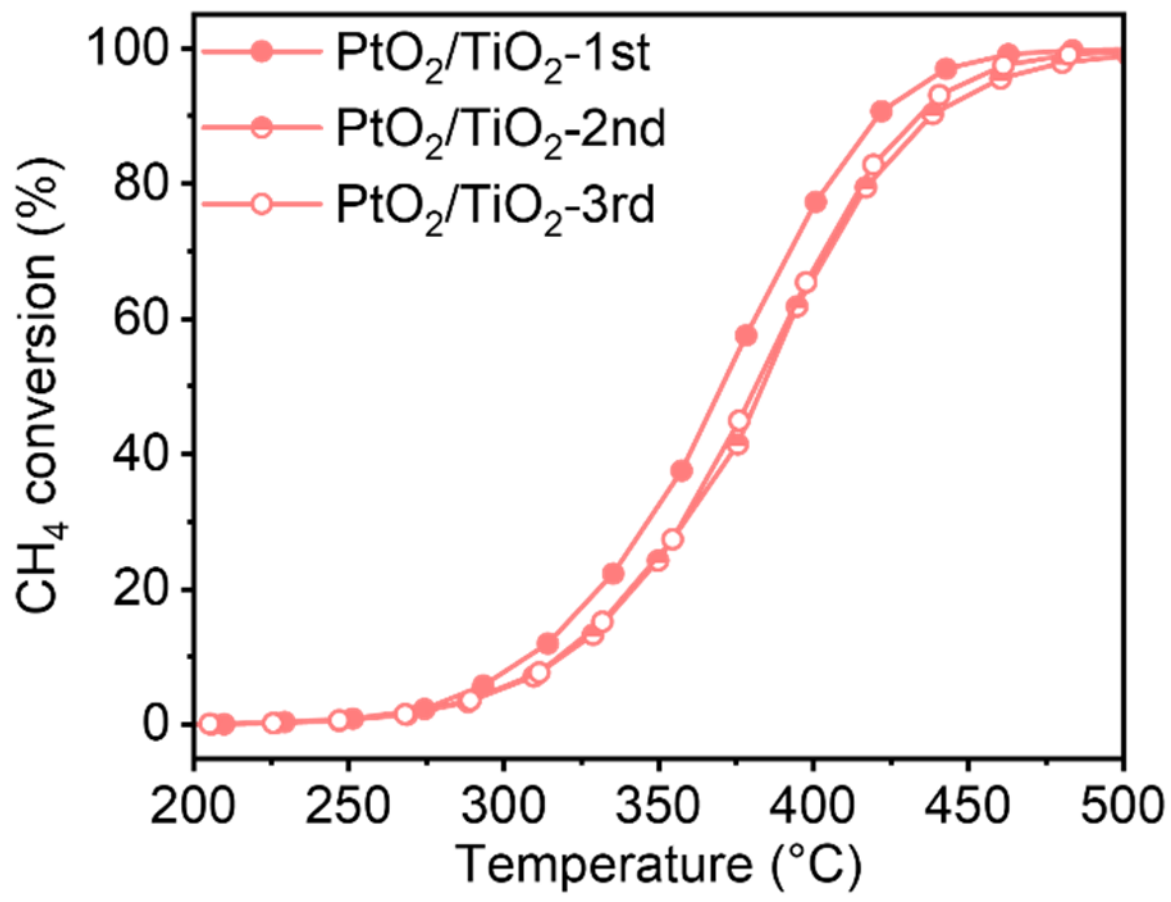

Supplementary Fig. 26 Cycling stability of PtO_2/TiO_2. Repeating ignition cycles of light-off curves of methane oxidation over PtO_2/TiO_2 catalyst.

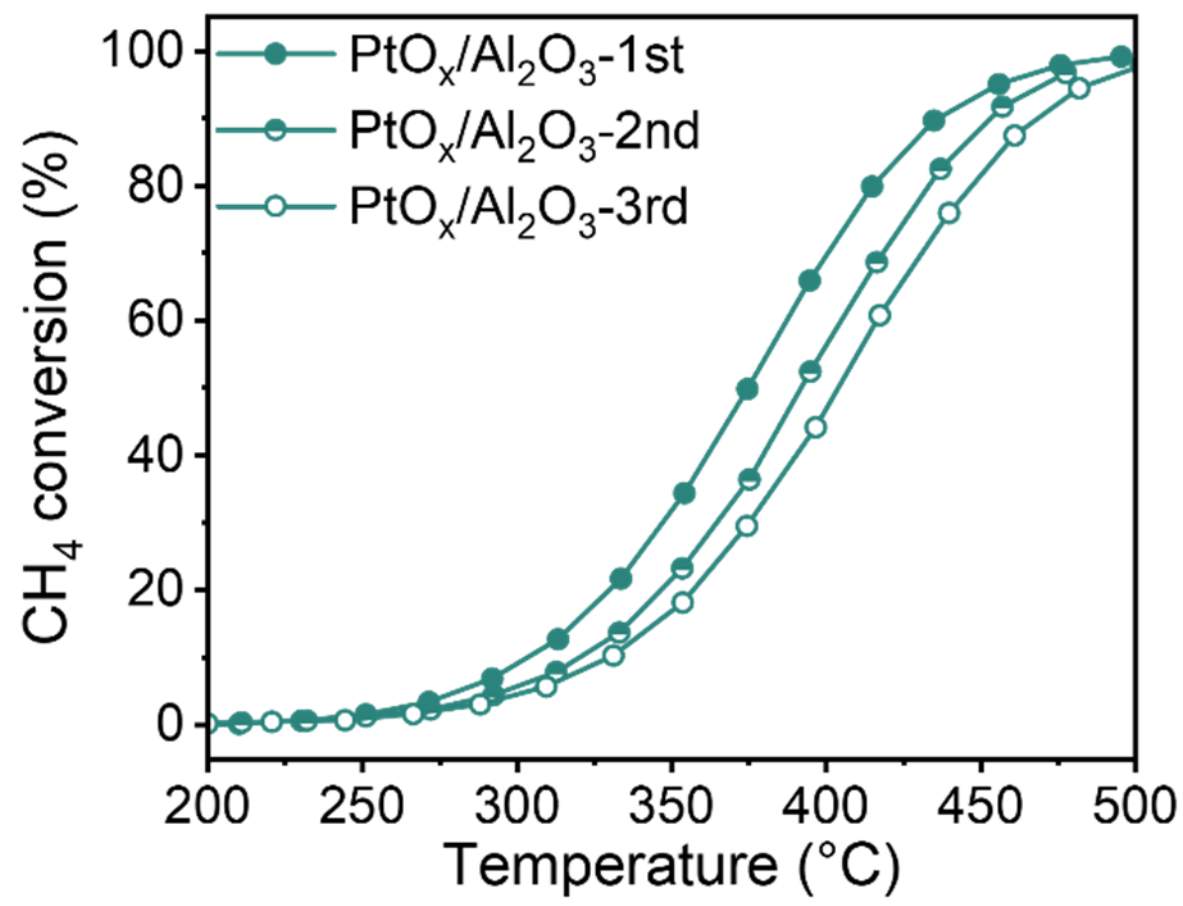

Supplementary Fig. 27 Cycling stability of PtO_x/Al_2O_3. Repeating ignition cycles of light-off curves of methane oxidation over PtO_x/Al_2O_3 catalyst.

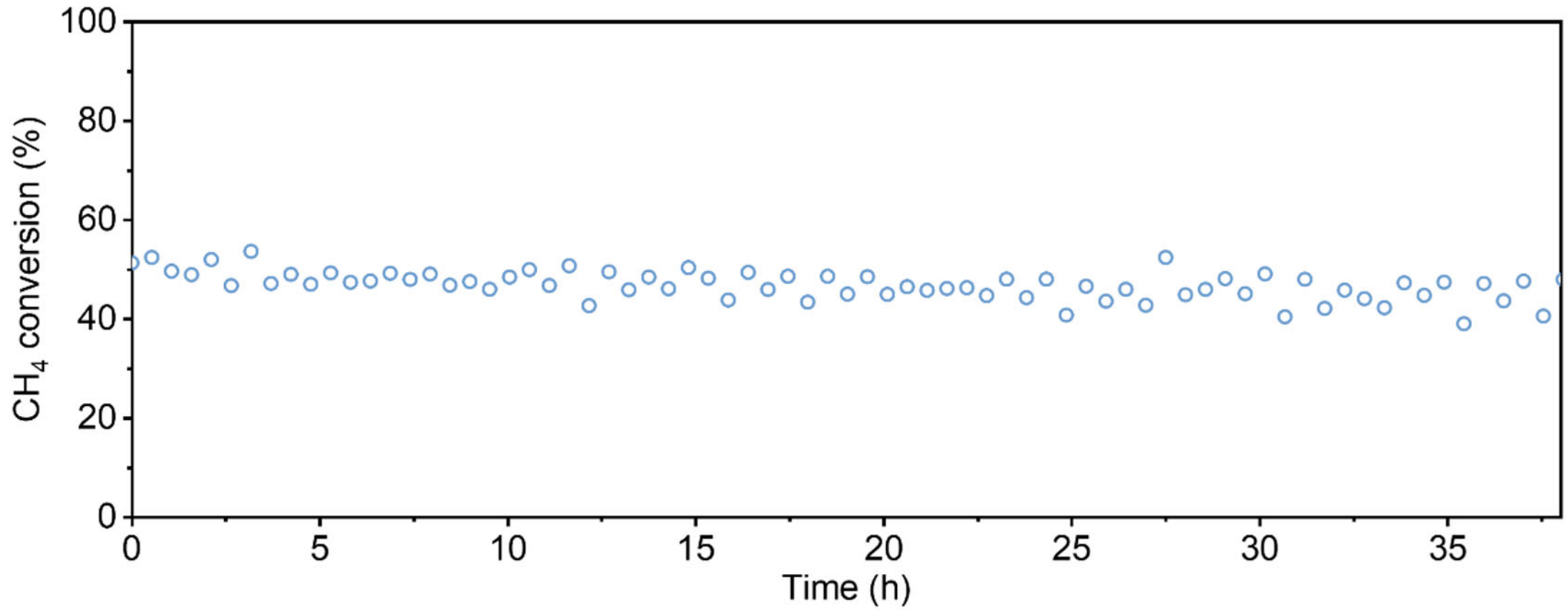

Supplementary Fig. 28 Long-term stability for CH_4 oxidation. Long-term stability test of PtO_2/SnO_2 for CH_4 oxidation under 5 vol% H_2O at 385 °C.

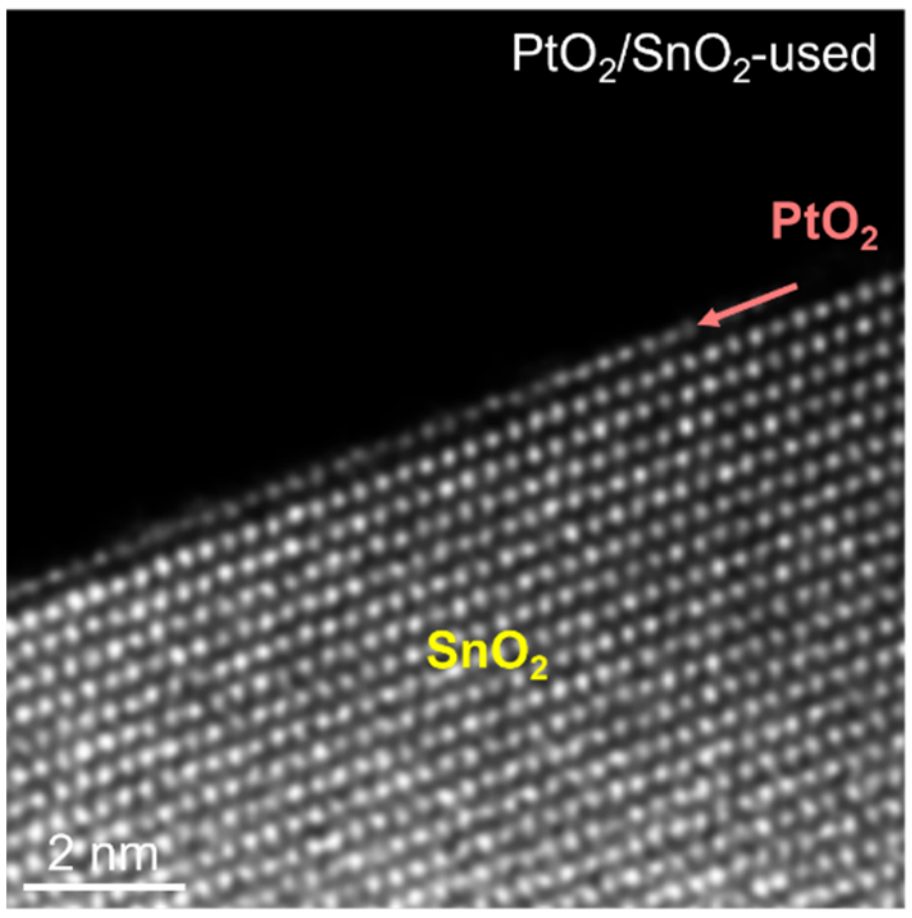

Supplementary Fig. 29 Atomic structure of PtO_2/SnO_2-used. The representative low-damage atomic-scale HAADF-STEM image of PtO_2/SnO_2-used.

Atomic-scale HAADF-STEM imaging of the PtO_2/SnO_2 catalyst after methane oxidation (PtO_2/SnO_2-used) confirmed that the PtO_2-MO retained its atomic monolayer structure on the SnO_2 surface during reaction.

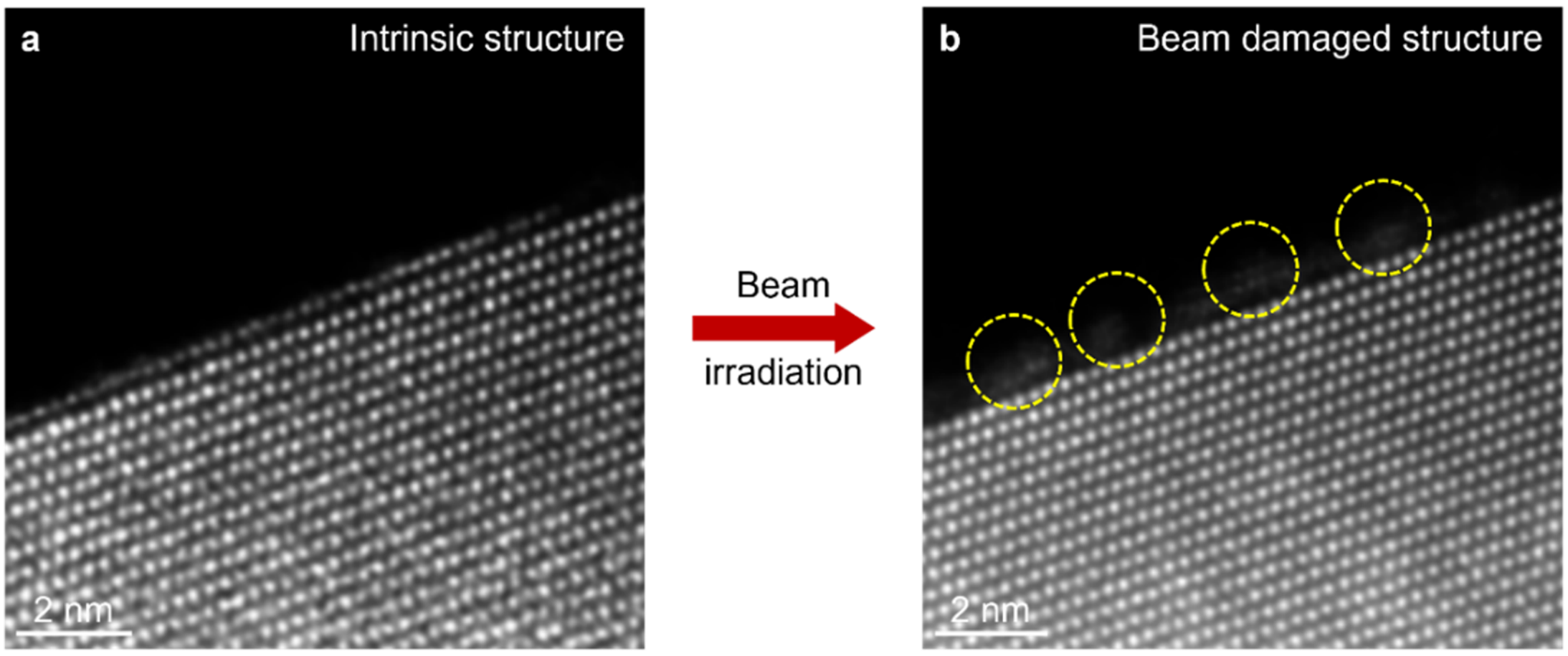

Supplementary Fig. 30 Electron-beam-induced restructuring of PtO_2/SnO_2-used. (a) The HAADF-STEM image of PtO_2/SnO_2-used was acquired by the low-damage HAADF-STEM imaging method under the beam current of ~ 50 pA. (b) The HAADF-STEM image was acquired by observing the same area in (a) continuously under HAADF-STEM mode at the beam current of ~ 50 pA for a few seconds. The yellow circle regions in HAADF-STEM image identify the beam-damaged structure of Pt (small Pt nanoparticles).

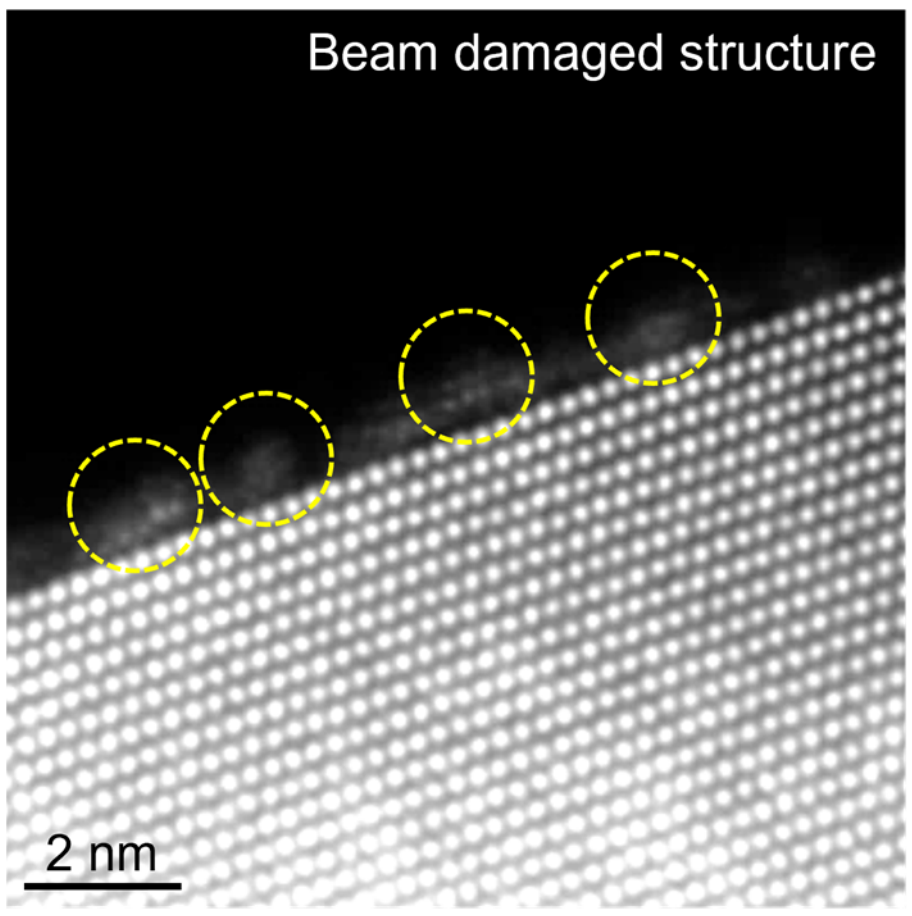

Supplementary Fig. 31 Beam-damaged PtO_2/SnO_2-used. The contrast-adjusted HAADF-STEM image of PtO_2/SnO_2-used after electron beam damage. The image is derived from Supplementary Fig. 30b with only contrast adjusted to improve the visibility of beam-induced Pt-containing nanoparticle-like species, which are marked by yellow circles.

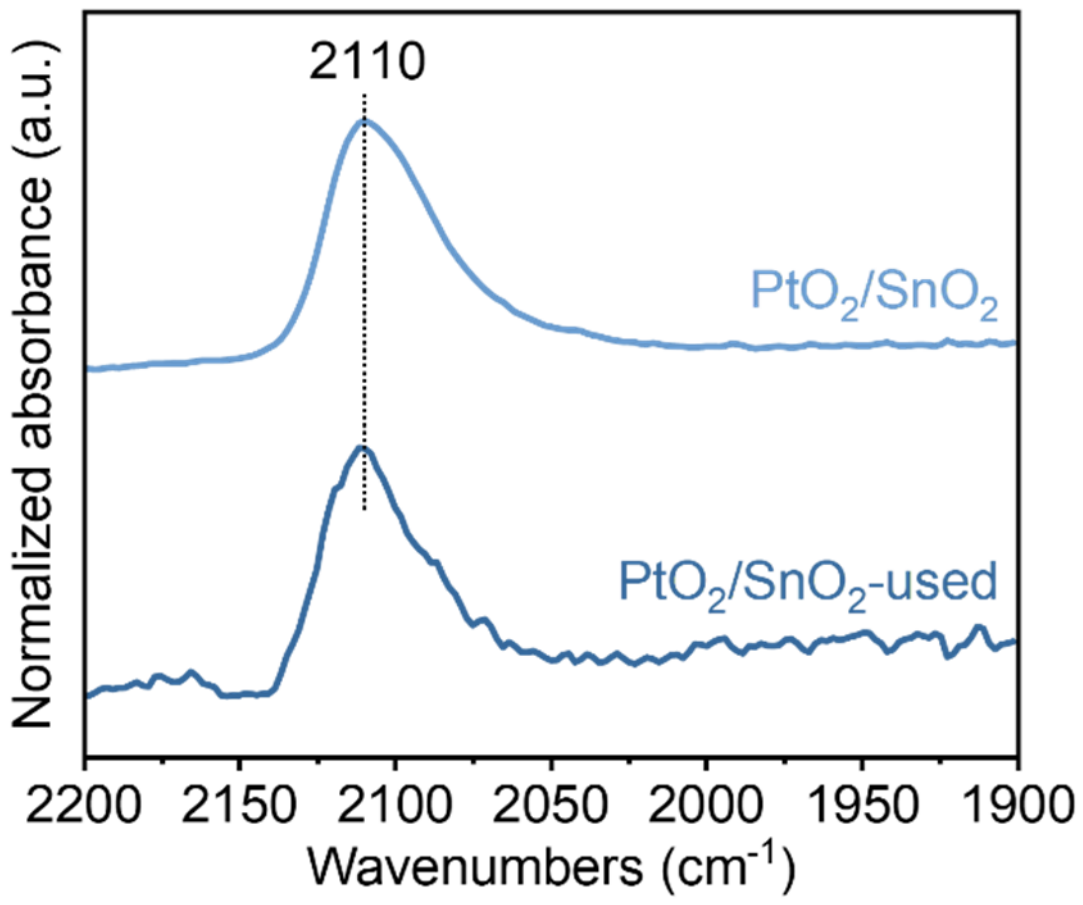

Supplementary Fig. 32 CO-DRIFTS of fresh and used PtO_2/SnO_2. In-situ DRIFTS of CO adsorbed at saturation coverage of PtO_2/SnO_2 and PtO_2/SnO_2-used. The DRIFT spectra are normalized to the maximum intensity of the CO stretch for each catalyst.

In-situ DRIFTS measurements show that the PtO_2/SnO_2-used exhibits a CO stretching band identical to that of the fresh PtO_2/SnO_2, with a peak at 2110 cm^{-1} characteristic of CO adsorbed on high-valent Pt species. This observation demonstrates that the PtO_2-MO retained its electronic state on the SnO_2 surface after methane oxidation.

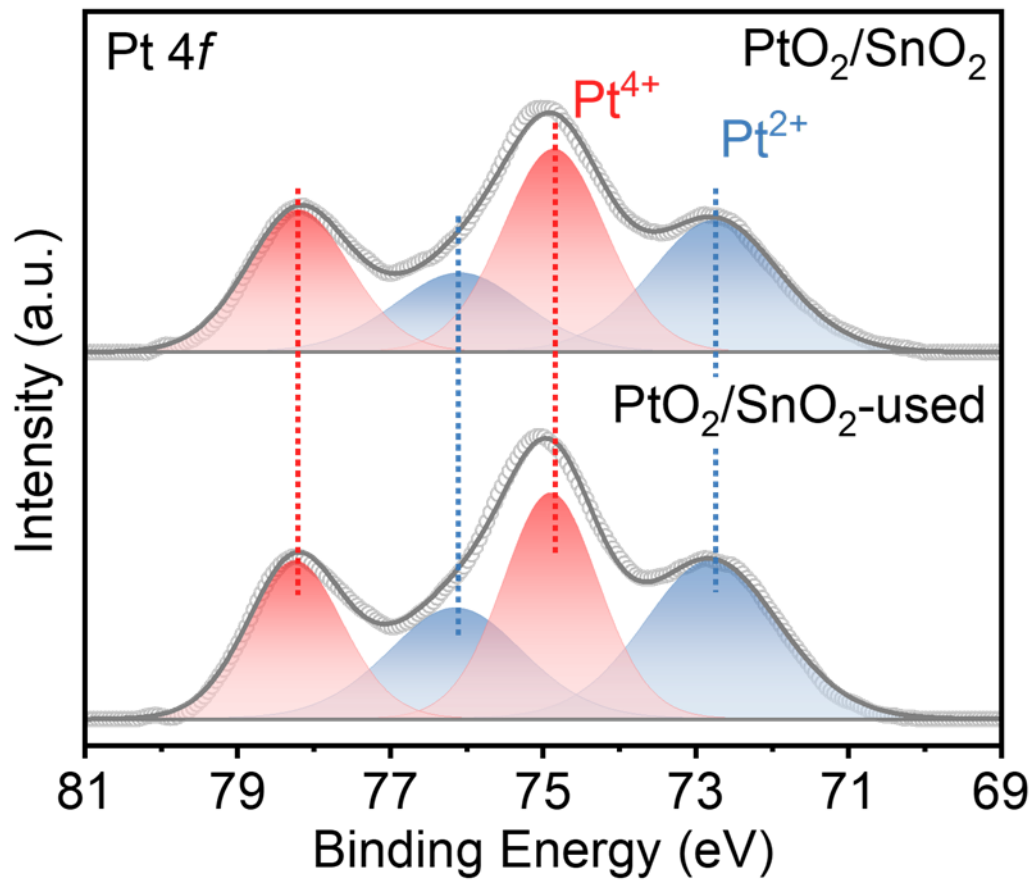

Supplementary Fig. 33 XPS of fresh and used PtO_2/SnO_2. XPS of Pt 4*f* region for PtO_2/SnO_2 and PtO_2/SnO_2-used.

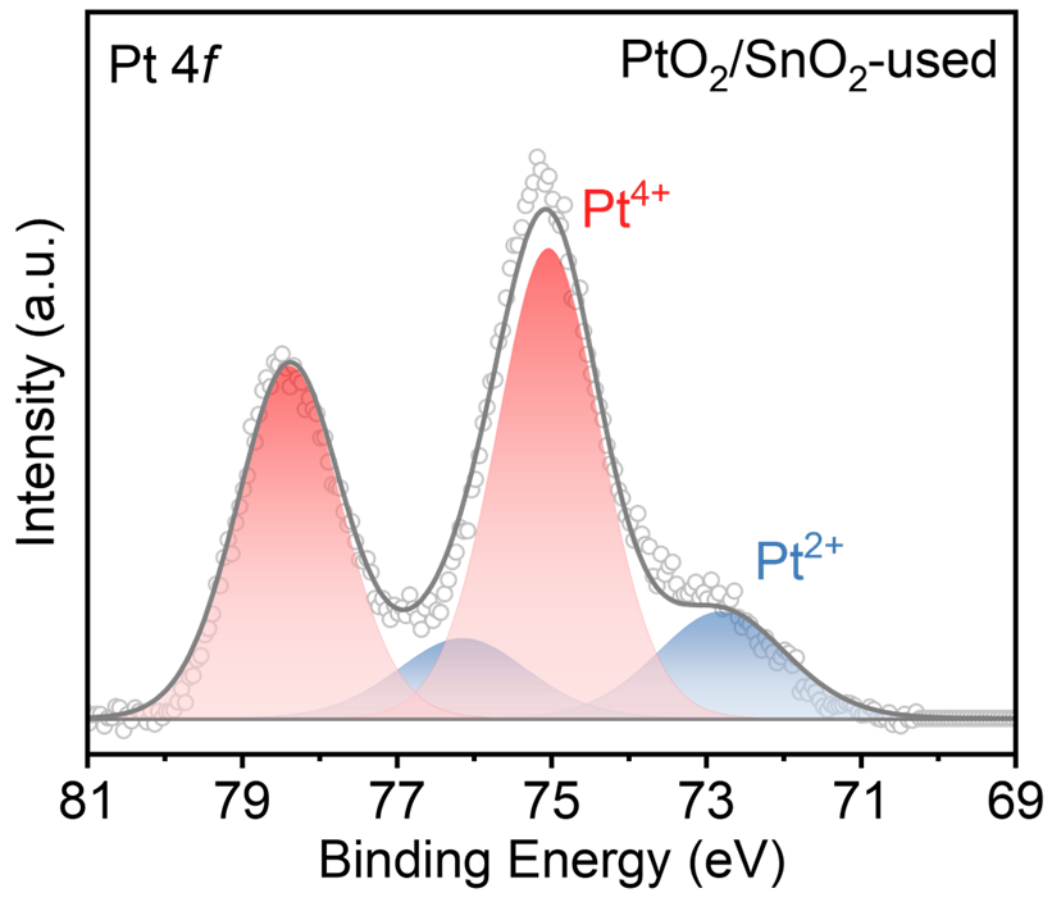

Supplementary Fig. 34 Quasi-in situ XPS of PtO_2/SnO_2-used. Quasi-in situ XPS of Pt 4*f* region for PtO_2/SnO_2-used after three methane oxidation cycles. The sample was transferred to the XPS analysis chamber under vacuum after reaction pretreatment without exposure to air.

The quasi-in situ Pt 4*f* XPS spectrum shows that Pt remains predominantly oxidized after methane oxidation, with Pt^{2+} and Pt^{4+} fractions of 22.0% and 78.0%, respectively. The higher Pt^{4+} fraction compared with conventionally measured ex situ samples indicates that the oxidized Pt state observed after reaction is not an artifact of air exposure during sample handling and that conventional ex situ XPS does not overestimate the Pt oxidation state after reaction.

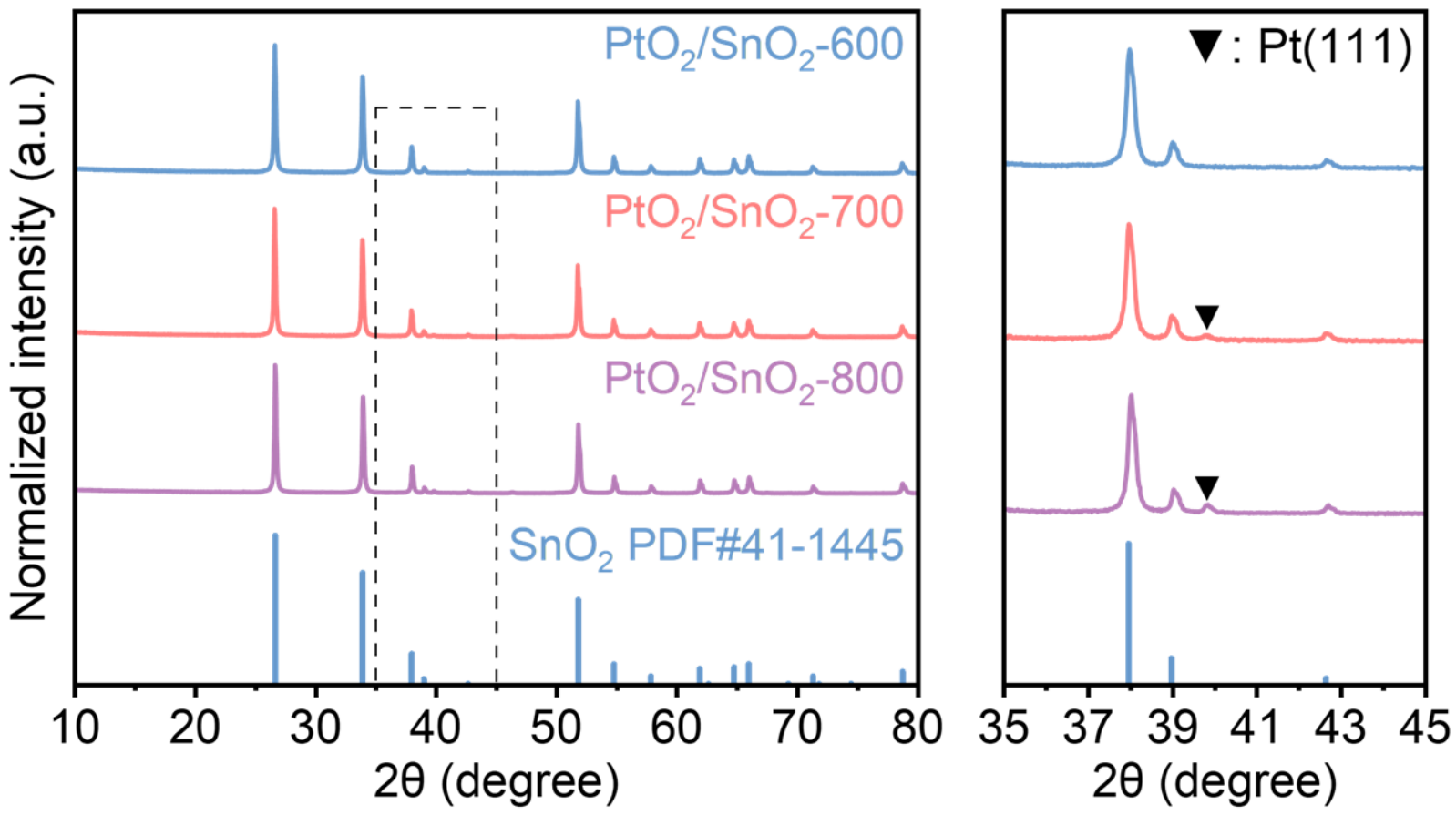

Supplementary Fig. 35 Thermal stability of PtO_2/SnO_2. The XRD patterns of PtO_2/SnO_2-600, PtO_2/SnO_2-700, and PtO_2/SnO_2-800.

To evaluate the thermal stability limit of Pt oxide species in PtO_2/SnO_2, the catalyst was further calcined in air at 600, 700, and 800 °C. The resulting samples are denoted as PtO_2/SnO_2-600, PtO_2/SnO_2-700, and PtO_2/SnO_2-800, respectively. As shown in Supplementary Fig. 35, no obvious Pt-related crystalline reflections are observed after calcination at 600 °C, indicating that no XRD-detectable Pt-containing particles are formed at this temperature. In contrast, a weak diffraction peak assignable to metallic Pt(111) appears after calcination at 700 °C and becomes more pronounced after calcination at 800 °C. These results indicate that the Pt oxide species in PtO_2/SnO_2 begin to thermally decompose and aggregate into XRD-detectable metallic Pt particles at approximately 700 °C under harsh air-calcination conditions.

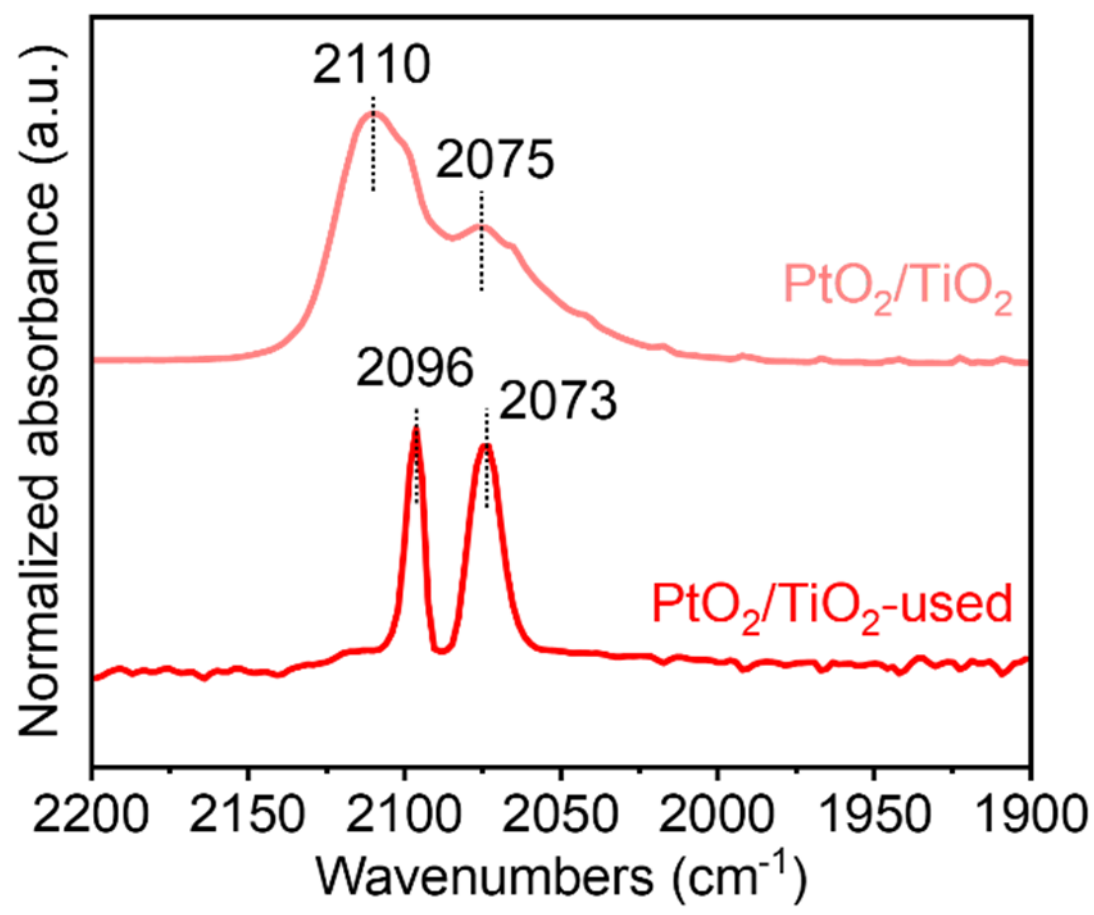

Supplementary Fig. 36 CO-DRIFTS of fresh and used PtO_2/TiO_2. In-situ DRIFTS of CO adsorbed at saturation coverage of PtO_2/TiO_2 and PtO_2/TiO_2-used. The DRIFT spectra are normalized to the maximum intensity of the CO stretch for each catalyst.

In-situ DRIFTS measurements show that, compared with the characteristic band at 2110 cm^{-1} and the additional band at 2075 cm^{-1} in fresh PtO_2/TiO_2, the PtO_2/TiO_2-used sample exhibits distinct features at 2096 cm^{-1} and 2073 cm^{-1}, attributable to CO adsorption on metallic Pt^0 species with different coordination environments. Although no Pt^0 signals are observed in the XPS of PtO_2/TiO_2-used (Supplementary Fig. 37), the emergence of these CO bands indicates that partial reduction of Pt occurs upon CO exposure under DRIFTS conditions. Furthermore, the differences in CO adsorption bands between PtO_2/TiO_2 and PtO_2/TiO_2-used confirm that the coordinated environment of Pt-MO species on TiO_2 undergoes changes during the methane oxidation reaction.

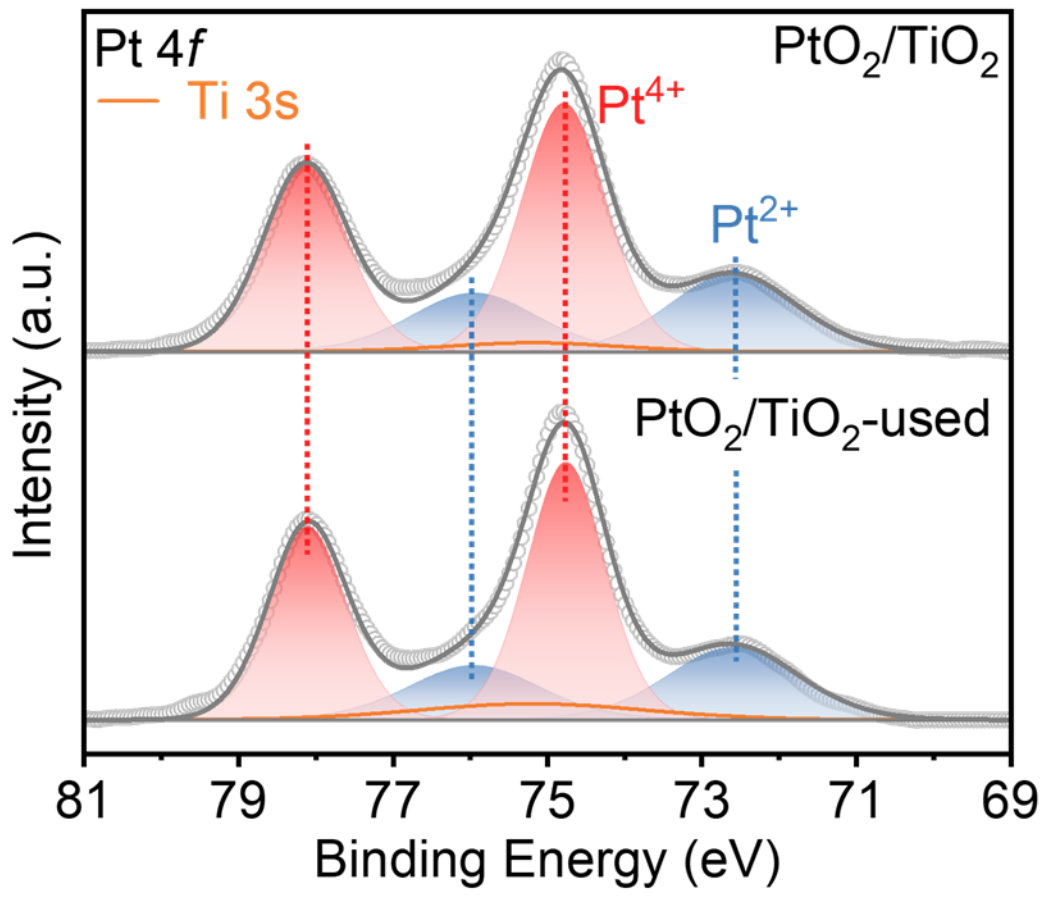

Supplementary Fig. 37 XPS of fresh and used PtO_2/TiO_2. XPS of Pt 4*f* region for PtO_2/TiO_2 and PtO_2/TiO_2-used.

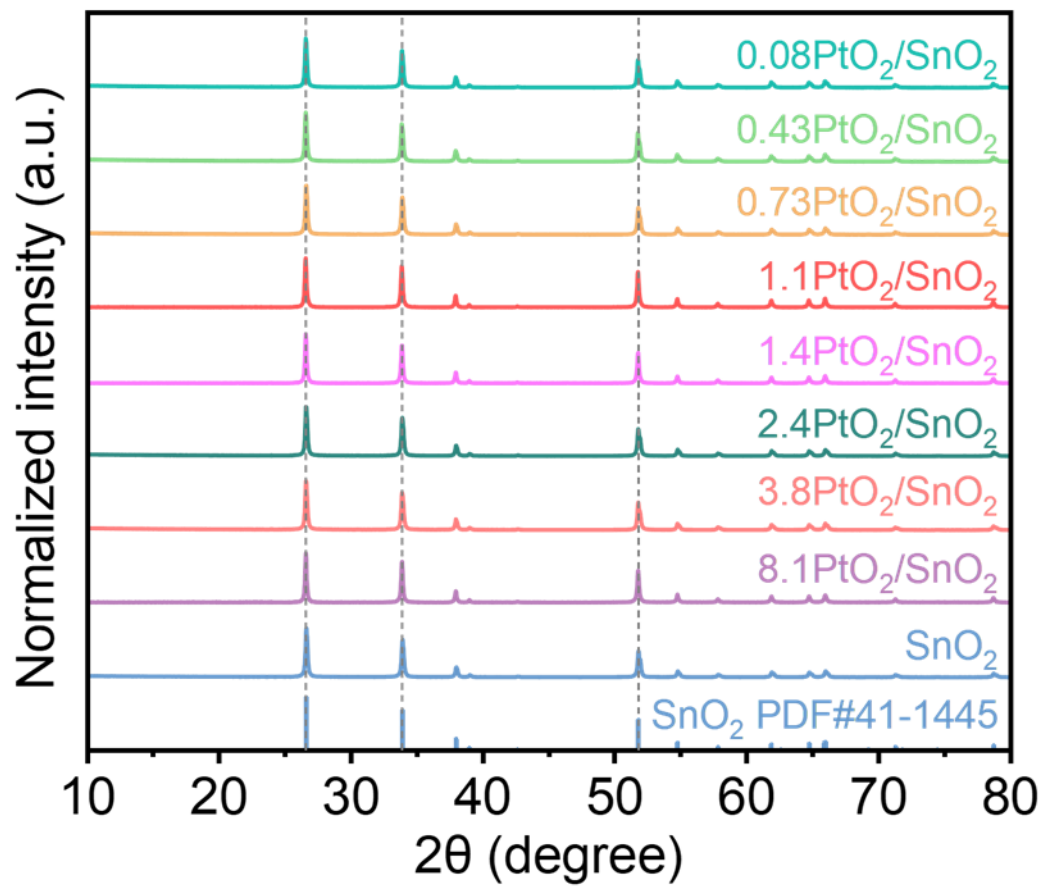

Supplementary Fig. 38 XRD characterization of PtO_2/SnO_2 with different Pt loadings. The XRD patterns of PtO_2/SnO_2 catalysts with different Pt loadings. All samples were prepared using the same impregnation-calcination protocol and calcined in air at 500 °C for 2 h.

No X-ray diffraction (XRD) peaks associated with Pt were observed for any of the PtO_2/SnO_2 catalysts with different Pt loadings, indicating that the Pt species were highly dispersed on SnO_2 even at loadings as high as 8.1 wt%.

Supplementary Note 3: The theoretical maximum dispersion threshold for a PtO_2 monolayer on SnO_2 (X%), defined based on the SnO_2 (110) facet model, was calculated by the following equations:

$$\sigma_{\mathrm{Sn}} = \sigma_{\mathrm{Pt}} \tag{1}$$

$$X\% = \frac{\sigma_{\mathrm{Pt}} \times M_{\mathrm{Pt}} \times S_{\mathrm{BET}}}{1+\sigma_{\mathrm{Pt}} \times M_{\mathrm{Pt}} \times S_{\mathrm{BET}}} \times 100\% \tag{2}$$

Where σ_{Sn} (mol m^{-2}) is the areal density of Sn atoms on the SnO_2 (110) surface, σ_{Pt} (mol m^{-2}) is the areal density of Pt atoms on the PtO_2/SnO_2 (110) surface, M_{Pt} (g mol^{-1}) is the molar mass of Pt and S_{BET} (m^2 g^{-1}) is the specific surface area of the SnO_2 support.

In our epitaxial growth model, each Pt site in the PtO_2 layer corresponds to one Sn site on the SnO_2 surface. Specifically, based on the structural model we established, the

σ_{Sn} was determined to be approximately 9.17 atoms nm^{-2} (equivalent to 1.52×10^{-5} mol m^{-2}). By incorporating the measured S_{BET} of 6.7 m^2 g^{-1}, the calculated threshold is 1.95 wt%.

Because the $SnO_2(110)$ facet has the lowest surface Sn atom site density (that is, the largest surface area per surface site) among the considered facets, this value represents an upper bound. In addition, real faceted/polyhedral SnO_2 particles contain multiple facets, steps, defects, and partially inaccessible surface areas, which may further reduce the effective capacity for forming an epitaxial PtO_2 monolayer. Therefore, the practical PtO_2 monolayer dispersion threshold on SnO_2 is expected to be close to or slightly lower than 1.95 wt%.

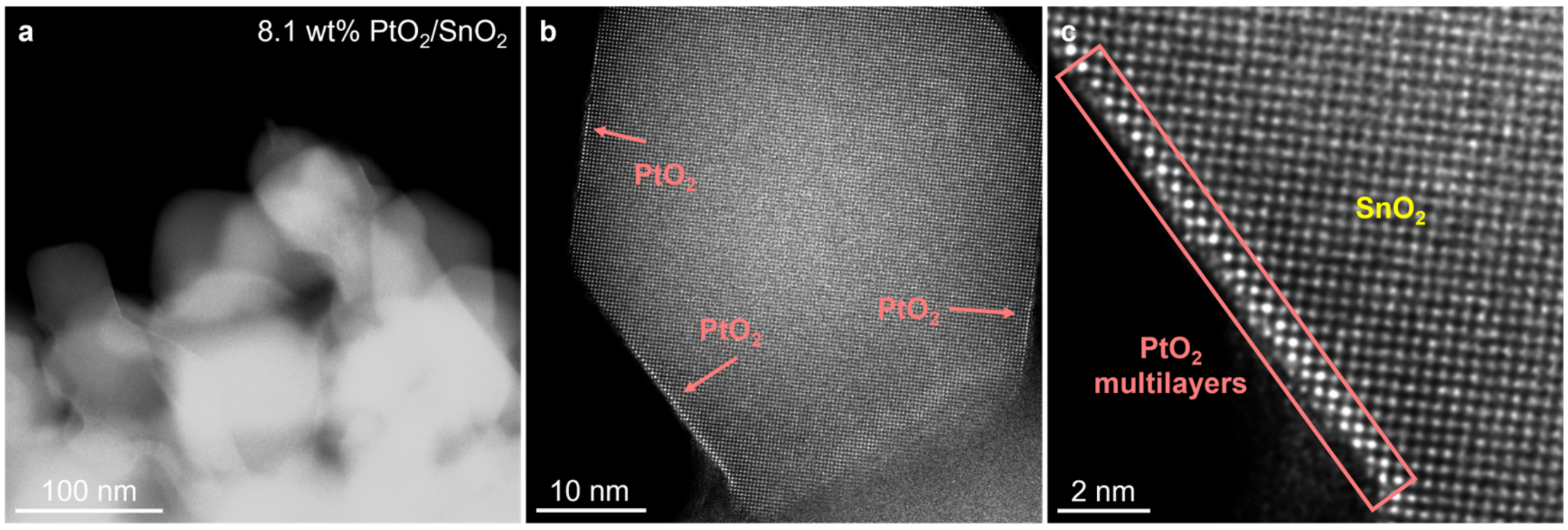

Supplementary Fig. 39 HAADF-STEM images of 8.1 wt% PtO_2/SnO_2. Representative (a, b) low-magnification and (c) atomic-scale HAADF-STEM images of 8.1 wt% PtO_2/SnO_2 catalyst.

Owing to the high surface coverage of PtO_2 on SnO_2, the Pt species in the 8.1 wt% PtO_2/SnO_2 catalyst could be directly identified by Z-contrast in HAADF-STEM imaging. The atomic-scale HAADF-STEM images reveal the presence of 2-4 atomic layers of PtO_2 on the SnO_2 surface.

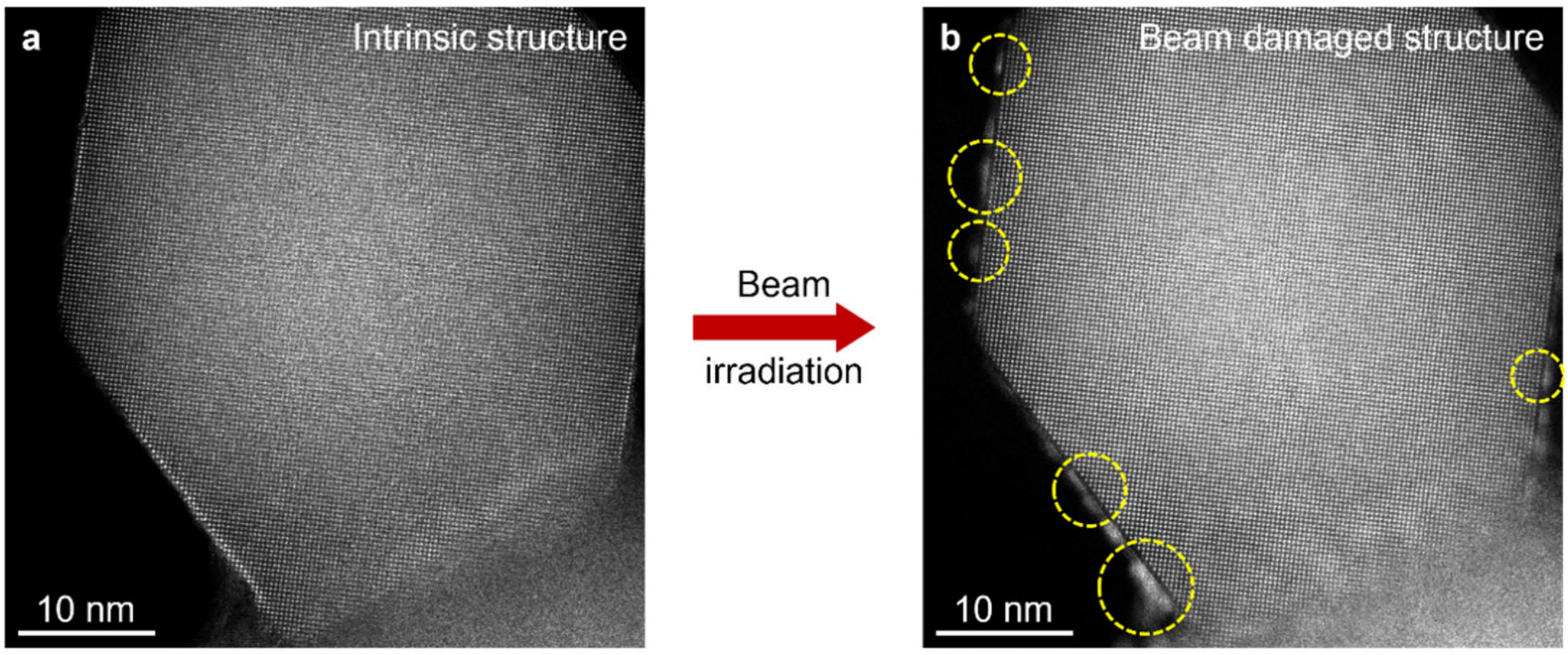

Supplementary Fig. 40 Electron-beam-induced restructuring of 8.1 wt% PtO_2/SnO_2. (a) The HAADF-STEM image of 8.1 wt% PtO_2/SnO_2 was acquired by the low-damage HAADF-STEM imaging method under the beam current of ~ 50 pA. (b) The HAADF-STEM image was acquired by observing the same area in (a) continuously under HAADF-STEM mode at the beam current of ~ 50 pA for a few seconds. The yellow circle regions in HAADF-STEM image identify the beam-damaged structure of Pt (small Pt nanoparticles).

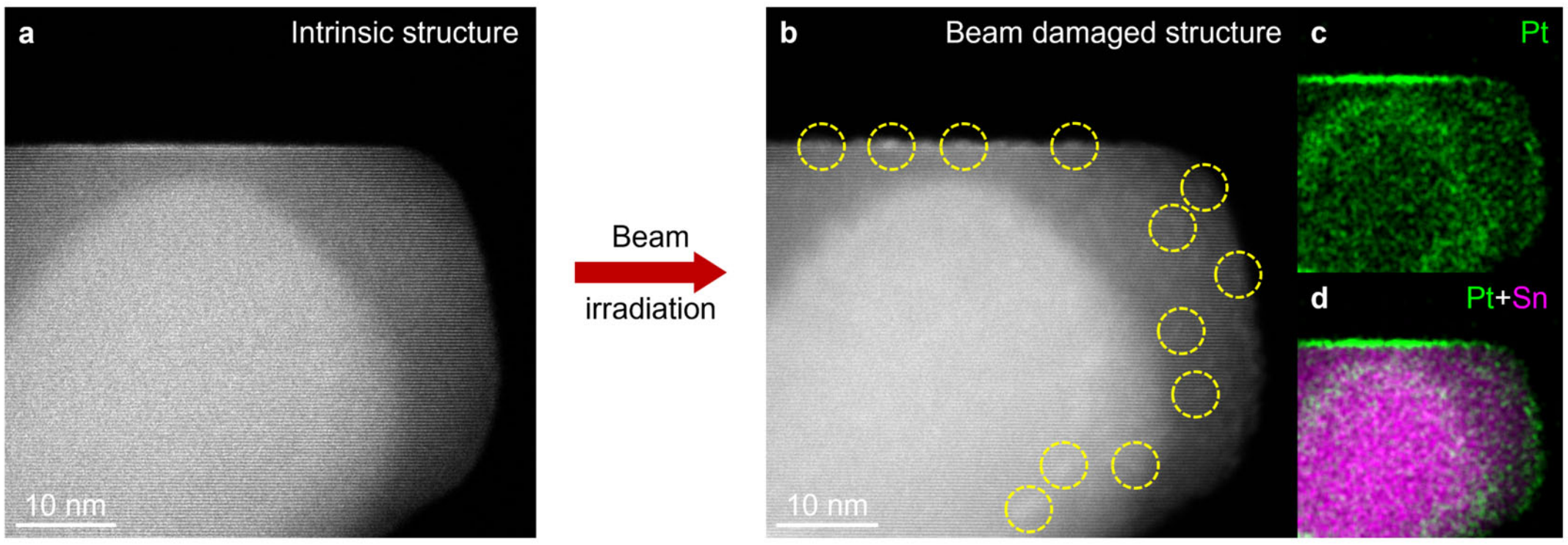

Supplementary Fig. 41 Electron-beam-induced restructuring and EDS mapping of 8.1 wt% PtO_2/SnO_2. (a) The HAADF-STEM image of 8.1 wt% PtO_2/SnO_2 was acquired by the low-damage HAADF-STEM imaging method under the beam current of ~ 50 pA. (b) The HAADF-STEM image was acquired by observing the same area in (a) continuously under HAADF-STEM mode at the beam current of ~ 50 pA for a few seconds. (c-d) The corresponding EDS elemental mapping images of (c) Pt and (d) Pt+Sn compositions. The yellow circle regions in HAADF-STEM image identify the beam-damaged structure of Pt (small Pt nanoparticles).

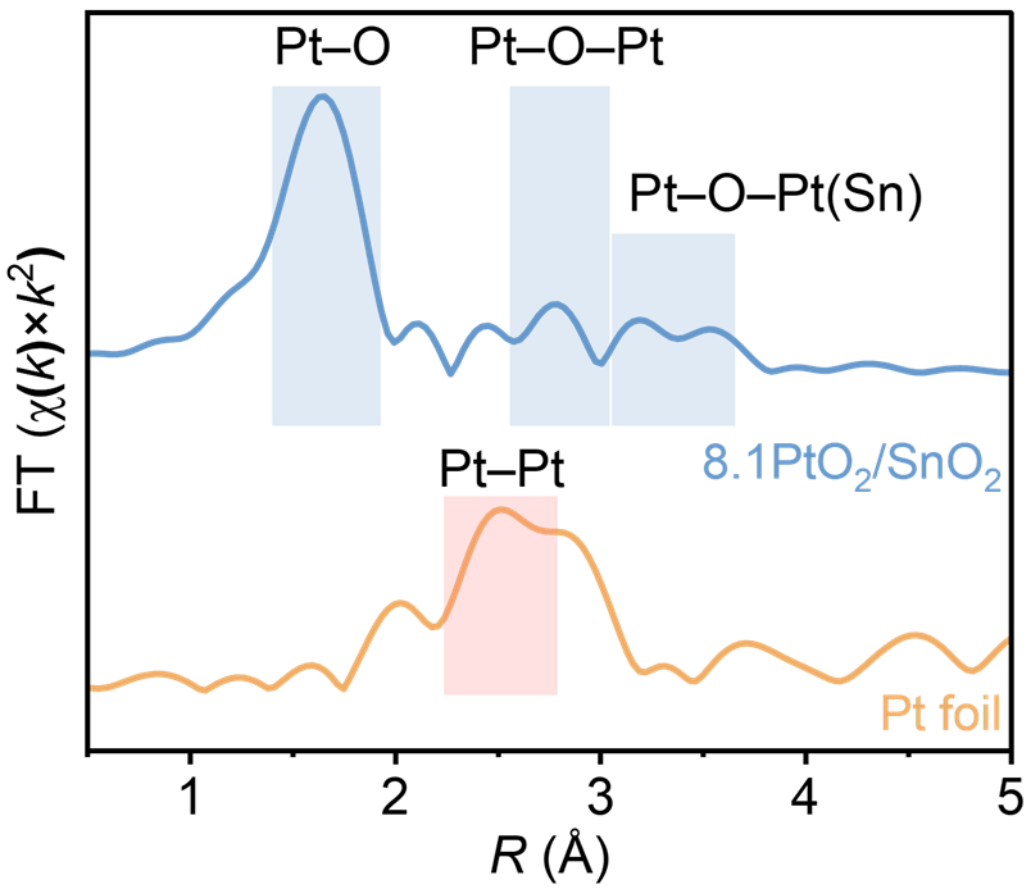

Supplementary Fig. 42 Pt L_3-edge EXAFS of 8.1 wt% PtO_2/SnO_2. FT-EXAFS spectra at the Pt L_3-edge of 8.1 wt% PtO_2/SnO_2 and Pt foil.

Supplementary Note 4: Pt L_3-edge XAS and EXAFS fitting were performed for the high-loading 8.1 wt% PtO_2/SnO_2 sample to further examine the local coordination environment of Pt. The Fourier transform of the k^2-weighted EXAFS spectrum shows a dominant first-shell peak at approximately 1.7 Å before phase correction, corresponding to Pt–O coordination, together with higher-shell features at approximately 2.8 and 3.4 Å before phase correction (Supplementary Fig. 42). Quantitative EXAFS fitting gives a Pt–O coordination number of 4.4 ± 0.3 with a Pt–O distance of 2.01 ± 0.01 Å, and a Pt–(O)–Pt coordination number of 6.4 ± 3.5 with a Pt–(O)–Pt distance of 3.13 ± 0.02 Å (Supplementary Fig. 43 and Supplementary Table 7). These results indicate that Pt remains mainly in an oxidic Pt–O coordination environment even at high Pt loading, consistent with the two-dimensional oxidic structures observed by HAADF-STEM.

Representative scattering paths derived from the PtO_2/SnO_2 structural model show that the long Pt–(O)–Pt path and the interfacial Pt–(O)–Sn path are close in distance (Supplementary Table 8). Therefore, the weak high-R feature was fitted as an unresolved Pt–(O)–M contribution at 3.84 ± 0.04 Å, where M represents Pt or Sn. Because the Pt–(O)–Pt and Pt–(O)–Sn contributions strongly overlap, this high-R shell is used only as auxiliary evidence for the higher-shell interfacial structure, and its

coordination number is not quantitatively interpreted.

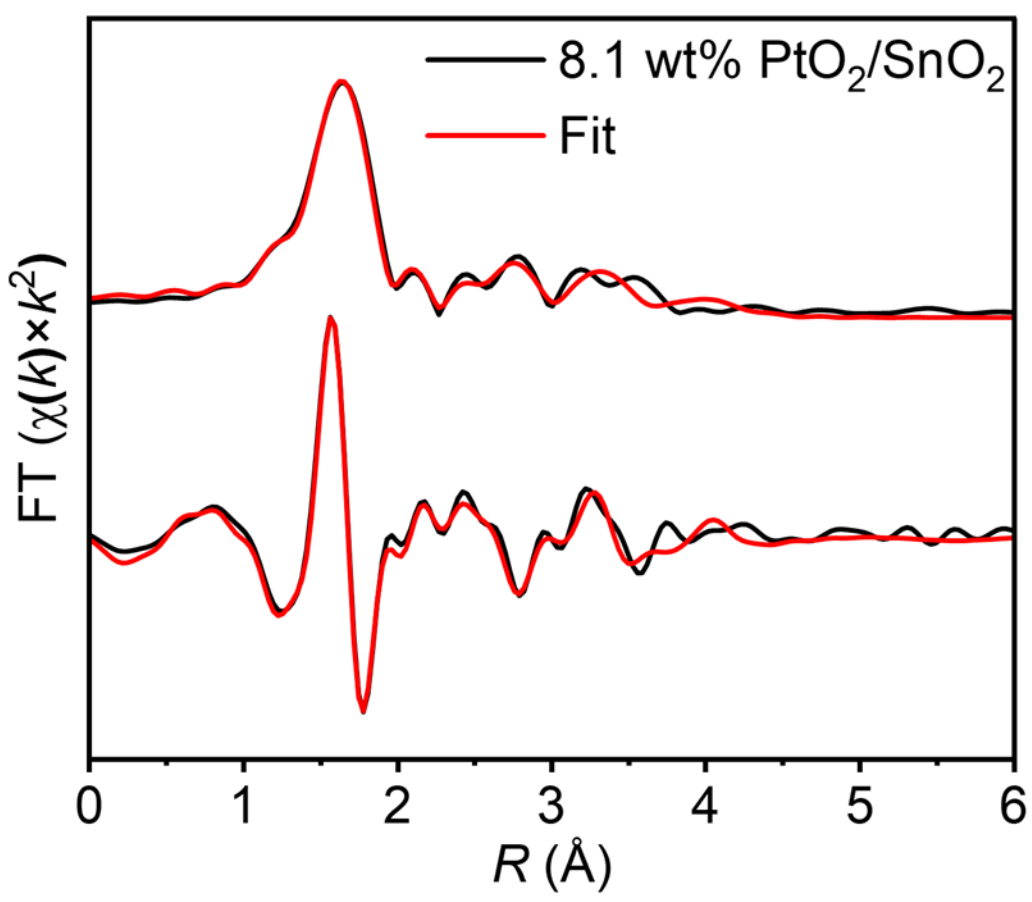

Supplementary Fig. 43 EXAFS fitting of 8.1 wt% PtO_2/SnO_2. The fit of the FT EXAFS spectra (top) and the FT imaginary portion (bottom) of 8.1 wt% PtO_2/SnO_2.

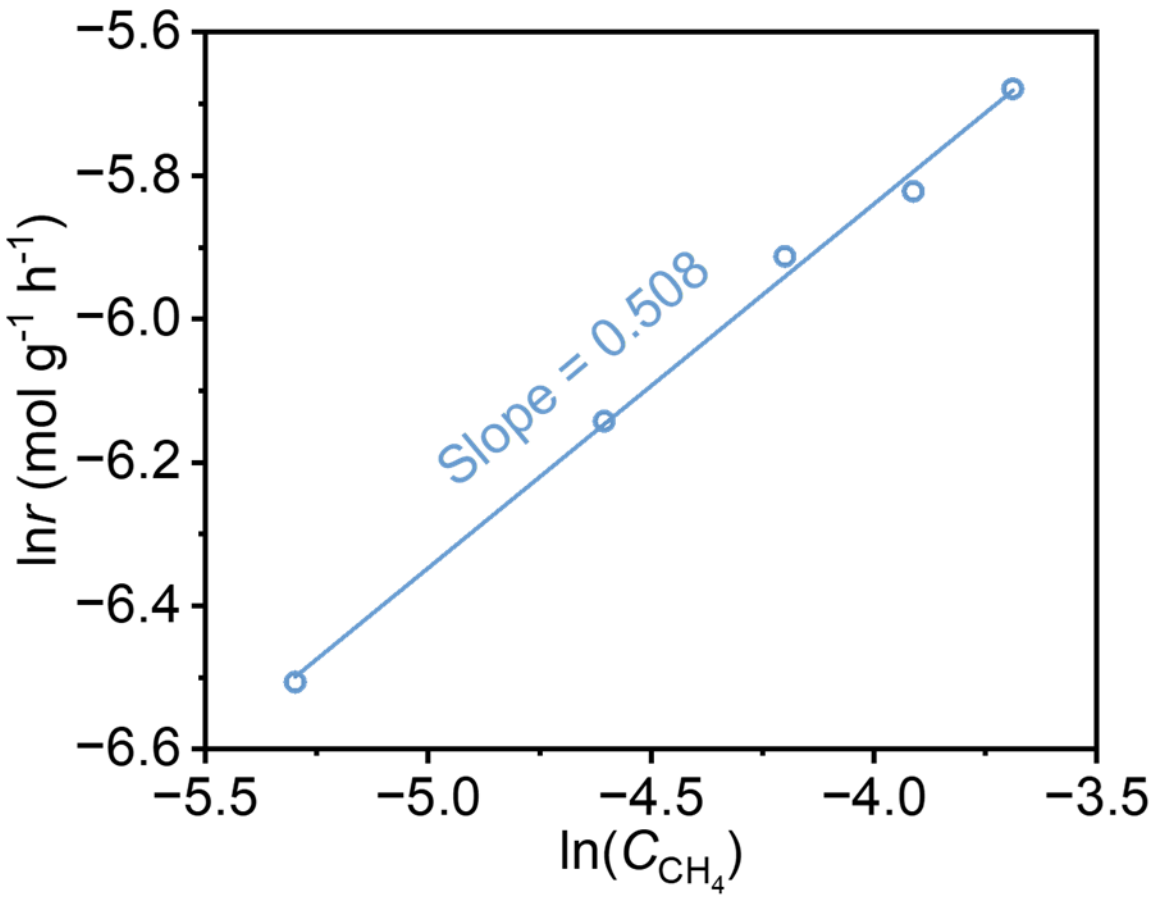

Supplementary Fig. 44 CH_4 reaction order over PtO_2/SnO_2. The reaction order of CH_4 in methane oxidation of PtO_2/SnO_2 catalyst.

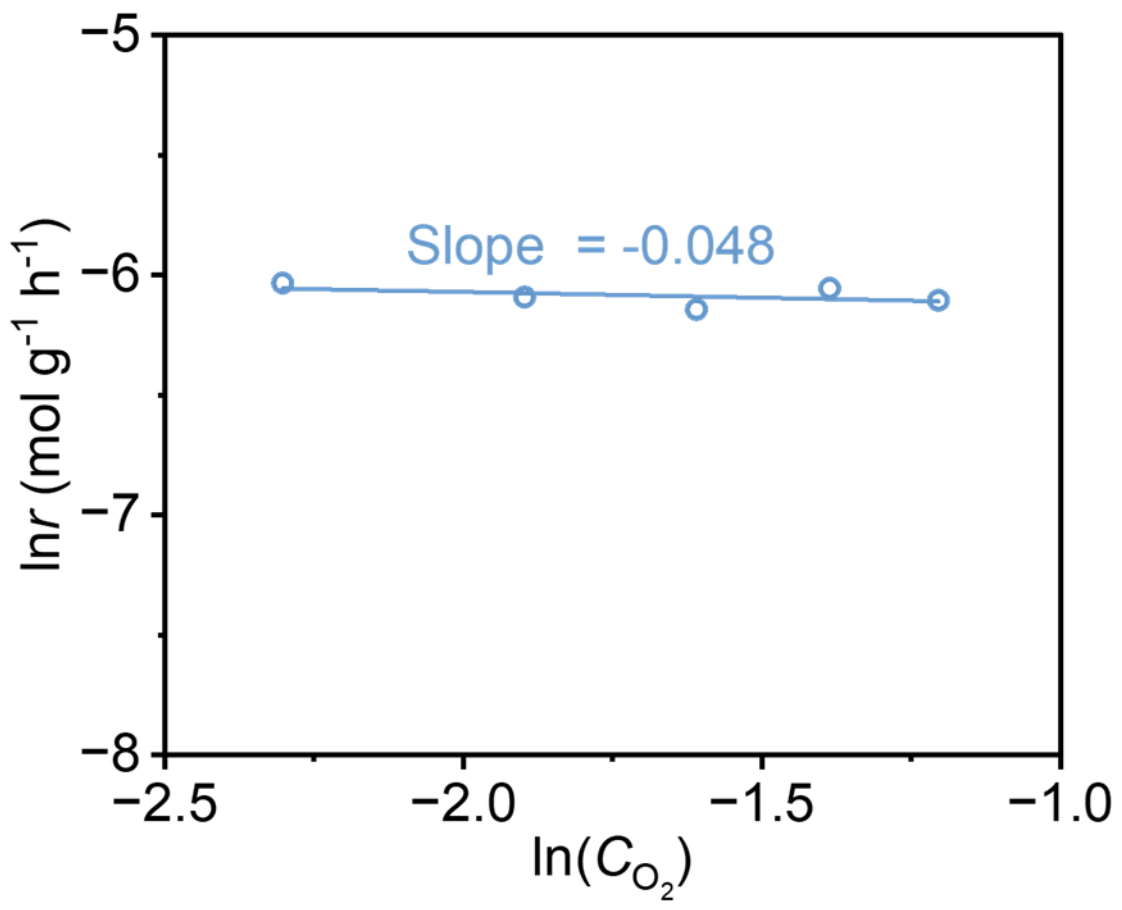

Supplementary Fig. 45 O_2 reaction order over PtO_2/SnO_2. The reaction order of O_2 in methane oxidation of PtO_2/SnO_2 catalyst.

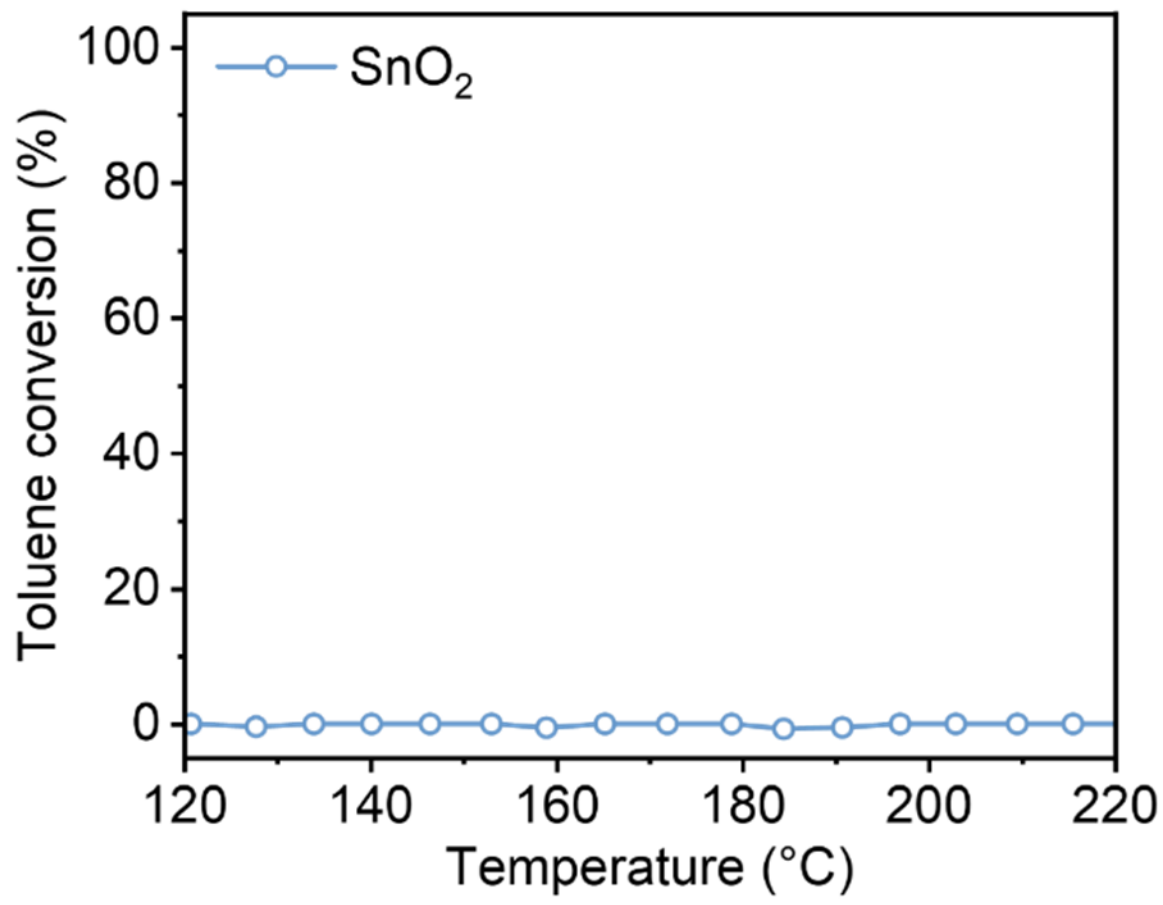

Supplementary Fig. 46 Toluene oxidation over SnO_2. The light-off curve of toluene oxidation over SnO_2 support.

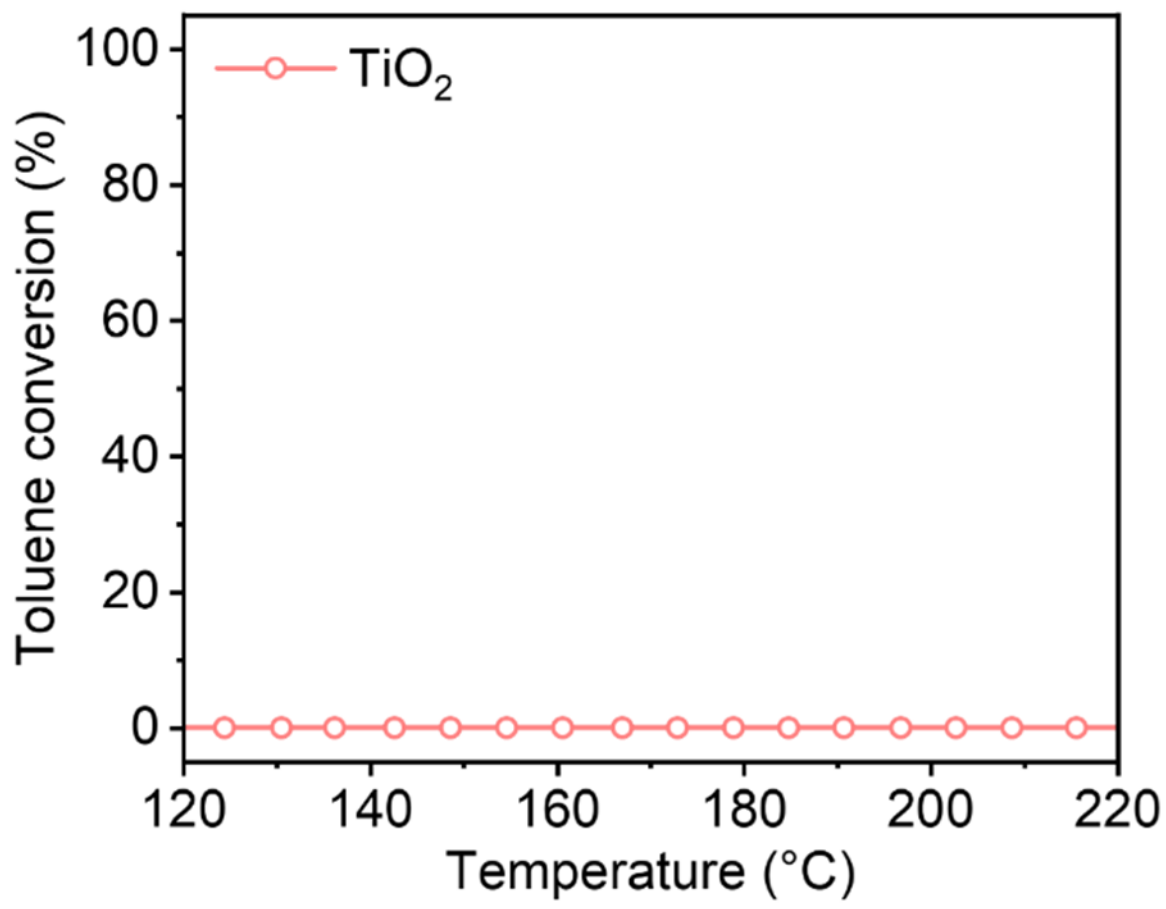

Supplementary Fig. 47 Toluene oxidation over TiO_2. The light-off curve of toluene oxidation over TiO_2 support.

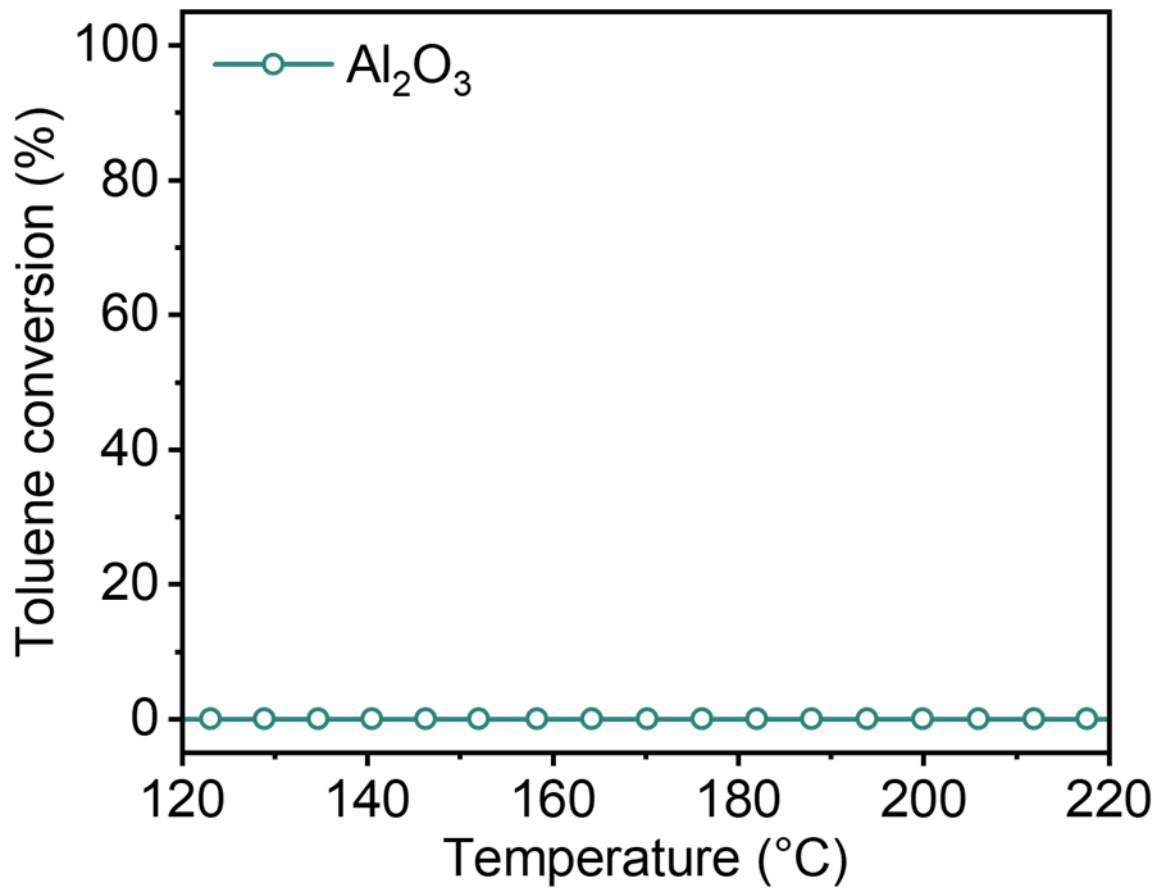

Supplementary Fig. 48 Toluene oxidation over Al_2O_3. The light-off curve of toluene oxidation over Al_2O_3 support.

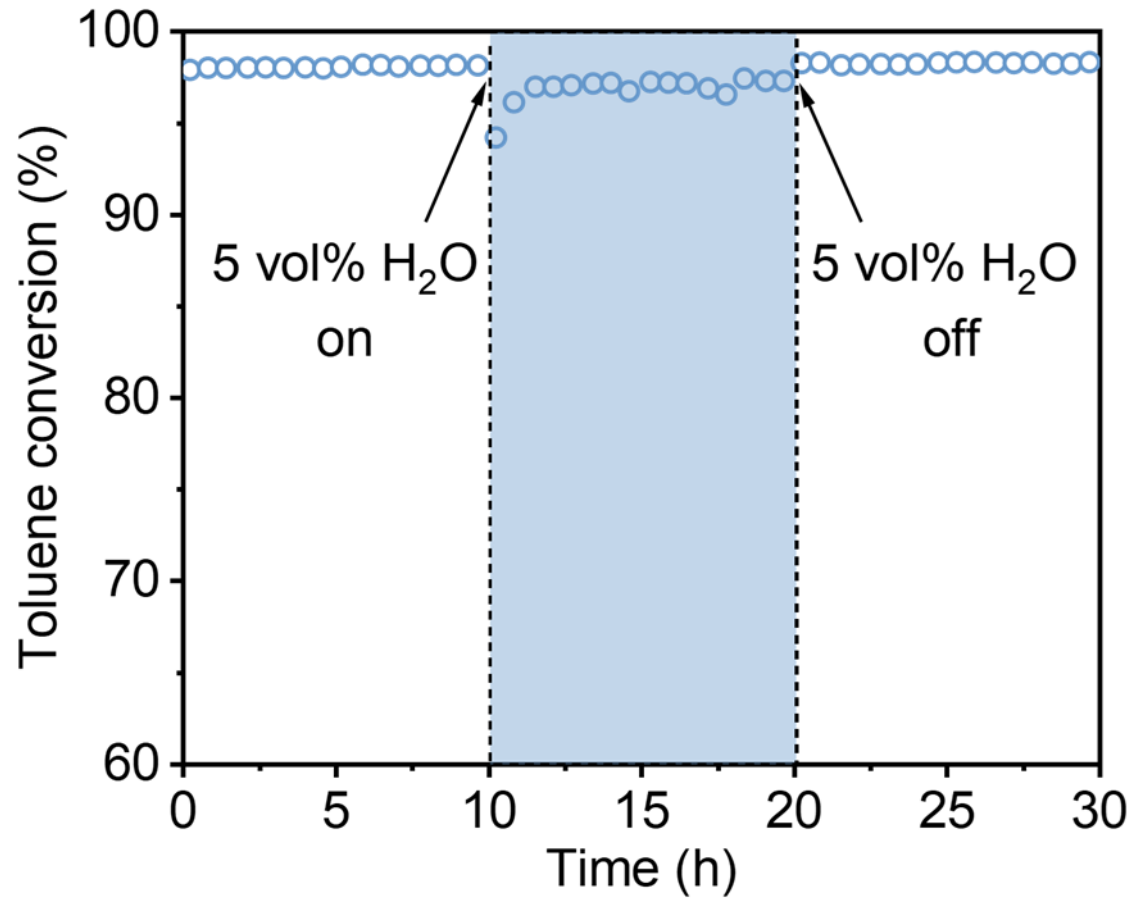

Supplementary Fig. 49 Long-term stability for toluene oxidation. The long-term stability test of PtO_2/SnO_2 for toluene oxidation under dry and 5 vol% H_2O conditions at 185 °C.

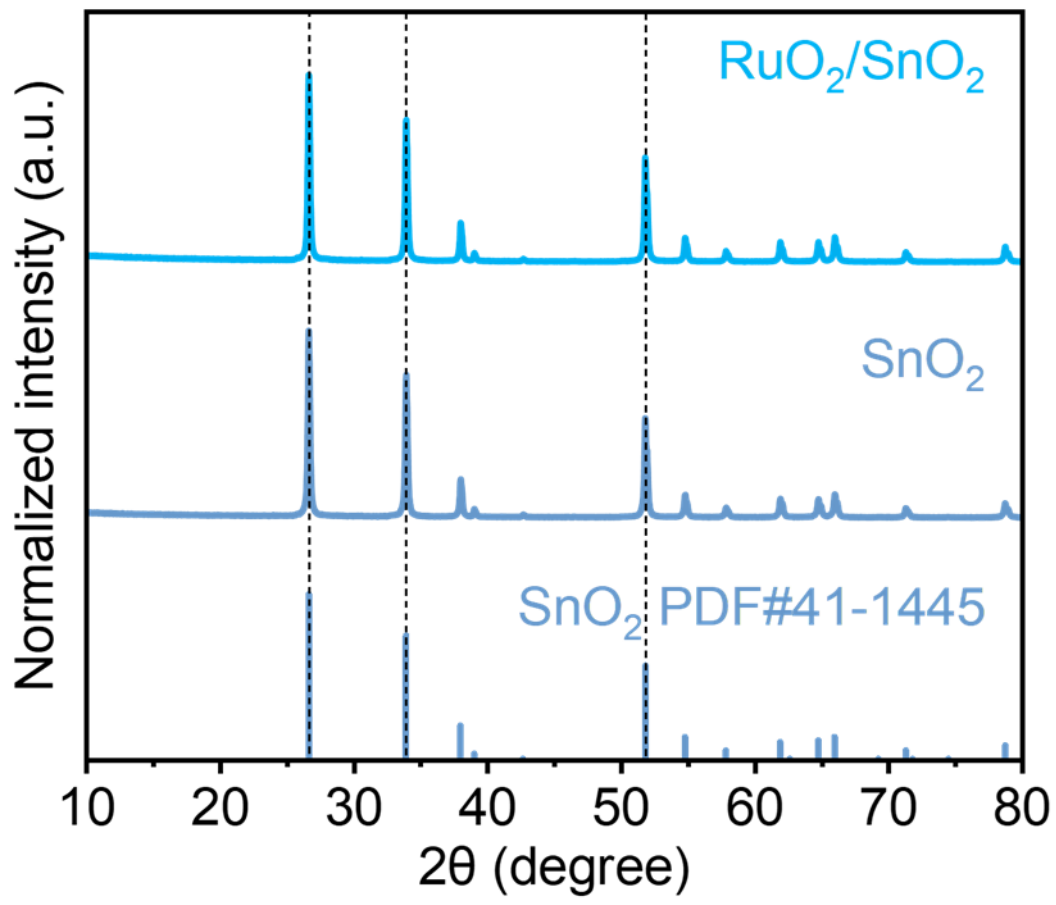

Supplementary Fig. 50 XRD characterization of RuO_2/SnO_2. The XRD patterns of RuO_2/SnO_2 and SnO_2.

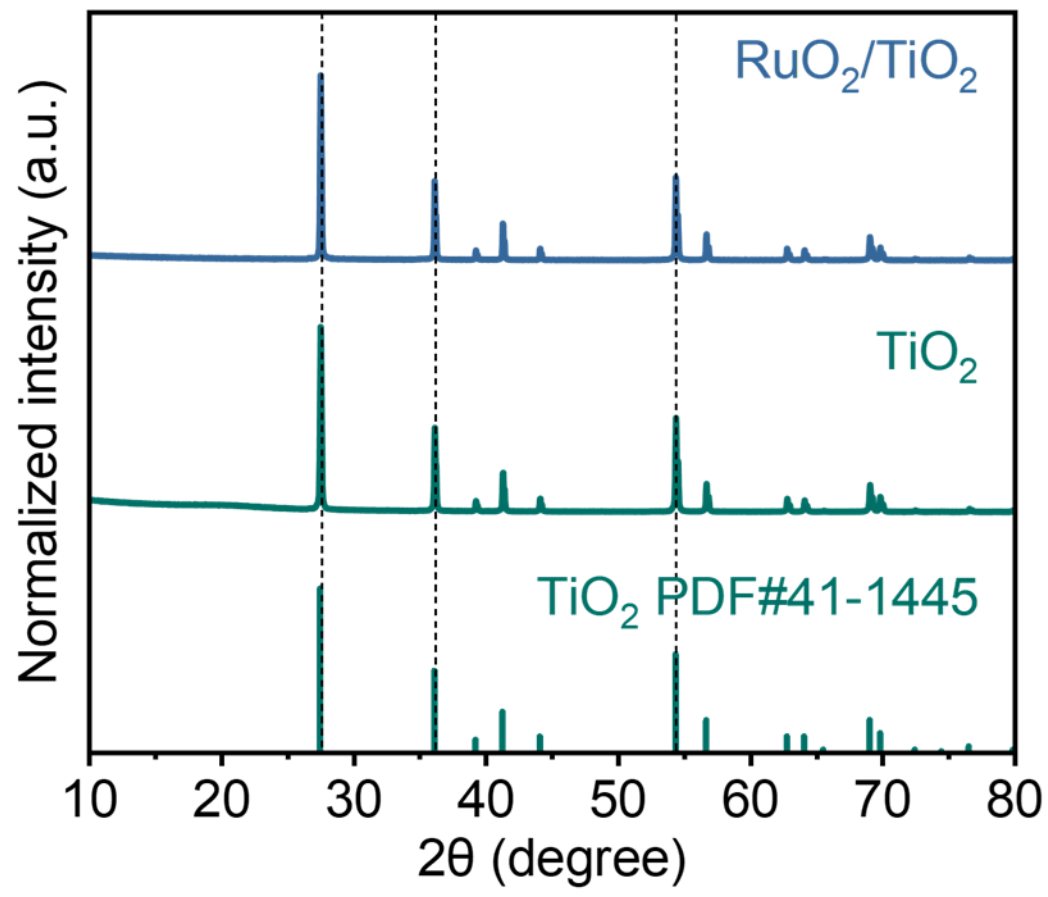

Supplementary Fig. 51 XRD characterization of RuO_2/TiO_2. The XRD patterns of RuO_2/TiO_2 and TiO_2.

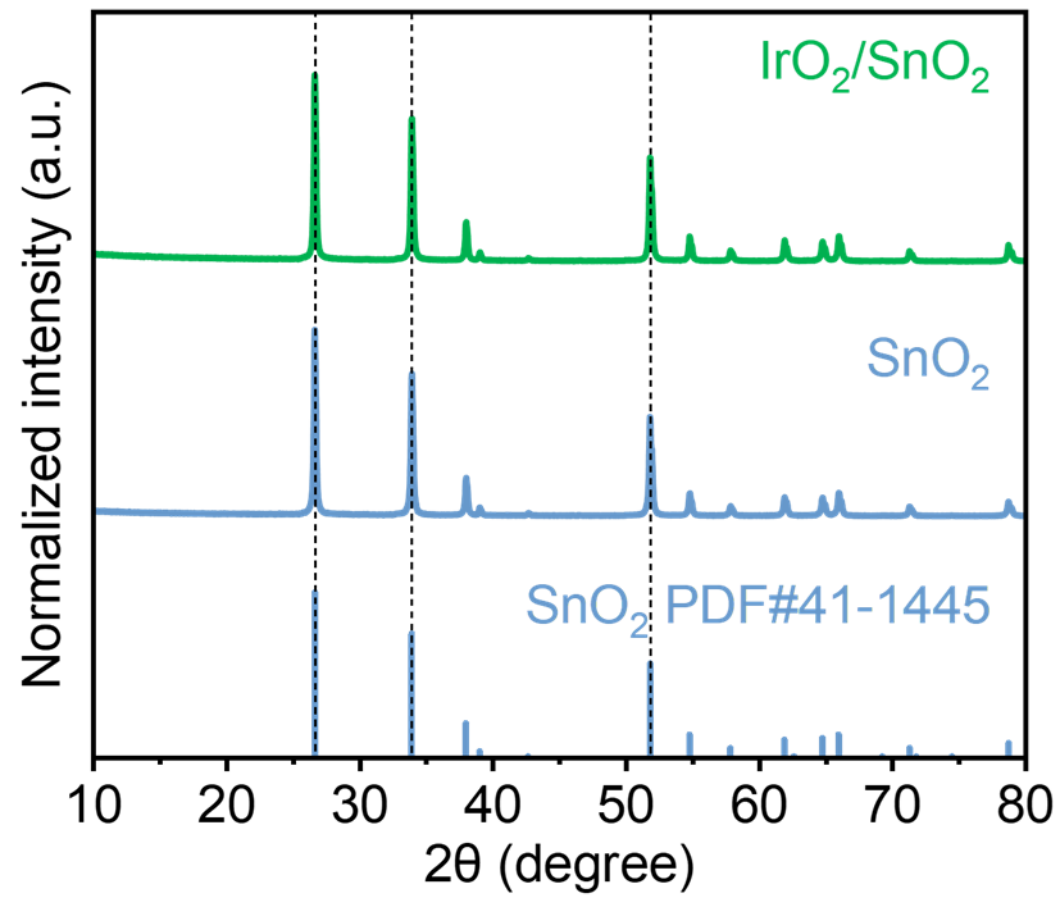

Supplementary Fig. 52 XRD characterization of IrO_2/SnO_2. The XRD patterns of IrO_2/SnO_2 and SnO_2.

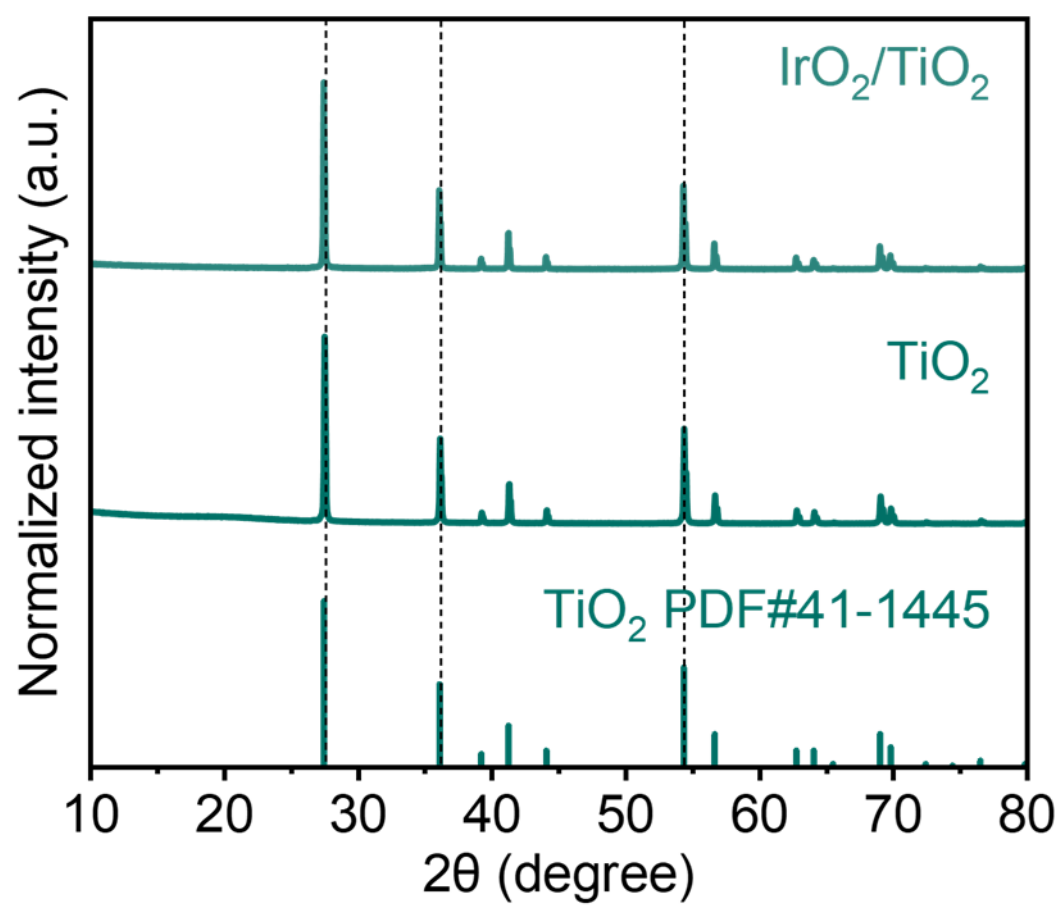

Supplementary Fig. 53 XRD characterization of IrO_2/TiO_2. The XRD patterns of IrO_2/TiO_2 and TiO_2.

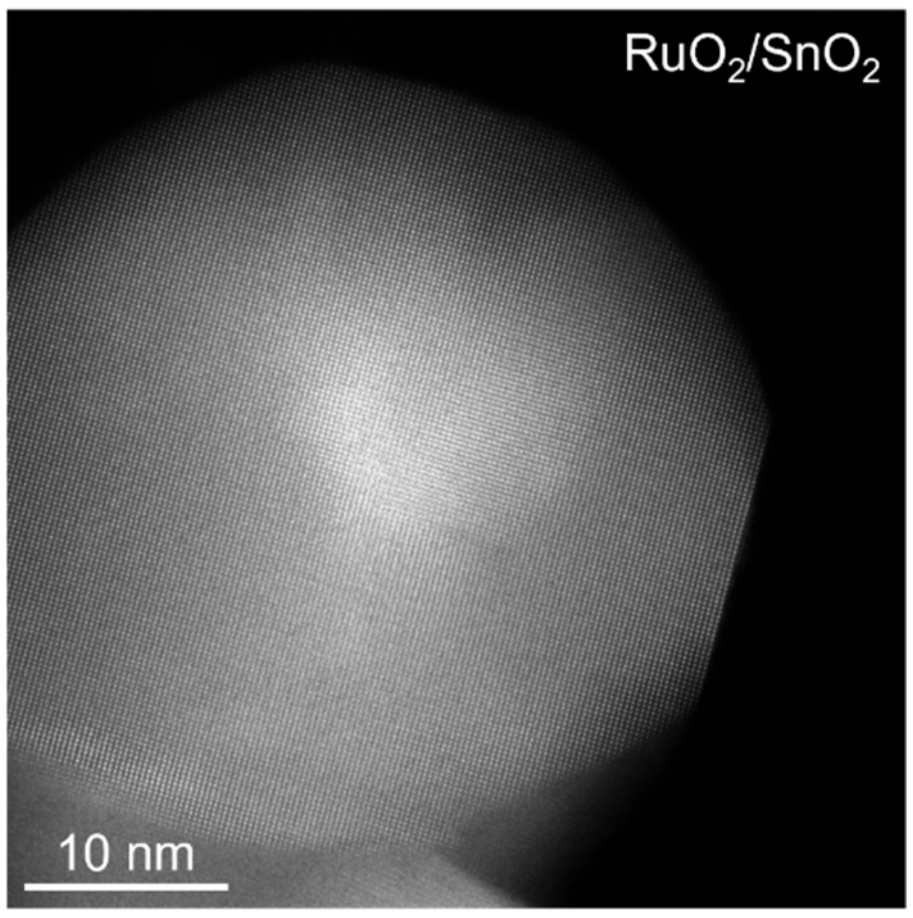

Supplementary Fig. 54 HAADF-STEM image of RuO_2/SnO_2. The representative low-magnification HAADF-STEM image of RuO_2/SnO_2 catalyst.

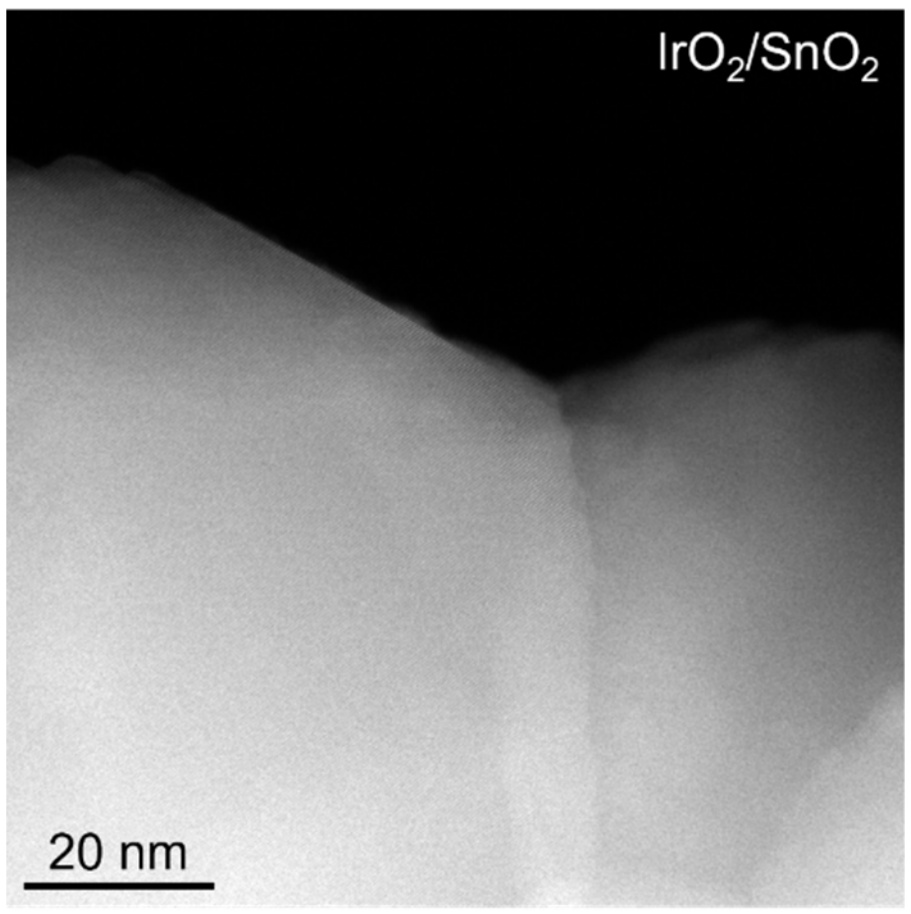

Supplementary Fig. 55 HAADF-STEM image of IrO_2/SnO_2. The representative low-magnification HAADF-STEM image of IrO_2/SnO_2 catalyst.

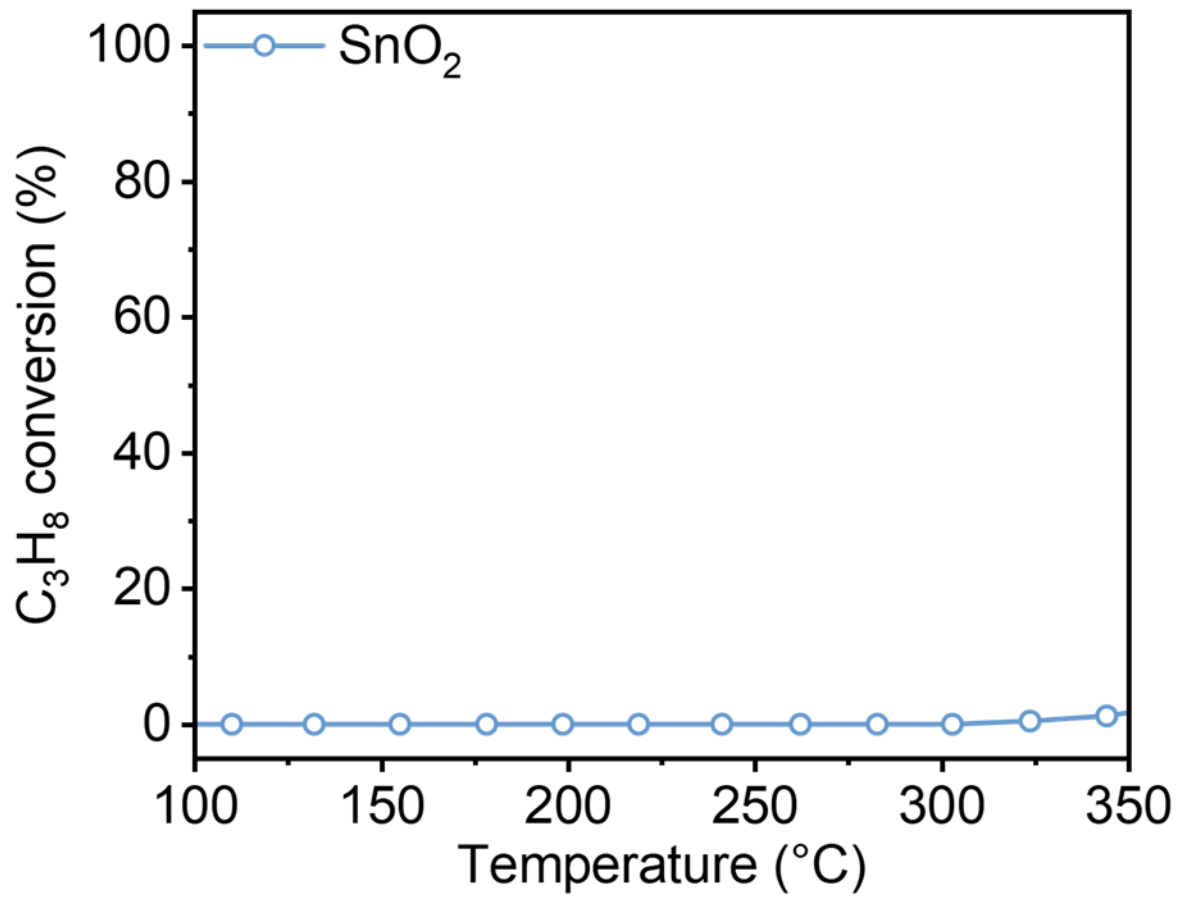

Supplementary Fig. 56 C_3H_8 oxidation over SnO_2. The light-off curve of C_3H_8 oxidation over SnO_2 support.

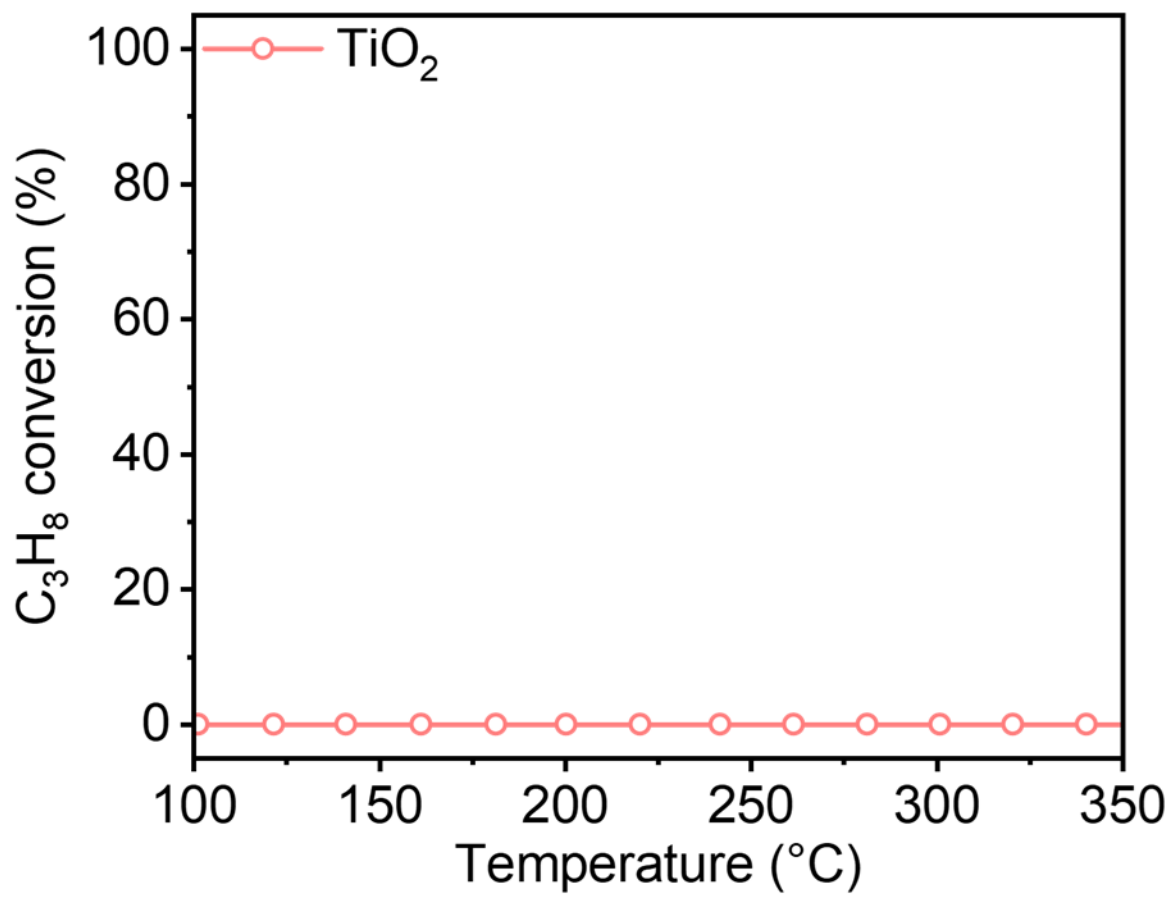

Supplementary Fig. 57 C_3H_8 oxidation over TiO_2. The light-off curve of C_3H_8 oxidation over TiO_2 support.

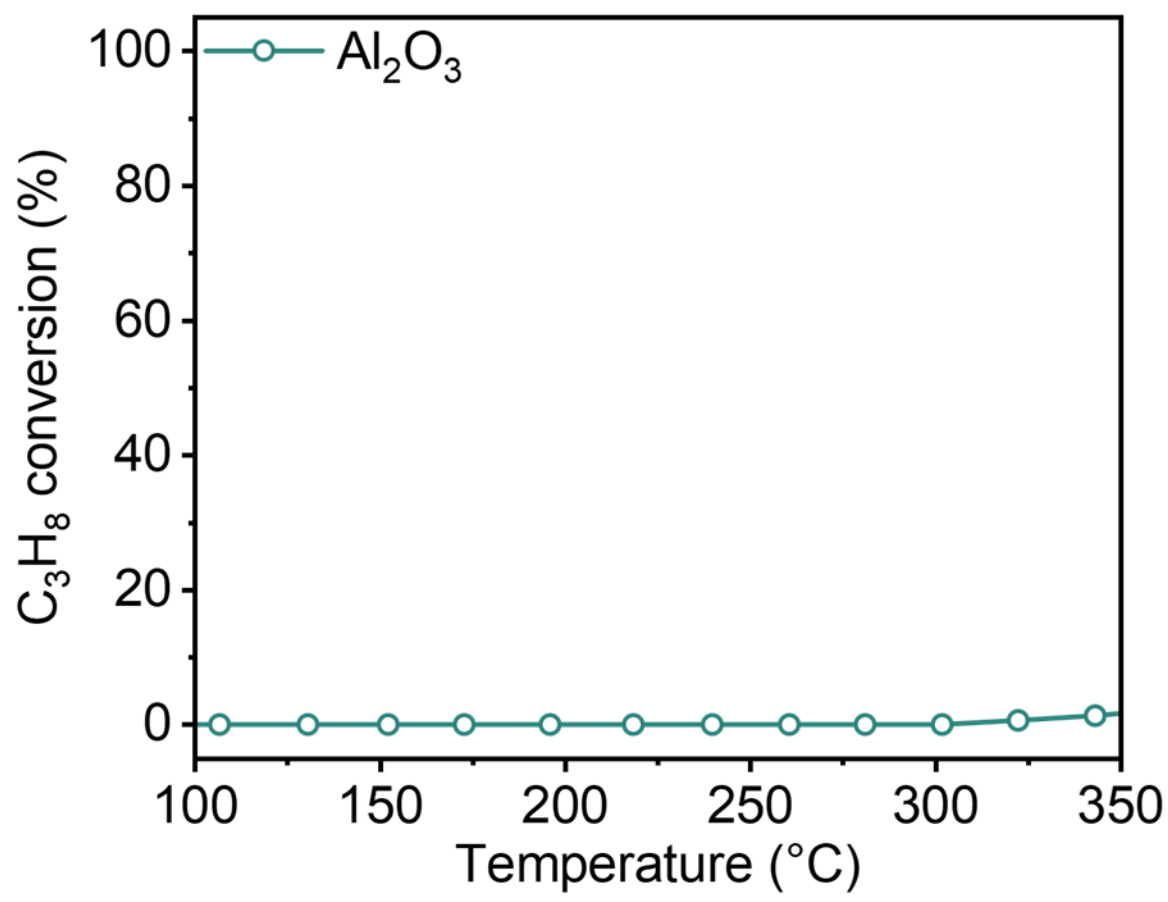

Supplementary Fig. 58 C_3H_8 oxidation over Al_2O_3. The light-off curve of C_3H_8 oxidation over Al_2O_3 support.

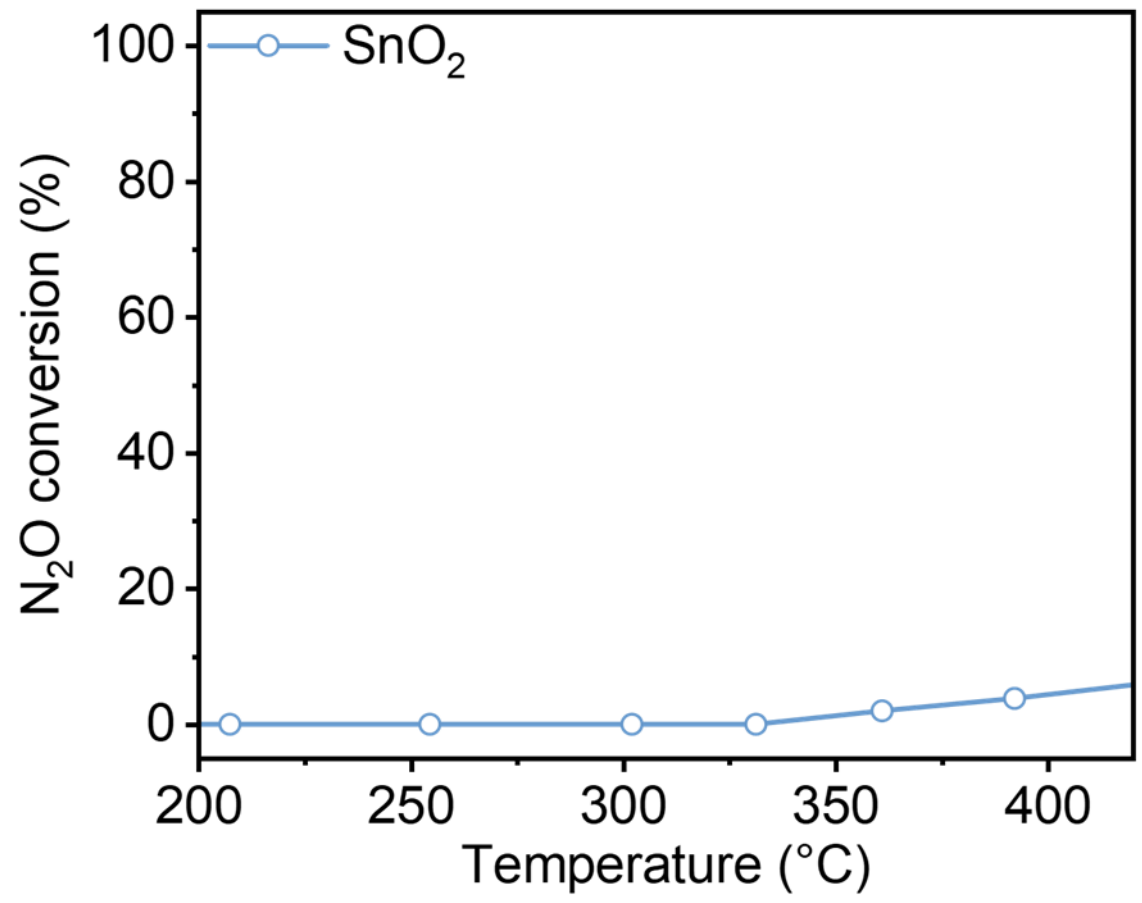

Supplementary Fig. 59 N_2O conversion over SnO_2. The light-off curves of N_2O conversion over SnO_2 support.

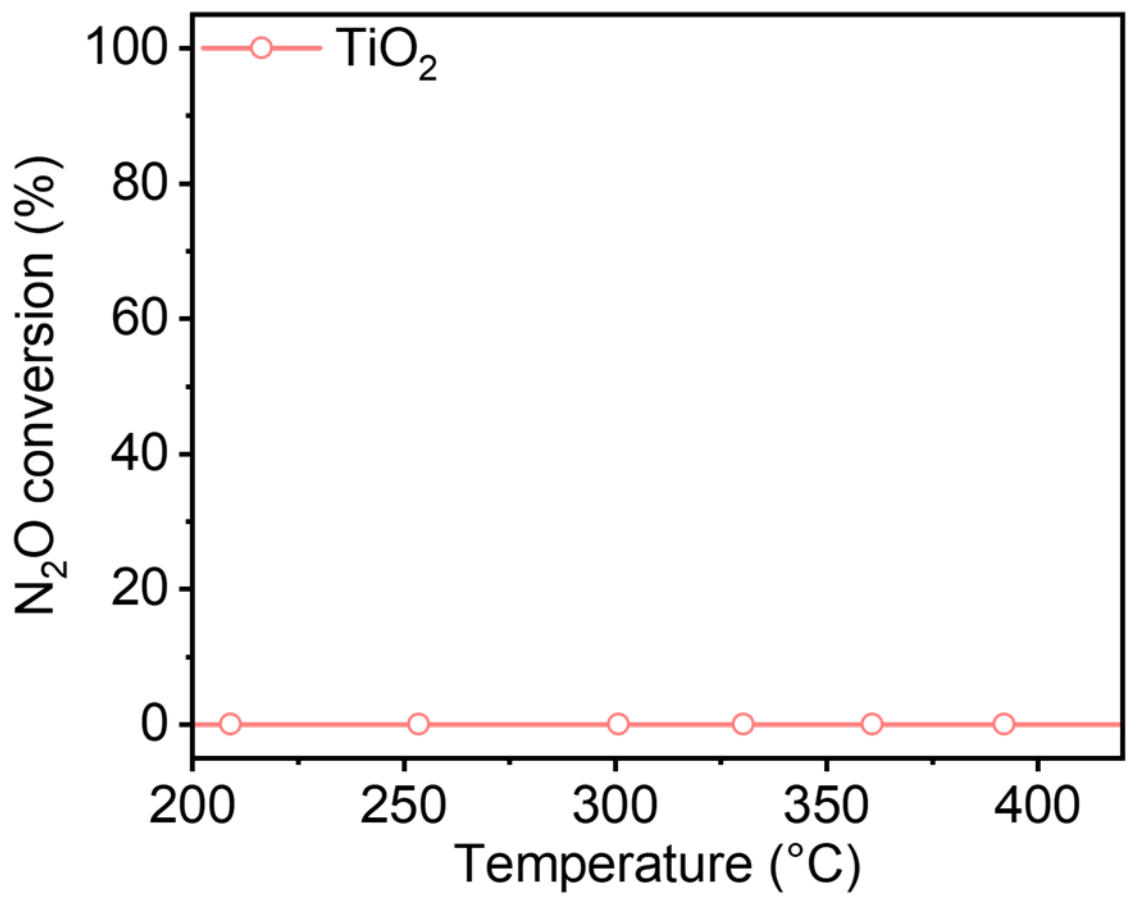

Supplementary Fig. 60 N_2O conversion over TiO_2. The light-off curves of N_2O conversion over TiO_2 support.

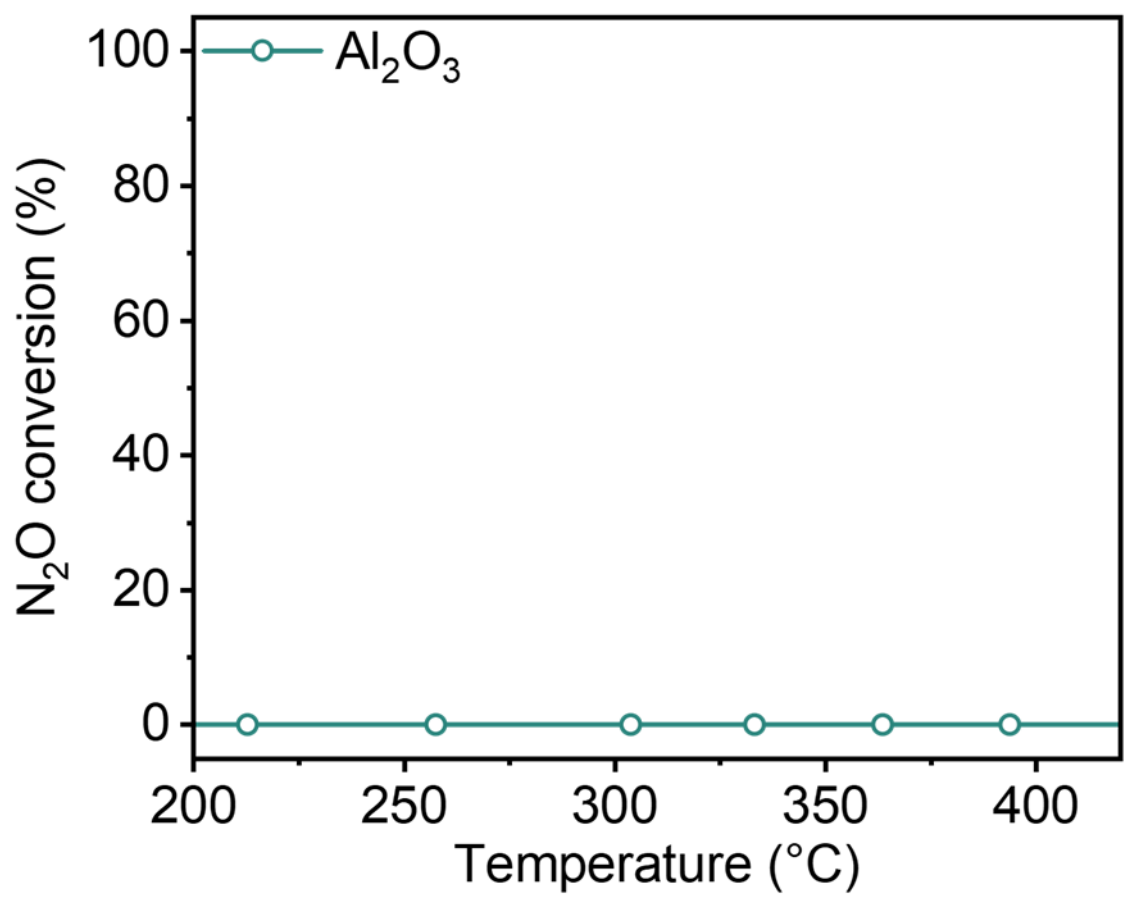

Supplementary Fig. 61 N_2O conversion over Al_2O_3. The light-off curves of N_2O conversion over Al_2O_3 support.

Supplementary Table 1 Chemical compositions of series catalysts.

Catalyst	Actual Pt loading (wt%) [a]
PtO_2/SnO_2	0.73
PtO_2/TiO_2	0.71
PtO_x/Al_2O_3	0.73

a: Determined by ICP-OES

Supplementary Table 2 Lattice parameters of the rutile oxides.

Sample	PDF-*	Crystal plane spacing (Å)				
		(110)	(101)	(200)	(111)	(211)
PtO_2	#37-1087	3.18	2.57	-	2.23	1.69
RuO_2	#40-1290	3.18	2.56	2.25	2.22	1.69
IrO_2	#43-1019	3.18	2.58	2.25	2.24	1.70
TiO_2	#21-1276	3.24	2.49	2.30	2.19	1.69
SnO_2	#41-1445	3.35	2.64	2.37	2.31	1.76

* ICDD/JCPDS PDF retrievals.

Supplementary Table 3 XPS results of PtO_2/SnO_2 and PtO_2/TiO_2 catalysts.

Sample	Relative proportion (%)	
	Pt^{4+}	Pt^{2+}
PtO_2/SnO_2	50.4	49.6
PtO_2/SnO_2-used	53.5	46.5
PtO_2/TiO_2	68.3	31.7
PtO_2/TiO_2-used	68.2	31.8

Supplementary Table 4 Curve fitting parameters for Pt L_3-edge EXAFS of PtO_2/SnO_2 and PtO_2/TiO_2.

Sample	Path	CNs	*R* (Å)	σ^2 (Å^2)	ΔE_0 (eV)	R-factor
PtO_2/SnO_2	Pt–O	4.1±0.3	1.99±0.01	0.002±0.001	8.8±0.7	0.006
	Pt–(O)–Pt	2.8±1.2	3.12±0.02	0.004±0.003		
PtO_2/TiO_2	Pt–O	4.8±1.1	2.00±0.02	0.001[a]	12.1±2.5	0.045
	Pt–(O)–Pt	3.3±4.9	3.04±0.06	0.002[a]		

CN, coordination number; *R*, the distance between the absorber and backscatter atoms; σ^2, Debye-Waller factor; ΔE_0, inner potential correction; R-factor indicates the goodness of the fit. The obtained S_0^2 of Pt foil was 0.90, and S_0^2 was fixed in the subsequent fitting of Pt L_3-edge for the samples. EXAFS curves-fitting ranges: PtO_2/SnO_2, 3.0 Å^{-1} ≤ k ≤ 13 Å^{-1}, 1.15 Å ≤ R ≤ 2.8 Å; PtO_2/TiO_2, 3.0 Å^{-1} ≤ k ≤12.5 Å^{-1}, 1.2 Å ≤ R ≤ 2.7 Å. [a] σ^2 was constrained to positive values during fitting.

Supplementary Table 5 Comparison of catalytic performance in lean-burn CH_4 oxidation for reported Pt-based catalysts.

Sample	Pt loading (wt%)	Feed gas composition	WHSV (mL g^{-1} h^{-1})	T_{90} (°C)	Reaction rate (μmol g_{Pt}^{-1} s^{-1})	Pt dispersion (%)	TOF (s^{-1})	Ref.
**PtO_2/SnO_2**	**0.73**	**1% CH_4, 20% O_2, N_2**	**30,000**	**370**	**15.4 (260 °C)**<br>**87.1 (300 °C)**	**100**	**3.00×10^{-3} (260 °C)**<br>**1.70×10^{-2} (300 °C)**	**This work**
Pt/ZrO_2	1.0	0.2% CH_4, 10% O_2, He	30,000	425	3.2 (300 °C)	-	-	1
SO_2 treated Pt/CeO_2	4	0.05% CH_4, 8% O_2, Ar	15,000 h^{-1}	>500	4.6 (300 °C)	-	-	2
Pt/δ-Al_2O_3 (N0)	1.5	1% CH_4, 4% O_2, He	-	485	12.2 (300 °C)	95	2.00×10^{-3} (300 °C)	3
Pt/Alumina	1.5	0.3% CH_4, 16% O_2, He	78,000	-	15.4 (300 °C)	81	3.70×10^{-3} (300 °C)	4
Pt_1/CF-W	1.0	0.5% CH_4, 20% O_2, N_2	20,000	386	83.6 (300 °C)	100	1.63×10^{-2} (300 °C)	5
Pt/γ-Al_2O_3	1	0.2% CH_4, 10% O_2, N_2	30,000	510	0.1 (310 °C)	37	5.83×10^{-5} (310 °C)	6
Pt/Cr_2O_3	1	0.2% CH_4, 10% O_2, N_2	30,000	390	6.8 (310 °C)	93	1.55×10^{-3} (310 °C)	6
Pt/rutile-400	0.56	1% CH_4, 20% O_2, He	36,000	~390	35.9 (310 °C)	22.5	3.11×10^{-2} (310 °C)	7
0.5H_2O_2-Pt_1/R-Mn_2O_3-5D	0.82	1% CH_4, 18% O_2, He	20,000	605	24.1 (400 °C)	100	4.70×10^{-3} (400 °C)	8
Pt@CeO_2 (13.8 nm)	1.0	1% CH_4, 2% O_2, N_2	555,555	600	686.9 (450 °C)	-	-	9
Pt/Al_2O_3	1.8	0.2% CH_4, 10% O_2, N_2	400,000	>625	20.5 (525 °C)	-	-	10
1Pt/Fe_2O_3-C700	1.9	0.5% CH_4, 3% O_2, He	90,000	>700	558 (700 °C)	100	0.1 (700 °C)	11
Pt/r-TiO_2	2.01	1% CH_4, air	36,000	382	-	76.87	-	12
Pt/SnO_2	1.0	0.2% CH_4, 10% O_2, N_2	60,000	391	-	-	-	13
Pt/CeO_2/3DOM LF	1.0	1% CH_4, 20% O_2, Ar	45,000	502	-	-	-	14

Supplementary Table 6 Chemical compositions of Pd-based catalysts.

Catalyst	Actual Pd loading (wt%) [a]
Pd/SnO_2	0.88
Pd/Al_2O_3	1.0

a: Determined by ICP-OES

Supplementary Table 7 Curve fitting parameters for Pt L_3-edge EXAFS of 8.1 wt% PtO_2/SnO_2.

Sample	Path	CNs	*R* (Å)	σ^2 (Å^2)	$\triangle E_0$ (eV)	R-factor
8.1 wt% PtO_2/SnO_2	Pt–O	4.4±0.3	2.01±0.01	0.002±0.001	10.0±0.8	0.012
	Pt–(O)–Pt	6.4±3.5	3.13±0.02	0.012±0.005		
	Pt–(O)–M[a]	n.q.[a]	3.84±0.04	n.q.[a]		

CN, coordination number; *R*, the distance between the absorber and backscatter atoms; σ^2, Debye-Waller factor; $\triangle E_0$, inner potential correction; R-factor indicates the goodness of the fit. The obtained S_0^2 of Pt foil was 0.90, and S_0^2 was fixed in the subsequent fitting of Pt L_3-edge for the samples. EXAFS curve-fitting ranges: 3.0 Å^{-1} ≤ k ≤13 Å^{-1}, 1.2 Å ≤ R ≤ 3.5 Å. [a] M represents unresolved Pt or Sn scattering. According to the model-derived distances, the long Pt–(O)–Pt-related path overlaps with the interfacial Pt–(O)–Sn-related path; therefore, this high-R contribution was treated as an unresolved Pt–(O)–M shell. Its CN and σ^2 were not quantitatively interpreted due to the large fitting uncertainty. n.q., not quantified.

Supplementary Table 8 Representative model-derived path distances from the PtO_2/SnO_2 CIF structure.

Path	*R* (Å)
Pt–O	2.08
Pt–(O)–Pt short	3.19
Pt–(O)–Pt long	3.71
Pt–(O)–Sn	3.76

The listed distances are derived from the epitaxial PtO_2/SnO_2 model CIF structure and are used only for path assignment. They are not EXAFS-fitted distances.

Supplementary References

1. Torralba, R. *et al.* Total oxidation of methane over sulfur poisoning resistant Pt/ZrO_2 catalyst: Effect of Pt^{2+}–Pt^{4+} and Pt^{2+}–Zr^{4+} dipoles at metal-support interface. *Catal. Lett.* **151**, 1592–1603 (2021).
2. Kylhammar, L., Carlsson, P.-A. & Skoglundh, M. Sulfur promoted low-temperature oxidation of methane over ceria supported platinum catalysts. *J. Catal.* **284**, 50–59 (2011).
3. Marceau, E., Lauron-Pernot, H. & Che, M. Influence of the metallic precursor and of the catalytic reaction on the activity and evolution of Pt(Cl)/δ-Al_2O_3 catalysts in the total oxidation of methane. *J. Catal.* **197**, 394–405 (2001).
4. Gracia, F. J., Miller, J. T., Kropf, A. J. & Wolf, E. E. Kinetics, FTIR, and controlled atmosphere EXAFS study of the effect of chlorine on Pt-supported catalysts during oxidation reactions. *J. Catal.* **209**, 341–354 (2002).
5. Yang, J. *et al.* Modulating the strong metal-support interaction of single-atom catalysts via vicinal structure decoration. *Nat. Commun.* **13**, 4244 (2022).
6. Corro, G., Torralba, R., Pal, U., Olivares-Xometl, O. & Fierro, J. L. G. Total oxidation of methane over Pt/Cr_2O_3 catalyst at low temperature: Effect of Pt^0–Pt^{x+} dipoles at the metal–support interface. *J. Phys. Chem. C* **123**, 2882–2893 (2019).
7. Zhang, X. *et al.* Crystal-phase-mediated restructuring of Pt on TiO_2 with tunable reactivity: Redispersion versus reshaping. *ACS Catal.* **12**, 3634–3643 (2022).
8. Yan, D., Chen, J. & Jia, H. Temperature-induced structure reconstruction to prepare a thermally stable single-atom platinum catalyst. *Angew. Chem. Int. Ed.* **59**, 13562–13567 (2020).
9. Lee, S., Seo, J. & Jung, W. Sintering-resistant Pt@CeO_2 nanoparticles for high-temperature oxidation catalysis. *Nanoscale* **8**, 10219–10228 (2016).
10. Wilburn, M. S. & Epling, W. S. Sulfur deactivation and regeneration of mono- and bimetallic Pd-Pt methane oxidation catalysts. *Appl. Catal., B* **206**, 589–598 (2017).
11. Lang, R. *et al.* Non defect-stabilized thermally stable single-atom catalyst. *Nat. Commun.* **10**, 234 (2019).
12. Hu, B. *et al.* Crystalline phase-modulated PtO_x nanoparticles exhibit superior methane combustion performance and sulfur poisoning resistance: Multi-interaction regulation. *J. Environ. Chem. Eng.* **12**, 113140 (2024).
13. Wang, R. *et al.* Electron withdrawal from methane by Pt atoms on stannic oxide for highly active low-temperature combustion. *Environ. Sci. Technol.* **59**, 12121–12131 (2025).
14. Xu, H. *et al.* Enhanced thermal stability of lean methane combustion by structural interactions of CeO_2 with Pt/3DOM $LaFeO_3$ catalysts. *Fuel* **364**, 131069 (2024).
