# Supplementary material for: Epitaxial-strain engineering of supported tensile noble metallene oxides for enhanced oxidation catalysis
Source: Nat Commun. 2026 Oct 13;17:11579. doi: 10.1038/s41467-026-78119-2 (PMC13752311; doi:10.1038/s41467-026-78119-2)
Supplement: Supplementary file 2 [file 41467_2026_78119_MOESM2_ESM.pdf]

nature portfolio

Peer Review File

Epitaxial-strain engineering of supported tensile noble metallene oxides for enhanced oxidation catalysis

Corresponding Author: Professor Yun Guo

This file contains all reviewer reports in order by version, followed by all author rebuttals in order by version.

Version 0:

Reviewer comments:

Reviewer #1

(Remarks to the Author)
This manuscript reports an epitaxial strain strategy to stabilize and modulate the electronic structure of supported PtO_2 metallene oxide. By employing rutile TiO_2 and rutile SnO_2 supports with distinct lattice parameters, the authors claimed the epitaxial growth of PtO_2 metallene oxide layers with controlled strain states, where the larger lattice SnO_2 substrate imposes biaxial tensile strain on PtO_2. The tensile-strained PtO_2/SnO_2 exhibits a reconfigured Pt–O electronic environment that simultaneously improves structural resilience and enhances the intrinsic reactivity of PtO_2, delivering markedly improved methane oxidation performance.

The authors adopted the common wet impregnation method and achieved the metallene oxides, which is not well characterized using the present approaches from the reviewer's opinion. It is difficult to determine the existence of the new morphology of the metal oxide claimed. Furthermore, the present materials are not well active for methane oxidation. The real challenge of the methane oxidation is the low-temperature oxidation at the temperature of < 300 celsius. The reviewer therefore can not give the positive comments.

From the HRSTEM image of the present manuscript, it is hard to determine the atoms are the loaded Pt atoms or the outmost surface atoms of the support. Since the atom weight between Pt and Sn atoms, the Pt atoms should be more bright than Sn under the STEM model. The presence of PtO_2 species is mainly depicted by modelling and no experimental evidence is provided for the presence of PtO_2, because the Pt atom can coordinate with oxygen atoms derived from the oxides. Unless, a non-oxide support was used to confirm the presence of PtO_2.

The authors stated that the atoms on the surface of the support is PtO_2 metallene oxide, if that is correct, how did the authors define this single layer atoms? what is the difference between the metallene oxide and singel atoms, or atomically dispersed metals? If that is actually the single atom catalysts?

Reviewer #2

(Remarks to the Author)
The manuscript describes Pt catalysts where the active structures involve epitaxial thin films of Pt oxide on oxide supports. The authors aim to show that the reactivity for several oxidation reactions (per gm Pt) is enhanced through the epitaxial structures due to tensile strain in the Pt oxide. At the low metal loadings, it is difficult to establish the level of strain via electron microscopy and diffraction, which are the primary techniques used by the authors. A further complication is that the Pt structures are not stable, and easily transformed by the probes (electron microscope, or CO DRIFTS) that are used to study the Pt. Nonetheless, the authors have used a broad range of characterization techniques to investigate the nature of the Pt and the results are interesting. I do have some concerns about the characterization results that aim to validate the central hypothesis:
1) The title of the paper is 'Epitaxial strain engineering" To demonstrate that there is strain, the authors should present characterization of the bulk PtO_2, the PtO_2/TiO_2 (with less strain) the Pt/Al_2O_3 reference and compare with PtO_2/SnO_2 which has the most strain. The only technique that can yield precise information on the bond distances is EXAFS. The authors present data on the SnO_2 based catalysts; similar data is lacking for the other two catalysts. The FT of the XAFS for PtO_2 is shown in Supp figure 11, where the Pt-O-Pt peak seems to be at the same position for both the PtO_2/SnO_2 and the PtO_2. I would like to see the fits for all of these samples, to validate the claim of strain engineering. Otherwise, it appears to

be a case where the SnO_2 support enhances the reactivity of the metal oxide.
2) While Supplementary Table 6 presents a comparison of the performance of the SnO_2 based catalysts with the literature, the unit is moles reacted per gram Pt per second. Very few of the catalysts in the literature are atomically dispersed, so the value of reactivity needs to be divided by the fractional dispersion to get a fair comparison. Or the dispersion of the Pt should be included in this table.
3) There is no in-situ data validating the stability of the SnO_2 based catalysts. After methane oxidation, the sample is cooled and ex-situ analysis is performed. It is well known that dispersed Pt will get oxidized upon air exposure. Hence, the XPS results have to be considered in light of the tendency of Pt to get oxidized upon air exposure.
4) Figure 1b is meant to show that there are no XRD reflections attributed to Pt. But the figure is misleading since the peaks are not labelled. They should be clearly labelled as coming from the SnO_2 or TiO_2 support.
5) When showing images such as Figure 1c, the authors need to be aware that the identity of the surface layer (labelled PtO_2) cannot be conclusively identified from the contrast, or the lattice spacing. The authors need to show EDS analysis of each of these images so the reader can see the Pt loading and compare it with the average loading, as inferred from the lower magnification images shown in the supplementary materials.
6) Figure 2g shows the XANES for the PtO_2/SiO_2 compared with bulk PtO_2. The preparation of this PtO_2 reference needs to be described and its XRD pattern included in the SM. The reactivity of this reference PtO_2 needs to be compared (per surface Pt atom) with the catalysts described here (see comment 1).
7) Figure 2d shows that the PtO_2 on TiO_2 is reduced at a much lower temperature than PtO_2/SnO_2. This is consistent with the postulate that the SnO_2 based catalyst is strained and hence more difficult to reduce. As pointed out by the authors (line 262), PtO_2 will generally convert into metallic Pt as it is heated, so retaining the oxide is key to the performance of these catalysts. The evidence presented here in terms of the multiple cycles of methane oxidation and longer-term stability as well as post-reaction characterization, suggests that this PtO_2/SnO_2 is indeed stable under the conditions. For practical applications involving these catalysts, it would be good to know at what temperature this strained PtO_2 ultimately starts to convert to particulate form.
8) Since the PtO_2/SnO_2 can be easily reduced in H_2 (Fig. 2d), will it convert back to the strained oxide structure. In other words, is the catalyst after TPR transformed irreversibly into a particulate Pt structure?
9) The reactivity for methane oxidation on the PtO_2/SnO_2 is clearly superior to the reference PtO_x/Al_2O_3 (Figure 3a). But additional explanation is needed for Figure 3b where a plateau in reactivity is reached at a loading ~1.95 wt%. The authors argue that after the theoretical monolayer loading is exceeded, the specific activity drops (Figure 3b). The authors need to provide more details for the derivation of this theoretical maximum. The equation presented on line 167 of the SM leaves many open questions. The BET surface area is not stated anywhere, and neither is sigma Sn or Sigma Pt. The authors need to state how this areal density of Sn or Pt atoms is determined. And once this areal density (mol/m^2) is known, why can't they simply multiply by the BET surface area (m^2/g) and molecular weight to get the maximum loading (gm Pt/gcatalyst)?
10) While images of well-defined facets of SnO_2 make a convincing case for the presence of 2-D Pt on the surface, the low magnification images show typical spheroidal particles of SnO_2. This means these particles expose a variety of surface facets. Hence, when considering the areal density of Sn or Pt (see comment 9) they need to consider an average of the most commonly exposed surface facets of SnO_2.
11) Supplementary figure 28 shows XRD data for a range of Pt loadings on SnO_2. What was the treatment temperature for these samples? I have the same comment for all of the figures, where the sample treatments should be specified in the figure caption.
12) And, as I stated in my comment (2) above, an overall EDS needs to accompany all these figures, like Figure 29, so the reader can see the actual loading of Pt on the sample.
13) Figure 29 is very interesting in that even at a high loading of Pt (8.1 wt%), only 2-D structures are seen. The authors should present EXAFS of this catalyst, because it may reveal the presence of Pt-O-Sn bonds which may be responsible for the enhanced reactivity and stability of these catalysts.
14) The authors suggest that prolonged examination in the microscope causes the 2-D structure to transform into clusters. For the reader to see this clearly, the contrast in the SM Figure 4b needs to be adjusted (make it brighter) so the reader can clearly see the structures that are formed. And more importantly, it is not clear why there is all the Pt in the region away from the SnO_2 structure (Fig. 4c). The same comment (about contrast) applies to other images where beam damaged regions are shown (as in Figure 23a) because the structures that are formed after beam damage are not visible.
15) When showing epitaxial structures where the surface is at an angle different from 90 degrees (as in Figure 5c and other images in the SM) to the SnO_2 lattice planes, the authors need to show the interatomic spacing along the surface, not the way it is shown in Figure 5c.
In summary, this work shows a catalyst system where deposition of Pt on SnO_2 creates 2-D structures that show improved reactivity for several oxidation reactions. The stability of these structures makes this work of interest to the catalysis of oxidation reactions.

Reviewer #3

(Remarks to the Author)
This work contains new results and could be considered for publication. However, the authors should revise their manuscript before acceptance for publication in accordance with the following comments.
1. To better understand the electronic structures and the chemical active Pt species in PtO_2/SnO_2 and PtO_2/TiO_2, the binding energies of the elements must be indicated.
2. The authors report for PtO_2/SnO_2 smaller amounts of Pt^{4+} (49.6%), relative to PtO_2/TiO_2 (68.3%), and explain this result, suggesting a strain-induced redistribution of electron density at the Pt centers. However, as SnO_2 and TiO_2 present semiconducting properties, the effect of energy alignment between PtO_2-SnO_2 and PtO_2-TiO_2 must be deeply considered

and related to their physical and chemical properties. The same must be applied for RuO_2 and IrO_2 based catalysts.

3. In Figure 3 (b), the estimated values of the reaction rate of methane oxidation are reported as μmol gPt^{-1} s^{-1}. However, these values do not represent the real catalytic behavior of PtO_2. Instead reaction rate must be estimated as μmol(surface Pt site)$^{-1}$$s^{-1}$.

Version 1:

Reviewer comments:

Reviewer #1

(Remarks to the Author)
The authors tried to address the questions raised by the reviewer. I do not have further comments about the revision version of this manuscript. Thanks for providing me the opportunity to read this work. It can be accepted considering the improved quality of the work.

Reviewer #2

(Remarks to the Author)
The authors have done considerable additional work and addressed all of the comments. The work is now suitable for publication.

Reviewer #3

(Remarks to the Author)
I have carefully examined the authors' responses to my previous comments and the revised version of the manuscript.
The authors have satisfactorily addressed my major concerns. In particular:
1. Regarding the different Pt4+ fractions observed for PtO2/SnO2 and PtO2/TiO2, the authors have now provided additional experimental evidence by comparing the band gaps of the two supports. The results indicate no significant difference in their electronic structures from this perspective, supporting their interpretation. Similar considerations have also been appropriately extended to the RuO2 and IrO2 based catalysts.
2. The authors have also adequately addressed my concern regarding the normalization of the methane oxidation rates. Their explanation and the corresponding revisions clarify the catalytic activity assessment and satisfactorily resolve this issue.
Overall, I consider that the authors have responded appropriately to my comments, and I believe that the revised manuscript is now suitable for publication.

Open Access This Peer Review File is licensed under a Creative Commons Attribution 4.0 International License, which permits use, sharing, adaptation, distribution and reproduction in any medium or format, as long as you give appropriate credit to the original author(s) and the source, provide a link to the Creative Commons license, and indicate if changes were made.
In cases where reviewers are anonymous, credit should be given to 'Anonymous Referee' and the source.

The images or other third party material in this Peer Review File are included in the article's Creative Commons license, unless indicated otherwise in a credit line to the material. If material is not included in the article's Creative Commons license and your intended use is not permitted by statutory regulation or exceeds the permitted use, you will need to obtain permission directly from the copyright holder.
To view a copy of this license, visit https://creativecommons.org/licenses/by/4.0/

Response to reviewers' comments (NCOMMS-26-009959-T)

REVIEWER COMMENTS

Reviewer #1 (Remarks to the Author):

This manuscript reports an epitaxial strain strategy to stabilize and modulate the electronic structure of supported PtO_2 metallene oxide. By employing rutile TiO_2 and rutile SnO_2 supports with distinct lattice parameters, the authors claimed the epitaxial growth of PtO_2 metallene oxide layers with controlled strain states, where the larger lattice SnO_2 substrate imposes biaxial tensile strain on PtO_2. The tensile-strained PtO_2/SnO_2 exhibits a reconfigured Pt–O electronic environment that simultaneously improves structural resilience and enhances the intrinsic reactivity of PtO_2, delivering markedly improved methane oxidation performance.

Q1: The authors adopted the common wet impregnation method and achieved the metallene oxides, which is not well characterized using the present approaches from the reviewer's opinion. It is difficult to determine the existence of the new morphology of the metal oxide claimed. Furthermore, the present materials are not well active for methane oxidation. The real challenge of the methane oxidation is the low-temperature oxidation at the temperature of < 300 celsius. The reviewer therefore can not give the positive comments.

Response:

We sincerely thank the reviewer for this critical comment. We fully understand the reviewer's concern that the existence of an atomically thin Pt oxide morphology must be established with great care, especially because conventional wet impregnation would normally be expected to generate dispersed Pt species or Pt-containing nanoparticles rather than an ordered two-dimensional oxide overlayer.

In the revised manuscript, we have therefore strengthened both the structural evidence and the discussion of the formation mechanism. We clarify that the **key point of this synthesis is not the wet impregnation procedure itself, but the rutile-rutile epitaxial relationship between PtO_2 and the oxide supports under oxidative calcination conditions.** The lattice matching between rutile PtO_2 and rutile SnO_2 or TiO_2 lowers the interfacial energy and promotes the formation of an atomically thin epitaxial Pt oxide overlayer rather than three-dimensional Pt oxide particles (*Chem. Commun.* 2026, 62, 9620–9636). Importantly, under identical preparation conditions, Pt deposited on the non-rutile Al_2O_3 support forms PtO_x clusters rather than an oxide overlayer, confirming that rutile lattice matching is essential for PtO_2-MO formation. This point is discussed in detail in our response to Q3.

To further support this assignment, we have added and expanded several lines of evidence in the revised manuscript and Supplementary Information. First, we performed HAADF-STEM image simulations with different PtO_2 coverages to explain the experimentally observed contrast of the Pt-containing surface layer. Second, we added a pure SnO_2 beam-irradiation control experiment, showing that the SnO_2 surface remains stable under the same imaging conditions, whereas the topmost layer in PtO_2/SnO_2 transforms into Pt-containing nanoparticles upon prolonged irradiation, as confirmed by EDS mapping. These added analyses directly address the reviewer's concern regarding the identity of the topmost surface layer and are discussed in detail in our response to Q2.

Regarding methane oxidation activity, however, we would like to emphasize that the focus of this work is not solely to maximize methane oxidation activity. Rather, the central objective of this work is to address a different but important challenge: stabilizing PtO_2-MO, a normally metastable and reducible Pt oxide phase, under reaction conditions. **Conventional Pt-based catalysts generally suffer from the instability of Pt oxide species at elevated temperatures, which limits their performance and durability in lean-burn methane oxidation.** In this context, the tensile-strained PtO_2/SnO_2 catalyst

shows markedly improved activity and durability relative to PtO_2/TiO_2 and conventional Pt-based references. We have also expanded the benchmark comparison with representative Pt-based methane oxidation catalysts in the revised Supplementary Information on Page S69 to provide a more balanced evaluation of its catalytic performance.

We hope that these additional experiments, analyses, and revisions adequately address the reviewer's concerns regarding the structural assignment, formation mechanism, and catalytic relevance of the PtO_2-MO catalysts.

Q2: From the HRSTEM image of the present manuscript, it is hard to determine the atoms are the loaded Pt atoms or the outmost surface atoms of the support. Since the atom weight between Pt and Sn atoms, the Pt atoms should be more bright than Sn under the STEM model.

Response:

We sincerely thank the reviewer for this important comment. We agree that, according to the Z-contrast principle in HAADF-STEM, an isolated Pt atom (Z = 78) would be expected to give a higher intensity than an isolated Sn atom (Z = 50). However, the experimentally observed HAADF-STEM intensity is not determined solely by the atomic number, but also depends strongly on the projected column thickness and the number of atoms in each projected column.

In our case, the SnO_2 particles have a relatively large thickness of approximately 60 nm, meaning that each projected Sn column contains a large number of superimposed Sn atoms from the support. In contrast, the Pt loading of the PtO_2/SnO_2 catalyst is only 0.73 wt%, which is lower than the calculated theoretical maximum dispersion threshold for a complete PtO_2 monolayer on SnO_2 (1.95 wt%). This corresponds to approximately 37% of a monolayer coverage based on this theoretical threshold. Therefore, the number of Pt atoms projected in a surface Pt column is much smaller than the number of Sn atoms

projected in the underlying SnO_2 support column. Under these conditions, the Pt-containing surface columns at the SnO_2 particle edge are not necessarily expected to appear brighter than the much thicker SnO_2 support columns. Meanwhile, in the high-loading 8.1 wt% PtO_2/SnO_2 sample, where the PtO_2 coverage is much higher, the Pt-containing overlayers can be directly resolved with stronger contrast (**Fig. R1**, reproduced from Supplementary Figs. 39b and 39c in the revised Supplementary Information).

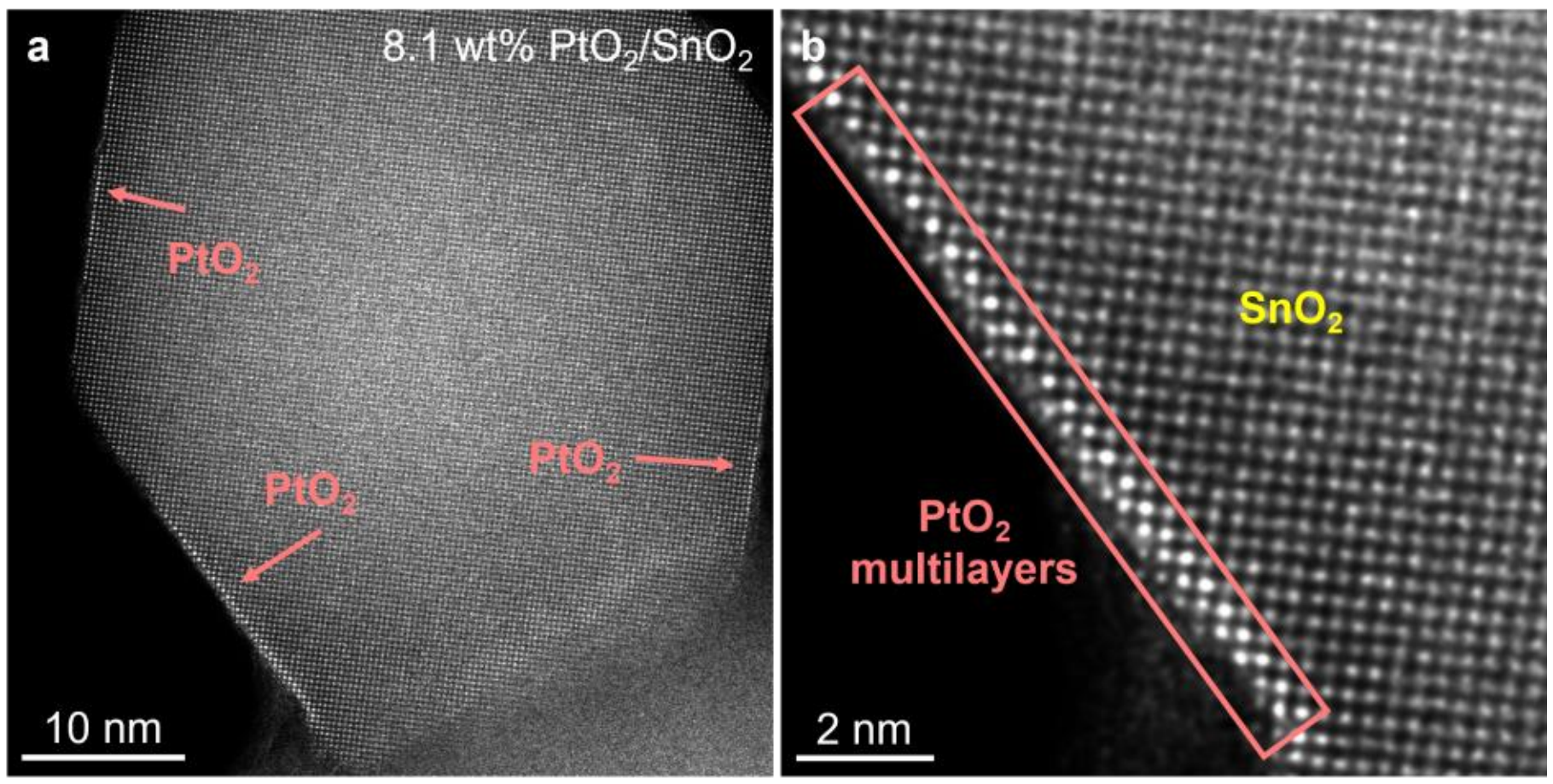

Fig. R1 Representative (a) low-magnification and (b) atomic-scale HAADF-STEM images of 8.1 wt% PtO_2/SnO_2 catalyst.

To further verify this interpretation, we performed HAADF-STEM image simulations using a SnO_2(110) slab model with a thickness of approximately 10 nm, in which PtO_2 overlayers with different edge-exposed coverages, including one monolayer (1 ML), one-half monolayer (1/2 ML), and one-third monolayer (1/3 ML), were constructed (**Figs. R2-R5**). The simulation results show that Pt columns are brighter than Sn columns at full monolayer coverage, as expected from Z-contrast (**Fig. R2**). However, the relative Pt-column intensity decreases markedly as the PtO_2 coverage decreases (**Fig. R3**). **At approximately 1/3 ML coverage, the simulated Pt-containing columns become less intense than the SnO_2 support columns** (**Fig. R4**). The line-intensity analysis also shows that the apparent contrast is strongly affected by projection through the SnO_2 slab. These simulations therefore explain why the surface Pt-containing layer in the low-loading (0.73 wt%) PtO_2/SnO_2 catalyst does not appear brighter than the SnO_2 support in the

experimental HAADF-STEM images (**Figs. R5a-5b**).

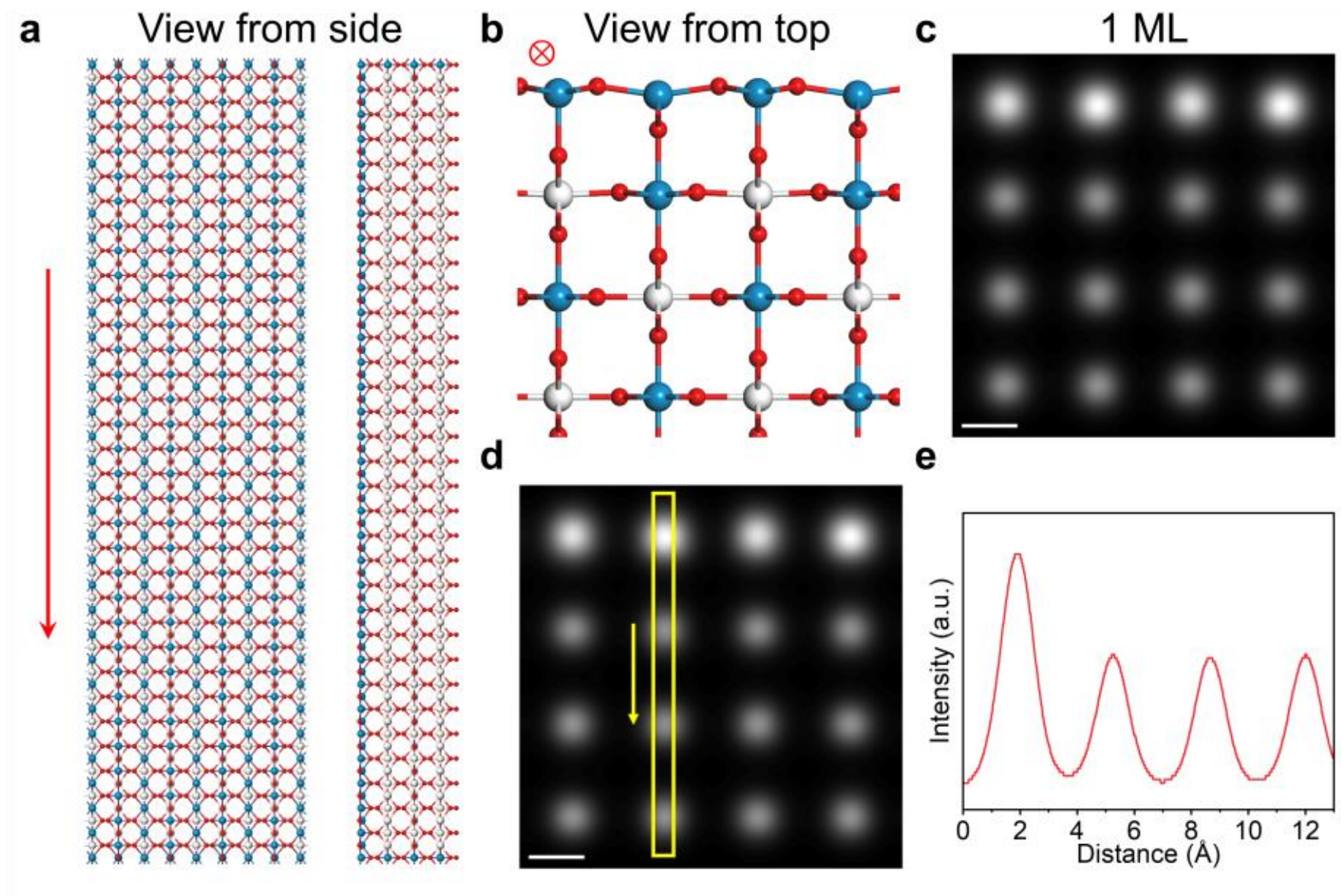

Fig. R2 (a, b) Atomic structural models, (c, d) simulated HAADF-STEM images, and (e) corresponding intensity profile for 1 ML of PtO_2 on the $SnO_2(110)$ surface. The blue, white, and red balls denote Pt, Sn, and O atoms, respectively. The intensity profile was taken from the rectangular box along the yellow arrow in (d). Scale bars, 2 Å in (c) and (d).

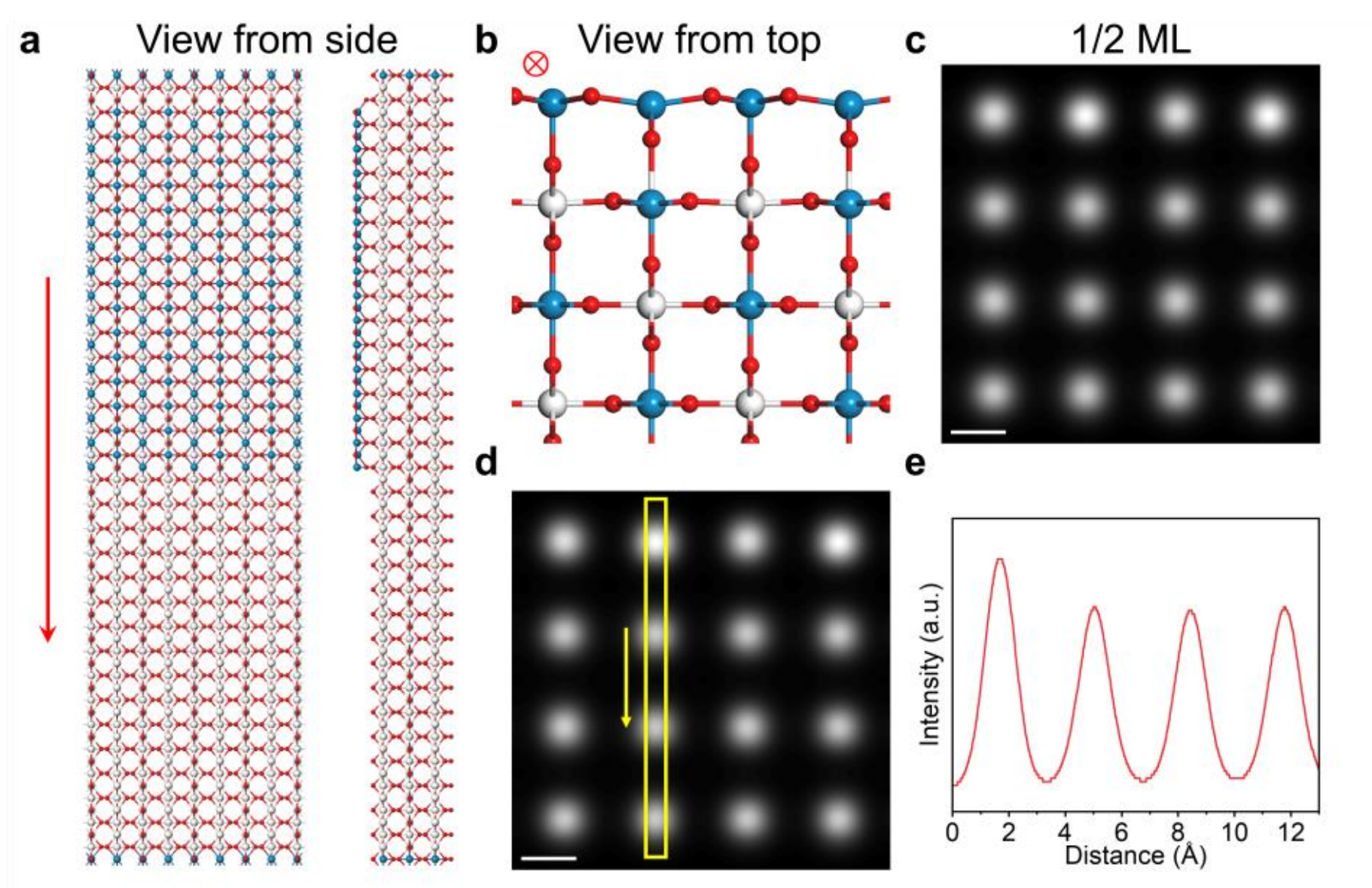

Fig. R3 (a, b) Atomic structural models, (c, d) simulated HAADF-STEM images, and (e) corresponding intensity profile for 1/2 ML of PtO_2 on the $SnO_2(110)$ surface. The blue,

white, and red balls denote Pt, Sn, and O atoms, respectively. The intensity profile was taken from the rectangular box along the yellow arrow in (d). Scale bars: 2 Å in (c) and (d).

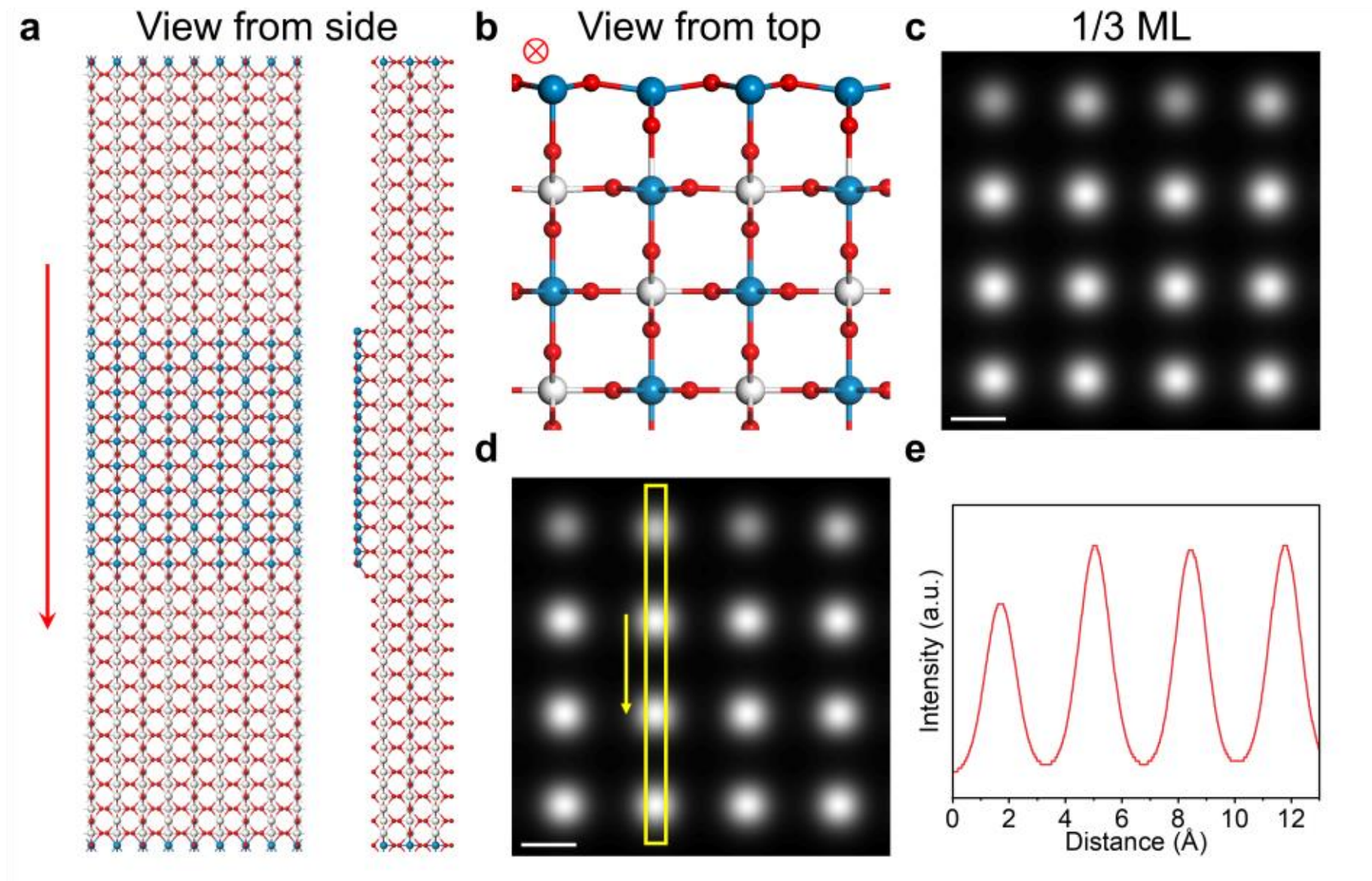

Fig. R4 (a, b) Atomic structural models, (c, d) simulated HAADF-STEM images, and (e) corresponding intensity profile for 1/3 ML of PtO_2 on the $SnO_2(110)$ surface. The blue, white, and red balls denote Pt, Sn, and O atoms, respectively. The intensity profile was taken from the rectangular box along the yellow arrow in (d). Scale bars: 2 Å in (c) and (d).

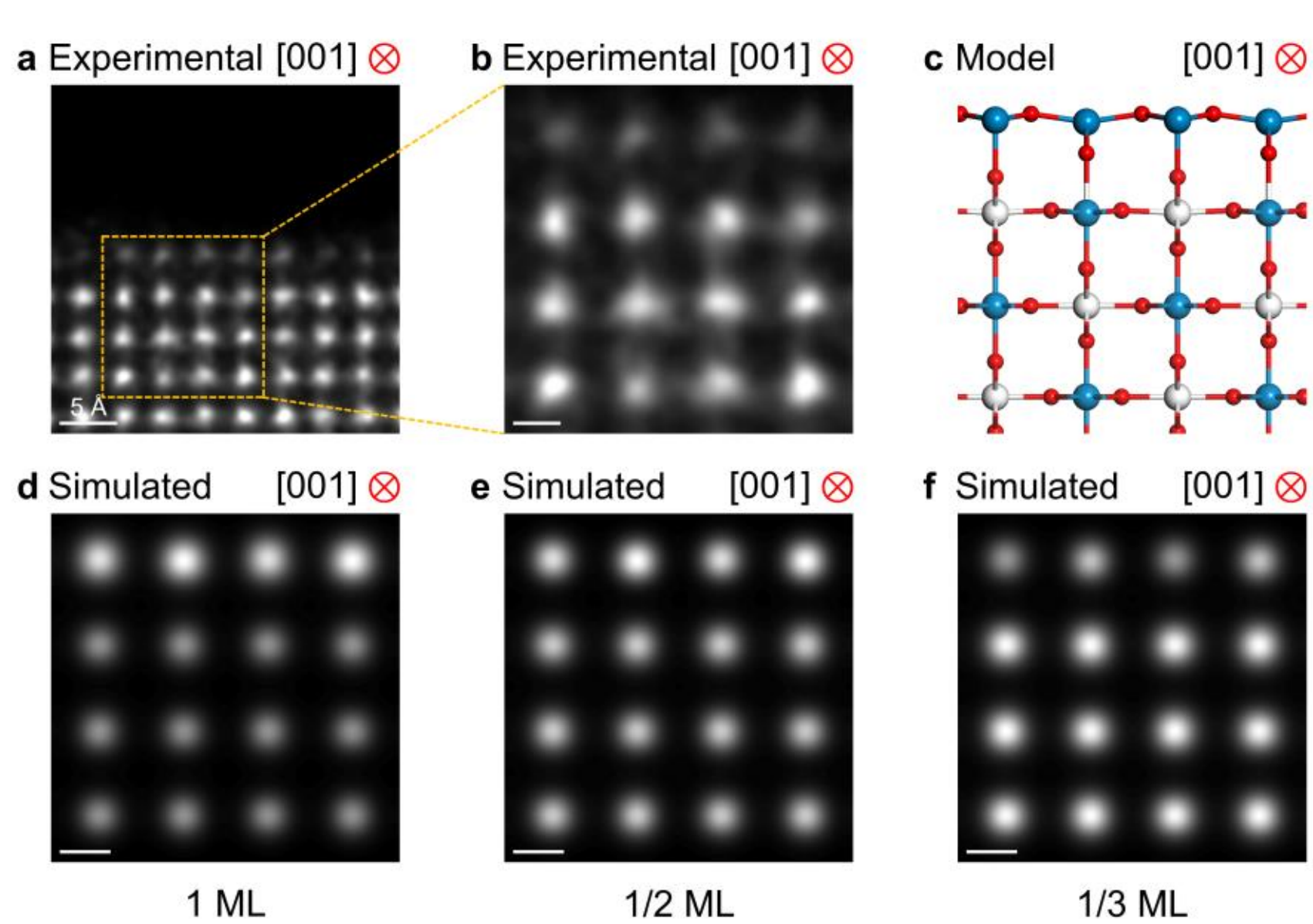

Fig. R5. (a) HAADF-STEM image taken from Fig. 1d. (b) Magnified HAADF-STEM image of the golden boxed region in (a). (c) Atomic structural model of PtO_2 on the $SnO_2(110)$ surface. (d–f) Simulated HAADF-STEM images for PtO_2 coverages of 1 ML, 1/2 ML, and 1/3 ML on the $SnO_2(110)$ surface, respectively. Scale bars, 2 Å in (b) and (d-f).

In addition to the HAADF-STEM simulations, we further distinguished the supported Pt oxide layer from the outermost SnO_2 surface atoms by comparing their response to prolonged electron-beam irradiation. As shown in **Fig. R6** (reproduced from Supplementary Fig. 5 in the revised Supplementary Information), prolonged irradiation of PtO_2/SnO_2 induces the transformation of the outermost surface layer into nanoparticle-like species. Importantly, subsequent EDS elemental mapping confirms that these beam-induced nanoparticles are Pt-containing species (**Figs. R6 c-d**), providing direct evidence that the beam-sensitive surface layer originates from the loaded Pt species rather than from the SnO_2 support.

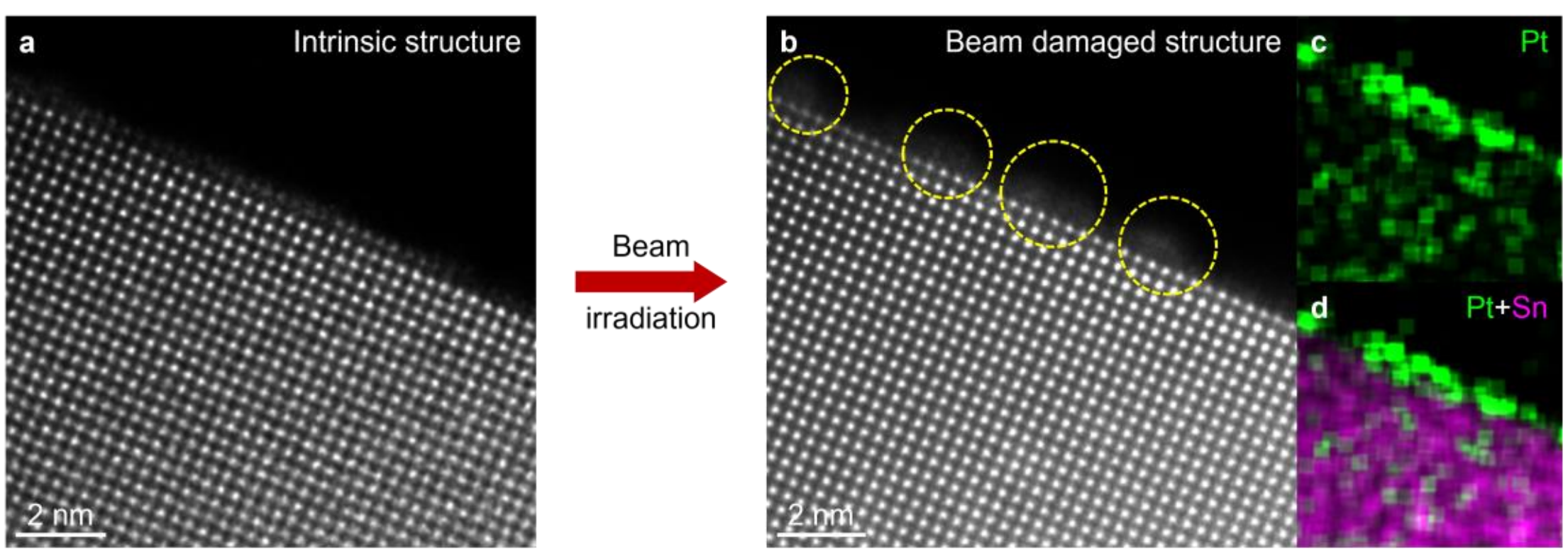

Fig. R6 (a) The HAADF-STEM image of PtO_2/SnO_2 was acquired by the low-damage HAADF-STEM imaging method under the beam current of ~ 50 pA. (b) The HAADF-STEM image was acquired by observing the same area in (a) continuously under HAADF-STEM mode at the beam current of ~ 50 pA for a few seconds. (c-d) The corresponding EDS elemental mapping images of (c) Pt and (d) Pt+Sn compositions. The yellow circle

regions in HAADF-STEM image identify the beam-damaged structure of Pt (small Pt nanoparticles).

To further exclude the possibility that the observed transformation arises from the intrinsic instability of the SnO_2 surface, we have now added a control experiment on pure SnO_2 under the same imaging conditions. As shown in **Fig. R7**, pure SnO_2 remains structurally stable upon prolonged electron-beam irradiation, with no obvious surface reconstruction or nanoparticle formation. This contrast demonstrates that the nanoparticle-like species observed after beam exposure in PtO_2/SnO_2 do not originate from the SnO_2 surface itself, but from the beam-induced restructuring of the supported Pt oxide layer. Together with the EDS identification of the beam-induced particles, these results provide strong evidence that the topmost beam-sensitive layer in PtO_2/SnO_2 corresponds to loaded Pt oxide species rather than the outermost SnO_2 support atoms.

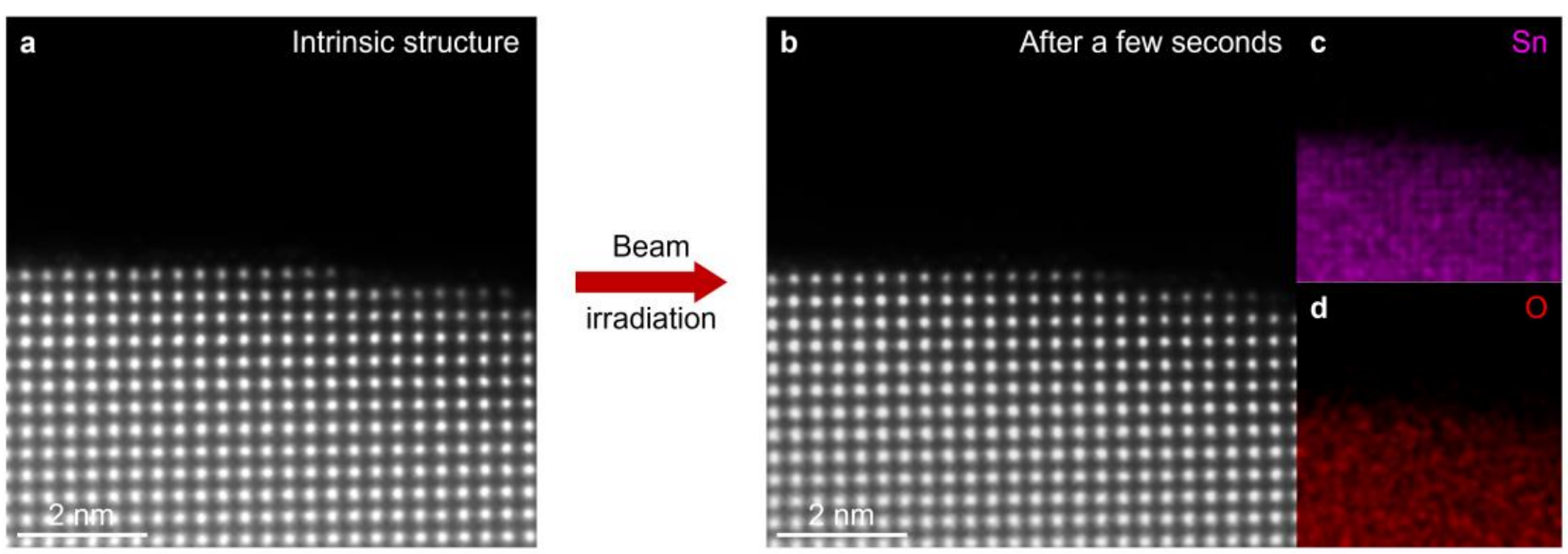

Fig. R7 (a) The HAADF-STEM image of pure SnO_2 support was acquired by the low-damage HAADF-STEM imaging method under the beam current of ~ 50 pA. (b) The HAADF-STEM image was acquired by observing the same area in (a) continuously under HAADF-STEM mode at the beam current of ~ 50 pA for a few seconds. (c-d) The corresponding EDS elemental mapping images of (c) Sn and (d) O compositions.

Figs. R2-R5 have been added to the revised Supplementary Information as Supplementary Figs. 7-10, respectively, and Fig. R7 has been added as Supplementary Fig. 4.

Q3: The presence of PtO_2 species is mainly depicted by modelling and no experimental evidence is provided for the presence of PtO_2, because the Pt atom can coordinate with oxygen atoms derived from the oxides. Unless, a non-oxide support was used to confirm the presence of PtO_2.

Response:

We sincerely appreciate the reviewer's comment. We agree that Pt–O coordination alone does not necessarily prove the formation of a PtO_2-MO, because isolated or atomically dispersed Pt species may also coordinate with oxygen atoms from an oxide support. However, we would like to emphasize that our assignment of PtO_2-MO is not based on structural modelling alone, but on multiple complementary experimental observations.

First, low-damage HAADF-STEM reveals a laterally connected surface overlayer epitaxially registered with the rutile SnO_2 or TiO_2 support, rather than isolated Pt atoms or randomly distributed clusters. The measured lattice spacings of this surface overlayer are consistent with rutile β-PtO_2 and with the epitaxial relationship expected from lattice matching between rutile PtO_2 and the rutile oxide supports. In addition, as discussed in our response to the reviewer's previous comment in Q2, the beam-irradiation/EDS analysis provides further evidence that the observed topmost layer is associated with the loaded Pt species rather than the outermost SnO_2 support atoms. Similar atomically thin PtO_2 layers on rutile TiO_2 have also been directly resolved in our previous work (*J. Am. Chem. Soc.* 2024, 146, 3764–3772), providing an important structural reference for the present assignment.

Second, spectroscopic characterizations support the presence of high-valent Pt oxide species. XPS shows dominant Pt^{2+}/Pt^{4+} components without detectable metallic Pt^0 signals after oxidative preparation (Fig. 2e in the revised manuscript) and after catalytic reaction

(Supplementary Fig. 33 in the revised Supplementary Information), excluding the formation of metallic Pt nanoparticles under these conditions. Together with the XANES/EXAFS/CO-DRIFTS results showing Pt–O coordination, these observations are consistent with the presence of Pt oxide species on the rutile supports.

Regarding the reviewer's suggestion of using a non-oxide support, we respectfully note that the purpose of this work is to stabilize a rutile-type PtO_2 overlayer through epitaxial lattice matching with rutile oxide supports. **A non-oxide support would not provide the oxygen sublattice or the rutile structural template required for epitaxial PtO_2 formation**, and therefore would not be a relevant control for testing the epitaxial stabilization mechanism proposed here. Nevertheless, we performed a control experiment using **Al_2O_3** as a **non-rutile oxide support** under the same preparation conditions. In contrast to rutile SnO_2 and TiO_2, where epitaxially registered atomically thin Pt oxide overlayers are observed, HAADF-STEM imaging shows that Pt deposited on Al_2O_3 forms PtO_x clusters (**Fig. R8**). This control directly addresses the reviewer's concern: **although Al_2O_3 also provides oxygen coordination sites for Pt, it does not generate PtO_2-MO by the same synthesis method.** Therefore, the formation of PtO_2-MO cannot be attributed simply to Pt coordination with support oxygen atoms; instead, it requires the rutile structural template and lattice matching between PtO_2 and the rutile oxide support.

We have clarified this point in the revised manuscript on Pages 7-8, and added Fig. R8 as Supplementary Fig. 12 in the revised Supplementary Information.

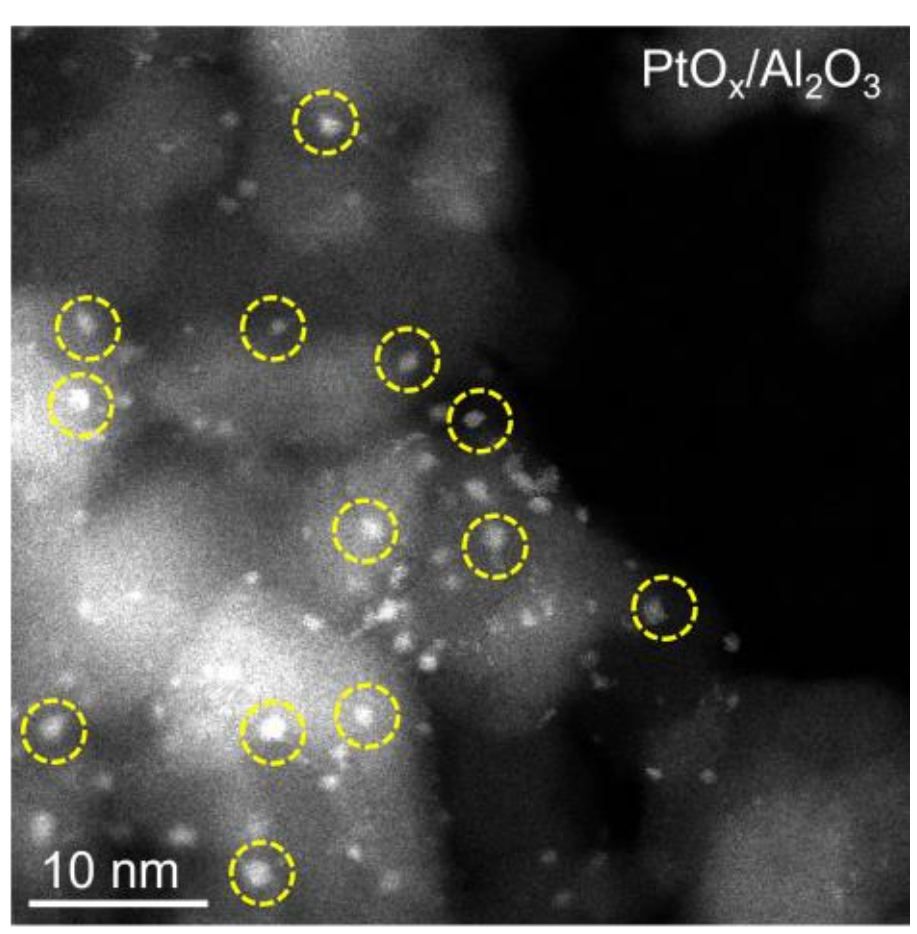

Fig. R8 Representative HAADF-STEM image of PtO_x/Al_2O_3. The yellow circle regions in HAADF-STEM image identify the Pt clusters.

Q4: The authors stated that the atoms on the surface of the support is PtO_2 metallene oxide, if that is correct, how did the authors define this single layer atoms? what is the difference between the metallene oxide and singel atoms, or atomically dispersed metals? If that is actually the single atom catalysts?

Response:

We sincerely thank the reviewer for this important question. We agree that it is necessary to clearly distinguish the PtO_2 metallene oxide from conventional single-atom catalysts.

In this work, the term "single layer" refers to an atomically thin Pt-containing oxide overlayer that contains only one Pt atomic layer in the out-of-plane direction and is epitaxially registered with the rutile oxide support. This is different from isolated Pt atoms dispersed on the support surface. Although Pt is distributed at the atomic scale, the Pt species in PtO_2-MO are not isolated. Instead, **the Pt atoms are laterally connected through oxygen bridges, forming an extended Pt–O–Pt network within a two-dimensional oxide overlayer** (*Acc. Mater. Res.* 2026, doi: 10.1021/accountsmr.6c00009).

This structural connectivity is the key distinction between PtO_2-MO and conventional single-atom catalysts. In a typical Pt single-atom catalyst (*Chem. Rev.* 2018, 118, 4981–5079; *Adv. Mater.* 2025, 37, 2504518), Pt atoms are spatially isolated and stabilized by local coordination sites on the support, without long-range lateral Pt–O–Pt connectivity or epitaxial lattice registry. In contrast, PtO_2-MO shows a laterally connected surface overlayer with lattice spacings consistent with rutile PtO_2 and an epitaxial relationship with the rutile SnO_2 or TiO_2 support. Therefore, although the Pt atoms are highly dispersed at the atomic scale, the structure should not be classified as a conventional single-atom

catalyst.

We realize that our previous wording, “a single atomic layer of PtO_2”, may have caused ambiguity and could be confused with isolated Pt single atoms. We have therefore revised the description on Page 6 of the revised manuscript to clarify that PtO_2-MO is an atomically thin Pt oxide overlayer containing one Pt atomic layer, rather than isolated Pt single atoms.

Reviewer #2 (Remarks to the Author):

The manuscript describes Pt catalysts where the active structures involve epitaxial thin films of Pt oxide on oxide supports. The authors aim to show that the reactivity for several oxidation reactions (per gm Pt) is enhanced through the epitaxial structures due to tensile strain in the Pt oxide. At the low metal loadings, it is difficult to establish the level of strain via electron microscopy and diffraction, which are the primary techniques used by the authors. A further complication is that the Pt structures are not stable, and easily transformed by the probes (electron microscope, or CO DRIFTS) that are used to study the Pt. Nonetheless, the authors have used a broad range of characterization techniques to investigate the nature of the Pt and the results are interesting. I do have some concerns about the characterization results that aim to validate the central hypothesis:

Q1: The title of the paper is 'Epitaxial strain engineering" To demonstrate that there is strain, the authors should present characterization of the bulk PtO_2, the PtO_2/TiO_2 (with less strain) the Pt/Al_2O_3 reference and compare with PtO_2/SnO_2 which has the most strain. The only technique that can yield precise information on the bond distances is EXAFS. The authors present data on the SnO_2 based catalysts; similar data is lacking for the other two catalysts. The FT of the XAFS for PtO_2 is shown in Supp figure 11, where the Pt-O-Pt peak seems to be at the same position for both the PtO_2/SnO_2 and the PtO_2. I would like to see the fits for all of these samples, to validate the claim of strain engineering. Otherwise, it appears to be a case where the SnO_2 support enhances the reactivity of the metal oxide.

Response:

We sincerely thank the reviewer for this important suggestion. Following the reviewer's suggestion, we performed additional Pt L_3-edge XAS measurements and EXAFS fitting for PtO_2/SnO_2 and PtO_2/TiO_2. The corresponding FT-EXAFS spectra and fitting results are shown in **Figs. R9-R11**. As shown in **Fig. R9**, both samples show a

dominant first-shell peak at approximately 1.7 Å before phase correction, corresponding to Pt–O coordination, and a weaker higher-shell feature at approximately 2.8 Å before phase correction, associated with Pt–(O)–Pt scattering. Quantitative EXAFS fitting is required to compare the actual scattering path lengths. The fitting results are shown in **Figs. R10** and **R11** and summarized in **Table R1**. The fitted Pt–O coordination numbers are 4.1 ± 0.3 for PtO_2/SnO_2 and 4.8 ± 1.1 for PtO_2/TiO_2, with similar Pt–O distances of 1.99 ± 0.01 Å and 2.00 ± 0.02 Å, respectively. In contrast, the fitted higher-shell Pt–(O)–Pt distance is longer for PtO_2/SnO_2 than for PtO_2/TiO_2, with values of 3.12 ± 0.02 Å and 3.04 ± 0.06 Å, respectively.

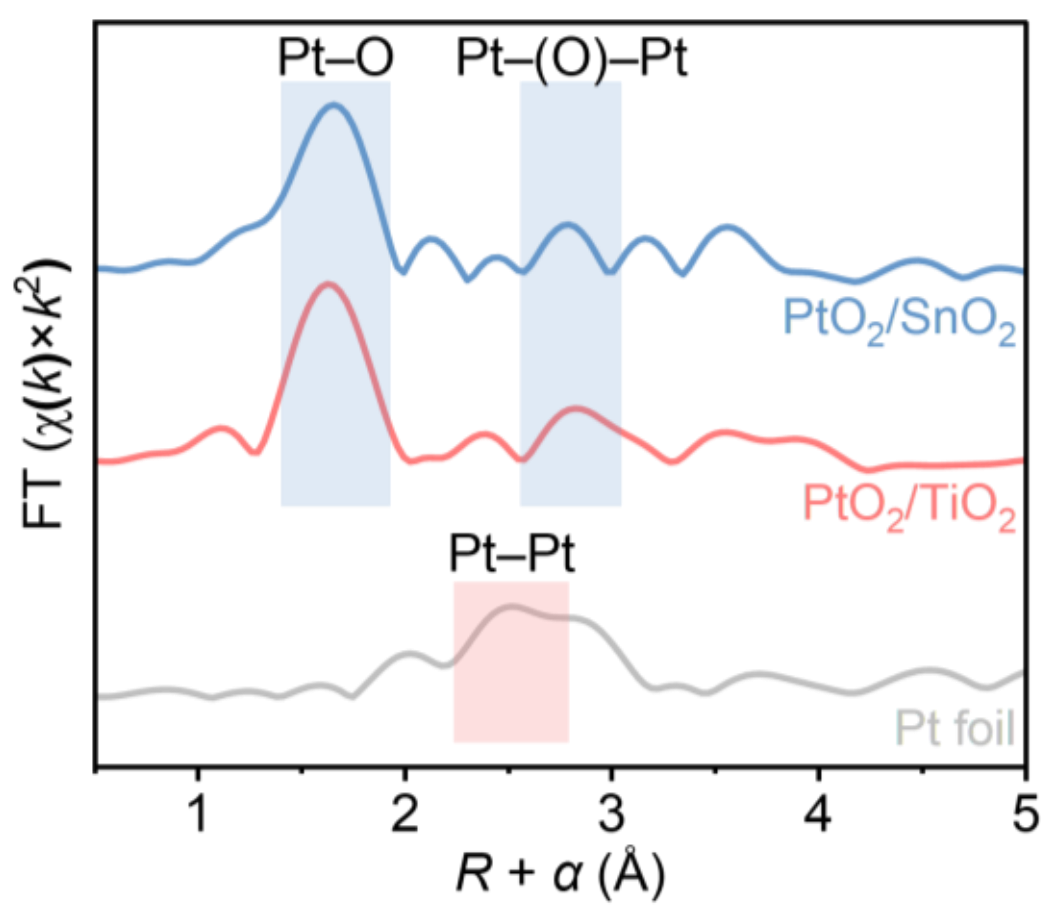

Fig. R9 FT-EXAFS spectra at the Pt L_3-edge of PtO_2/SnO_2, PtO_2/TiO_2 and Pt foil.

These EXAFS results suggest that the support-dependent structural difference is mainly reflected in the higher-shell Pt–(O)–Pt geometry rather than in a large change of the nearest-neighbor Pt–O bond length. This trend is consistent with our epitaxial structural model, in which the larger lattice parameter of SnO_2 imposes tensile distortion on the PtO_2 overlayer, leading to an expanded in-plane Pt–(O)–Pt geometry relative to PtO_2/TiO_2. However, because the higher-shell signal is weak, especially for PtO_2/TiO_2, we do not rely solely on the EXAFS-derived coordination numbers to establish the strain assignment. We would also like to clarify that, for an atomically thin epitaxial oxide overlayer supported on crystalline oxide particles, EXAFS provides ensemble-averaged local scattering information, whereas the epitaxial relationship and in-plane lattice distortion are more

directly evaluated by real-space atomic-resolution STEM analysis (*Nat. Commun.* 2018, 9, 2722; *Nature* 2021, 598, 76–81; *Nat. Commun.* 2022, 13, 5957). Therefore, the strain assignment is based on the combined evidence from low-damage HAADF-STEM imaging, FFT/line-profile analysis, structural modelling, and the EXAFS-derived Pt–(O)–Pt distance trend.

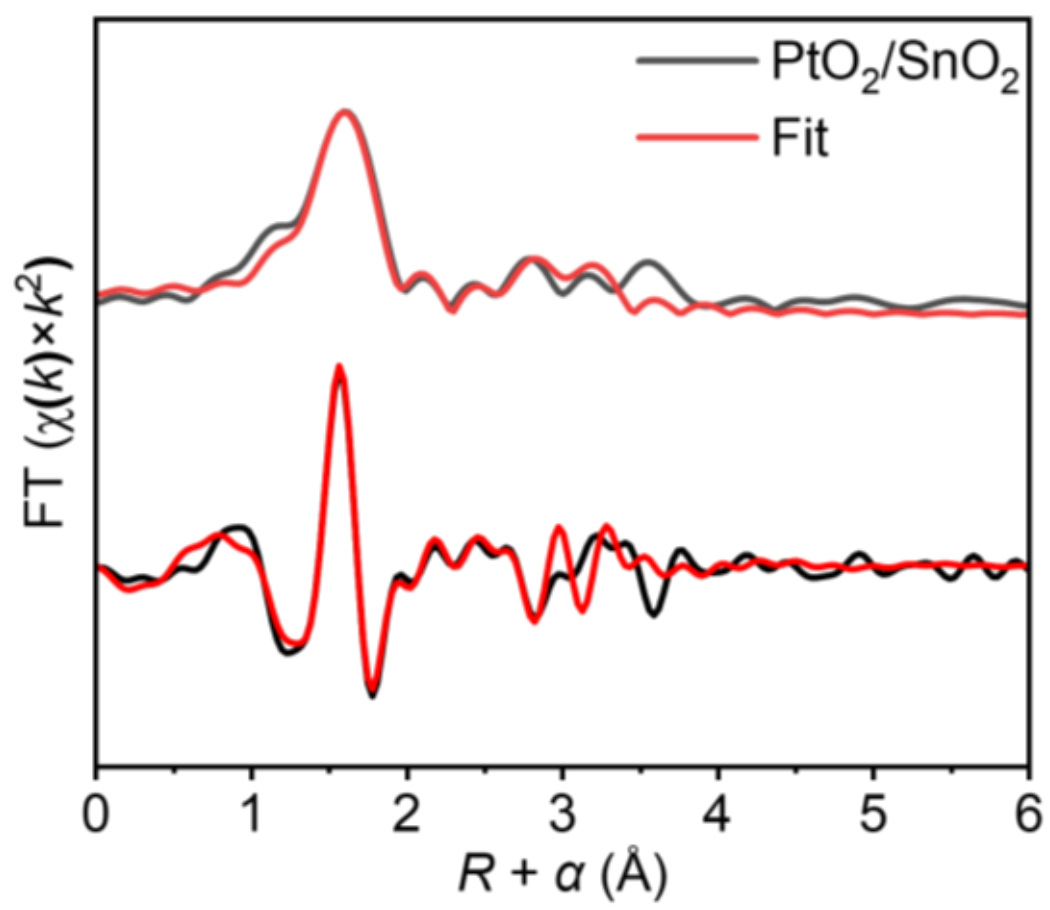

Fig. R10 The fit of the FT EXAFS spectra (top) and the FT imaginary portion (bottom) of PtO_2/SnO_2.

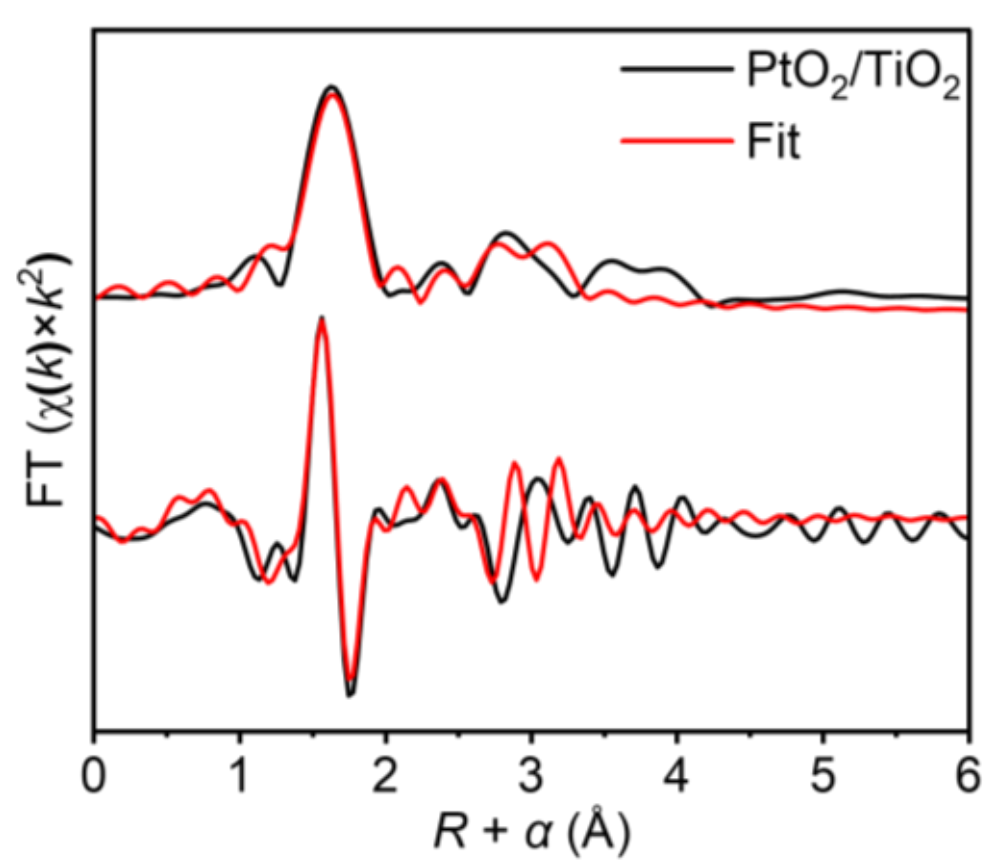

Fig. R11 The fit of the FT EXAFS spectra (top) and the FT imaginary portion (bottom) of PtO_2/TiO_2.

Table R1. Curve fitting parameters for Pt L_3-edge EXAFS of PtO_2/SnO_2 and PtO_2/TiO_2.

Sample	Path	CNs	R (Å)	σ^2 (Å^2)	ΔE_0 (eV)	R-factor

PtO_2/SnO_2	Pt–O	4.1±0.3	1.99±0.01	0.002±0.001	8.8±0.7	0.006
	Pt–(O)–Pt	2.8±1.2	3.12±0.02	0.004±0.003		
PtO_2/TiO_2	Pt–O	4.8±1.1	2.00±0.02	0.001[a]	12.1±2.5	0.045
	Pt–(O)–Pt	3.3±4.9	3.04±0.06	0.002[a]		

CN, coordination number; *R*, the distance between the absorber and backscatter atoms; σ^2, Debye-Waller factor; ΔE_0, inner potential correction; R-factor indicates the goodness of the fit. The obtained S_0^2 of Pt foil was 0.90, and S_0^2 was fixed in the subsequent fitting of Pt L_3-edge for the samples. EXAFS curves fitting ranges: PtO_2/SnO_2, 3.0 $Å^{-1} \leq k \leq 13$ $Å^{-1}$, 1.15 Å ≤ R ≤ 2.8 Å; PtO_2/TiO_2, 3.0 $Å^{-1} \leq k \leq 12.5$ $Å^{-1}$, 1.2 Å ≤ R ≤ 2.7 Å. [a] σ^2 was constrained to positive values during fitting.

In addition, following the reviewer's suggestion, we collected the Pt L_3-edge EXAFS data of PtO_x/Al_2O_3 for comparison and provide the results in **Fig. R12**. The Fourier-transformed EXAFS spectrum of PtO_x/Al_2O_3 shows clear Pt–O and higher-shell Pt–(O)–Pt scattering features, supporting the presence of oxidic PtO_x species in this reference sample. However, the structural implication of the Pt–(O)–Pt contribution in PtO_x/Al_2O_3 is different from that in PtO_2/SnO_2 and PtO_2/TiO_2, because PtO_x/Al_2O_3 does not form an epitaxially registered PtO_2 overlayer. As shown by HAADF-STEM imaging, Pt deposited on Al_2O_3 forms PtO_x clusters rather than a continuous lattice-matched PtO_2 overlayer (**Fig. R13**). Therefore, PtO_x/Al_2O_3 serves as a conventional non-epitaxial oxide-supported Pt reference for catalytic comparison, but it is not an appropriate structural reference for evaluating epitaxial strain. Accordingly, the EXAFS data of PtO_x/Al_2O_3 were not incorporated into the revised strain-analysis section, which focuses on the epitaxially matched PtO_2/SnO_2 and PtO_2/TiO_2 systems.

Regarding the bulk PtO_2 reference, we have removed this reference from the revised main figure to avoid any potential misunderstanding. We will further clarify this point in detail in our response to Q6.

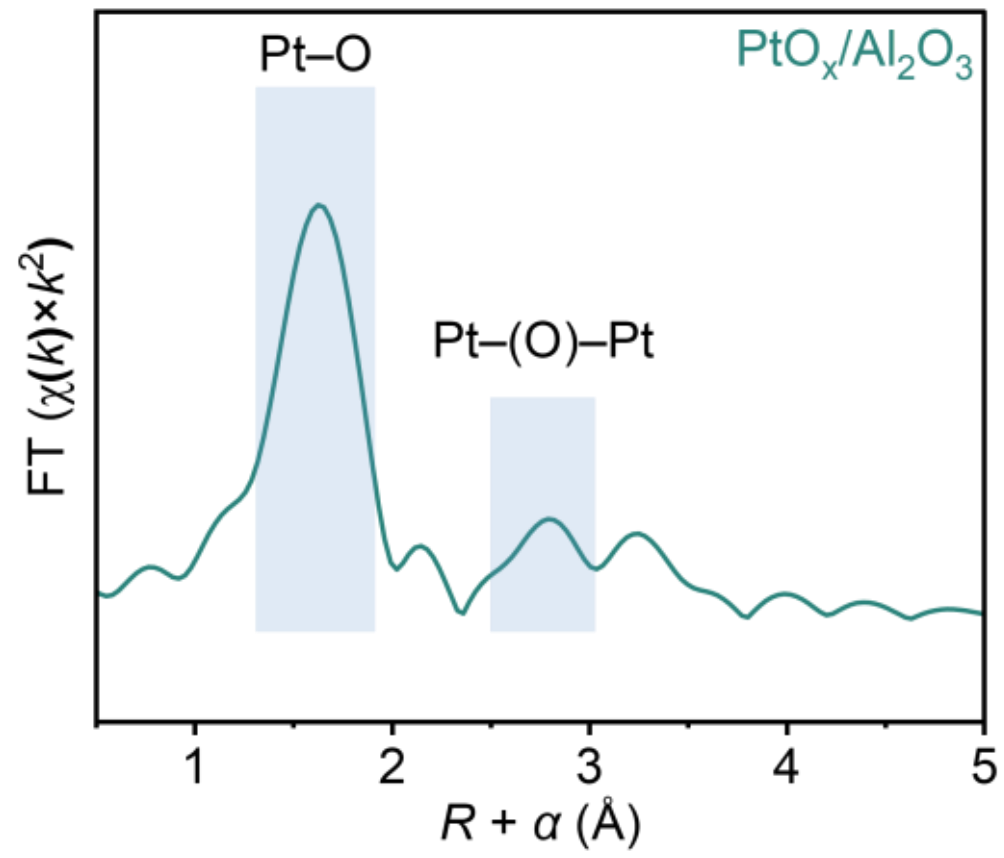

Fig. R12 FT-EXAFS spectrum at the Pt L_3-edge of PtO_x/Al_2O_3 catalyst.

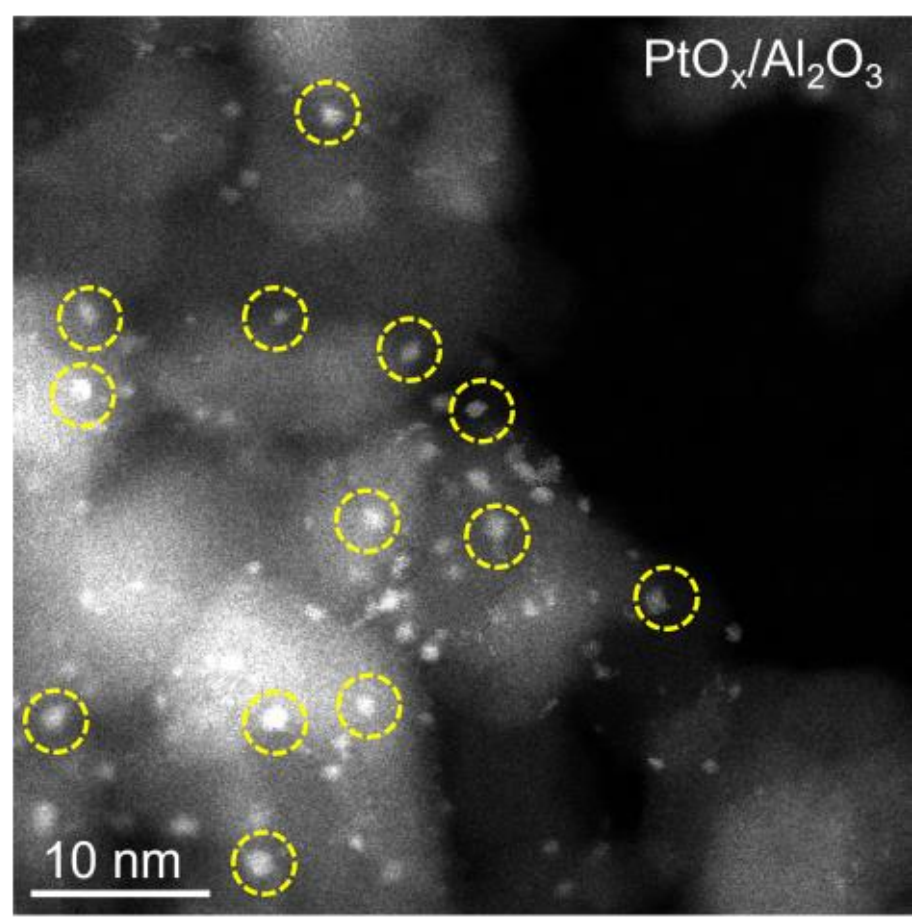

Fig. R13 Representative HAADF-STEM image of PtO_x/Al_2O_3. The yellow circle regions in HAADF-STEM image identify the Pt clusters.

Fig. R9 has been incorporated into the revised manuscript as Fig. 2h, Figs. R10, R11, and R13, together with Table R1, have been incorporated into the revised Supplementary Information as Supplementary Figs. 19, 20, and 12, and Supplementary Table 4, respectively. The related discussion has also been added to the revised manuscript on Page 11.

Q2: While Supplementary Table 6 presents a comparison of the performance of the SnO_2 based catalysts with the literature, the unit is moles reacted per gram Pt per second. Very

few of the catalysts in the literature are atomically dispersed, so the value of reactivity needs to be divided by the fractional dispersion to get a fair comparison. Or the dispersion of the Pt should be included in this table.

Response:

We sincerely appreciate the reviewer's valuable suggestion. Following the reviewer's suggestion, we have revised the catalytic comparison table mentioned by the reviewer by adding the reported Pt dispersion and, where sufficient information is available, the corresponding TOF values normalized by exposed Pt sites.

This revised comparison is presented below as **Table R2** and has been included as Supplementary Table 5 in the revised Supplementary Information.

Table R2 Comparison of catalytic performance in lean-burn CH_4 oxidation for reported Pt-based catalysts.

Sample	Pt loading (wt%)	Feed gas composition	WHSV (mL g^{-1} h^{-1})	T_{90} (°C)	Reaction rate (μmol g_{Pt}^{-1} s^{-1})	Pt dispersion (%)	TOF (s^{-1})	Ref.
**PtO_2/SnO_2**	**0.73**	**1% CH_4, 20% O_2, N_2**	**30,000**	**370**	**15.4 (260 °C)** **87.1 (300 °C)**	**100**	**3.00×10^{-3} (260 °C) 1.70×10^{-2} (300 °C)**	**This work**
Pt/ZrO_2	1.0	0.2% CH_4, 10% O_2, He	30,000	425	3.2 (300 °C)	-	-	1
SO_2 treated Pt/CeO_2	4	0.05% CH_4, 8% O_2, Ar	15,000 h^{-1}	>500	4.6 (300 °C)	-	-	2
Pt/δ-Al_2O_3 (N0)	1.5	1% CH_4, 4% O_2, He	-	485	12.2 (300 °C)	95	2.00×10^{-3} (300 °C)	3
Pt/Alumina	1.5	0.3% CH_4, 16% O_2, He	78,000	-	15.4 (300 °C)	81	3.70×10^{-3} (300 °C)	4
Pt_1/CF-W	1.0	0.5% CH_4, 20% O_2, N_2	20,000	386	83.6 (300 °C)	100	1.63×10^{-2} (300 °C)	5

Pt/γ-Al_2O_3	1	0.2% CH_4, 10% O_2, N_2	30,000	510	0.1 (310 °C)	37	5.83×10^{-5} (310 °C)	6
Pt/Cr_2O_3	1	0.2% CH_4, 10% O_2, N_2	30,000	390	6.8 (310 °C)	93	1.55×10^{-3} (310 °C)	6
Pt/rutile-400	0.56	1% CH_4, 20% O_2, He	36,000	~390	35.9 (310 °C)	22.5	3.11×10^{-2} (310 °C)	7
0.5H_2O_2-Pt_1/R-Mn_2O_3-5D	0.82	1% CH_4, 18% O_2, He	20,000	605	24.1 (400 °C)	100	4.70×10^{-3} (400 °C)	8
Pt@CeO_2 (13.8 nm)	1.0	1% CH_4, 2% O_2, N_2	555,555	600	686.9 (450 °C)	-	-	9
Pt/Al_2O_3	1.8	0.2% CH_4, 10% O_2, N_2	400,000	>625	20.5 (525 °C)	-	-	10
1Pt/Fe_2O_3-C700	1.9	0.5% CH_4, 3% O_2, He	90,000	>700	558 (700 °C)	100	0.1 (700 °C)	11
Pt/r-TiO_2	2.01	1% CH_4, air	36,000	382	-	76.87	-	12
Pt/SnO_2	1.0	0.2% CH_4, 10% O_2, N_2	60,000	391	-	-	-	13
Pt/CeO_2/3DOM LF	1.0	1% CH_4, 20% O_2, Ar	45,000	502	-	-	-	14

Q3: There is no in-situ data validating the stability of the SnO_2 based catalysts. After methane oxidation, the sample is cooled and ex-situ analysis is performed. It is well known that dispersed Pt will get oxidized upon air exposure. Hence, the XPS results have to be considered in light of the tendency of Pt to get oxidized upon air exposure.

Response:

We sincerely thank the reviewer for this helpful suggestion.

We agree that in situ XPS would be valuable for directly probing the Pt chemical state under methane oxidation conditions. We also considered the possibility of in situ near-

ambient-pressure XPS in response to the reviewer's concern. However, in our available setup, the maximum accessible pressure is below 1 mbar, which is far below the pressure used in the catalytic methane oxidation tests (> 1000 mbar). According to the Pt-O phase diagram shown in **Fig. R14** (*J. Phys. Chem. C* 2017, 122, 662–669), PtO_2 is thermodynamically unstable at the reaction-treatment temperature (500 °C) under such low-pressure conditions (< 10^{-3} bar) and tends to decompose into metallic Pt. Therefore, in situ XPS performed at approximately 1 mbar would not faithfully reproduce the actual methane oxidation environment and could artificially perturb the PtO_2 phase.

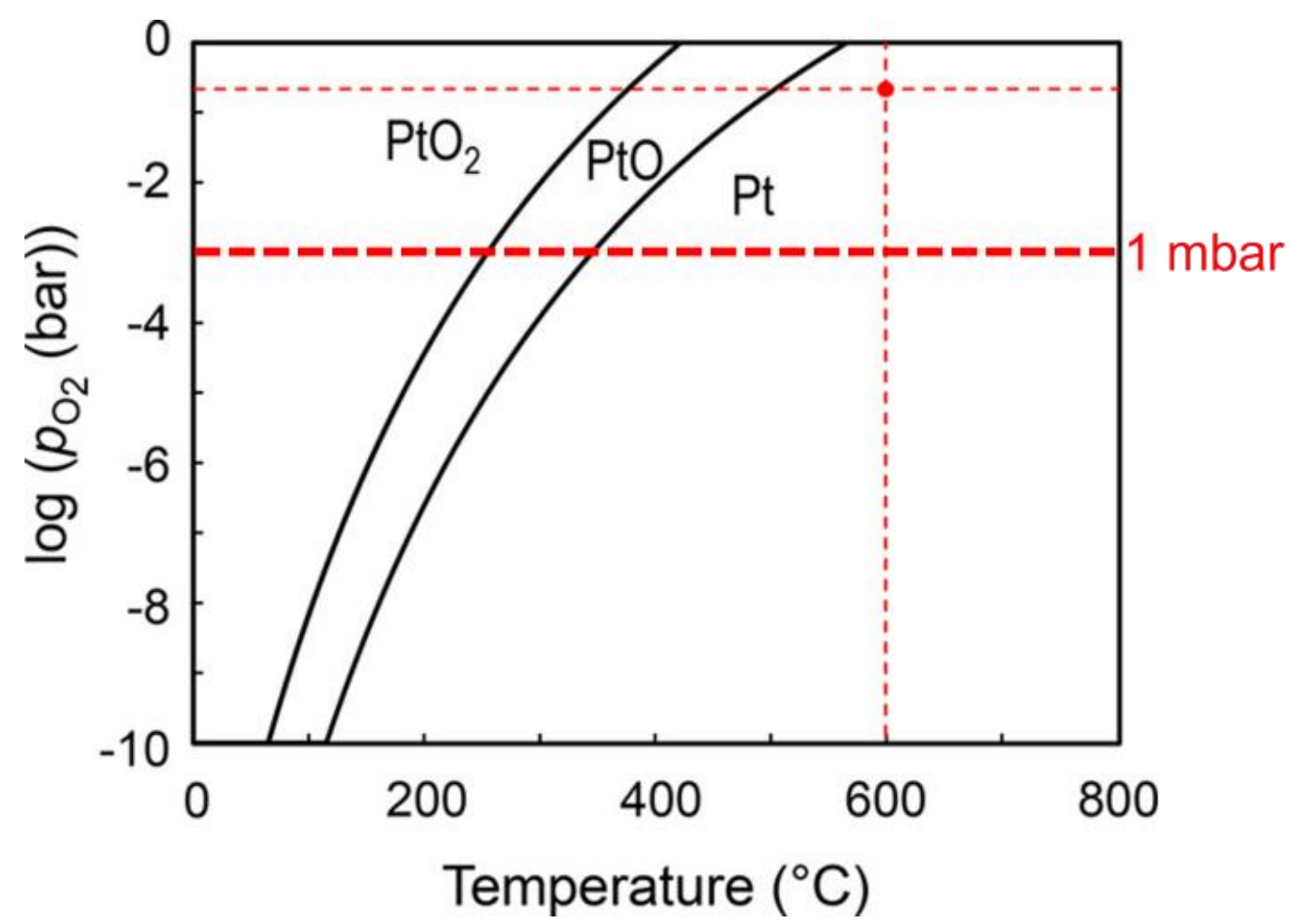

Fig. R14 Phase relationships in the partial-oxygen pressure versus temperature plots of the Pt–O system. (*J. Phys. Chem. C* 2017, 122, 662–669).

For this reason, and to directly address the reviewer's concern regarding possible air-induced oxidation during conventional ex situ handling, we performed quasi-in situ XPS measurements on PtO_2/SnO_2 after methane oxidation. A pretreatment chamber was used for the catalyst reaction treatment, following our previously established method (*Nat. Catal.* 2022, 5, 99–108). After three methane oxidation cycles under real reaction conditions, the chamber was evacuated, and the sample was directly transferred into the vacuum analysis chamber without exposure to air. This procedure minimizes air-induced oxidation during sample handling and, importantly, allows the catalyst to be treated under real methane oxidation conditions before vacuum transfer. Therefore, compared with conventional ex

situ XPS and low-pressure near-ambient-pressure XPS, this quasi-in situ approach provides a more representative evaluation of the Pt chemical state after methane oxidation in our experimental setup.

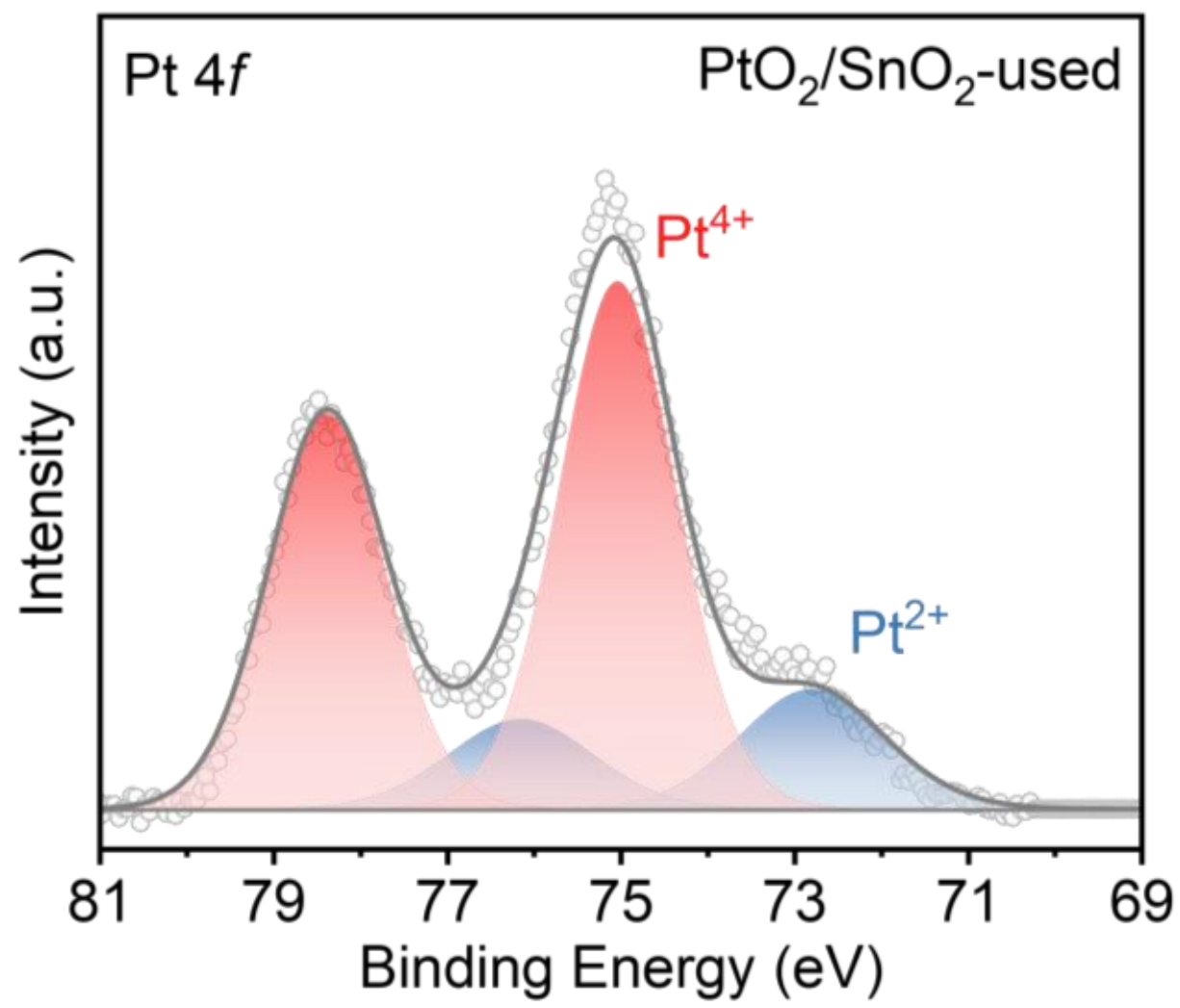

Fig. R15 Quasi-in situ XPS of Pt 4*f* region for PtO_2/SnO_2-used.

As shown in **Fig. R15**, the quasi-in situ Pt 4*f* XPS spectrum of PtO_2/SnO_2-used shows a Pt^{4+} $4f_{7/2}$ component at 75.0 eV and a Pt^{2+} $4f_{7/2}$ component at 72.8 eV. The relative proportions of Pt^{2+} and Pt^{4+} are 22.0% and 78.0%, respectively. Notably, the Pt^{4+} fraction in the quasi-in situ XPS spectrum of PtO_2/SnO_2-used is higher than that in both the conventionally measured fresh and used PtO_2/SnO_2 catalysts. This observation indicates that the high Pt^{4+} fraction is not an artifact caused by air exposure. If air exposure during conventional ex situ handling were the dominant origin of Pt oxidation, the conventionally measured used sample would be expected to show an equal or even higher Pt^{4+} fraction than the quasi-in situ sample. Instead, the opposite trend is observed.

This difference suggests that the highly oxidized Pt-O surface state generated under the lean methane oxidation atmosphere is partially relaxed during conventional ex situ handling, including cooling, air exposure, and subsequent evacuation before XPS analysis. Such relaxation may involve reorganization of surface Pt-O coordination and/or loss of weakly bound oxygen-containing surface species, leading to a Pt oxidation-state

distribution closer to that of the fresh catalyst. Therefore, conventional ex situ XPS likely underestimates, rather than overestimates, the oxidation state of Pt after methane oxidation.

Overall, the quasi-in situ XPS results demonstrate that the Pt species in PtO_2/SnO_2 remain predominantly oxidized after methane oxidation treatment, even when air exposure is avoided. Therefore, the oxidized Pt signals observed after reaction should be attributed to the retained oxidized Pt species associated with PtO_2-MO species rather than to air-induced oxidation during conventional ex situ sample handling.

Fig. R15 has been added as Supplementary Fig. 34 in the revised Supplementary Information, and the corresponding discussion has been included on Page S36.

Q4: Figure 1b is meant to show that there are no XRD reflections attributed to Pt. But the figure is misleading since the peaks are not labelled. They should be clearly labelled as coming from the SnO_2 or TiO_2 support.

Response:

We sincerely thank the reviewer for this helpful suggestion. Following the reviewer's recommendation, we have revised Fig. 1b by clearly labelling the XRD diffraction peaks originating from the SnO_2 and TiO_2 supports, as shown in **Fig. R16**.

Fig. R16 has been included as Fig. 1b in the revised manuscript.

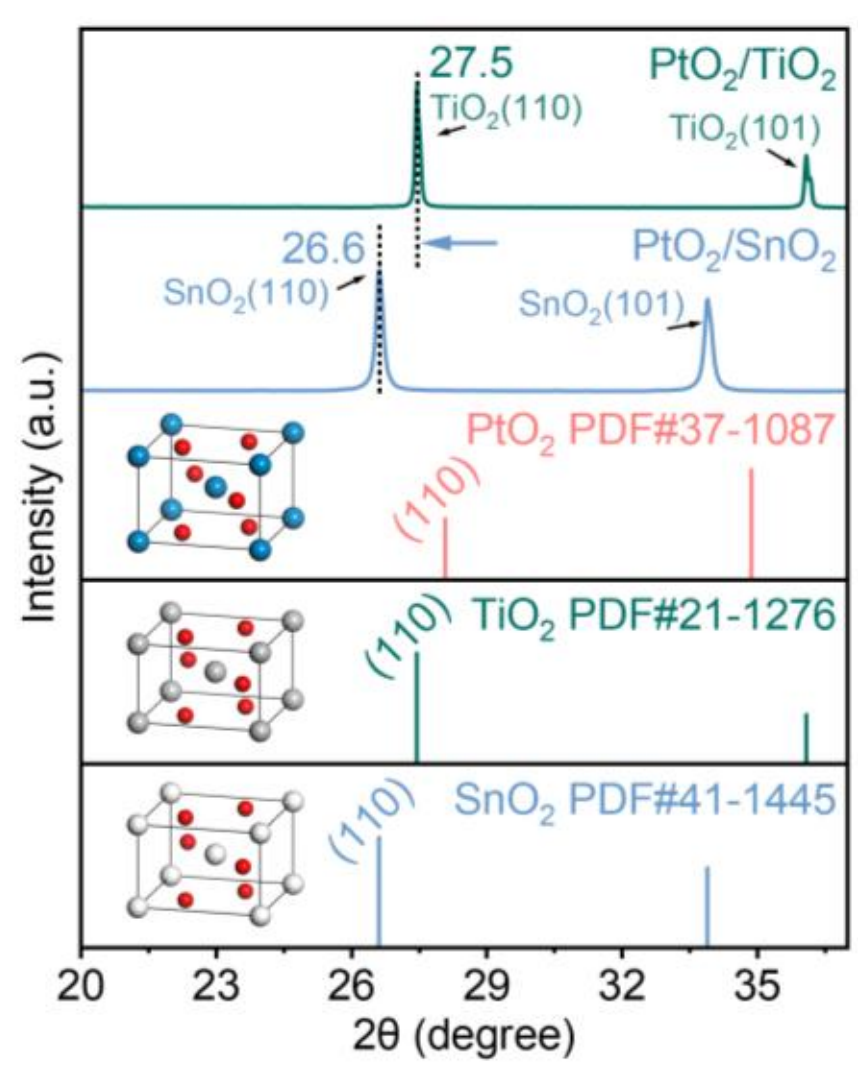

Fig. R16 The XRD patterns of PtO_2/SnO_2, PtO_2/TiO_2, and the unit cell schematic illustration for PtO_2, TiO_2, and SnO_2.

Q5: When showing images such as Figure 1c, the authors need to be aware that the identity of the surface layer (labelled PtO_2) cannot be conclusively identified from the contrast, or the lattice spacing. The authors need to show EDS analysis of each of these images so the reader can see the Pt loading and compare it with the average loading, as inferred from the lower magnification images shown in the supplementary materials.

Response:

We sincerely thank the reviewer for this important comment.

We would first like to clarify that Fig. 1c in the main text and Supplementary Fig. 5 in the Supplementary Information (**Fig. R17**) correspond to the same region of the same PtO_2/SnO_2 sample. The image in Fig. 1c was rotated only to better align the experimental image with the structural model for a more intuitive comparison. Therefore, the EDS maps shown in Supplementary Figs. 5c and 5d correspond directly to the region displayed in Fig. 1c. We have revised the figure captions and the main text to explicitly state this correspondence.

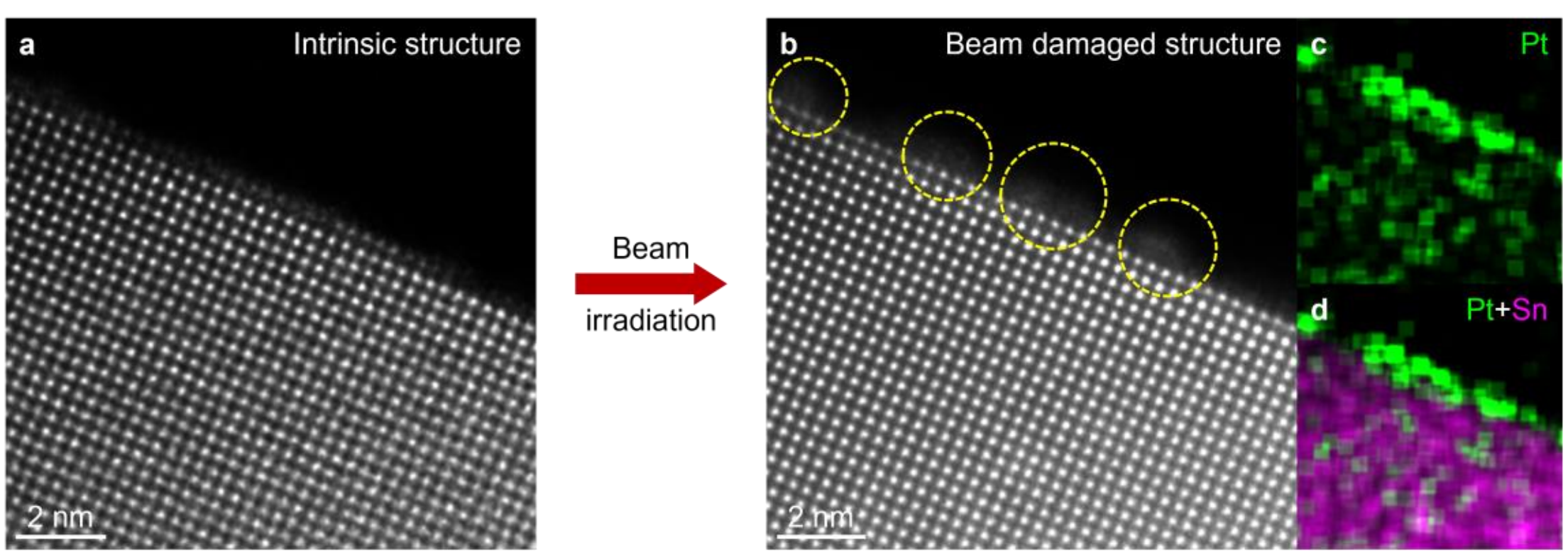

Fig. R17 (a) The HAADF-STEM image of PtO_2/SnO_2 was acquired by the low-damage HAADF-STEM imaging method under the beam current of ~ 50 pA. (b) The HAADF-

STEM image was acquired by observing the same area in (a) continuously under HAADF-STEM mode at the beam current of ~ 50 pA for a few seconds. (c-d) The corresponding EDS elemental mapping images of (c) Pt and (d) Pt+Sn compositions. The yellow circle regions in HAADF-STEM image identify the beam-damaged structure of Pt (small Pt nanoparticles).

We also note that acquiring EDS maps requires a substantially higher electron dose than low-damage HAADF-STEM imaging. Under such conditions, the atomically thin Pt oxide layer is prone to beam-induced restructuring. To verify the origin of this beam-sensitive surface layer, we performed prolonged electron-beam irradiation experiments on PtO_2/SnO_2 and pure SnO_2 under the same imaging conditions. For PtO_2/SnO_2, the topmost surface layer transformed into nanoparticle-like species upon prolonged irradiation, and EDS elemental mapping confirmed that these beam-induced nanoparticles are Pt-containing species (**Fig. R17**). In contrast, pure SnO_2 remained structurally stable under identical irradiation conditions, with no obvious surface reconstruction or particle formation (**Fig. R18**). This comparison supports the assignment that the beam-sensitive topmost layer originates from loaded Pt oxide species rather than from the SnO_2 support surface. Consequently, we intentionally utilized low-damage HAADF-STEM imaging to minimize the electron beam irradiation, which allowed us to successfully capture the epitaxial relationship at the PtO_2/SnO_2 interface.

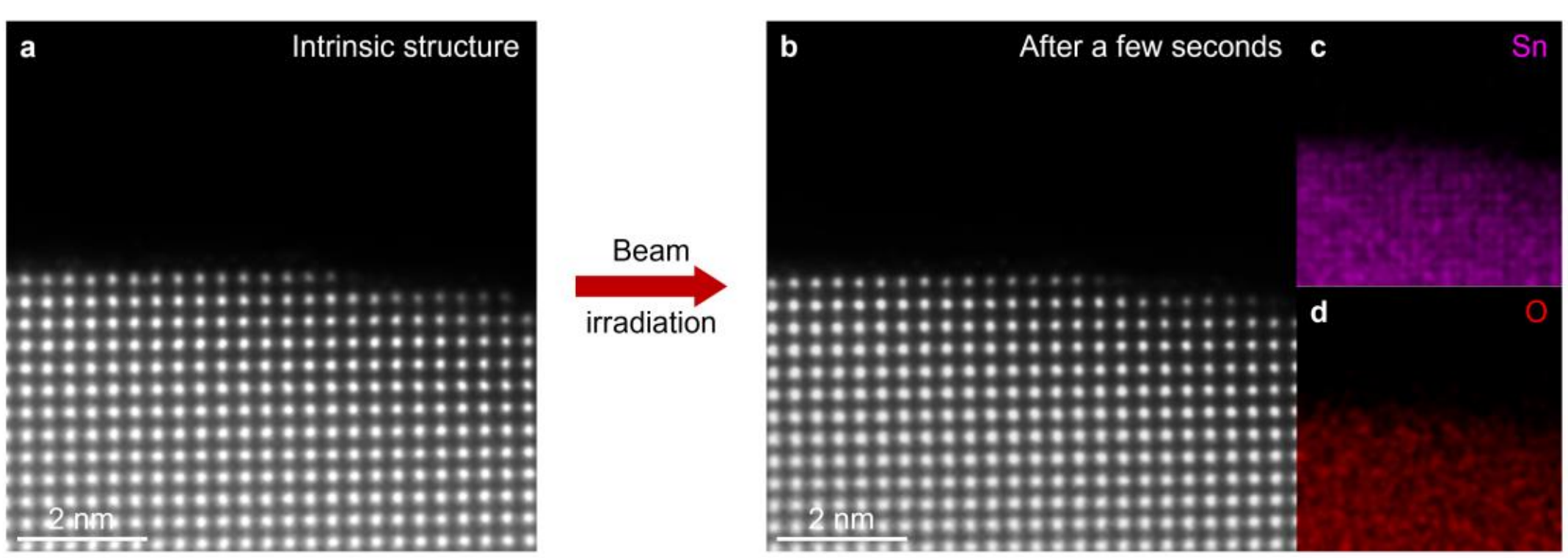

Fig. R18 (a) The HAADF-STEM image of pure SnO_2 support was acquired by the low-damage HAADF-STEM imaging method under the beam current of ~ 50 pA. (b) The HAADF-STEM image was acquired by observing the same area in (a) continuously under HAADF-STEM mode at the beam current of ~ 50 pA for a few seconds. (c-d) The corresponding EDS elemental mapping images of (c) Sn and (d) O compositions.

To assist the readers, we have added a clarifying sentence in the revised manuscript:

"*Fig. 1d presents the enlarged HAADF-STEM image of the boxed region in Fig. 1c, which corresponds to the same region shown in Supplementary Fig. 5 and was rotated for display to facilitate comparison with the structural model*".

We have also added a note after Supplementary Fig. 5 in the revised Supplementary Information:

"*The HAADF-STEM image in (a) corresponds to the same region shown in Fig. 1c, before rotation for display.*"

Fig. R18 has been added as Supplementary Fig. 4 in the revised Supplementary Information.

Q6: Figure 2g shows the XANES for the PtO_2/SiO_2 compared with bulk PtO_2. The preparation of this PtO_2 reference needs to be described and its XRD pattern included in the SM. The reactivity of this reference PtO_2 needs to be compared (per surface Pt atom) with the catalysts described here (see comment 1).

Response:

We sincerely thank the reviewer for this helpful comment.

In the original manuscript, the bulk PtO_2 spectrum was included only as a conventional Pt(IV) oxide reference for XANES/EXAFS oxidation-state comparison, similar to the use of Pt foil as a Pt^0 reference. Such bulk PtO_2 references are commonly used in Pt L_3-edge XAS studies as benchmarks for high-valent Pt oxide species in many studies (*Nat. Mater.*

2023, 22, 619–626; *Nat. Commun.* 2024, 15, 6827). However, this bulk PtO_2 reference was provided by the synchrotron beamline as a standard reference material and was not prepared as a catalyst sample in this work. Its role was therefore limited to providing a qualitative oxidation-state benchmark rather than serving as a catalytic reference or a structural analogue of the epitaxially strained PtO_2-MO overlayer on SnO_2.

The reviewer's request prompted us to reconsider the role of this bulk PtO_2 reference in the main figure. Although PtO_2 can exist in both α-PtO_2 and β-PtO_2 forms, bulk β-PtO_2 is a high-pressure phase and is unlikely to be present as a conventional bulk reference under ambient-pressure conditions. Thus, bulk PtO_2 references commonly used in XAS are generally associated with conventional α-PtO_2 reference materials (*Platinum Metals Rev.* 2011, 55, 233–245). In contrast, the PtO_2-MO in this work adopts a rutile-type β-PtO_2 structure on SnO_2 through epitaxial stabilization. Therefore, placing the conventional bulk α-PtO_2 reference together with PtO_2/SnO_2 in the main figure could potentially imply an unintended structural analogy between the two.

To avoid this potential misunderstanding, we have removed the bulk PtO_2 reference from the revised main figure and clarified in the revised text that the assignment of high-valent Pt oxide species is based on the combined evidence from XPS, XANES/EXAFS and CO-DRIFTS, rather than on direct structural comparison with bulk PtO_2 alone.

Following the reviewer's suggestion in Q1, we have instead compared the methane oxidation activity of the catalysts prepared in this work on the basis of exposed Pt sites, and the corresponding site-normalized activity/TOF values have been added to the revised Supplementary Table 5.

Q7: Figure 2d shows that the PtO_2 on TiO_2 is reduced at a much lower temperature than PtO_2/SnO_2. This is consistent with the postulate that the SnO_2 based catalyst is strained and hence more difficult to reduce. As pointed out by the authors (line 262), PtO_2 will generally

convert into metallic Pt as it is heated, so retaining the oxide is key to the performance of these catalysts. The evidence presented here in terms of the multiple cycles of methane oxidation and longer-term stability as well as post-reaction characterization, suggests that this PtO_2/SnO_2 is indeed stable under the conditions. For practical applications involving these catalysts, it would be good to know at what temperature this strained PtO_2 ultimately starts to convert to particulate form.

Response:

We sincerely thank the reviewer for this constructive suggestion. To determine the thermal stability limit of the strained PtO_2 species, we performed additional calcination treatments of PtO_2/SnO_2 in air at 600, 700 and 800 °C. The resulting samples were denoted as PtO_2/SnO_2-600, PtO_2/SnO_2-700 and PtO_2/SnO_2-800, respectively, and were characterized by XRD.

As shown in **Fig. R19**, no obvious Pt-related crystalline reflections are observed after calcination at 600 °C, indicating that the Pt species remain highly dispersed and that no XRD-detectable Pt-containing particles are formed at this temperature. In contrast, after calcination at 700 °C, a weak diffraction peak assignable to metallic Pt(111) begins to appear, indicating the onset of thermal decomposition/aggregation of Pt oxide species into particulate metallic Pt. After calcination at 800 °C, the Pt(111) reflection becomes more pronounced, confirming further growth of metallic Pt particles. These results indicate that the strained PtO_2 species in PtO_2/SnO_2 start to transform into XRD-detectable particulate Pt at approximately 700 °C in air.

We have added these additional thermal stability data as Supplementary Fig. 35 and have included the corresponding discussion in the revised Supplementary Information.

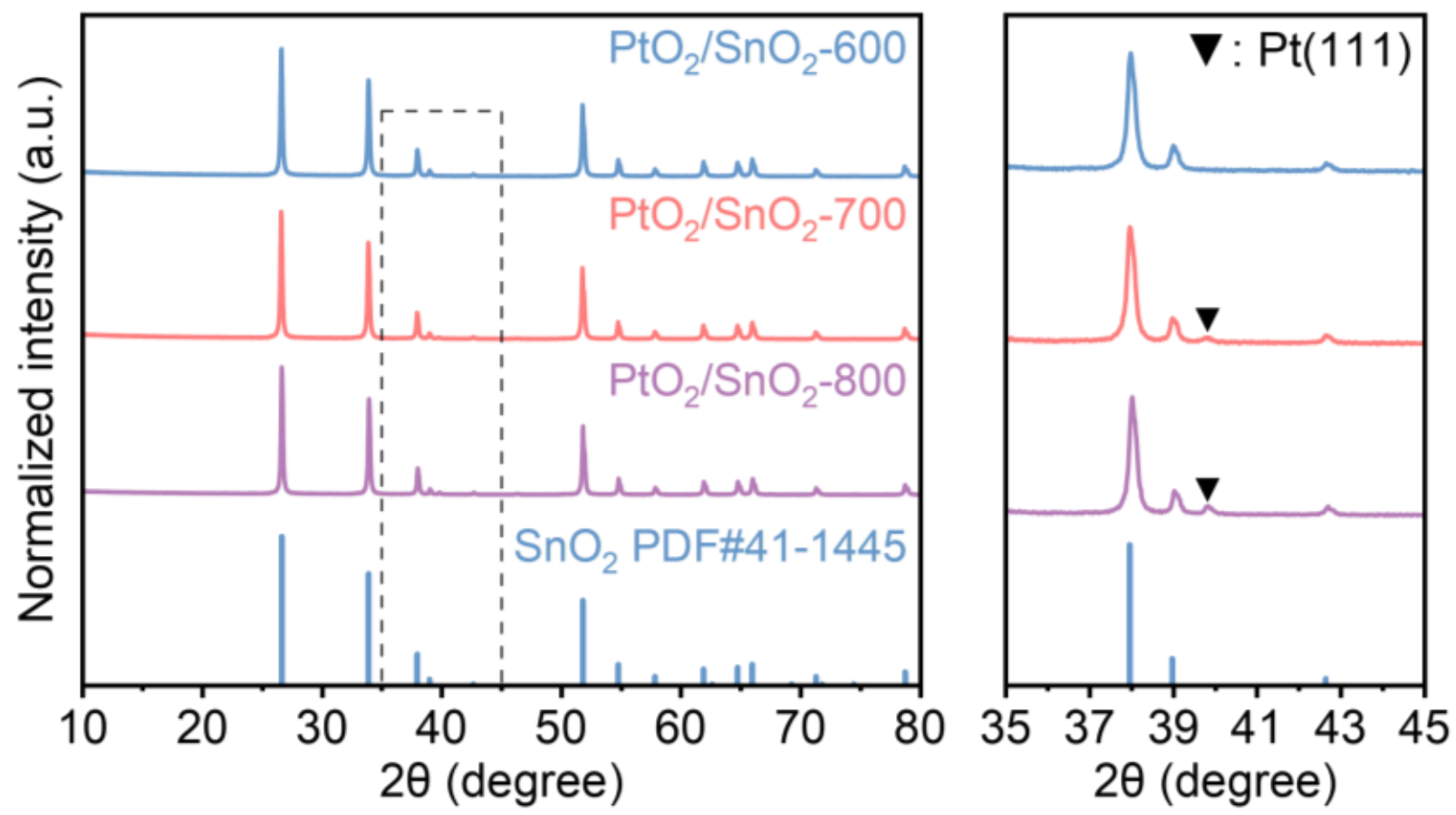

Fig. R19 The XRD patterns of PtO_2/SnO_2-600, PtO_2/SnO_2-700, and PtO_2/SnO_2-800.

Q8: Since the PtO_2/SnO_2 can be easily reduced in H_2 (Fig. 2d), will it convert back to the strained oxide structure. In other words, is the catalyst after TPR transformed irreversibly into a particulate Pt structure?

Response:

We sincerely thank the reviewer for this insightful comment.

Following the reviewer's suggestion, we performed a reduction-reoxidation experiment to examine whether the reduced Pt species can be restored to the original strained PtO_2-MO structure. Because the Pt loading in the standard PtO_2/SnO_2 catalyst is low, XRD is not sufficiently sensitive to detect possible crystalline phases formed after reduction and reoxidation. Therefore, we used the high-loading 8.1 wt% PtO_2/SnO_2 sample as a structurally more sensitive model to track phase evolution by XRD.

Specifically, 8.1 wt% PtO_2/SnO_2 was first reduced at 400 °C for 2 h under a 10% H_2/Ar atmosphere, and the obtained sample was denoted as 8.1 wt% PtO_2/SnO_2-400R. The reduced sample was subsequently reoxidized at 500 °C for 12 h in air, and the resulting sample was denoted as 8.1 wt% PtO_2/SnO_2-400R-500O.

As shown in **Fig. R20**, besides the diffraction peaks from the SnO_2 support, the XRD pattern of 8.1 wt% PtO_2/SnO_2-400R shows additional pronounced diffraction peaks

assignable to $PtSn_2$ and Pt_2Sn_3 phases, indicating that high-temperature H_2 reduction induces the formation of Pt-Sn intermetallic/alloy phases. After reoxidation at 500 °C in air, the $PtSn_2$ and Pt_2Sn_3 reflections are no longer dominant, while new diffraction peaks assigned to the Pt_3Sn phase are clearly observed. This result indicates that subsequent air reoxidation does not restore the original strained PtO_2-MO structure, but instead leads to further restructuring of the reduced Pt-Sn phases into another stable Pt-Sn intermetallic phase.

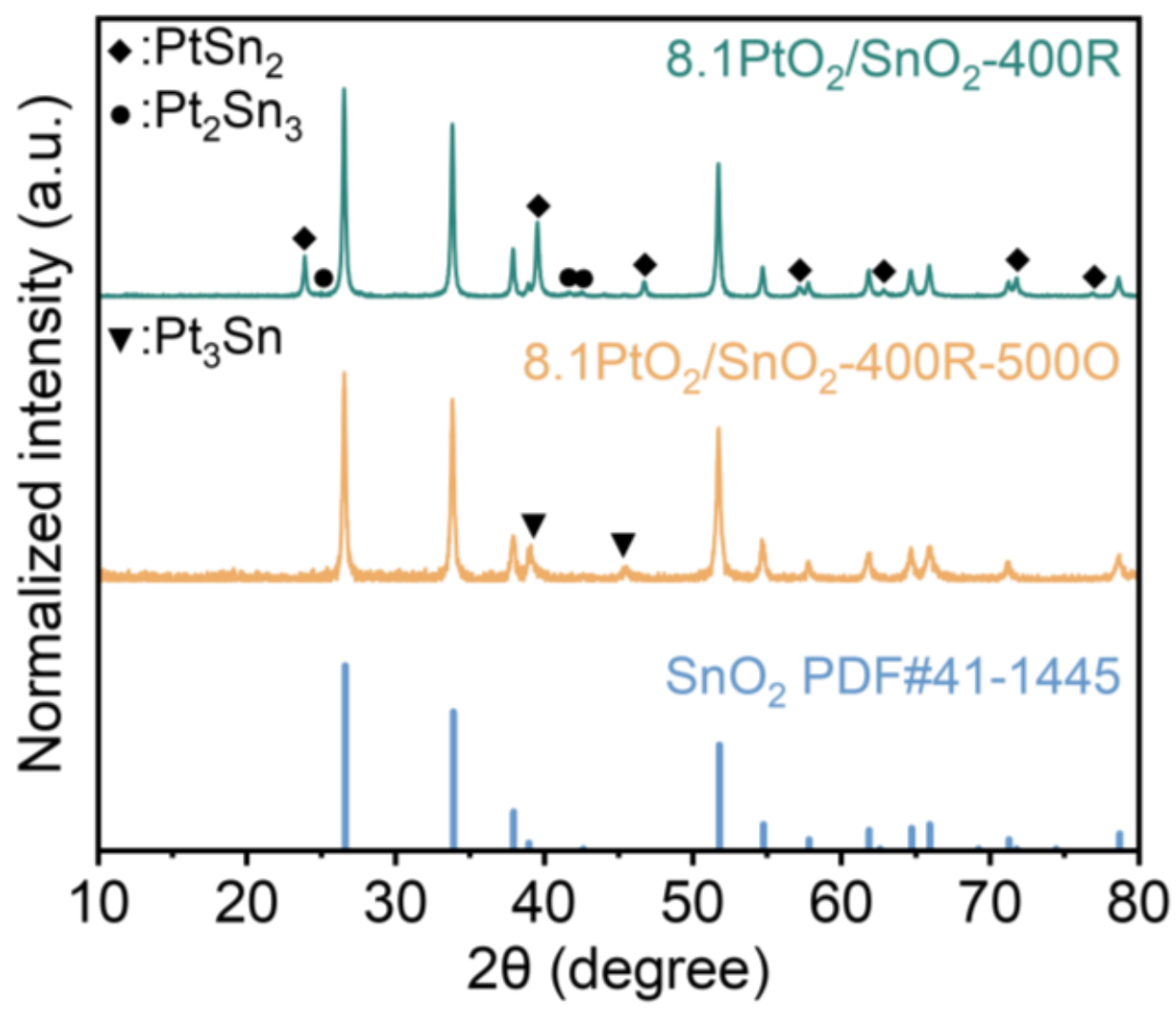

Fig. R20 The XRD patterns of 8.1 wt% PtO_2/SnO_2-400R and 8.1 wt% PtO_2/SnO_2-400R-500O.

Therefore, the structural transformation of PtO_2/SnO_2 after high-temperature H_2 reduction is largely irreversible, leading to XRD-detectable Pt-Sn-containing particulate phases rather than recovery of the atomically thin strained PtO_2 overlayer. Since this experiment is not directly related to the stability of PtO_2-MO under lean methane oxidation conditions, we have not incorporated these data into the revised manuscript or Supplementary Information to avoid distracting from the main focus of the study.

Q9: The reactivity for methane oxidation on the PtO_2/SnO_2 is clearly superior to the reference PtO_x/Al_2O_3 (Figure 3a). But additional explanation is needed for Figure 3b where a plateau in reactivity is reached at a loading ~1.95 wt%. The authors argue that after the theoretical monolayer loading is exceeded, the specific activity drops (Figure 3b). The authors need to provide more details for the derivation of this theoretical maximum. The equation presented on line 167 of the SM leaves many open questions. The BET surface area is not stated anywhere, and neither is sigma Sn or Sigma Pt. The authors need to state how this areal density of Sn or Pt atoms is determined. And once this areal density (mol/m^2) is known, why can't they simply multiply by the BET surface area (m^2/g) and molecular weight to get the maximum loading (gm Pt/gcatalyst)?

Response:

We sincerely thank the reviewer for the constructive comments and for pointing out the need for a more transparent derivation of the theoretical maximum PtO_2 monolayer dispersion threshold.

In our epitaxial growth model, the PtO_2 overlayer is assumed to be commensurately aligned with the $SnO_2(110)$ surface, with each Pt site in the PtO_2 layer corresponding to one surface Sn site of the $SnO_2(110)$ model. Therefore, the areal density of Pt sites in the PtO_2 monolayer was taken to be equal to the areal density of surface Sn sites, namely $\sigma_{Pt} = \sigma_{Sn}$. The surface Sn density σ_{Sn} was determined by counting the number of surface Sn atoms in the $SnO_2(110)$ surface unit cell and dividing it by the corresponding surface area. This gives $\sigma_{Sn} = \sigma_{Pt} = 9.17$ atoms nm^{-2}, corresponding to 1.52×10^{-5} mol m^{-2}.

Using the measured BET surface area of the SnO_2 support, $S_{BET} = 6.7\ m^2\ g^{-1}$, the Pt mass required to form one ideal PtO_2 monolayer on 1 g of SnO_2 is calculated as:

$$m_{Pt} = \sigma_{Pt} \times M_{Pt} \times S_{BET}$$

$$= 1.52 \times 10^{-5}\ \text{mol m}^{-2} \times 195.08\ \text{g mol}^{-1} \times 6.7\ \text{m}^2\ \text{g}^{-1}$$

$$= 0.0199\ g_{Pt}/g_{SnO2}$$

The corresponding Pt loading in the final catalyst is therefore:

$$X\% = \frac{m_{Pt}}{1+m_{Pt}} \times 100\%$$

$$= 0.0199 / (1 + 0.0199) \times 100\%$$

$$\approx 1.95 \text{ wt\%}$$

Here, the denominator accounts for the total catalyst mass after Pt loading, namely 1 g of SnO_2 support plus the Pt mass required to form the ideal monolayer. This calculation is therefore equivalent to the reviewer's suggested approach based on the areal density, BET surface area and Pt atomic weight, with the additional conversion from g Pt per g SnO_2 to wt% Pt in the final catalyst.

We also clarify that 1.95 wt% should be regarded as an ideal upper-limit estimate based on the SnO_2(110) facet model. Because real SnO_2 particles contain multiple facets, steps, defects and partially inaccessible surface areas, the practical PtO_2 monolayer dispersion threshold is expected to be close to or slightly lower than this value.

To assist the readers, we have updated and expanded the clarifying Note in the revised Supplementary Information on Pages S40-S41 to provide a more detailed and transparent derivation of the theoretical maximum dispersion threshold.

"*Note: The theoretical maximum dispersion threshold for a PtO_2 monolayer on SnO_2 (X%), defined based on the SnO_2(110) facet model, was calculated by the following equations:*

$$\sigma_{Sn} = \sigma_{Pt}$$

$$X\% = \frac{\sigma_{Pt} \times M_{Pt} \times S_{BET}}{1+\sigma_{Pt} \times M_{Pt} \times S_{BET}} \times 100\%$$

Where σ_{Sn} (mol m^{-2}) is the areal density of Sn atoms on the SnO_2(110) surface, σ_{Pt} (mol m^{-2}) is the areal density of Pt atoms on the PtO_2/SnO_2(110) surface, M_{Pt} (g mol^{-1}) is the molar mass of Pt, and S_{BET} (m^2 g^{-1}) is the specific surface area of the SnO_2 support.

In our epitaxial growth model, each Pt site in the PtO_2 layer corresponds to one Sn site on the SnO_2 surface. Specifically, based on the structural model we established, the σ_{Sn} was determined to be approximately 9.17 atoms nm^{-2} (equivalent to 1.52×10^{-5} mol m^{-2}). By incorporating the measured S_{BET} of 6.7 m^2 g^{-1}, the calculated threshold is 1.95 wt%.

Because the $SnO_2(110)$ facet has the lowest surface Sn atom site density (that is, the largest surface area per surface site) among the considered facets, this value represents an upper bound. In addition, real faceted/polyhedral SnO_2 particles contain multiple facets, steps, defects, and partially inaccessible surface areas, which may further reduce the effective capacity for forming an epitaxial PtO_2 monolayer. Therefore, the practical PtO_2 monolayer dispersion threshold on SnO_2 is expected to be close to or slightly lower than 1.95 wt%."

Q10: While images of well-defined facets of SnO_2 make a convincing case for the presence of 2-D Pt on the surface, the low magnification images show typical spheroidal particles of SnO_2. This means these particles expose a variety of surface facets. Hence, when considering the areal density of Sn or Pt (see comment 9) they need to consider an average of the most commonly exposed surface facets of SnO_2.

Response:

We sincerely thank the reviewer for this constructive comment. We agree that the SnO_2 particles are not single-facet crystals and expose multiple surface facets. More precisely, the low-magnification images show faceted/polyhedral SnO_2 particles rather than ideal spheroidal particles. Therefore, the theoretical monolayer dispersion threshold should be regarded as an approximate estimate based on a representative facet model, rather than an exact experimental value for all exposed surfaces.

In the present calculation, the $SnO_2(110)$ surface was selected as the representative model for two reasons. First, $SnO_2(110)$ is generally regarded as the most thermodynamically stable low-energy facet of rutile SnO_2 and is also the facet directly used in our epitaxial structural model. Second, among the considered rutile SnO_2 facets, the $SnO_2(110)$ surface gives the largest theoretical capacity for epitaxial PtO_2 monolayer growth. Calculations based on other facets give lower monolayer dispersion thresholds.

Therefore, the value calculated from the $SnO_2(110)$ model should be regarded as an ideal upper-limit estimate.

We have revised the Supplementary Information on Page S41 to clarify that the calculated value of 1.95 wt% represents an ideal upper-limit estimate based on the $SnO_2(110)$ facet model, rather than an exact monolayer capacity for all surface sites in the real catalyst. In real faceted/polyhedral SnO_2 particles, the presence of multiple facets, steps, defects, and partially inaccessible surface areas would further reduce the effective capacity for forming an epitaxial PtO_2 monolayer. Therefore, the practical PtO_2 monolayer dispersion threshold is expected to be close to or lower than this ideal value.

Q11: Supplementary figure 28 shows XRD data for a range of Pt loadings on SnO_2. What was the treatment temperature for these samples? I have the same comment for all of the figures, where the sample treatments should be specified in the figure caption.

Response:

We sincerely thank the reviewer for this constructive suggestion. The samples shown in Supplementary Fig. 38, including the PtO_2/SnO_2 catalysts with different Pt loadings, were all prepared using the same standard treatment protocol: aging for 6 h, drying at 80 °C overnight, and calcination at 500 °C for 2 h in air.

To avoid ambiguity, we have revised the caption of Supplementary Fig. 38 to explicitly state that these samples were calcined at 500 °C for 2 h in air. In addition, to avoid repetitive descriptions in every figure caption while ensuring clarity, we have added a general statement in the revised manuscript on Page 18 specifying that, unless otherwise stated, all catalyst samples were prepared using the same standard treatment protocol. For figures involving special treatments, such as high-temperature calcination, H_2 treatment, or post-reaction characterization, we have revised the corresponding captions to explicitly state the relevant treatment conditions.

Q12: And, as I stated in my comment (2) above, an overall EDS needs to accompany all these figures, like Figure 29, so the reader can see the actual loading of Pt on the sample.

Response:

We sincerely thank the reviewer for this valuable suggestion. Following the reviewer's recommendation, we have added EDS elemental mapping for the 8.1 wt% PtO_2/SnO_2 sample. The added EDS mapping results are presented in **Fig. R21**.

Fig. R21 has been added as Supplementary Fig. 41 in the Supplementary Information.

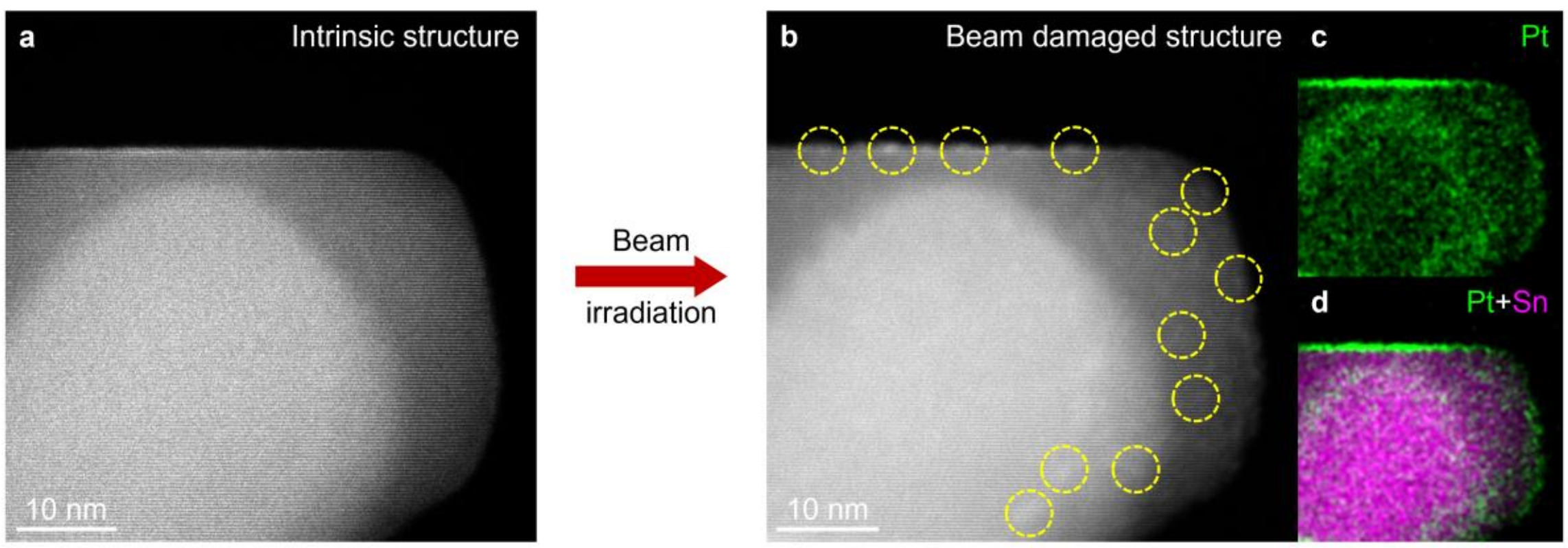

Fig. R21 (a) The HAADF-STEM image of 8.1 wt% PtO_2/SnO_2 was acquired by the low-damage HAADF-STEM imaging method under the beam current of ~ 50 pA. (b) The HAADF-STEM image was acquired by observing the same area in (a) continuously under HAADF-STEM mode at the beam current of ~ 50 pA for a few seconds. (c-d) The corresponding EDS elemental mapping images of (c) Pt and (d) Pt+Sn compositions. The yellow circle regions in HAADF-STEM image identify the beam-damaged structure of Pt (small Pt nanoparticles).

Q13: Figure 29 is very interesting in that even at a high loading of Pt (8.1 wt%), only 2-D structures are seen. The authors should present EXAFS of this catalyst, because it may

reveal the presence of Pt-O-Sn bonds which may be responsible for the enhanced reactivity and stability of these catalysts.

Response:

We sincerely thank the reviewer for this helpful suggestion. Following the reviewer's suggestion, we added Pt L_3-edge XAS and EXAFS fitting results for the 8.1 wt% PtO_2/SnO_2 catalyst.

As shown in **Fig. R22**, the Fourier transform of the k^2-weighted EXAFS spectrum of 8.1 wt% PtO_2/SnO_2 shows a dominant first-shell peak at approximately 1.7 Å before phase correction, corresponding to Pt–O coordination, and clear higher-shell features at approximately 2.8 and 3.4 Å before phase correction. The quantitative EXAFS fitting results are shown in **Fig. R23** and summarized in **Table R3**. The fitting gives a Pt–O coordination number of 4.4 ± 0.3 with a Pt–O distance of 2.01 ± 0.01 Å. The fitted Pt–(O)–Pt coordination number is 6.4 ± 3.5, with a Pt–(O)–Pt distance of 3.13 ± 0.02 Å. These results indicate that Pt remains mainly in an oxidic Pt–O coordination environment even at high Pt loading, consistent with the two-dimensional oxidic structures observed by HAADF-STEM.

To examine whether Pt–(O)–Sn interfacial linkages may contribute to the higher-shell EXAFS features, we further analyzed representative scattering paths derived from the PtO_2/SnO_2 model CIF structure. As summarized in **Table R4**, the model contains a first-shell Pt–O path at 2.08 Å, two Pt–(O)–Pt related higher-shell paths at 3.19 and 3.71 Å, and an interfacial Pt–(O)–Sn related path at 3.76 Å. The model therefore indicates that the long Pt–(O)–Pt related path and the interfacial Pt–(O)–Sn related path are very close in distance.

Because these higher-shell contributions strongly overlap, the present EXAFS data cannot reliably separate them into independent quantitative Pt–(O)–Pt and Pt–(O)–Sn shells. For this reason, the weak high-R feature in 8.1 wt% PtO_2/SnO_2 was fitted as an unresolved Pt–(O)–M contribution at 3.84 ± 0.04 Å, where M represents Pt or Sn. This assignment is consistent with the possible presence of Pt–(O)–Sn interfacial linkages

between the epitaxial PtO_2 layer and the SnO_2 support. However, because of the overlap between Pt–(O)–Pt and Pt–(O)–Sn scattering paths and the large uncertainty in the coordination number of this high-R shell, we use this Pt–(O)–M contribution only as auxiliary evidence for the high-shell interfacial structure and do not quantitatively interpret its coordination number.

Overall, the EXAFS results show that the high-loading 8.1 wt% PtO_2/SnO_2 catalyst retains an oxidic Pt–O coordination environment and exhibits higher-shell features consistent with an extended PtO_2 overlayer and possible PtO_2/SnO_2 interfacial contribution. Together with the HAADF-STEM observation of two-dimensional Pt oxide structures, these results support the formation of an epitaxially coupled PtO_2/SnO_2 interfacial structure even at high Pt loading. We have clarified in the revised manuscript that this analysis is used to support the structural assignment of the high-loading sample, while the enhanced reactivity and stability are discussed in terms of the tensile-strained PtO_2-MO structure stabilized by the rutile SnO_2 support.

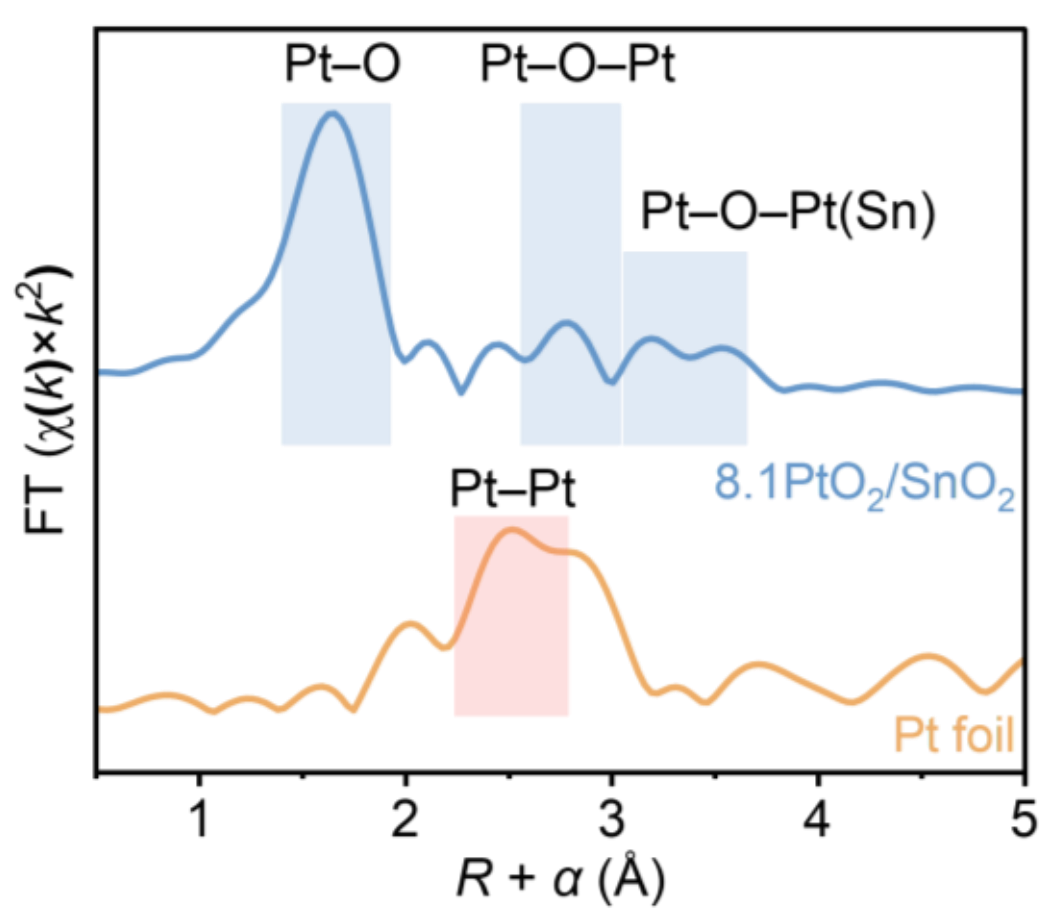

Fig. R22 FT-EXAFS spectra at the Pt L_3-edge of 8.1 wt% PtO_2/SnO_2 catalyst.

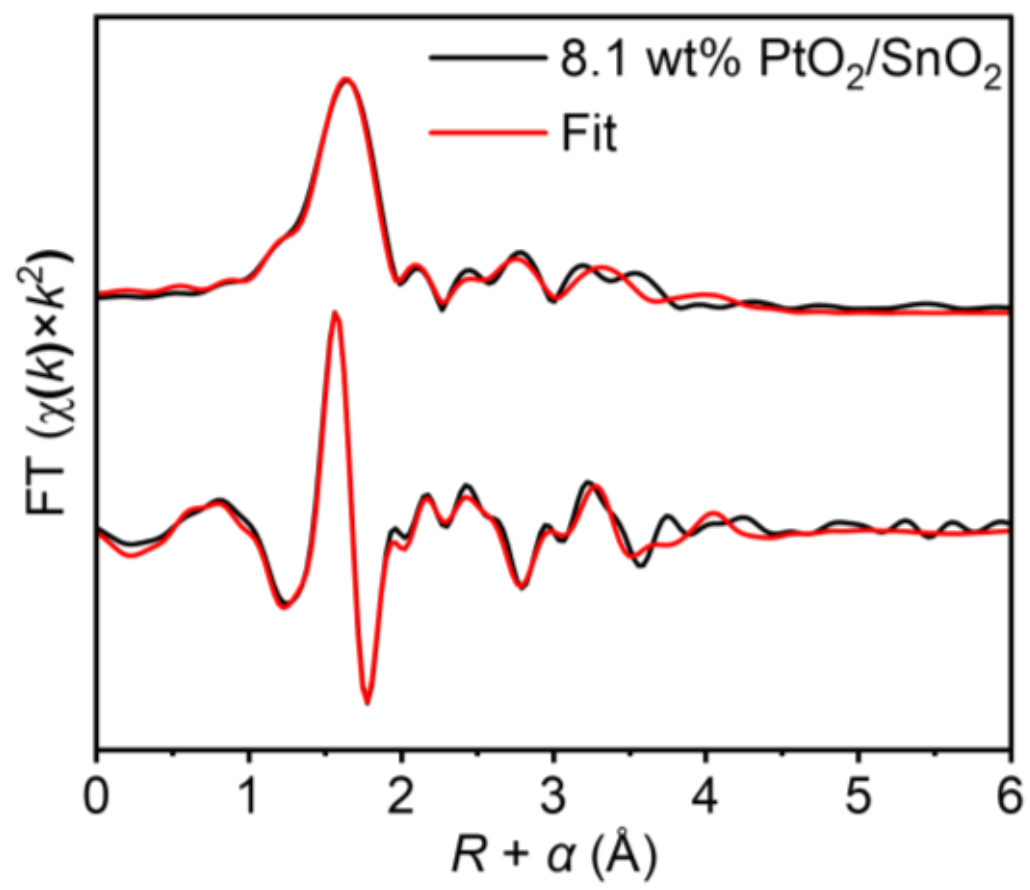

Fig. R23 The fit of the FT EXAFS spectra (top) and the FT imaginary portion (bottom) of 8.1 wt% PtO_2/SnO_2.

Table R3 Curve fitting parameters for Pt L_3-edge EXAFS of 8.1 wt% PtO_2/SnO_2.

Sample	Path	CNs	R (Å)	σ^2 (Å^2)	ΔE_0 (eV)	R-factor
8.1 wt% PtO_2/SnO_2	Pt–O	4.4±0.3	2.01±0.01	0.002±0.001	10.0±0.8	0.012
	Pt–(O)–Pt	6.4±3.5	3.13±0.02	0.012±0.005		
	Pt–(O)–M [a]	n.q.[a]	3.84±0.04	n.q.[a]		

CN, coordination number; R, the distance between the absorber and backscatter atoms; σ^2, Debye-Waller factor; ΔE_0, inner potential correction; R-factor indicates the goodness of the fit. The obtained S_0^2 of Pt foil was 0.90, and S_0^2 was fixed in the subsequent fitting of Pt L_3-edge for the samples. EXAFS curve fitting ranges: 3.0 Å^{-1} ≤ k ≤13 Å^{-1}, 1.2 Å ≤ R ≤ 3.5 Å. [a] M represents unresolved Pt or Sn scattering. According to the model-derived distances, the long Pt–(O)–Pt-related path overlaps with the interfacial Pt–(O)–Sn-related path; therefore, this high-R contribution was treated as an unresolved Pt–(O)–M shell. Its CN and σ^2 were not quantitatively interpreted due to the large fitting uncertainty. n.q., not quantified.

Table R4 Representative model-derived path distances from the PtO_2/SnO_2 CIF structure.

Path	R (Å)
Pt–O	2.08
Pt–(O)–Pt short	3.19
Pt–(O)–Pt long	3.71

Pt–(O)–Sn	3.76

The listed distances are derived from the epitaxial PtO_2/SnO_2 model CIF structure and are used only for path assignment. They are not EXAFS-fitted distances.

Figs. R22 and R23, together with Tables R3 and R4, have been incorporated into the revised Supplementary Information as Supplementary Figs. 42 and 43, and Supplementary Tables 7 and 8, respectively. The related discussion has also been added to the revised Supplementary Information on Page S45.

Q14: The authors suggest that prolonged examination in the microscope causes the 2-D structure to transform into clusters. For the reader to see this clearly, the contrast in the SM Figure 4b needs to be adjusted (make it brighter) so the reader can clearly see the structures that are formed. And more importantly, it is not clear why there is all the Pt in the region away from the SnO_2 structure (Fig. 4c). The same comment (about contrast) applies to other images where beam damaged regions are shown (as in Figure 23a) because the structures that are formed after beam damage are not visible.

Response:

We sincerely thank the reviewer for this helpful suggestion. Following the reviewer's recommendation, we prepared contrast-adjusted versions of the images referred to by the reviewer, namely Supplementary Figs. 4b and 23b in the original submission, which correspond to Supplementary Figs. 5b and 30b in the revised Supplementary Information, respectively. These contrast-adjusted HAADF-STEM images are shown in **Figs. R24** and **R25** and have been incorporated into the revised Supplementary Information as Supplementary Figs. 6 and 31, respectively. Only the brightness and contrast were adjusted, and yellow circles were added to mark the beam-induced Pt-containing nanoparticle-like species for clarity.

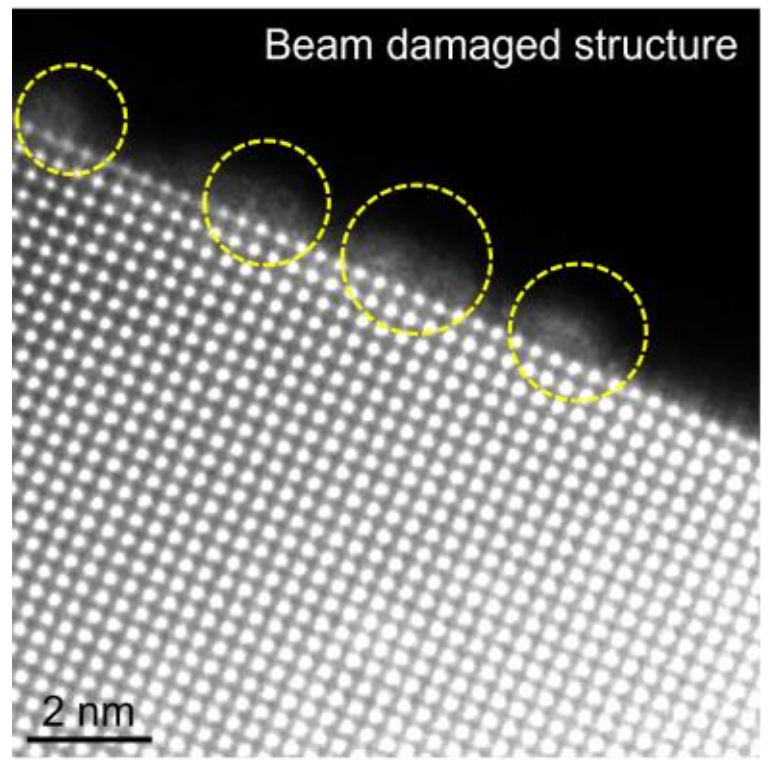

Fig. R24 The contrast-adjusted HAADF-STEM image of PtO_2/SnO_2 after electron beam damage. The yellow circle regions in HAADF-STEM image identify the beam-damaged structure of Pt (small Pt nanoparticles).

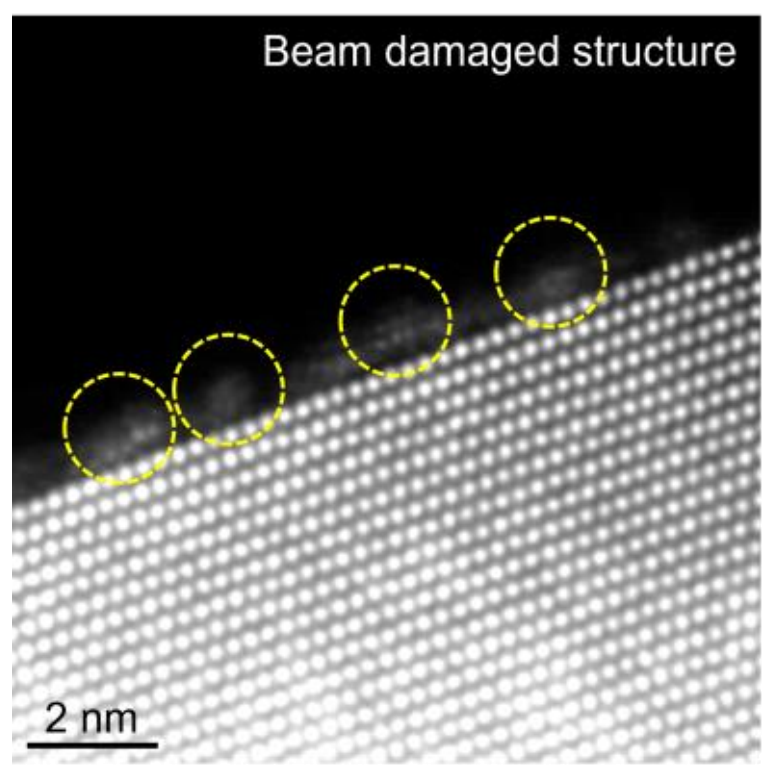

Fig. R25 The contrast-adjusted HAADF-STEM image of PtO_2/SnO_2-used after electron beam damage. The yellow circle regions in HAADF-STEM image identify the beam-damaged structure of Pt (small Pt nanoparticles).

Regarding why the Pt signal appears stronger near the outer edge of the SnO_2 particle in Supplementary Fig. 4c of the original submission (corresponding to Supplementary Fig. 5c in the revised Supplementary Information), we would like to clarify that this mainly arises from the projection geometry of EDS mapping. During EDS acquisition, the beam-sensitive PtO_2 overlayer can restructure into Pt-containing nanoparticle-like species. For the laterally exposed side surface or particle edge, the EDS signal contains the projected contribution from Pt-containing species generated along the side surface over a relatively

long projection length. In contrast, for the projected top surface, only an atomically thin PtO_2 overlayer contributes to the Pt signal before beam-induced restructuring, and thus the amount of Pt-containing species generated along the beam direction is much smaller. Consequently, the Pt signal appears much stronger at the particle edge than on the projected top surface.

This interpretation is further supported by the additional data for the high-loading 8.1 wt% PtO_2/SnO_2 sample provided in our response to Q12. As shown in **Fig. R21c**, an obvious Pt signal can also be observed on the projected top surface of the SnO_2 support in the EDS map. Therefore, the stronger Pt signal near the outer edge in Supplementary Fig. 4c of the original submission (corresponding to Supplementary Fig. 5c in the revised Supplementary Information) does not indicate that Pt is detached from the SnO_2 support or only located at the edge. Rather, it reflects the different projected Pt amounts from the side surface and the top surface in two-dimensional EDS mapping after beam-induced restructuring.

To avoid ambiguity, we have added a clarifying note in the revised Supplementary Information and revised the captions of Supplementary Figs. 4 and 23 of the original submission (corresponding to Supplementary Figs. 5 and 30 in the revised Supplementary Information), to explain the projection effect and the apparent difference between edge and top-surface Pt signals after beam-induced restructuring.

Q15: When showing epitaxial structures where the surface is at an angle different from 90 degrees (as in Figure 5c and other images in the SM) to the SnO_2 lattice planes, the authors need to show the interatomic spacing along the surface, not the way it is shown in Figure 5c.

Response:

We sincerely thank the reviewer for this valuable suggestion. Following the reviewer's

suggestion, we have revised Supplementary Fig. 5c of the original submission (corresponding to Supplementary Fig. 11c in the revised Supplementary Information) by adding line-intensity profiles taken along the surface direction of the epitaxial layer and the underlying support, as shown in **Figs. R26a** and **R26b**. These profiles explicitly quantify the projected interatomic spacing along the surface atomic rows and provide a more accurate comparison between the epitaxial PtO_2 layer and the underlying support.

Figs. R26a and R26b have been added as Supplementary Figs. 11c and 11d, respectively.

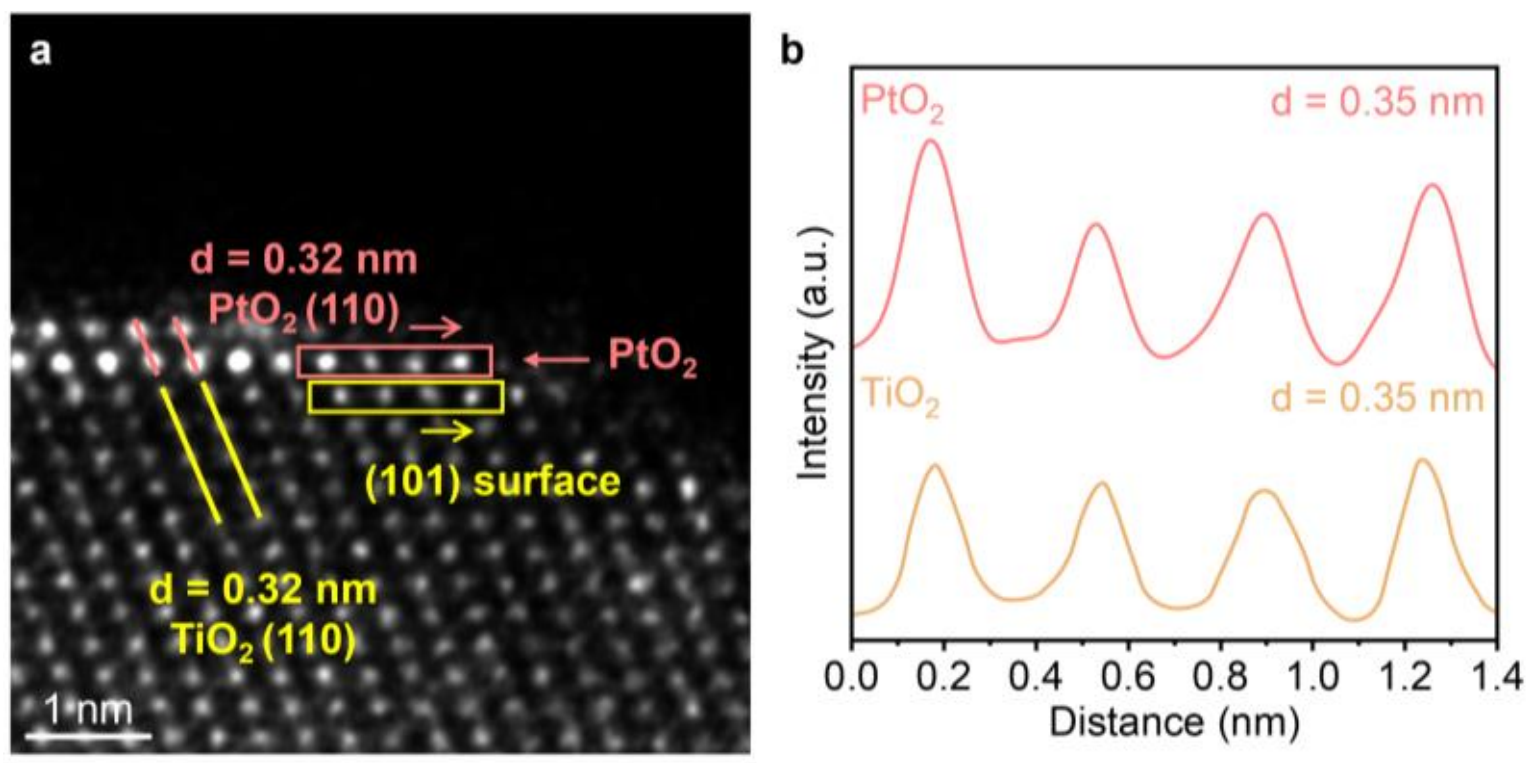

Fig. R26 (a) Atomic-scale HAADF-STEM images of PtO_2/TiO_2 catalyst. (b) Intensity profiles of PtO_2 and TiO_2 taken from the selected red and yellow areas in (a).

In summary, this work shows a catalyst system where deposition of Pt on SnO_2 creates 2-D structures that show improved reactivity for several oxidation reactions. The stability of these structures makes this work of interest to the catalysis of oxidation reactions.

Response:

We also sincerely thank the reviewer for the positive summary and for recognizing the relevance of the stability of these two-dimensional Pt oxide structures to oxidation catalysis. We hope that the additional experiments, analyses, and textual revisions have further strengthened the manuscript.

Reviewer #3 (Remarks to the Author):

This work contains new results and could be considered for publication. However, the authors should revise their manuscript before acceptance for publication in accordance with the following comments.

Q1: To better understand the electronic structures and the chemical active Pt species in PtO_2/SnO_2 and PtO_2/TiO_2, the binding energies of the elements must be indicated.

Response:

We sincerely appreciate the referee's valuable suggestion. To better clarify the electronic structures, we have added the corresponding Pt 4*f* binding-energy values in the revised manuscript on Page 10:

"*The high-resolution Pt 4f spectra of both PtO_2/SnO_2 and PtO_2/TiO_2 (Fig. 2e) can be deconvoluted into four components corresponding to Pt^{2+} $4f_{7/2}$, Pt^{4+} $4f_{7/2}$, Pt^{2+} $4f_{5/2}$, and Pt^{4+} $4f_{5/2}$, located at 72.6 ± 0.1, 74.7 ± 0.1, 75.9 ± 0.1, and 78.0 ± 0.1 eV, respectively.*"

Q2: The authors report for PtO_2/SnO_2 smaller amounts of Pt^{4+} (49.6%), relative to PtO_2/TiO_2 (68.3%), and explain this result, suggesting a strain-induced redistribution of electron density at the Pt centers. However, as SnO_2 and TiO_2 present semiconducting properties, the effect of energy alignment between PtO_2-SnO_2 and PtO_2-TiO_2 must be deeply considered and related to their physical and chemical properties. The same must be applied for RuO_2 and IrO_2 based catalysts.

Response:

We sincerely thank the reviewer for this valuable suggestion. We agree that, because SnO_2 and TiO_2 are semiconducting oxide supports, the possible contribution of interfacial energy alignment and charge transfer should be considered when discussing the electronic

structures of PtO_2/SnO_2 and PtO_2/TiO_2.

To evaluate this possibility, we performed ultraviolet photoelectron spectroscopy (UPS) measurements on the SnO_2 and TiO_2 supports under the same conditions. As shown in **Fig. R27**, the UPS-derived work functions of SnO_2 and TiO_2 are 4.01 and 4.03 eV, respectively, with a very small difference of only 0.02 eV. These values are consistent with reported literature values for SnO_2 and TiO_2 (*Energy Environ. Sci.* 2015, 8, 2928–2934; *J. Solid State Electrochem.* 2023, 27, 1935–1943; *Adv. Sci.* 2018, 5, 1800130). This result suggests that the difference in support-induced interfacial charge transfer arising solely from energy alignment is likely limited. Therefore, although the contribution from interfacial energy alignment cannot be completely excluded, it is unlikely to be the dominant origin of the observed difference in the Pt^{4+} fraction between PtO_2/SnO_2 and PtO_2/TiO_2.

The same consideration has also been added for the RuO_2 and IrO_2 systems. Because these catalysts use the same SnO_2 and TiO_2 supports, the nearly identical work functions of the two supports suggest that support-energy-alignment effects should also be limited in these systems. Therefore, the improved performance of RuO_2/SnO_2 and IrO_2/SnO_2 is more likely associated with epitaxial lattice matching and tensile-strain stabilization.

Fig. R27 has been added as Supplementary Fig. 18, and the corresponding discussion has been added to the revised manuscript on Page 10.

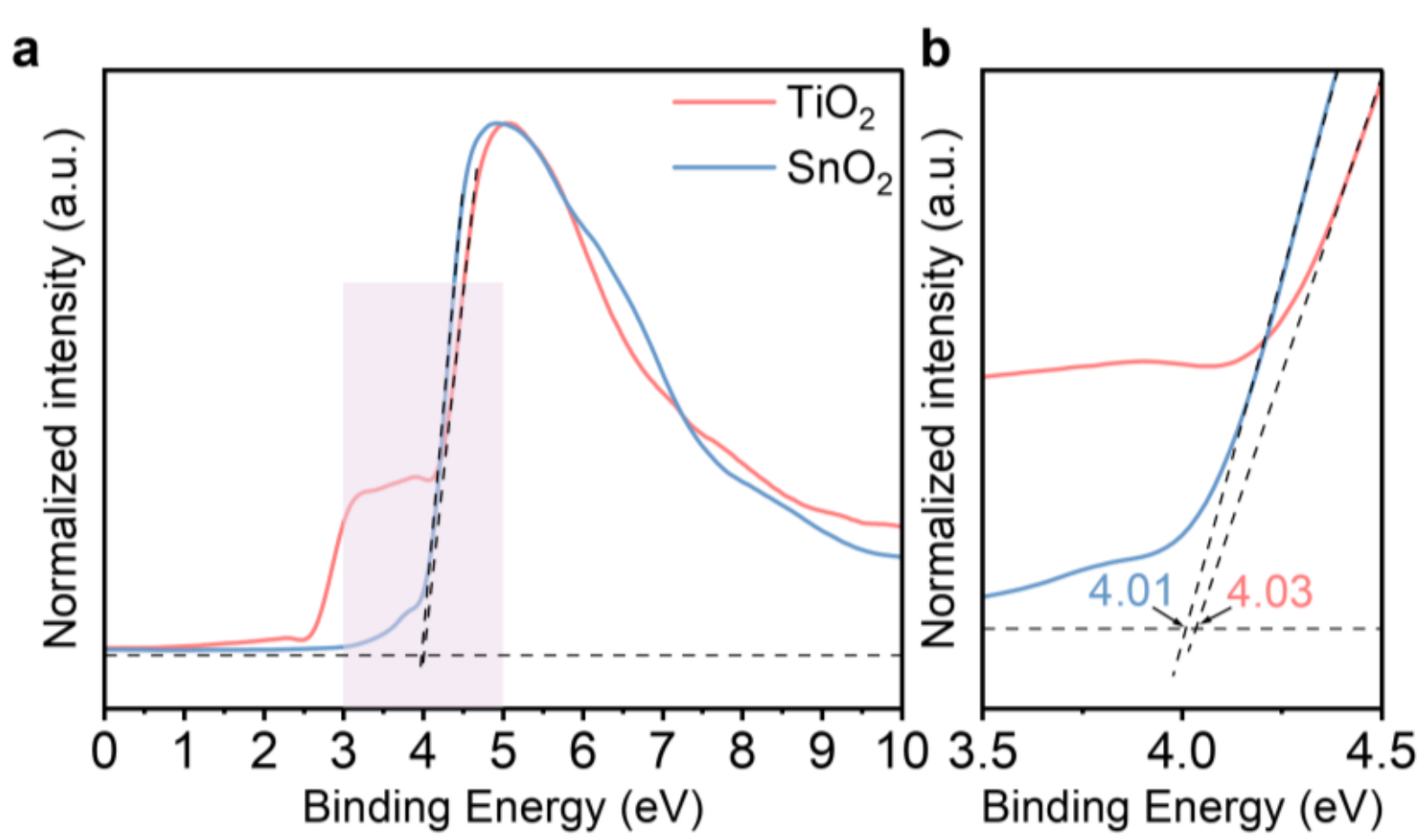

Fig. R27 (a) UPS spectra and (b) UPS-derived work functions of the TiO_2 and SnO_2 supports.

Q3: In Figure 3 (b), the estimated values of the reaction rate of methane oxidation are reported as μmol g_{Pt}^{-1} s^{-1}. However, these values do not represent the real catalytic behavior of PtO_2. Instead reaction rate must be estimated as μmol(surface Pt site)$^{-1}$ s^{-1}.

Response:

We sincerely appreciate the reviewer's valuable suggestion.

Following the reviewer's suggestion, we have added Pt dispersion and site-normalized TOF values to the revised catalytic comparison table. For the 0.73 wt% PtO_2/SnO_2 catalyst, the Pt loading is well below the theoretical PtO_2 monolayer dispersion threshold of 1.95 wt%. Based on the atomically thin PtO_2 overlayer model, the Pt atoms in this catalyst are considered to be exposed surface Pt sites, with an estimated Pt dispersion of 100%. The corresponding TOF values were calculated using the total Pt amount as the number of accessible Pt sites. The calculated TOF values are 3.00×10^{-3} s^{-1} at 260 °C and 1.70×10^{-2} s^{-1} at 300 °C, respectively.

However, for catalysts with Pt loadings above the theoretical monolayer threshold, the number of exposed Pt sites cannot be accurately determined from the nominal Pt loading because additional Pt species may form multilayered PtO_2 structures. Therefore, to avoid unsupported assumptions, we do not calculate site-normalized TOF values for these high-loading samples. The Pt-mass-normalized rates in Fig. 3b are retained only to show the loading-dependent activity trend.

The revised comparison, including Pt loading, Pt dispersion, Pt-mass-normalized rate and TOF, has been added as Supplementary Table 5 in the revised Supplementary Information and is presented below as **Table R5**.

Table R5 Comparison of catalytic performance in lean-burn CH_4 oxidation for reported Pt-based catalysts.

Sample	Pt loading (wt%)	Feed gas composition	WHSV (mL g^{-1} h^{-1})	T_{90} (°C)	Reaction rate (μmol g_{Pt}^{-1} s^{-1})	Pt dispersion (%)	TOF (s^{-1})	Ref.
**PtO_2/SnO_2**	**0.73**	**1% CH_4, 20% O_2, N_2**	**30,000**	**370**	**15.4 (260 °C)**<br>**87.1 (300 °C)**	**100**	**3.00×10^{-3} (260 °C)**<br>**1.70×10^{-2} (300 °C)**	**This work**
Pt/ZrO_2	1.0	0.2% CH_4, 10% O_2, He	30,000	425	3.2 (300 °C)	-	-	1
SO_2 treated Pt/CeO_2	4	0.05% CH_4, 8% O_2, Ar	15,000 h^{-1}	>500	4.6 (300 °C)	-	-	2
Pt/δ-Al_2O_3 (N0)	1.5	1% CH_4, 4% O_2, He	-	485	12.2 (300 °C)	95	2.00×10^{-3} (300 °C)	3
Pt/Alumina	1.5	0.3% CH_4, 16% O_2, He	78,000	-	15.4 (300 °C)	81	3.70×10^{-3} (300 °C)	4
Pt_1/CF-W	1.0	0.5% CH_4, 20% O_2, N_2	20,000	386	83.6 (300 °C)	100	1.63×10^{-2} (300 °C)	5
Pt/γ-Al_2O_3	1	0.2% CH_4, 10% O_2, N_2	30,000	510	0.1 (310 °C)	37	5.83×10^{-5} (310 °C)	6
Pt/Cr_2O_3	1	0.2% CH_4, 10% O_2, N_2	30,000	390	6.8 (310 °C)	93	1.55×10^{-3} (310 °C)	6
Pt/rutile-400	0.56	1% CH_4, 20% O_2, He	36,000	~390	35.9 (310 °C)	22.5	3.11×10^{-2} (310 °C)	7
0.5H_2O_2-Pt_1/R-Mn_2O_3-5D	0.82	1% CH_4, 18% O_2, He	20,000	605	24.1 (400 °C)	100	4.70×10^{-3} (400 °C)	8
Pt@CeO_2 (13.8 nm)	1.0	1% CH_4, 2% O_2, N_2	555,555	600	686.9 (450 °C)	-	-	9
Pt/Al_2O_3	1.8	0.2% CH_4, 10% O_2, N_2	400,000	>625	20.5 (525 °C)	-	-	10

1Pt/Fe_2O_3-C700	1.9	0.5% CH_4, 3% O_2, He	90,000	>700	558 (700 °C)	100	0.1 (700 °C)	11
Pt/r-TiO_2	2.01	1% CH_4, air	36,000	382	-	76.87	-	12
Pt/SnO_2	1.0	0.2% CH_4, 10% O_2, N_2	60,000	391	-	-	-	13
Pt/CeO_2/3DOM LF	1.0	1% CH_4, 20% O_2, Ar	45,000	502	-	-	-	14

Response to reviewers' comments (NCOMMS-26-009959A)

REVIEWER COMMENTS

Reviewer #1 (Remarks to the Author):

The authors tried to address the questions raised by the reviewer. I do not have further comments about the revision version of this manuscript. Thanks for providing me the opportunity to read this work. It can be accepted considering the improved quality of the work.

Response:

We sincerely thank the reviewer for the positive assessment of our revised manuscript and for the constructive comments throughout the review process.

Reviewer #2 (Remarks to the Author):

The authors have done considerable additional work and addressed all of the comments. The work is now suitable for publication.

Response:

We sincerely thank the reviewer for the positive assessment of our revised manuscript. We greatly appreciate the reviewer's constructive comments and suggestions, which have helped us substantially improve the quality of the manuscript.

Reviewer #3 (Remarks to the Author):

I have carefully examined the authors' responses to my previous comments and the revised version of the manuscript.

The authors have satisfactorily addressed my major concerns. In particular:

1. Regarding the different Pt^{4+} fractions observed for PtO_2/SnO_2 and PtO_2/TiO_2, the authors have now provided additional experimental evidence by comparing the band gaps of the two supports. The results indicate no significant difference in their electronic structures from this perspective, supporting their interpretation. Similar considerations have also been appropriately extended to the RuO_2 and IrO_2 based catalysts.

2. The authors have also adequately addressed my concern regarding the normalization of the methane oxidation rates. Their explanation and the corresponding revisions clarify the catalytic activity assessment and satisfactorily resolve this issue.

Overall, I consider that the authors have responded appropriately to my comments, and I believe that the revised manuscript is now suitable for publication.

Response:

We sincerely thank the reviewer for the careful evaluation of our revised manuscript and for the positive assessment. We greatly appreciate the thoughtful comments and suggestions provided throughout the review process.
